# Total syntheses of shizukaols A and E

**DOI:** 10.1038/s41467-018-06245-7

**Published:** 2018-10-02

**Authors:** Jian-Li Wu, Yin-Suo Lu, Bencan Tang, Xiao-Shui Peng

**Affiliations:** 1 Department of Chemistry, and State Key Laboratory of Synthetic Chemistry, The Chinese University of Hong Kong, Shatin, New Territories Hong Kong SAR, China; 2 Innovative Drug R&D Center, Shenzhen Salubris Pharmaceuticals Co., LTD., Baoan District Shenzhen, 518040 China; 3 Department of Chemical and Environmental Engineering, The University of Nottingham Ningbo China, Zhejiang, 315100 China; 4 Shenzhen Municipal Key Laboratory of Chemical Synthesis of Medicinal Organic Molecules, Shenzhen Research Institute, The Chinese University of Hong Kong, Shenzhen, 518507 China

## Abstract

Shizukaols possess a common heptacyclic framework containing more than ten contiguous stereocenters and potential biological activities. Here we report that the total syntheses of shizukaols A (**1**) and E (**2**), two lindenane-type dimers from the Chloranthaceae family, are achieved via a modified biomimetic Diels–Alder reaction. The common crucial biomimetic diene **23** and ethylene species (**6**, **17**) are obtained through either a highly *Z*-selective olefination of α-siloxy ketone with ynolate anions or an intramolecular Horner–Wadsworth–Emmons olefination from commercially available Wieland–Miescher ketone (**7**). This synthetic approach here opens up practical avenues for the total syntheses of the intriguing Chloranthaceae family members, as well as the understanding of their relevant biological action in nature.

## Abstract (web summary)

Shizukaols, bioactive dimers naturally occurring in the Chloranthaceae family, possess a complex polycyclic framework with more than ten contiguous stereocenters. Here the authors report the total syntheses of shizukaols A and E achieved via a modified Diels–Alder reaction mimicking the biosynthetic pathway.

## Introduction

**Fig. 1:**
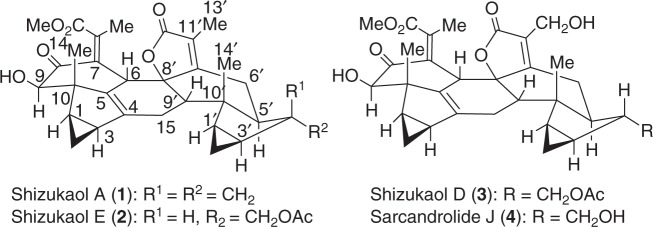
Structures of dimeric lindenane-type representives. Shizukaol family possesses a common heptacyclic framework with >10 contiguous stereocenters

**Fig. 2:**
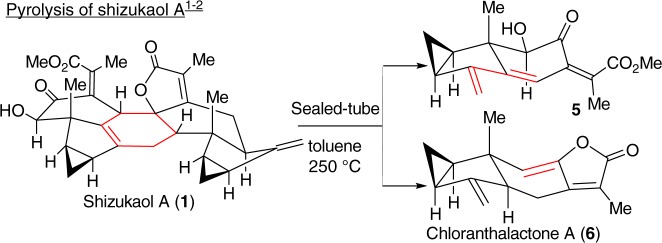
Pyrolysis of shizukaol A. Shizukaol A was pyrolysized to afford relatively unstable diene **5** and known chloranthalactone A (**6**) via a retro Diels–Alder fragmentation of the linking six-membered ring in a sealed tube

In 1990, shizukaol A (**1**) (Fig. 1) was isolated from *Chloranthus japonicus*^1,2^ as the first dimeric lindenane-type sesquiterpenoid. Subsequently, shizukaol E (**2**) (Fig. 1) was successively isolated from *Chloranthus japonicus* in 1995^3,4^. To date, >110 congeners with a similar polycyclic core have been identified from the Chloranthaceae family^5–7^. These molecules possess a common heptacyclic framework containing >10 contiguous stereocenters. Noteworthy is that shizukaol E (**2**) showed potential anti-HIV-1 replication activities and significant inhibition effects against the currently most popular NNRTI-resistant HIV-1, together with the inhibitory activities on HCV replication, making it attractive as a potential therapy against the co-infection of HIV-1 and HCV^8^. In addition, most of these dimeric lindenane-type molecules show impressive bioactivities, such as remarkable cytotoxicity^9^, inhibition of the delayed rectifier K^+^ current^10,11^, and antimalarial activities^12^. Therefore, the promising bioactivities and synthetic challenges arising from these unprecedented molecules have initiated a significant interest to synthetic chemists^13–30^. However, only one total synthesis of two dimeric members^30^ and a number of relevant synthetic studies^13–29^ have been achieved. In 2017, Liu and coworkers reported the first total syntheses of the dimeric shizukaol D (**3**) and sarcandrolide J (**4**)^30^ through a cascade protocol featuring furan formation/alkene isomerization/Diels–Alder reaction. As shown in Fig. 2, the pyrolysis of shizukaol A (**1**) in a sealed tube resulted in a retro Diels–Alder fragmentation of the linking six-membered ring to furnish two products: the relatively unstable diene **5** and the previously known chloranthalactone A (**6**)^1,2^. This led to a biogenetic hypothesis for the lindenane dimeric family in which enzymatic *endo*-Diels–Alder reaction of two lindenane-type components (such as **5** and **6**) forms a common linking six-membered ring. Based on this hypothesis, we have illustrated a practical strategy for constructing the heptacyclic framework of the Chloranthaceae family via an *endo-*Diels–Alder reaction as the pivotal step, as shown in Fig. 2^28,29^. Encouraged by our preliminary results of *endo-*Diels–Alder reaction^28,29^, herein we accomplished the total syntheses of two members of this dimeric family, shizukaols A (**1**) and E (**2**), through mimicking a biosynthetic approach.

## Results

### Retrosynthetic analysis

**Fig. 3:**
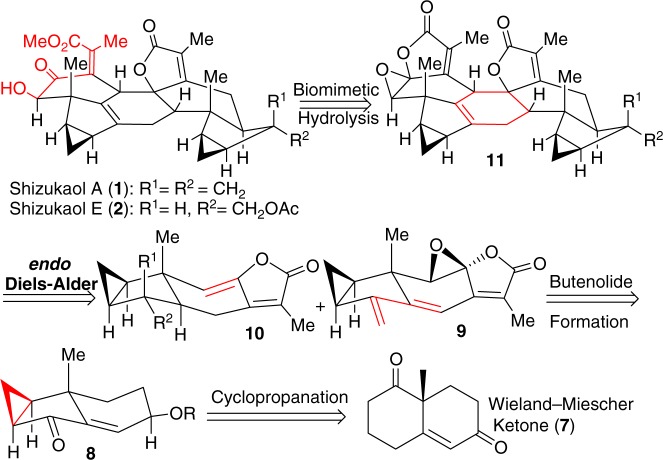
Retrosynthetic analysis toward shizukaols A and E. This proposed protocol is featuring with a modified biomimetic approach involving an *endo-*Diels–Alder reaction and biomimetic transformation

Inspired by our preliminary investigations^28,29^ on *endo-*Diels–Alder reaction to generate heptacyclic framework of the Chloranthaceae family, we considered to design more suitable precusors, being at least biogenetically much closer to components **5** and **6** for further transformation. Inspection of the structures of shizukaols A (**1**) and E (**2**) reveals a linking six-membered ring and a hydroxyl ketoester moiety to be the common structural features of many dimeric lindenane-type molecules. In consideration of the likely instability of **5**, as well as the long synthetic steps in the conversion of our preliminary [4+2] cycloaddition products to shizukaol A (**1**), we reason that the synthesis of a crucial heptacyclic core with suitable functional groups for subsequent conversions to naturally occurring shizukaols A (**1**) and E (**2**) should be a more practical approach. Moreover, our preliminary investigation proved that the late-stage conversion of the epoxy unit in **9** (Fig. 3) to the hydroxyl ketoester unit of shizukaols A (**1**) and E (**2**), a common framework of the dimeric family members, is a more step-economic strategy for total syntheses. In addition, the epoxide moiety in triene **9** is expected to prevent a further [4+2] cycloaddition of relevant alkenyl unit. Therefore, on basis of these considerations and the aforementioned biogenetic hypothesis, we think that the critical step for the construction of skeleton relevant to that of shizukaols A (**1**) and E (**2**) would then be the expedient and pragmatic conversion of readily accessible precursor **10** and biomimetic epoxide **9** into the *endo-*Diels–Alder reaction product **11** via the relevant transition state. Upon *endo-*Diels–Alder reaction of **9** and **10**, epoxy compound **11** could readily be obtained which then undergoes a biomimetic transformation of epoxy unit to the common hydroxyl ketoester species of the shizukaol family, eventually achieving shizukaols A (**1**) and E (**2**).

### Total syntheses of shizukaols A and E

**Fig. 4:**
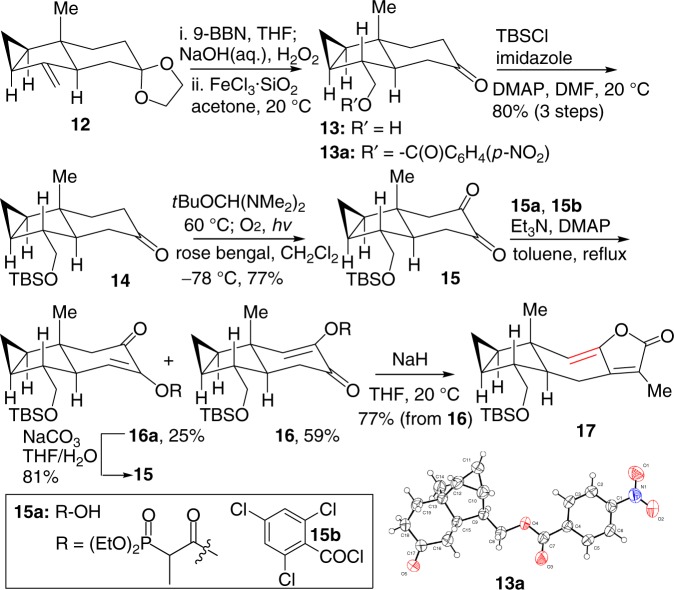
Synthesis of ethylene 17. 9-BBN 9-borabicyclo[3.3.1]nonane, TBS tert-butyldimethylsilyl, DMAP 4-dimethylaminopyridine, DMP Dess–Martin periodinane

Based on the aforementioned synthetic strategy, we then commenced the synthesis of ethylene **17** from known compound **12** (Fig. 4)^28,29^, which was obtained through the sequence of classical Mitsunobu reaction^31,32^, nickel-mediated reduction^33,34^, and modified Julia–Kocienski olefination (see Supporting Information for details)^35^. Compound **12** was then treated with 9-BBN to form an inseparable mixture of diastereoisomers in a ratio of 8:1 (*α*/*β*). Deprotection with FeCl_3_•SiO_2_ led to alcohol **13** with an α-hydroxymethyl group as the major product. It is noteworthy that Zhao and coworkers reported the synthesis of the single stereoisomer of **13** with β-hydroxymethyl group under a similar hydroboration condition^19^. Therefore, in order to further identify the stereochemistry, protection of hydroxyl group in **13** with *p-*nitrobenzoyl group provided compound **13a** in a quantitative yield. After many trials, compound **13a** gave finally satisfactory single crystals from CH_2_Cl_2_, whose X-ray diffraction study convincingly showed an α-hydroxymethyl group in **13a** to be consistent with its relevant orientation in shizukaol E (**2**). The stereoselectivity of this hydroboration is presumably caused by the presence of 1,3-*syn*-diaxial repulsion in alkene **12**. Subsequent protection of the primary alcohol with a TBS group gave ketone **14**, which reacted with Bredereck’s reagent and singlet oxygen successfully to form dione **15** in 77% yield^36,37^. The resulting dione **15** underwent Yamaguchi esterification^38^ with commercially available 2-(diethoxyphosphoryl)propanoic acid (**15a)** and 2,4,6-trichlorobenzoyl chloride (**15b**) to furnish an easily separable mixture of **16** and its regioisomer **16a** in 59% and 25% yields, respectively. The undesired regioisomer **16a** could be recycled into dione **15** in 81% yield in the presence of Na_2_CO_3_. Then, treatment of **16** with NaH in THF led to the desired ethylene **17** in 77% yield.

**Fig. 5:**
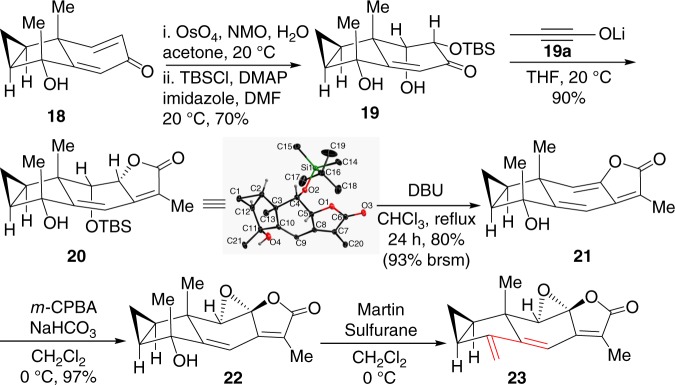
Synthesis of diene **23**. NMO *N*-methylmorpholine *N*-oxide, TBS *tert*-butyldimethylsilyl, DMAP 4-dimethylaminopyridine, DBU 1,8-diazabicyclo[5.4.0]undec-7-ene, and *m*-CPBA *meta*-chloroperbenzoic acid

With ethylene **17** in hand, we turned our attention to the synthesis of diene **23** from known enone **18**^28,29^. As shown in Fig. 5, regio- and stereoselective dihydroxylation of enone **18** in the presence of a catalytic amount of OsO_4_ and one equivalent of NMO afforded exclusively the corresponding diol from the less-hindered α-face. Upon regioselective protection of the less sterically hindered secondary alcohol with a TBS group, α-siloxy ketone **19** was furnished in 70% yield. The regioselectivity of silylation could be ascribed to the steric hindrance of the angular methyl and the bulky silyl group. Next, treatment of α-siloxy ketone **19** with freshly prepared ynolate **19a** led to a highly *Z*-selective olefination of **19** under a mild condition^39–41^. As a result, butenolide **20** was smoothly generated via a spontaneous silyl migration and lactonization. Moreover, the stereochemistry of **20** was confirmed through an X-ray crystallographic analysis of a single crystal of butenolide **20** grown from CH_2_Cl_2_. Lactone **20** was then allowed to undergo elimination of OTBS with DBU to generate **21** in 80% yield, which increased to 93% yield after recovery of **20**. As shown in Fig. 3, we predicted that β-epoxide would facilitate subsequent transformation. Lamentably, we failed to form directly the β-epoxide from alkene **21** after considerable testing of epoxidation protocols. Similarly, we failed to convert the α-epoxide of compound **22** into β-epoxide after extensive efforts. We therefore planned to reverse its relevant stereochemistry at a later stage. Epoxidation of **21** with *m*-CPBA led exclusively to α-epoxide **22**. Then, dehydration of **22** with Martin sulfurane smoothly gave rise to triene **23**^42,43^, which was very unstable and easy to dimerize in its neat form or undiluted solution. Fortunately, diene **23** was relatively stable when diluted in toluene or xylene and stored at −20 °C for immediate use without obvious dimerization or polymerization.

**Fig. 6:**
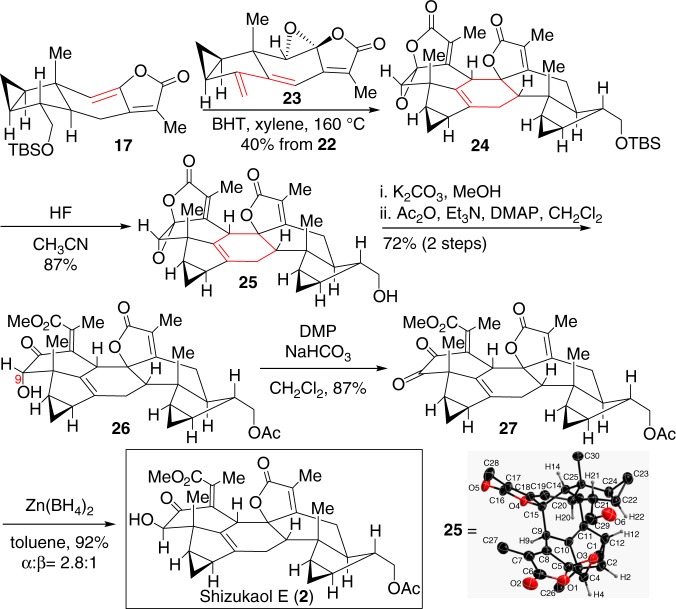
Total synthesis of shizukaol E (**2**). BHT butylated hydroxytoluene, DMP Dess–Martin periodinane, DMAP 4-dimethylaminopyridine

Next, we investigated the possibility of an *endo*-Diels–Alder reaction between electron-deficient electrophilic diene **23** and electron-rich C8′–C9′ double bond of nucleophilic ethylene **17**^44–46^. As shown in Fig. 6, treatment of compounds **17** and **23** with butylated hydroxytoluene (BHT) at 160 °C in xylene successfully led to the *endo-*Diels–Alder reaction cycloadduct **24** as a single detectable isomer. Noteworthy, the homo-dimerization product of diene **23** was observed as the major by-product of this Diels–Alder reaction. Therefore, in order to reduce the homo-dimerization of diene **23**, it is essential for *endo*-Diels–Alder reaction that a high dilute solution of **23** was added into a refluxing dilute solution of **17** very slowly. Then, removal of the silyl group in **24** led to the crucial alcohol **25** in 87% yield as a solid. After many trials, alcohol **25** gave eventually satisfactory single crystals from CH_2_Cl_2_. Its X-ray diffraction study confirmed that alcohol **25** was the desired *endo-*Diels–Alder reaction product. However, efforts to improve this Diels–Alder reaction using Lewis acid/base catalysts caused decomposition of the sensitive substrates. Therefore, this thermal Diels–Alder reaction appears to be an acceptable approach for generating **24** as a single detectable dimer. With alcohol **25** in hand, it was then allowed to undergo hydrolysis with K_2_CO_3_ to generate the corresponding diol, thereby installing the crucial hydroxyl ketoester moiety of the natural product family in a biomimetic transformation. Selective acetylation of the primary alcohol then provided monoacetate **26** in 72% yield in two steps. Next, efforts to invert the secondary α-hydroxyl group in **26** were unsuccessful, probably because the hydroxy group at C9 of **26** was sterically hindered. Even the corresponding mesylate and triflate, which were easily epimerized, did not give positive results when treated with alkaline reagents such as DBU and KNO_2_^47^. Therefore, oxidation of alcohol **26** with Dess–Martin periodinane led to the dione **27**. Treatment of **27** with Zn(BH_4_)_2_ eventually gave the synthetic shizukaol E (**2**) with a C9 β-hydroxyl group, together with recyclable compound **26** in 92% yield with an *α*/*β* ratio of 2.8:1 (see Supplementary Table 3). The synthetic shizukaol E (**2**) was fully characterized, and its NMR spectroscopic data are identical to those reported in the literature^3,4^. However with the exception of Zn(BH_4_)_2_, other reductive reagents such as NaBH_4_, L-selectride, etc. resulted in much lower stereoselectivity than that of Zn(BH_4_)_2_. Some reducing reagents such as LiAl(*t*BuO)_3_H even led only to compound **26** with α-selectivity. Presumably, the stereoselectivity of this reduction results from coordination between Zn(BH_4_)_2_ and the two carbonyl groups at C8 and C9 in dione **27**. The relevant DFT calculations of the reduction of dione **27** with Zn(BH_4_)_2_ was performed using Gaussian 09 to understand the observed stereoselectivity (see Supplementary Table 12 and Supplementary Figure 4).

**Fig. 7:**
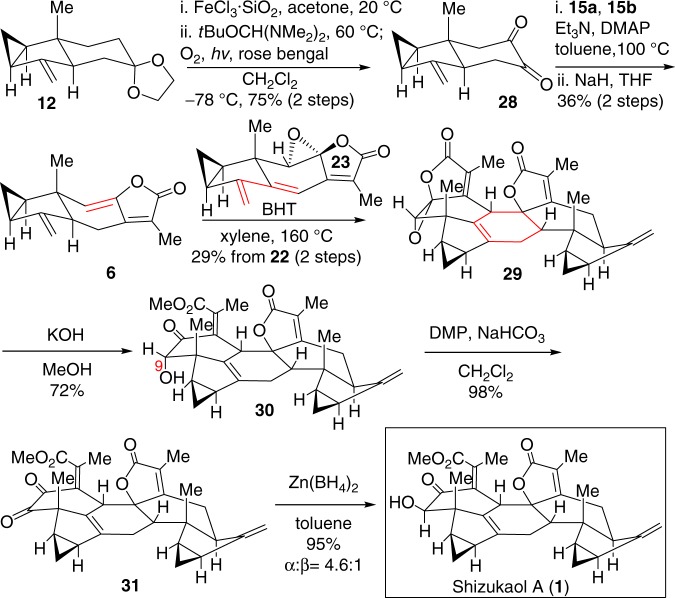
Total synthesis of shizukaol A (**1**). DMAP 4-dimethylaminopyridine, BHT butylated hydroxytoluene, DMP Dess–Martin periodinane

With the completion of the total synthesis of shizukaol E (**2**), we then concerned on the total synthesis of shizukaol A (**1**). As shown in Fig. 7, when ethylene glycol in compound **12** was removed with FeCl_3_•SiO_2_ in acetone, the resulting ketone underwent a conversion similar to the transformation of **14** to **15**. Subsequent sun-lamp irradiation afforded exclusively dione **28** in 75% overall yield for two steps. Upon a similar procedure as described above, regioselective Yamaguchi esterification of dione **28** followed by intramolecular Horner–Wadsworth–Emmons olefination^48^ finally gave rise to chloranthalactone A (**6**) as nucleophilic ethylene for Diels–Alder reaction. During workup and purification process, **6** was partially oxidized to the respective epoxide in air. Then, exposure of **6** and **23** under a thermal condition provided exclusively the desired *endo*-Diels–Alder cycloadduct **29**. To better understand the observed stereochemical outcome of the *endo*-Diels–Alder reaction between diene **23** and ethylene species **17** or **6**, we carried out computational studies of diene **23** and ethylene **6** using Gaussian 09^49^, and the relevant results were consistent with our experimental observations (see Supporting Information for details). Subsequently, a biomimetic hydrolysis with KOH in MeOH gave compound **30** in 72% yield. Oxidation of **30** with Dess–Martin periodinane followed by reduction of dione **31** with Zn(BH_4_)_2_, eventually achieved shizukaol A (**1**) and alcohol **30** in 95% yield with an *α*/*β* ratio of 4.6:1. The spectroscopic data of synthetic shizukaol A (**1**), including MS, IR, and NMR, are in full agreement with those reported^1,2^.

## Discussion

In summary, we achieved the first total syntheses of shizukaols A (**1**) and E (**2**) from commercially available Wieland–Miescher ketone (**7**) through mimicking the modified biogenetic pathway in 0.1% overall yield over 24 steps and 0.15% overall yield over 28 steps in the longest linear sequence, respectively. According to the structural features of the shizukaol family, an efficient butenolide formation was developed for the synthesis of the common biomimetic diene **23** and ethylene species (**6** and **17**) using either a highly *Z*-selective olefination of α-siloxy ketone with ynolate anions or an intramolecular Horner–Wadsworth–Emmons olefination. Inspired by the hypothetical biogenetic pathway for the shizukaol family and the pyrolysis results, we proposed and validated a modified biomimetic approach involving an *endo-*Diels–Alder reaction and biomimetic transformation. This methodology may be practical for the syntheses of other members in the intriguing Chloranthaceae family, as well as other potential lead molecules for understanding of their relevant biological action and biogenetically synthetic protocol in nature.

## Methods

### General

All reagents and solvents were of reagent grade. Further purification and drying following the guidelines of Perrin and Armarego^50^ were used when necessary. Organic solvents were concentrated under reduced pressure on a rotary evaporator in a water bath no more than 40 ℃ unless otherwise specified. Thin-layer chromatography (TLC) was performed on E. Merck silica gel 60 F254 (0.25 mm thickness) coated on aluminum plates. Chromatographic purification of products was performed on Macherey–Nagel–Kieselgel 60 M (230–400 mesh). Visualization of the developed chromatogram was performed by acidic ceric ammonium molybdate and subsequent heating. Melting points were measured with a Stuart Melting Point Apparatus (SMP40) in Celsius degrees and were uncorrected. Nuclear magnetic resonance (NMR) spectra were recorded with a Bruker ADVANCE-III NMR spectrometer at 400.13 MHz (^1^H) or at 100.6 MHz (^13^C). All NMR measurements were carried out in CDCl_3_ and internally referenced to residual solvent signals (referenced at δ 7.26 ppm in ^1^H, and δ 77.16 ppm for central line of the triplet in ^13^C). Data for ^1^H NMR are reported as follows: chemical shift (δ ppm), multiplicity (s = singlet, d = doublet, t = triplet, q = quartet, brs = broad singlet, dd = doublet of doublets, dt = doublet of triplets, td = triplet of doublets, ddd = doublet of doublets of doublets, m = multiplet), integration, coupling constant (Hz), and assignment. Data for ^13^C NMR are reported in terms of chemical shift. Mass spectrometry (MS) and high-resolution mass spectrometry (HRMS) were measured on a ThermoFinnigan MAT 95XL. Elemental analyses were carried out by Shanghai Institute of Organic Chemistry, the Chinese Academy of Science, PRC. Selected crystals for X-ray analyses were used for intensity data collection on either a Bruker AXS Kappa Apex II Duo diffractometer or Bruker D8 Venture X-Ray Diffractometer at 173 K using frames of oscillation range 0.3°, with 2° < *θ* < 28°. Infrared spectra (IR) were recorded on a Nicolet 420 FT-IR spectrometer as thin film on potassium bromide discs.

## Electronic supplementary material

Supplementary Data 1

Supplementary Information

Description of Additional Supplementary Files

## Data Availability

The X-ray crystallographic coordinates for structures reported in this article have been deposited at the Cambridge Crystallographic Data Centre (CCDC) under deposition numbers CCDC 1827569 for compound **13a**, CCDC 1833455 for compound **20**, and CCDC 1833456 for compound **25**. These data are provided free of charge by The Cambridge Crystallographic Data Centre. These data can be obtained free of charge from the Cambridge Crystallographic Data Centre via http://www.ccdc.cam.ac.uk/data_request/cif.
