# Supplementary material for: Total syntheses of shizukaols A and E
Source: Nat Commun. 2018 Oct 2;9:4040. doi: 10.1038/s41467-018-06245-7 (PMC6168560; doi:10.1038/s41467-018-06245-7)
Supplement: Supplementary file 1 — Supplementary Data 1 [file 41467_2018_6245_MOESM1_ESM.docx]

**Cartesian Coordinate**

**Diene 23**

| C | -2.49086 | 1.250693 | 0.292426 |
| --- | --- | --- | --- |
| C | -1.14061 | 0.721925 | -0.00397 |
| C | -1.25834 | -0.75602 | -0.38029 |
| C | -2.60722 | -1.17092 | 0.234747 |
| C | -3.35489 | 0.075659 | 0.646358 |
| H | -2.61941 | -2.05259 | 0.867827 |
| H | -3.9259 | 0.117157 | 1.567861 |
| C | -1.20332 | -0.90692 | -1.91315 |
| H | -0.19982 | -0.66411 | -2.27765 |
| H | -1.44559 | -1.93436 | -2.20453 |
| H | -1.90669 | -0.2334 | -2.40759 |
| C | -3.86772 | -0.78288 | -0.47985 |
| H | -3.81669 | -0.39542 | -1.49172 |
| H | -4.75289 | -1.37531 | -0.27205 |
| C | -0.09597 | -1.56721 | 0.158747 |
| C | 0.028761 | 1.369833 | 0.150133 |
| H | 0.054533 | 2.425468 | 0.40853 |
| C | 1.261631 | 0.608099 | 0.109178 |
| O | 0.368019 | -1.25816 | 1.474885 |
| C | 2.558366 | 0.89171 | -0.09127 |
| C | 3.310461 | -0.38913 | 0.002857 |
| O | 4.48113 | -0.58449 | -0.14695 |
| H | -0.05946 | -2.62548 | -0.09985 |
| C | 3.2625 | 2.165503 | -0.40668 |
| H | 2.562601 | 3.001445 | -0.46924 |
| H | 4.012025 | 2.385775 | 0.359892 |
| H | 3.796441 | 2.079306 | -1.35837 |
| C | 1.155373 | -0.85178 | 0.390929 |
| O | 2.418417 | -1.41116 | 0.271297 |
| C | -2.8591 | 2.532953 | 0.290682 |
| H | -3.86885 | 2.828803 | 0.556372 |
| H | -2.16523 | 3.324069 | 0.021519 |

**6**

| C | 2.447555 | 1.158743 | -0.41249 |
| --- | --- | --- | --- |
| C | 1.24306 | -0.81317 | 0.218988 |
| C | 2.530564 | -1.25795 | -0.50819 |
| C | 3.290328 | 0.000671 | -0.88533 |
| H | 2.462513 | -2.09174 | -1.2004 |
| H | 3.793186 | 0.090463 | -1.84359 |
| C | 1.422436 | -0.76171 | 1.751055 |
| H | 0.468346 | -0.55372 | 2.242831 |
| H | 1.788223 | -1.72391 | 2.122306 |
| H | 2.133297 | 0.016242 | 2.048672 |
| C | 3.85888 | -0.9576 | 0.124932 |
| H | 3.908121 | -0.65983 | 1.165943 |
| H | 4.702045 | -1.55608 | -0.20552 |
| C | 0.007776 | -1.6314 | -0.07964 |
| C | -1.32786 | 0.474085 | 0.100166 |
| C | -2.64469 | 0.735034 | 0.041566 |
| C | -3.34244 | -0.55524 | -0.18829 |
| O | -4.51302 | -0.7767 | -0.31019 |
| H | 0.049641 | -2.71045 | -0.19651 |
| C | -3.40977 | 2.008081 | 0.153298 |
| H | -2.74136 | 2.870429 | 0.207795 |
| H | -4.07744 | 2.126866 | -0.70554 |
| H | -4.04109 | 1.998962 | 1.047813 |
| C | -1.15633 | -0.97484 | -0.08751 |
| O | -2.38816 | -1.55411 | -0.2591 |
| C | 2.851398 | 2.383056 | -0.08958 |
| H | 3.89714 | 2.670088 | -0.1447 |
| H | 2.145915 | 3.143982 | 0.234198 |
| C | 1.044574 | 0.612453 | -0.37479 |
| H | 0.776431 | 0.434541 | -1.42791 |
| C | -0.12153 | 1.362301 | 0.256359 |
| H | -0.29271 | 2.328549 | -0.23151 |
| H | 0.073436 | 1.566947 | 1.317159 |

**C9'*Si*-TS-*endo*-*Re***

| C | -0.06905 | 1.705526 | -1.07975 |
| --- | --- | --- | --- |
| C | -0.10732 | 2.840857 | -0.05355 |
| C | 1.376423 | 3.202053 | 0.120428 |
| C | 2.196949 | 2.364952 | -0.83939 |
| H | 1.742752 | 3.411374 | 1.121188 |
| H | 3.163115 | 1.95886 | -0.56183 |
| C | -0.95781 | 4.02094 | -0.56328 |
| H | -2.00157 | 3.713653 | -0.68443 |
| H | -0.91951 | 4.851658 | 0.149536 |
| H | -0.60079 | 4.383648 | -1.52865 |
| C | 2.069447 | 3.853635 | -1.04108 |
| H | 1.484318 | 4.20347 | -1.88552 |
| H | 2.944107 | 4.456594 | -0.81915 |
| C | -0.7973 | 2.371957 | 1.217351 |
| C | -1.14276 | 0.871356 | -1.30326 |
| H | -1.19826 | 0.217765 | -2.1673 |
| C | -2.17255 | 0.82739 | -0.31608 |
| O | -0.59247 | 1.010119 | 1.600958 |
| C | -3.4065 | 0.278944 | -0.24475 |
| C | -3.89762 | 0.488469 | 1.142816 |
| O | -4.93737 | 0.154965 | 1.632066 |
| H | -0.87398 | 3.072817 | 2.048509 |
| C | -4.28084 | -0.34124 | -1.27409 |
| H | -3.80032 | -0.3429 | -2.25463 |
| H | -4.51423 | -1.37998 | -1.01715 |
| H | -5.22825 | 0.204763 | -1.33423 |
| C | -1.83425 | 1.35349 | 1.0405 |
| O | -2.94111 | 1.192243 | 1.858351 |
| C | 3.03674 | -0.5258 | 1.78611 |
| C | 2.48617 | -1.4666 | -0.36452 |
| C | 3.808614 | -0.67941 | -0.50008 |
| C | 4.142642 | -0.09602 | 0.859687 |
| H | 3.99288 | -0.10966 | -1.40429 |
| H | 4.555893 | 0.902637 | 0.973327 |
| C | 2.759263 | -2.97264 | -0.15926 |
| H | 1.832176 | -3.55158 | -0.19139 |
| H | 3.423905 | -3.34977 | -0.94314 |
| H | 3.232111 | -3.16132 | 0.809654 |
| C | 5.017446 | -1.15857 | 0.254887 |
| H | 5.031151 | -2.14541 | 0.702337 |
| H | 5.980353 | -0.82877 | -0.12258 |
| C | 1.475071 | -1.35527 | -1.50952 |
| C | -0.2665 | -1.84615 | 0.278328 |
| C | -1.52344 | -2.36213 | 0.232311 |
| C | -1.87567 | -2.52586 | -1.18458 |
| O | -2.86327 | -2.96971 | -1.70852 |
| H | 1.756424 | -1.83525 | -2.44734 |
| C | -2.43895 | -2.77941 | 1.334418 |
| H | -3.47989 | -2.56585 | 1.071416 |
| H | -2.36815 | -3.85562 | 1.529821 |
| H | -2.19992 | -2.24717 | 2.259638 |
| C | 0.174905 | -1.64699 | -1.08019 |
| O | -0.79695 | -2.06728 | -1.94793 |
| C | 3.096257 | -0.62059 | 3.110921 |
| H | 4.002967 | -0.37826 | 3.657185 |
| H | 2.234221 | -0.93096 | 3.694972 |
| C | 1.853326 | -0.8269 | 0.904105 |
| H | 1.466253 | 0.151114 | 0.577144 |
| C | 0.664321 | -1.61093 | 1.436213 |
| H | 0.15835 | -1.06374 | 2.235882 |
| H | 0.984519 | -2.57726 | 1.849244 |
| C | 1.233918 | 1.496544 | -1.58042 |
| C | 1.536408 | 0.428232 | -2.42825 |
| H | 0.765069 | 0.123161 | -3.13201 |
| H | 2.539403 | 0.370102 | -2.84184 |

***Endo-Re*-Product, 29**

| C | 0.42491 | 1.552026 | -0.99528 |
| --- | --- | --- | --- |
| C | 0.364653 | 2.942724 | -0.38237 |
| C | 1.832429 | 3.396992 | -0.476 |
| C | 2.628054 | 2.310474 | -1.16646 |
| H | 2.255956 | 3.944023 | 0.360817 |
| H | 3.624759 | 2.028648 | -0.83847 |
| C | -0.59444 | 3.871587 | -1.15399 |
| H | -1.6233 | 3.496912 | -1.10891 |
| H | -0.58165 | 4.878343 | -0.72287 |
| H | -0.30787 | 3.946843 | -2.20442 |
| C | 2.419581 | 3.64159 | -1.83943 |
| H | 1.769118 | 3.670079 | -2.70785 |
| H | 3.267716 | 4.317127 | -1.88941 |
| C | -0.20975 | 2.859794 | 1.021882 |
| C | -0.74984 | 0.616443 | -1.03838 |
| H | -1.32155 | 0.782116 | -1.96245 |
| C | -1.67303 | 0.936228 | 0.111996 |
| O | 0.047611 | 1.676145 | 1.791121 |
| C | -2.96718 | 0.658784 | 0.310748 |
| C | -3.37293 | 1.300218 | 1.594906 |
| O | -4.44129 | 1.294052 | 2.131545 |
| H | -0.27418 | 3.770582 | 1.61696 |
| C | -3.9519 | -0.10146 | -0.5083 |
| H | -3.58243 | -0.26822 | -1.52377 |
| H | -4.16043 | -1.07684 | -0.05517 |
| H | -4.90015 | 0.442031 | -0.55072 |
| C | -1.22298 | 1.820147 | 1.228636 |
| O | -2.28697 | 1.97317 | 2.11112 |
| C | 2.294824 | -1.13439 | 2.187478 |
| C | 1.935836 | -1.66732 | -0.14542 |
| C | 3.382617 | -1.13913 | 0.033012 |
| C | 3.591112 | -0.81021 | 1.497896 |
| H | 3.811732 | -0.49048 | -0.72035 |
| H | 4.136969 | 0.075838 | 1.808436 |
| C | 1.899779 | -3.2064 | -0.10225 |
| H | 0.906687 | -3.58889 | -0.3555 |
| H | 2.61384 | -3.62217 | -0.82087 |
| H | 2.152126 | -3.58818 | 0.892172 |
| C | 4.355226 | -1.93525 | 0.858401 |
| H | 4.120029 | -2.95456 | 1.141678 |
| H | 5.40944 | -1.75902 | 0.66849 |
| C | 1.1783 | -1.20868 | -1.44085 |
| C | -0.82154 | -1.76068 | 0.025438 |
| C | -1.75154 | -2.62373 | -0.39336 |
| C | -1.95714 | -2.40846 | -1.84501 |
| O | -2.7157 | -2.96422 | -2.59013 |
| H | 1.149959 | -2.06717 | -2.12064 |
| C | -2.50419 | -3.68676 | 0.333079 |
| H | -3.58045 | -3.48604 | 0.300329 |
| H | -2.3509 | -4.65816 | -0.14709 |
| H | -2.19517 | -3.75189 | 1.378686 |
| C | -0.34224 | -0.92281 | -1.15034 |
| O | -1.10661 | -1.42008 | -2.25995 |
| C | 2.112903 | -1.40819 | 3.475503 |
| H | 2.943337 | -1.42121 | 4.174979 |
| H | 1.124142 | -1.60987 | 3.878943 |
| C | 1.247462 | -1.06815 | 1.109935 |
| H | 1.142551 | 0.001639 | 0.898827 |
| C | -0.14457 | -1.63321 | 1.358713 |
| H | -0.70906 | -0.9656 | 2.024524 |
| H | -0.10183 | -2.61466 | 1.847817 |
| C | 1.630251 | 1.242685 | -1.47468 |
| C | 1.817149 | -0.04633 | -2.21915 |
| H | 1.308939 | 0.024914 | -3.19044 |
| H | 2.864543 | -0.25995 | -2.4416 |

**C9'*Si*-TS-*exo*-*Re***

| C | -1.20092 | -1.28338 | 1.002379 |
| --- | --- | --- | --- |
| C | -2.36715 | -2.13553 | 0.497548 |
| C | -1.75679 | -3.54069 | 0.378148 |
| C | -0.34013 | -3.49605 | 0.893858 |
| H | -2.0073 | -4.13352 | -0.49591 |
| H | 0.467571 | -4.02552 | 0.397974 |
| C | -3.55223 | -2.08453 | 1.485878 |
| H | -3.9512 | -1.06727 | 1.561231 |
| H | -4.35621 | -2.7477 | 1.14887 |
| H | -3.24654 | -2.40074 | 2.484545 |
| C | -1.39331 | -4.26466 | 1.645102 |
| H | -1.726 | -3.87439 | 2.601683 |
| H | -1.34675 | -5.34765 | 1.593919 |
| C | -2.92744 | -1.57708 | -0.79609 |
| C | -1.14201 | 0.106768 | 0.864863 |
| H | -0.55445 | 0.725456 | 1.539882 |
| C | -2.25602 | 0.709696 | 0.139511 |
| O | -2.04868 | -0.92542 | -1.72409 |
| C | -2.93437 | 1.870008 | 0.185207 |
| C | -3.95506 | 1.834991 | -0.89674 |
| O | -4.76077 | 2.669905 | -1.18983 |
| H | -3.75123 | -2.11216 | -1.2676 |
| C | -2.83881 | 3.054333 | 1.085388 |
| H | -2.10016 | 2.898551 | 1.875568 |
| H | -2.5658 | 3.950395 | 0.516325 |
| H | -3.81265 | 3.255175 | 1.542762 |
| C | -2.87471 | -0.12312 | -0.93413 |
| O | -3.86648 | 0.625829 | -1.5543 |
| C | 2.496249 | 3.127002 | 1.119386 |
| C | 1.321994 | 1.707474 | -0.4396 |
| C | 0.405145 | 2.886227 | -0.06269 |
| C | 1.144689 | 3.763712 | 0.929476 |
| H | -0.65098 | 2.689318 | 0.040977 |
| H | 0.649652 | 4.206855 | 1.78914 |
| C | 2.067678 | 1.97822 | -1.76594 |
| H | 2.633178 | 1.10009 | -2.09116 |
| H | 1.350411 | 2.230422 | -2.55267 |
| H | 2.772557 | 2.81002 | -1.66313 |
| C | 0.807001 | 4.283248 | -0.44029 |
| H | 1.609329 | 4.450134 | -1.14955 |
| H | 0.006227 | 5.014589 | -0.49488 |
| C | 0.672294 | 0.336836 | -0.58371 |
| C | 2.997555 | -0.51429 | -0.10092 |
| C | 3.652834 | -1.57034 | -0.61409 |
| C | 2.640478 | -2.49906 | -1.16171 |
| O | 2.784871 | -3.58801 | -1.64934 |
| H | -0.05194 | 0.179214 | -1.37708 |
| C | 5.109339 | -1.8822 | -0.68416 |
| H | 5.430511 | -1.99694 | -1.72427 |
| H | 5.706831 | -1.09547 | -0.21691 |
| H | 5.322179 | -2.83066 | -0.18037 |
| C | 1.548703 | -0.72598 | -0.31463 |
| O | 1.400397 | -1.91651 | -1.02089 |
| C | 3.621002 | 3.718178 | 1.510039 |
| H | 3.653235 | 4.778439 | 1.741782 |
| H | 4.547487 | 3.160348 | 1.619282 |
| C | 2.307993 | 1.674708 | 0.762124 |
| H | 1.72208 | 1.259662 | 1.596738 |
| C | 3.501472 | 0.758437 | 0.52627 |
| H | 4.027704 | 0.547383 | 1.465734 |
| H | 4.230402 | 1.233644 | -0.14295 |
| C | -0.10026 | -2.08554 | 1.328791 |
| C | 1.13355 | -1.52978 | 1.616593 |
| H | 1.186575 | -0.59087 | 2.15857 |
| H | 1.998 | -2.17885 | 1.733862 |

**C9'*Si*-*exo*-*Re*-29**

| C | -1.32959 | -0.72199 | 1.111534 |
| --- | --- | --- | --- |
| C | -2.76639 | -0.76824 | 1.61901 |
| C | -2.87048 | -2.18489 | 2.193875 |
| C | -1.57197 | -2.90458 | 1.911227 |
| H | -3.81973 | -2.70472 | 2.107385 |
| H | -1.5382 | -3.94301 | 1.597155 |
| C | -3.10441 | 0.327792 | 2.637929 |
| H | -2.97283 | 1.324533 | 2.200712 |
| H | -4.14627 | 0.242034 | 2.964151 |
| H | -2.46532 | 0.250185 | 3.521393 |
| C | -1.96238 | -2.56213 | 3.327803 |
| H | -1.39002 | -1.78601 | 3.828207 |
| H | -2.29356 | -3.37164 | 3.970316 |
| C | -3.67796 | -0.5689 | 0.414347 |
| C | -0.79582 | 0.498064 | 0.415981 |
| H | -0.70225 | 1.282833 | 1.175826 |
| C | -1.86023 | 0.995262 | -0.53937 |
| O | -3.20521 | -1.10045 | -0.82729 |
| C | -1.92726 | 2.097198 | -1.29412 |
| C | -3.27903 | 2.131781 | -1.92162 |
| O | -3.74611 | 2.964326 | -2.64335 |
| H | -4.75671 | -0.63878 | 0.558005 |
| C | -0.94701 | 3.193236 | -1.54211 |
| H | -1.46634 | 4.154742 | -1.58504 |
| H | -0.18029 | 3.232316 | -0.76268 |
| H | -0.4497 | 3.053057 | -2.50873 |
| C | -3.16474 | 0.283272 | -0.66115 |
| O | -3.98745 | 1.032835 | -1.497 |
| C | 3.557358 | 1.300271 | 1.892915 |
| C | 1.804821 | 1.191975 | 0.221231 |
| C | 1.513556 | 2.369705 | 1.185971 |
| C | 2.610434 | 2.417866 | 2.2303 |
| H | 0.496492 | 2.594853 | 1.481671 |
| H | 2.396105 | 2.624296 | 3.274507 |
| C | 2.552402 | 1.696942 | -1.0309 |
| H | 2.63969 | 0.915534 | -1.78983 |
| H | 2.014122 | 2.538743 | -1.47778 |
| H | 3.566535 | 2.030853 | -0.78984 |
| C | 2.467605 | 3.533164 | 1.23375 |
| H | 3.227134 | 3.653674 | 0.470476 |
| H | 2.065169 | 4.468376 | 1.610301 |
| C | 0.591046 | 0.322341 | -0.28841 |
| C | 2.500273 | -1.3122 | -0.73523 |
| C | 2.669516 | -1.9079 | -1.9176 |
| C | 1.320882 | -2.15665 | -2.49539 |
| O | 1.045584 | -2.66138 | -3.54765 |
| H | 0.426109 | 0.633281 | -1.3243 |
| C | 3.900703 | -2.28217 | -2.67048 |
| H | 3.855648 | -1.88248 | -3.68795 |
| H | 4.801088 | -1.90779 | -2.17727 |
| H | 3.979501 | -3.37038 | -2.76075 |
| C | 1.020997 | -1.17949 | -0.44803 |
| O | 0.378667 | -1.69262 | -1.61999 |
| C | 4.840492 | 1.205646 | 2.22794 |
| H | 5.332108 | 1.979021 | 2.810209 |
| H | 5.441821 | 0.347343 | 1.940125 |
| C | 2.752763 | 0.311847 | 1.086589 |
| H | 2.096979 | -0.18294 | 1.820091 |
| C | 3.475896 | -0.75239 | 0.261395 |
| H | 3.865709 | -1.54622 | 0.912278 |
| H | 4.339529 | -0.32023 | -0.25891 |
| C | -0.71338 | -1.89551 | 1.226037 |
| C | 0.660485 | -2.14213 | 0.705505 |
| H | 1.399106 | -2.08137 | 1.514632 |
| H | 0.743024 | -3.15493 | 0.294194 |

**C9'*Si*-TS-*endo*-*Si***

| C | 0.42491 | 1.552026 | -0.99528 |
| --- | --- | --- | --- |
| C | 0.364653 | 2.942724 | -0.38237 |
| C | 1.832429 | 3.396992 | -0.476 |
| C | 2.628054 | 2.310474 | -1.16646 |
| H | 2.255956 | 3.944023 | 0.360817 |
| H | 3.624759 | 2.028648 | -0.83847 |
| C | -0.59444 | 3.871587 | -1.15399 |
| H | -1.6233 | 3.496912 | -1.10891 |
| H | -0.58165 | 4.878343 | -0.72287 |
| H | -0.30787 | 3.946843 | -2.20442 |
| C | 2.419581 | 3.64159 | -1.83943 |
| H | 1.769118 | 3.670079 | -2.70785 |
| H | 3.267716 | 4.317127 | -1.88941 |
| C | -0.20975 | 2.859794 | 1.021882 |
| C | -0.74984 | 0.616443 | -1.03838 |
| H | -1.32155 | 0.782116 | -1.96245 |
| C | -1.67303 | 0.936228 | 0.111996 |
| O | 0.047611 | 1.676145 | 1.791121 |
| C | -2.96718 | 0.658784 | 0.310748 |
| C | -3.37293 | 1.300218 | 1.594906 |
| O | -4.44129 | 1.294052 | 2.131545 |
| H | -0.27418 | 3.770582 | 1.61696 |
| C | -3.9519 | -0.10146 | -0.5083 |
| H | -3.58243 | -0.26822 | -1.52377 |
| H | -4.16043 | -1.07684 | -0.05517 |
| H | -4.90015 | 0.442031 | -0.55072 |
| C | -1.22298 | 1.820147 | 1.228636 |
| O | -2.28697 | 1.97317 | 2.11112 |
| C | 2.294824 | -1.13439 | 2.187478 |
| C | 1.935836 | -1.66732 | -0.14542 |
| C | 3.382617 | -1.13913 | 0.033012 |
| C | 3.591112 | -0.81021 | 1.497896 |
| H | 3.811732 | -0.49048 | -0.72035 |
| H | 4.136969 | 0.075838 | 1.808436 |
| C | 1.899779 | -3.2064 | -0.10225 |
| H | 0.906687 | -3.58889 | -0.3555 |
| H | 2.61384 | -3.62217 | -0.82087 |
| H | 2.152126 | -3.58818 | 0.892172 |
| C | 4.355226 | -1.93525 | 0.858401 |
| H | 4.120029 | -2.95456 | 1.141678 |
| H | 5.40944 | -1.75902 | 0.66849 |
| C | 1.1783 | -1.20868 | -1.44085 |
| C | -0.82154 | -1.76068 | 0.025438 |
| C | -1.75154 | -2.62373 | -0.39336 |
| C | -1.95714 | -2.40846 | -1.84501 |
| O | -2.7157 | -2.96422 | -2.59013 |
| H | 1.149959 | -2.06717 | -2.12064 |
| C | -2.50419 | -3.68676 | 0.333079 |
| H | -3.58045 | -3.48604 | 0.300329 |
| H | -2.3509 | -4.65816 | -0.14709 |
| H | -2.19517 | -3.75189 | 1.378686 |
| C | -0.34224 | -0.92281 | -1.15034 |
| O | -1.10661 | -1.42008 | -2.25995 |
| C | 2.112903 | -1.40819 | 3.475503 |
| H | 2.943337 | -1.42121 | 4.174979 |
| H | 1.124142 | -1.60987 | 3.878943 |
| C | 1.247462 | -1.06815 | 1.109935 |
| H | 1.142551 | 0.001639 | 0.898827 |
| C | -0.14457 | -1.63321 | 1.358713 |
| H | -0.70906 | -0.9656 | 2.024524 |
| H | -0.10183 | -2.61466 | 1.847817 |
| C | 1.630251 | 1.242685 | -1.47468 |
| C | 1.817149 | -0.04633 | -2.21915 |
| H | 1.308939 | 0.024914 | -3.19044 |
| H | 2.864543 | -0.25995 | -2.4416 |

**C9'*Si*-*endo*-*Si*-29**

| C | 0.918146 | 1.204636 | -0.64298 |
| --- | --- | --- | --- |
| C | 2.221376 | 1.886924 | -0.26885 |
| C | 1.94258 | 3.347877 | -0.68136 |
| C | 0.508871 | 3.450825 | -1.15029 |
| H | 2.732182 | 3.908235 | -1.17258 |
| H | 0.22432 | 4.063595 | -2.00058 |
| C | 2.568037 | 1.806201 | 1.236754 |
| H | 2.885452 | 0.797946 | 1.522268 |
| H | 3.392551 | 2.491368 | 1.460843 |
| H | 1.71241 | 2.081002 | 1.85626 |
| C | 0.933985 | 4.118466 | 0.130077 |
| H | 0.565875 | 3.700939 | 1.062274 |
| H | 1.026506 | 5.200038 | 0.126974 |
| C | 3.368801 | 1.152905 | -0.94668 |
| C | 0.718366 | -0.28026 | -0.80141 |
| H | 0.64777 | -0.41683 | -1.89607 |
| C | 1.992315 | -1.00803 | -0.50976 |
| O | 3.134971 | 0.448252 | -2.17395 |
| C | 2.363351 | -2.19989 | -0.02949 |
| C | 3.840227 | -2.31328 | -0.22555 |
| O | 4.566638 | -3.21453 | 0.076192 |
| H | 4.376058 | 1.559053 | -0.85337 |
| C | 1.597192 | -3.31339 | 0.607082 |
| H | 1.215177 | -3.0094 | 1.588176 |
| H | 0.746894 | -3.62114 | -0.00806 |
| H | 2.258439 | -4.17194 | 0.745307 |
| C | 3.221663 | -0.30674 | -1.00012 |
| O | 4.300447 | -1.16686 | -0.83619 |
| C | -1.71524 | 0.703051 | 3.047534 |
| C | -1.00969 | -0.70653 | 1.218799 |
| C | 0.175857 | -0.52901 | 2.19111 |
| C | -0.27867 | 0.357391 | 3.33336 |
| H | 1.170157 | -0.4185 | 1.778022 |
| H | 0.367634 | 1.121996 | 3.755482 |
| C | -1.81303 | -1.98479 | 1.529607 |
| H | -2.57275 | -2.16974 | 0.764383 |
| H | -1.14868 | -2.85398 | 1.558251 |
| H | -2.32721 | -1.91504 | 2.493236 |
| C | 0.070572 | -1.08399 | 3.584566 |
| H | -0.7223 | -1.77854 | 3.8374 |
| H | 1.006024 | -1.26751 | 4.104025 |
| C | -0.66548 | -0.72975 | -0.30482 |
| C | -3.09556 | -0.01408 | -0.48387 |
| C | -3.93273 | -0.62819 | -1.32284 |
| C | -3.16849 | -0.97869 | -2.549 |
| O | -3.55086 | -1.55061 | -3.53084 |
| H | -0.81066 | -1.75676 | -0.65875 |
| C | -5.37113 | -0.99801 | -1.19132 |
| H | -5.74492 | -0.79723 | -0.18451 |
| H | -5.97866 | -0.4352 | -1.90719 |
| H | -5.51086 | -2.05833 | -1.42129 |
| C | -1.72753 | 0.096739 | -1.12515 |
| O | -1.87798 | -0.54747 | -2.3954 |
| C | -2.64836 | 1.065745 | 3.922319 |
| H | -2.43068 | 1.14608 | 4.982908 |
| H | -3.66071 | 1.304208 | 3.606739 |
| C | -1.87436 | 0.545714 | 1.556711 |
| H | -1.3286 | 1.398752 | 1.126441 |
| C | -3.2639 | 0.48434 | 0.925597 |
| H | -3.73693 | 1.475007 | 0.935173 |
| H | -3.92599 | -0.18623 | 1.486765 |
| C | -0.01942 | 2.054453 | -1.05623 |
| C | -1.40498 | 1.607664 | -1.40531 |
| H | -1.61016 | 1.764393 | -2.47083 |
| H | -2.12072 | 2.243606 | -0.86702 |

**C9'*Si*-TS-*exo*-*Si***

| C | 1.161131 | 1.27098 | -0.51688 |
| --- | --- | --- | --- |
| C | 2.488434 | 1.832751 | -0.00412 |
| C | 2.258534 | 3.353859 | -0.05261 |
| C | 0.809203 | 3.618733 | -0.41789 |
| H | 3.044282 | 3.988315 | -0.45059 |
| H | 0.521639 | 4.446777 | -1.05871 |
| C | 2.819084 | 1.353857 | 1.426856 |
| H | 2.901704 | 0.263247 | 1.466904 |
| H | 3.778389 | 1.780713 | 1.740847 |
| H | 2.06147 | 1.647048 | 2.150837 |
| C | 1.326909 | 3.933244 | 0.968898 |
| H | 0.973972 | 3.31425 | 1.786886 |
| H | 1.449146 | 4.984305 | 1.2106 |
| C | 3.631354 | 1.265914 | -0.83475 |
| C | 0.987515 | -0.05372 | -0.817 |
| H | 0.037972 | -0.44684 | -1.16336 |
| C | 2.177841 | -0.8584 | -0.91758 |
| O | 3.394145 | 0.887152 | -2.19395 |
| C | 2.463319 | -2.17166 | -0.8616 |
| C | 3.92075 | -2.31924 | -1.11484 |
| O | 4.582467 | -3.31678 | -1.12722 |
| H | 4.639874 | 1.625756 | -0.6324 |
| C | 1.602129 | -3.34874 | -0.56449 |
| H | 0.590905 | -3.02993 | -0.29512 |
| H | 1.550534 | -4.02189 | -1.42661 |
| H | 2.021863 | -3.92632 | 0.266032 |
| C | 3.447237 | -0.12933 | -1.2328 |
| O | 4.467425 | -1.06653 | -1.32396 |
| C | -4.03579 | -1.28296 | -1.44937 |
| C | -3.30161 | 0.490081 | 0.009523 |
| C | -4.03735 | 1.119001 | -1.19657 |
| C | -4.48295 | -0.00681 | -2.11057 |
| H | -3.66482 | 2.045586 | -1.61668 |
| H | -4.41515 | 0.07265 | -3.19137 |
| C | -4.24519 | 0.330687 | 1.217957 |
| H | -3.70134 | -0.01089 | 2.103024 |
| H | -4.71609 | 1.289549 | 1.456923 |
| H | -5.03822 | -0.39523 | 1.013289 |
| C | -5.50015 | 0.83192 | -1.38923 |
| H | -6.07902 | 0.37841 | -0.59345 |
| H | -6.05428 | 1.544607 | -1.99177 |
| C | -2.03617 | 1.212728 | 0.513543 |
| C | -1.32717 | -1.11563 | 1.314656 |
| C | -0.4707 | -1.53815 | 2.288161 |
| C | 0.224341 | -0.356 | 2.792198 |
| O | 1.081744 | -0.22649 | 3.625941 |
| H | -2.2215 | 2.14086 | 1.057087 |
| C | -0.17477 | -2.90566 | 2.801196 |
| H | -0.41585 | -2.99027 | 3.866236 |
| H | -0.74521 | -3.66307 | 2.256582 |
| H | 0.892196 | -3.13395 | 2.698388 |
| C | -1.21241 | 0.311148 | 1.219713 |
| O | -0.28318 | 0.760958 | 2.100052 |
| C | -4.55867 | -2.4957 | -1.59934 |
| H | -5.40177 | -2.67161 | -2.26047 |
| H | -4.15504 | -3.35743 | -1.07418 |
| C | -2.87953 | -0.89571 | -0.56327 |
| H | -2.05161 | -0.68064 | -1.25898 |
| C | -2.34786 | -1.85625 | 0.491915 |
| H | -1.90688 | -2.74637 | 0.026335 |
| H | -3.16203 | -2.21101 | 1.138191 |
| C | 0.191615 | 2.291146 | -0.64974 |
| C | -1.15743 | 2.012098 | -0.9349 |
| H | -1.34253 | 1.209598 | -1.64924 |
| H | -1.78327 | 2.882074 | -1.12465 |

**C9'*Si*-*exo*-*Si*-29**

| C | 1.236856 | 1.507656 | -0.10127 |
| --- | --- | --- | --- |
| C | 2.688081 | 1.816239 | 0.167754 |
| C | 2.673271 | 3.357886 | 0.215806 |
| C | 1.240111 | 3.831819 | 0.075498 |
| H | 3.484393 | 3.89918 | -0.26157 |
| H | 0.980047 | 4.703369 | -0.51793 |
| C | 3.196241 | 1.19173 | 1.483984 |
| H | 3.126115 | 0.099507 | 1.455405 |
| H | 4.24361 | 1.466405 | 1.650876 |
| H | 2.604511 | 1.529432 | 2.335135 |
| C | 1.975902 | 4.016776 | 1.375972 |
| H | 1.660999 | 3.41202 | 2.220192 |
| H | 2.298497 | 5.020941 | 1.633238 |
| C | 3.489857 | 1.152645 | -0.9335 |
| C | 0.661053 | 0.15378 | -0.41616 |
| H | 0.143527 | 0.258997 | -1.38438 |
| C | 1.78199 | -0.82443 | -0.71198 |
| O | 2.834899 | 0.910546 | -2.19125 |
| C | 2.005995 | -2.14954 | -0.72497 |
| C | 3.348217 | -2.37136 | -1.35763 |
| O | 3.909415 | -3.41088 | -1.55083 |
| H | 4.559846 | 1.341462 | -1.01664 |
| C | 1.280926 | -3.38578 | -0.29432 |
| H | 0.22426 | -3.37118 | -0.56344 |
| H | 1.7622 | -4.24315 | -0.7702 |
| H | 1.354914 | -3.52114 | 0.789354 |
| C | 2.99824 | -0.17133 | -1.32396 |
| O | 3.88281 | -1.16703 | -1.71737 |
| C | -4.00898 | -0.41738 | -1.71251 |
| C | -2.90216 | 0.60881 | 0.191768 |
| C | -3.82012 | 1.656634 | -0.49092 |
| C | -4.49125 | 1.005578 | -1.68443 |
| H | -3.49605 | 2.687855 | -0.5532 |
| H | -4.62984 | 1.530319 | -2.6248 |
| C | -3.61554 | -0.00867 | 1.409057 |
| H | -2.93171 | -0.57111 | 2.046971 |
| H | -4.05931 | 0.787179 | 2.015351 |
| H | -4.41771 | -0.68932 | 1.104775 |
| C | -5.30531 | 1.427009 | -0.4933 |
| H | -5.74089 | 0.658425 | 0.134716 |
| H | -5.929 | 2.306634 | -0.61806 |
| C | -1.49378 | 1.121416 | 0.627032 |
| C | -1.11668 | -1.43069 | 0.604512 |
| C | -0.78967 | -2.13345 | 1.69221 |
| C | 0.116399 | -1.29992 | 2.518392 |
| O | 0.666105 | -1.57973 | 3.547774 |
| H | -1.5461 | 1.441473 | 1.673342 |
| C | -1.20286 | -3.49223 | 2.144488 |
| H | -1.8405 | -3.42325 | 3.032381 |
| H | -1.74476 | -4.02977 | 1.362811 |
| H | -0.32438 | -4.0784 | 2.43212 |
| C | -0.44079 | -0.07798 | 0.656159 |
| O | 0.244641 | -0.08148 | 1.916319 |
| C | -4.60888 | -1.46109 | -2.27677 |
| H | -5.55621 | -1.35636 | -2.79673 |
| H | -4.16963 | -2.45486 | -2.24747 |
| C | -2.70426 | -0.41971 | -0.95859 |
| H | -1.9834 | 0.055326 | -1.64464 |
| C | -2.10721 | -1.73983 | -0.4796 |
| H | -1.63061 | -2.26795 | -1.31731 |
| H | -2.88725 | -2.40271 | -0.08622 |
| C | 0.446724 | 2.574138 | -0.07626 |
| C | -1.02855 | 2.358603 | -0.19591 |
| H | -1.30036 | 2.218571 | -1.25447 |
| H | -1.5811 | 3.236684 | 0.149827 |

**C9'*Re*-TS-*endo*-*Si*1**

| C | -0.18285 | -4.13322 | -0.09889 |
| --- | --- | --- | --- |
| C | 0.032246 | -3.35282 | 1.184249 |
| C | 1.200231 | -3.71994 | 0.283095 |
| C | 0.013594 | -1.86566 | 1.270888 |
| C | 1.750147 | -2.41581 | -0.32614 |
| C | 1.087627 | -1.32358 | 0.520017 |
| C | 2.81613 | 0.247549 | -0.05324 |
| C | 3.261549 | -2.27675 | -0.29418 |
| C | 3.756451 | -0.90648 | -0.18693 |
| C | 3.46976 | 1.354939 | -0.45686 |
| C | 4.815514 | 0.925573 | -0.92306 |
| O | 4.929356 | -0.44496 | -0.76394 |
| O | 5.696089 | 1.586899 | -1.39098 |
| C | 1.524601 | -0.02412 | 0.52122 |
| C | -0.81436 | -1.11 | 2.135126 |
| H | -0.66815 | -3.66761 | -0.94711 |
| H | -0.44078 | -5.17853 | 0.038996 |
| H | -0.14828 | -3.89554 | 2.107768 |
| H | 1.910928 | -4.4793 | 0.594943 |
| C | 1.380358 | -2.24732 | -1.81605 |
| H | 3.852976 | -3.01387 | -0.83755 |
| H | -0.30436 | -0.33782 | 2.713177 |
| H | -1.4705 | -1.71716 | 2.758205 |
| O | 3.868137 | -1.78404 | 0.901155 |
| H | 0.300246 | -2.20792 | -1.968 |
| H | 1.80237 | -1.31618 | -2.20754 |
| H | 1.778109 | -3.08365 | -2.40114 |
| H | 1.023554 | 0.746115 | 1.09469 |
| C | -4.75383 | -2.11114 | -0.36988 |
| C | -5.28161 | -0.70461 | -0.29544 |
| C | -4.1529 | -1.15115 | 0.615237 |
| C | -4.69524 | 0.334475 | -1.21565 |
| C | -2.88958 | -0.36539 | 0.209204 |
| C | -3.51049 | 0.896157 | -0.47251 |
| C | -1.44536 | 2.103667 | -0.1024 |
| C | -2.03031 | 0.107174 | 1.391452 |
| C | -1.35309 | 1.307928 | 1.08227 |
| C | -0.70909 | 3.236855 | 0.102849 |
| C | -0.13438 | 3.145222 | 1.444425 |
| O | -0.59323 | 1.942024 | 2.014915 |
| O | 0.58511 | 3.887273 | 2.059061 |
| C | -2.45573 | 1.756735 | -1.16555 |
| C | -5.12173 | 0.676368 | -2.42732 |
| H | -4.18622 | -2.41203 | -1.24363 |
| H | -5.39615 | -2.88764 | 0.033261 |
| H | -6.2886 | -0.55428 | 0.081533 |
| H | -4.3301 | -1.32227 | 1.673559 |
| C | -2.03711 | -1.11617 | -0.82008 |
| H | -2.8944 | 2.676358 | -1.56959 |
| H | -5.98946 | 0.205361 | -2.8791 |
| H | -4.62188 | 1.449518 | -3.00501 |
| H | -1.89087 | -2.14787 | -0.50016 |
| H | -2.52402 | -1.12301 | -1.80173 |
| H | -1.05894 | -0.63602 | -0.92878 |
| H | -3.93455 | 1.495333 | 0.349177 |
| H | -2.00237 | 1.220736 | -2.00788 |
| H | -2.56164 | 0.149854 | 2.345525 |
| C | -0.48565 | 4.428778 | -0.7614 |
| H | 0.481152 | 4.366072 | -1.27765 |
| H | -1.26426 | 4.520115 | -1.52371 |
| H | -0.46822 | 5.340281 | -0.15755 |
| C | 3.043399 | 2.779311 | -0.53875 |
| H | 3.907715 | 3.433828 | -0.40041 |
| H | 2.623098 | 3.001416 | -1.52765 |
| H | 2.290021 | 3.018674 | 0.217561 |

**C9'*Re*-Int-*endo***

| C | -0.16801 | -4.22511 | 0.077865 |
| --- | --- | --- | --- |
| C | 0.055996 | -3.37727 | 1.316239 |
| C | 1.211708 | -3.75307 | 0.407432 |
| C | -0.02446 | -1.89134 | 1.301997 |
| C | 1.712282 | -2.45726 | -0.26184 |
| C | 1.013863 | -1.36157 | 0.554573 |
| C | 2.670385 | 0.261578 | -0.11921 |
| C | 3.218467 | -2.25531 | -0.25225 |
| C | 3.652603 | -0.86104 | -0.22712 |
| C | 3.274617 | 1.378196 | -0.5791 |
| C | 4.625237 | 0.98127 | -1.05756 |
| O | 4.794385 | -0.3771 | -0.84706 |
| O | 5.47086 | 1.655397 | -1.56997 |
| C | 1.417192 | -0.02818 | 0.514877 |
| C | -0.99063 | -1.10843 | 2.095325 |
| H | -0.69095 | -3.81188 | -0.77513 |
| H | -0.38894 | -5.27092 | 0.267314 |
| H | -0.09599 | -3.87009 | 2.273158 |
| H | 1.949034 | -4.48001 | 0.734352 |
| C | 1.338389 | -2.37079 | -1.75766 |
| H | 3.835341 | -2.99359 | -0.76508 |
| H | -0.47679 | -0.52036 | 2.867171 |
| H | -1.68277 | -1.78581 | 2.604564 |
| O | 3.819265 | -1.67359 | 0.904884 |
| H | 0.261986 | -2.42789 | -1.91957 |
| H | 1.688044 | -1.424 | -2.18249 |
| H | 1.810324 | -3.19104 | -2.30945 |
| H | 1.001303 | 0.70559 | 1.186033 |
| C | -4.56859 | -2.13904 | -0.57693 |
| C | -5.1201 | -0.75267 | -0.38705 |
| C | -3.97014 | -1.24579 | 0.470731 |
| C | -4.56505 | 0.362678 | -1.23336 |
| C | -2.72759 | -0.40384 | 0.115137 |
| C | -3.37684 | 0.889293 | -0.46828 |
| C | -1.34729 | 2.12774 | -0.0373 |
| C | -1.89348 | 0.000342 | 1.3529 |
| C | -1.17188 | 1.244372 | 1.056791 |
| C | -0.68564 | 3.296286 | 0.263295 |
| C | -0.104 | 3.13776 | 1.587973 |
| O | -0.4765 | 1.847865 | 2.048168 |
| O | 0.565267 | 3.863242 | 2.276097 |
| C | -2.35052 | 1.816518 | -1.11771 |
| C | -5.01695 | 0.786303 | -2.40937 |
| H | -4.01062 | -2.36136 | -1.47985 |
| H | -5.18962 | -2.95657 | -0.2247 |
| H | -6.124 | -0.64997 | 0.013513 |
| H | -4.13047 | -1.5022 | 1.514686 |
| C | -1.86943 | -1.05937 | -0.97089 |
| H | -2.81663 | 2.74673 | -1.46251 |
| H | -5.88352 | 0.333322 | -2.88136 |
| H | -4.5402 | 1.608772 | -2.93602 |
| H | -1.69185 | -2.10771 | -0.72892 |
| H | -2.3752 | -1.01455 | -1.94211 |
| H | -0.90674 | -0.54967 | -1.06821 |
| H | -3.80042 | 1.426382 | 0.397028 |
| H | -1.89082 | 1.344104 | -1.99391 |
| H | -2.59907 | 0.241259 | 2.164327 |
| C | -0.53843 | 4.552526 | -0.52206 |
| H | 0.267078 | 4.469228 | -1.26435 |
| H | -1.4574 | 4.797387 | -1.06455 |
| H | -0.28801 | 5.382342 | 0.143567 |
| C | 2.77932 | 2.775441 | -0.70717 |
| H | 3.62517 | 3.46285 | -0.78216 |
| H | 2.172326 | 2.893447 | -1.61362 |
| H | 2.157035 | 3.058261 | 0.149538 |

**C9'*Re*-TS-*endo*-Si2**

| C | -0.16291 | -4.24058 | 0.083834 |
| --- | --- | --- | --- |
| C | 0.064325 | -3.39704 | 1.323709 |
| C | 1.216855 | -3.76227 | 0.406586 |
| C | -0.02295 | -1.91089 | 1.314542 |
| C | 1.707422 | -2.46094 | -0.26011 |
| C | 1.00725 | -1.37396 | 0.566314 |
| C | 2.644592 | 0.266573 | -0.11676 |
| C | 3.212228 | -2.24719 | -0.25599 |
| C | 3.634581 | -0.84935 | -0.23256 |
| C | 3.235217 | 1.385492 | -0.58585 |
| C | 4.58536 | 0.998542 | -1.07485 |
| O | 4.768135 | -0.35706 | -0.86102 |
| O | 5.420325 | 1.678497 | -1.59681 |
| C | 1.401132 | -0.03505 | 0.536897 |
| C | -0.996 | -1.12657 | 2.100363 |
| H | -0.6937 | -3.82697 | -0.76408 |
| H | -0.37596 | -5.28874 | 0.269357 |
| H | -0.07817 | -3.89365 | 2.280135 |
| H | 1.959765 | -4.48723 | 0.725218 |
| C | 1.330444 | -2.37343 | -1.75509 |
| H | 3.83313 | -2.97918 | -0.77298 |
| H | -0.48893 | -0.54904 | 2.88473 |
| H | -1.70178 | -1.80089 | 2.594968 |
| O | 3.814737 | -1.66093 | 0.897974 |
| H | 0.255804 | -2.45222 | -1.91771 |
| H | 1.661467 | -1.41805 | -2.17584 |
| H | 1.818619 | -3.18141 | -2.31091 |
| H | 1.020851 | 0.674609 | 1.253343 |
| C | -4.56332 | -2.12821 | -0.57988 |
| C | -5.10185 | -0.73597 | -0.39538 |
| C | -3.95745 | -1.23706 | 0.465216 |
| C | -4.53572 | 0.371879 | -1.24422 |
| C | -2.70659 | -0.40801 | 0.107948 |
| C | -3.34354 | 0.889364 | -0.47885 |
| C | -1.31257 | 2.118809 | -0.04043 |
| C | -1.87067 | -0.009 | 1.346155 |
| C | -1.12826 | 1.224466 | 1.046826 |
| C | -0.67879 | 3.295991 | 0.278628 |
| C | -0.10606 | 3.135246 | 1.608101 |
| O | -0.45989 | 1.837316 | 2.053423 |
| O | 0.544514 | 3.867247 | 2.306584 |
| C | -2.30801 | 1.807001 | -1.12776 |
| C | -4.98278 | 0.796818 | -2.42159 |
| H | -4.00639 | -2.35926 | -1.48124 |
| H | -5.19263 | -2.9384 | -0.22536 |
| H | -6.10505 | -0.6227 | 0.004052 |
| H | -4.12139 | -1.48807 | 1.509904 |
| C | -1.85465 | -1.07435 | -0.97598 |
| H | -2.76622 | 2.73792 | -1.48081 |
| H | -5.85314 | 0.35056 | -2.893 |
| H | -4.49829 | 1.613663 | -2.9499 |
| H | -1.68408 | -2.12264 | -0.72894 |
| H | -2.36143 | -1.03061 | -1.94671 |
| H | -0.88863 | -0.57211 | -1.0769 |
| H | -3.7637 | 1.431838 | 0.384862 |
| H | -1.84485 | 1.3269 | -1.99775 |
| H | -2.57846 | 0.254389 | 2.148903 |
| C | -0.55211 | 4.565399 | -0.48936 |
| H | 0.257704 | 4.507568 | -1.22917 |
| H | -1.47344 | 4.800882 | -1.03195 |
| H | -0.32004 | 5.390056 | 0.189196 |
| C | 2.727805 | 2.778343 | -0.71561 |
| H | 3.566666 | 3.46879 | -0.83005 |
| H | 2.087221 | 2.879172 | -1.60047 |
| H | 2.134541 | 3.069852 | 0.158654 |

**C9'*Re*-*endo*-*Si*-29**

| C | 0.372968 | -4.47474 | -0.01714 |
| --- | --- | --- | --- |
| C | 0.470104 | -3.68583 | 1.261187 |
| C | 1.655851 | -3.76412 | 0.324755 |
| C | 0.067722 | -2.24306 | 1.25442 |
| C | 1.890847 | -2.3578 | -0.26666 |
| C | 0.897517 | -1.51489 | 0.510243 |
| C | 2.067306 | 0.554664 | -0.22954 |
| C | 3.275636 | -1.75702 | -0.11939 |
| C | 3.313726 | -0.29232 | -0.14489 |
| C | 2.377657 | 1.631981 | -0.9696 |
| C | 3.83216 | 1.526316 | -1.31902 |
| O | 4.352236 | 0.38624 | -0.76526 |
| O | 4.490077 | 2.278422 | -1.97757 |
| C | 0.977173 | -0.01213 | 0.646589 |
| C | -1.07877 | -1.59935 | 1.977681 |
| H | -0.25737 | -4.09581 | -0.81386 |
| H | 0.379865 | -5.55719 | 0.066438 |
| H | 0.453417 | -4.2211 | 2.206495 |
| H | 2.52182 | -4.37039 | 0.572227 |
| C | 1.636994 | -2.31997 | -1.79135 |
| H | 4.128674 | -2.29517 | -0.53298 |
| H | -0.79016 | -1.34148 | 3.005949 |
| H | -1.92098 | -2.2982 | 2.055926 |
| O | 3.588215 | -0.97346 | 1.04259 |
| H | 0.667579 | -2.74762 | -2.04856 |
| H | 1.66692 | -1.2917 | -2.1693 |
| H | 2.409849 | -2.89404 | -2.3144 |
| H | 1.485345 | 0.069532 | 1.620688 |
| C | -4.54048 | -1.96816 | -0.68188 |
| C | -4.82951 | -0.49729 | -0.55783 |
| C | -3.80449 | -1.1576 | 0.345809 |
| C | -4.06613 | 0.486234 | -1.40853 |
| C | -2.42072 | -0.55549 | -0.00481 |
| C | -2.84639 | 0.81983 | -0.58525 |
| C | -0.83789 | 1.844523 | 0.114753 |
| C | -1.55208 | -0.31442 | 1.258889 |
| C | -0.38351 | 0.69223 | 0.998803 |
| C | -0.70348 | 2.996166 | 0.781705 |
| C | -0.21213 | 2.690011 | 2.145348 |
| O | -0.1004 | 1.337554 | 2.261122 |
| O | 0.023983 | 3.446747 | 3.045771 |
| C | -1.6517 | 1.60988 | -1.12541 |
| C | -4.39333 | 0.954687 | -2.60804 |
| H | -4.0208 | -2.33365 | -1.55987 |
| H | -5.31022 | -2.63806 | -0.31178 |
| H | -5.80394 | -0.19915 | -0.1825 |
| H | -4.03 | -1.33261 | 1.394245 |
| C | -1.72213 | -1.36476 | -1.10569 |
| H | -1.9643 | 2.565837 | -1.55859 |
| H | -5.30303 | 0.640648 | -3.11075 |
| H | -3.76577 | 1.675119 | -3.12635 |
| H | -1.63733 | -2.41734 | -0.82265 |
| H | -2.29074 | -1.30094 | -2.03939 |
| H | -0.7176 | -0.99327 | -1.31513 |
| H | -3.23863 | 1.393438 | 0.270774 |
| H | -1.11772 | 1.046589 | -1.89922 |
| H | -2.20117 | 0.229615 | 1.9582 |
| C | -1.02204 | 4.401805 | 0.397733 |
| H | -1.11936 | 4.516983 | -0.68458 |
| H | -1.95355 | 4.732847 | 0.869355 |
| H | -0.23155 | 5.067997 | 0.75662 |
| C | 1.666786 | 2.849703 | -1.4714 |
| H | 2.27857 | 3.298219 | -2.25784 |
| H | 0.679544 | 2.620856 | -1.87517 |
| H | 1.543605 | 3.591373 | -0.67619 |

**C9'*Re*-TS-*exo*-*Si***

| C | 1.194326 | -4.10298 | -0.2179 |
| --- | --- | --- | --- |
| C | 1.143836 | -3.44585 | 1.137215 |
| C | 2.37265 | -3.30278 | 0.260127 |
| C | 0.59801 | -2.06286 | 1.293812 |
| C | 2.475488 | -1.83349 | -0.19045 |
| C | 1.440915 | -1.13631 | 0.687324 |
| C | 2.398971 | 1.022728 | 0.222286 |
| C | 3.814562 | -1.14621 | -0.00479 |
| C | 3.719636 | 0.300895 | 0.170445 |
| C | 2.639037 | 2.300764 | -0.11062 |
| C | 4.086169 | 2.418407 | -0.44737 |
| O | 4.683926 | 1.192034 | -0.28333 |
| O | 4.684853 | 3.38567 | -0.82036 |
| C | 1.246957 | 0.242793 | 0.706614 |
| C | -0.64837 | -1.65684 | 1.766644 |
| H | 0.552855 | -3.73008 | -1.00685 |
| H | 1.310702 | -5.18217 | -0.21158 |
| H | 1.150862 | -4.08325 | 2.016134 |
| H | 3.289639 | -3.82764 | 0.509693 |
| C | 2.145301 | -1.65696 | -1.68975 |
| H | 4.695547 | -1.55937 | -0.49524 |
| H | -0.73156 | -0.71186 | 2.290405 |
| H | -1.36798 | -2.40914 | 2.083509 |
| O | 4.069568 | -0.51302 | 1.253719 |
| H | 1.127849 | -1.97701 | -1.91313 |
| H | 2.24388 | -0.60624 | -1.98576 |
| H | 2.839277 | -2.24901 | -2.29619 |
| H | 0.698148 | 0.669011 | 1.539185 |
| C | -1.69214 | 4.204674 | -0.18809 |
| C | -2.82028 | 3.502582 | -0.89145 |
| C | -1.46254 | 2.837765 | -0.76675 |
| C | -3.77685 | 2.654399 | -0.09284 |
| C | -1.62743 | 1.59349 | 0.13888 |
| C | -3.14576 | 1.287031 | -0.09508 |
| C | -2.86101 | -1.04603 | 0.236961 |
| C | -0.82656 | 0.371224 | -0.32238 |
| C | -1.41519 | -0.88301 | -0.04975 |
| C | -3.26932 | -2.21015 | -0.30033 |
| C | -2.10772 | -2.81613 | -0.9838 |
| O | -1.04837 | -1.94165 | -0.88269 |
| O | -2.01681 | -3.85691 | -1.57751 |
| C | -3.65055 | 0.120514 | 0.754521 |
| C | -4.91497 | 3.0289 | 0.482039 |
| H | -1.74098 | 4.339176 | 0.886883 |
| H | -1.23813 | 5.036831 | -0.71798 |
| H | -3.16367 | 3.908413 | -1.83836 |
| H | -0.80941 | 2.749741 | -1.63051 |
| C | -1.41174 | 1.939638 | 1.624751 |
| H | -4.72121 | -0.05196 | 0.604322 |
| H | -5.2736 | 4.051679 | 0.416907 |
| H | -5.53329 | 2.322369 | 1.029678 |
| H | -0.46352 | 2.46101 | 1.782098 |
| H | -2.21509 | 2.586729 | 1.990468 |
| H | -1.4135 | 1.050938 | 2.258764 |
| H | -3.20419 | 0.955109 | -1.14395 |
| H | -3.48876 | 0.304602 | 1.823361 |
| H | -0.26246 | 0.447443 | -1.24701 |
| C | 1.776821 | 3.510461 | -0.19403 |
| H | 2.271092 | 4.34793 | 0.30773 |
| H | 1.629095 | 3.810371 | -1.23809 |
| H | 0.798983 | 3.343865 | 0.256766 |
| C | -4.60775 | -2.86634 | -0.35039 |
| H | -5.36129 | -2.28584 | 0.186564 |
| H | -4.93515 | -2.99755 | -1.38657 |
| H | -4.55644 | -3.86506 | 0.095033 |

**C9'*Re*-*exo*-*Si*-29**

| C | 1.267466 | -4.22454 | -0.05173 |
| --- | --- | --- | --- |
| C | 1.259817 | -3.48647 | 1.26069 |
| C | 2.437867 | -3.35197 | 0.321665 |
| C | 0.638216 | -2.13009 | 1.261759 |
| C | 2.472771 | -1.9096 | -0.21599 |
| C | 1.342112 | -1.25894 | 0.54649 |
| C | 2.130225 | 1.02145 | 0.021597 |
| C | 3.717315 | -1.10626 | 0.061111 |
| C | 3.479188 | 0.330048 | 0.148269 |
| C | 2.414585 | 2.301195 | -0.28584 |
| C | 3.893725 | 2.418782 | -0.4906 |
| O | 4.476529 | 1.217042 | -0.23634 |
| O | 4.515817 | 3.386573 | -0.82445 |
| C | 0.919573 | 0.192328 | 0.476712 |
| C | -0.7597 | -1.71886 | 1.611074 |
| H | 0.566892 | -3.90271 | -0.81583 |
| H | 1.436863 | -5.29614 | -0.013 |
| H | 1.327724 | -4.04589 | 2.189242 |
| H | 3.382286 | -3.83786 | 0.547074 |
| C | 2.232025 | -1.83998 | -1.73761 |
| H | 4.689304 | -1.44691 | -0.29439 |
| H | -0.81687 | -1.0109 | 2.447363 |
| H | -1.38832 | -2.57862 | 1.861256 |
| O | 3.750298 | -0.40112 | 1.311366 |
| H | 1.276706 | -2.29619 | -2.00175 |
| H | 2.21947 | -0.79977 | -2.08438 |
| H | 3.031168 | -2.36465 | -2.2724 |
| H | 0.808783 | 0.480149 | 1.531911 |
| C | -1.45598 | 4.136073 | -0.22101 |
| C | -2.58497 | 3.409378 | -0.8856 |
| C | -1.23797 | 2.732343 | -0.70728 |
| C | -3.54214 | 2.636865 | -0.02528 |
| C | -1.40589 | 1.537344 | 0.272433 |
| C | -2.92821 | 1.265594 | 0.050429 |
| C | -2.82617 | -1.02061 | 0.35195 |
| C | -0.56239 | 0.270477 | -0.11559 |
| C | -1.31072 | -1.06985 | 0.299716 |
| C | -3.34135 | -2.00605 | -0.39152 |
| C | -2.21682 | -2.68216 | -1.07738 |
| O | -1.06033 | -2.05093 | -0.72805 |
| O | -2.23978 | -3.60655 | -1.8439 |
| C | -3.50439 | 0.168362 | 0.939094 |
| C | -4.6684 | 3.060817 | 0.537656 |
| H | -1.51584 | 4.335109 | 0.844188 |
| H | -0.98127 | 4.928218 | -0.79311 |
| H | -2.92098 | 3.751333 | -1.86001 |
| H | -0.58893 | 2.583881 | -1.5652 |
| C | -1.16097 | 1.947432 | 1.733209 |
| H | -4.59134 | 0.09373 | 0.836466 |
| H | -5.01746 | 4.08043 | 0.406266 |
| H | -5.2854 | 2.402193 | 1.143199 |
| H | -0.19474 | 2.448041 | 1.850695 |
| H | -1.93984 | 2.631924 | 2.082908 |
| H | -1.16675 | 1.08659 | 2.408373 |
| H | -2.99557 | 0.89153 | -0.98488 |
| H | -3.26862 | 0.334488 | 1.996445 |
| H | -0.50861 | 0.221943 | -1.20993 |
| C | 1.672374 | 3.590483 | -0.39478 |
| H | 2.385266 | 4.400101 | -0.2211 |
| H | 1.253432 | 3.740296 | -1.3941 |
| H | 0.863327 | 3.658143 | 0.327894 |
| C | -4.74948 | -2.41912 | -0.6576 |
| H | -5.45949 | -1.8562 | -0.04793 |
| H | -4.99792 | -2.27362 | -1.71373 |
| H | -4.87374 | -3.48566 | -0.44691 |

**C9'*Re*-TS-*endo*-*Re***

| C | 1.439243 | -3.29498 | 3.01113 |
| --- | --- | --- | --- |
| C | 0.396277 | -2.30156 | 2.578511 |
| C | 1.837987 | -1.87355 | 2.717386 |
| C | 0.071436 | -2.24194 | 1.112406 |
| C | 2.383397 | -1.5483 | 1.313096 |
| C | 1.137975 | -1.6377 | 0.43284 |
| C | 2.095491 | -0.1036 | -1.18635 |
| C | 3.096336 | -0.21425 | 1.180411 |
| C | 2.939367 | 0.473717 | -0.10208 |
| C | 2.532535 | 0.393248 | -2.35454 |
| C | 3.698844 | 1.268433 | -2.05476 |
| O | 3.914875 | 1.267597 | -0.68878 |
| O | 4.392573 | 1.881462 | -2.81191 |
| C | 1.00323 | -0.99309 | -0.80714 |
| C | -1.15981 | -2.46183 | 0.521379 |
| H | 1.71446 | -4.08589 | 2.321707 |
| H | 1.433029 | -3.59466 | 4.054381 |
| H | -0.36463 | -1.9986 | 3.292276 |
| H | 2.157436 | -1.25073 | 3.547679 |
| C | 3.425601 | -2.58022 | 0.822722 |
| H | 4.023943 | -0.0782 | 1.736049 |
| H | -1.20085 | -2.79738 | -0.50695 |
| H | -2.01379 | -2.73388 | 1.138089 |
| O | 2.312517 | 0.976291 | 1.044078 |
| H | 2.992653 | -3.57871 | 0.746322 |
| H | 3.798357 | -2.30234 | -0.16889 |
| H | 4.273386 | -2.6228 | 1.515055 |
| H | 0.438479 | -1.4648 | -1.608 |
| C | 0.311086 | 3.427111 | 1.474326 |
| C | -0.93066 | 3.758264 | 0.688612 |
| C | -0.0435 | 2.593409 | 0.284827 |
| C | -2.24432 | 3.159951 | 1.121376 |
| C | -0.8508 | 1.296647 | 0.493255 |
| C | -2.3235 | 1.832735 | 0.410418 |
| C | -3.118 | -0.36467 | -0.19087 |
| C | -0.71185 | 0.29478 | -0.64952 |
| C | -1.77683 | -0.61213 | -0.73996 |
| C | -4.00535 | -1.04272 | -0.94983 |
| C | -3.25771 | -1.70574 | -2.03412 |
| O | -1.92287 | -1.34121 | -1.91048 |
| O | -3.64407 | -2.40407 | -2.9298 |
| C | -3.34533 | 0.75719 | 0.789001 |
| C | -3.13934 | 3.68137 | 1.954717 |
| H | 0.21435 | 3.011661 | 2.470552 |
| H | 1.151518 | 4.102953 | 1.354491 |
| H | -0.9487 | 4.68478 | 0.122658 |
| H | 0.597654 | 2.654677 | -0.59072 |
| C | -0.65185 | 0.687049 | 1.889657 |
| H | -4.36986 | 1.129741 | 0.682347 |
| H | -2.98439 | 4.650033 | 2.420208 |
| H | -4.06385 | 3.161266 | 2.19175 |
| H | 0.402919 | 0.519741 | 2.103749 |
| H | -1.06168 | 1.363492 | 2.647217 |
| H | -1.1835 | -0.25958 | 1.978832 |
| H | -2.49127 | 2.079287 | -0.65044 |
| H | -3.21779 | 0.446151 | 1.831491 |
| H | -0.33251 | 0.689924 | -1.58954 |
| C | 2.076538 | 0.174776 | -3.75613 |
| H | 1.88337 | 1.13453 | -4.24484 |
| H | 2.855979 | -0.32699 | -4.3388 |
| H | 1.167523 | -0.43091 | -3.79042 |
| C | -5.49439 | -1.11036 | -0.90313 |
| H | -5.92754 | -0.75246 | -1.84253 |
| H | -5.82505 | -2.1464 | -0.77559 |
| H | -5.89651 | -0.51677 | -0.07895 |

**C9'*Re*-*endo*-*Re***

| C | -1.59647 | -4.30589 | -1.29854 |
| --- | --- | --- | --- |
| C | -0.90673 | -3.14064 | -1.95821 |
| C | -2.36745 | -3.04698 | -1.58521 |
| C | -0.21162 | -2.19371 | -1.03583 |
| C | -2.51066 | -2.01218 | -0.45963 |
| C | -1.07444 | -1.55579 | -0.24478 |
| C | -1.85728 | 0.519055 | 0.799405 |
| C | -3.36153 | -0.80644 | -0.81926 |
| C | -2.97255 | 0.462082 | -0.19356 |
| C | -2.20617 | 1.414082 | 1.731902 |
| C | -3.49246 | 2.034905 | 1.312557 |
| O | -3.9051 | 1.438952 | 0.144152 |
| O | -4.12273 | 2.892144 | 1.860854 |
| C | -0.7196 | -0.47307 | 0.740669 |
| C | 1.267972 | -2.01023 | -0.96081 |
| H | -1.3163 | -4.53894 | -0.27564 |
| H | -1.82816 | -5.1693 | -1.914 |
| H | -0.57356 | -3.22826 | -2.98828 |
| H | -3.13823 | -3.08846 | -2.34875 |
| C | -3.13356 | -2.60178 | 0.819289 |
| H | -4.41248 | -0.95965 | -1.06495 |
| H | 1.754813 | -2.9701 | -0.74598 |
| H | 1.673527 | -1.68102 | -1.92456 |
| O | -2.73761 | 0.257473 | -1.55195 |
| H | -2.55445 | -3.45556 | 1.176874 |
| H | -3.16968 | -1.85353 | 1.61927 |
| H | -4.1575 | -2.93912 | 0.626188 |
| H | -0.64858 | -0.92261 | 1.743476 |
| C | -0.15701 | 3.64058 | -1.08645 |
| C | 1.240316 | 3.729163 | -0.53122 |
| C | 0.295862 | 2.635404 | -0.07002 |
| C | 2.396763 | 3.027553 | -1.19244 |
| C | 0.882769 | 1.278974 | -0.54996 |
| C | 2.399009 | 1.655726 | -0.56439 |
| C | 3.094589 | -0.5559 | -0.04415 |
| C | 0.699167 | 0.123797 | 0.470952 |
| C | 1.681365 | -1.05091 | 0.17695 |
| C | 3.898925 | -1.09159 | 0.878707 |
| C | 3.062489 | -1.92076 | 1.786017 |
| O | 1.76185 | -1.84426 | 1.371159 |
| O | 3.401689 | -2.55108 | 2.748952 |
| C | 3.324655 | 0.53698 | -1.04253 |
| C | 3.235992 | 3.503319 | -2.10608 |
| H | -0.31088 | 3.33857 | -2.11517 |
| H | -0.84947 | 4.402459 | -0.74186 |
| H | 1.469095 | 4.572633 | 0.113858 |
| H | -0.12373 | 2.68422 | 0.927372 |
| C | 0.402113 | 0.938689 | -1.96794 |
| H | 4.371507 | 0.857357 | -1.04032 |
| H | 3.137976 | 4.512052 | -2.49529 |
| H | 4.054119 | 2.901281 | -2.49315 |
| H | -0.68771 | 0.914492 | -2.0205 |
| H | 0.770773 | 1.683524 | -2.67958 |
| H | 0.765143 | -0.02639 | -2.3162 |
| H | 2.657291 | 1.834776 | 0.492886 |
| H | 3.070412 | 0.228041 | -2.06449 |
| H | 1.046899 | 0.529146 | 1.431722 |
| C | -1.54786 | 1.829715 | 3.00205 |
| H | -1.24011 | 2.880091 | 2.950516 |
| H | -2.25392 | 1.750975 | 3.834359 |
| H | -0.66911 | 1.217175 | 3.21958 |
| C | 5.360326 | -0.94321 | 1.136219 |
| H | 5.529485 | -0.5441 | 2.141074 |
| H | 5.852469 | -1.91993 | 1.096173 |
| H | 5.833912 | -0.28136 | 0.407876 |

**C9'*Re*-TS-*exo*-*Re***

| C | -2.34523 | 4.475775 | 0.184167 |
| --- | --- | --- | --- |
| C | -1.35954 | 3.515741 | 0.805029 |
| C | -2.81976 | 3.147099 | 0.698859 |
| C | -0.62382 | 2.615762 | -0.1322 |
| C | -2.9641 | 1.982847 | -0.29853 |
| C | -1.5079 | 1.641925 | -0.63561 |
| C | -1.96143 | -0.68627 | -0.94409 |
| C | -3.67663 | 0.787441 | 0.303799 |
| C | -3.11194 | -0.53149 | 0.001904 |
| C | -1.87589 | -2.00051 | -1.25961 |
| C | -3.03215 | -2.67965 | -0.62696 |
| O | -3.80174 | -1.73895 | 0.026448 |
| O | -3.32261 | -3.8432 | -0.65655 |
| C | -1.12292 | 0.433777 | -1.18656 |
| C | 0.754331 | 2.546562 | -0.34172 |
| H | -2.2823 | 4.645484 | -0.88605 |
| H | -2.61461 | 5.353502 | 0.763178 |
| H | -0.89827 | 3.773918 | 1.753771 |
| H | -3.44729 | 3.136557 | 1.584637 |
| C | -3.73044 | 2.361398 | -1.57849 |
| H | -4.73762 | 0.881853 | 0.536868 |
| H | 1.050258 | 2.171469 | -1.31164 |
| H | 1.376883 | 3.360362 | 0.024495 |
| O | -2.90504 | 0.06397 | 1.251201 |
| H | -3.22644 | 3.159621 | -2.12724 |
| H | -3.80912 | 1.494259 | -2.24223 |
| H | -4.74074 | 2.70211 | -1.32785 |
| H | -0.16043 | 0.293446 | -1.66761 |
| C | 5.350225 | 1.895953 | -0.45913 |
| C | 5.405615 | 0.722899 | 0.48003 |
| C | 4.179106 | 1.615752 | 0.439294 |
| C | 4.960163 | -0.62903 | -0.01773 |
| C | 3.004077 | 0.767964 | -0.0955 |
| C | 3.481179 | -0.6748 | 0.266598 |
| C | 1.209684 | -1.45397 | 0.320056 |
| C | 1.667852 | 1.029406 | 0.633012 |
| C | 0.877023 | -0.1175 | 0.765024 |
| C | 0.310345 | -2.30335 | 0.87918 |
| C | -0.5685 | -1.4986 | 1.751276 |
| O | -0.16839 | -0.16421 | 1.63718 |
| O | -1.42949 | -1.8527 | 2.504298 |
| C | 2.550162 | -1.73969 | -0.31199 |
| C | 5.707538 | -1.56933 | -0.58688 |
| H | 5.273329 | 1.713203 | -1.52518 |
| H | 5.968737 | 2.749137 | -0.19891 |
| H | 6.106701 | 0.762299 | 1.308197 |
| H | 3.976065 | 2.311054 | 1.248948 |
| C | 2.895151 | 0.85618 | -1.63088 |
| H | 2.875478 | -2.74816 | -0.03403 |
| H | 6.772616 | -1.42943 | -0.74554 |
| H | 5.280489 | -2.51716 | -0.90431 |
| H | 2.948701 | 1.888733 | -1.98411 |
| H | 3.717566 | 0.305167 | -2.09836 |
| H | 1.963734 | 0.4109 | -1.99544 |
| H | 3.401218 | -0.74124 | 1.363016 |
| H | 2.532199 | -1.69672 | -1.40753 |
| H | 1.713675 | 1.640433 | 1.532562 |
| C | -0.94634 | -2.72752 | -2.16729 |
| H | -0.77537 | -3.74275 | -1.79769 |
| H | -1.37357 | -2.81755 | -3.17272 |
| H | 0.01299 | -2.20679 | -2.24773 |
| C | 0.219808 | -3.79099 | 0.877722 |
| H | 0.464869 | -4.19268 | 1.867318 |
| H | -0.80353 | -4.11223 | 0.65177 |
| H | 0.89791 | -4.23072 | 0.141992 |

**C9'*Re*-*exo*-*Re*-29**

| C | 0.707851 | 4.56253 | 0.226068 |
| --- | --- | --- | --- |
| C | 0.696857 | 3.691485 | -1.00586 |
| C | 1.91943 | 3.747683 | -0.12136 |
| C | 0.129033 | 2.326542 | -0.80465 |
| C | 2.085204 | 2.378334 | 0.544381 |
| C | 0.842669 | 1.630627 | 0.083274 |
| C | 1.952123 | -0.52938 | 0.462767 |
| C | 3.291729 | 1.614407 | 0.01286 |
| C | 3.165132 | 0.155246 | -0.068 |
| C | 2.351635 | -1.67296 | 1.033717 |
| C | 3.820877 | -1.8 | 0.799622 |
| O | 4.255709 | -0.69208 | 0.116953 |
| O | 4.555569 | -2.67521 | 1.155632 |
| C | 0.621033 | 0.183576 | 0.432379 |
| C | -1.04141 | 1.802912 | -1.57109 |
| H | 0.081184 | 4.253761 | 1.058565 |
| H | 0.785381 | 5.63582 | 0.085045 |
| H | 0.662998 | 4.150459 | -1.98909 |
| H | 2.812598 | 4.275878 | -0.44093 |
| C | 2.231781 | 2.45907 | 2.070102 |
| H | 4.284374 | 2.038974 | 0.165311 |
| H | -1.8711 | 2.522785 | -1.5365 |
| H | -0.743 | 1.731187 | -2.62524 |
| O | 3.130839 | 0.914492 | -1.22872 |
| H | 1.385705 | 2.991454 | 2.512654 |
| H | 2.28106 | 1.457637 | 2.513694 |
| H | 3.149178 | 2.991106 | 2.343428 |
| H | 0.192817 | 0.07578 | 1.43892 |
| C | -5.05121 | 1.641281 | 0.162885 |
| C | -5.14228 | 0.164869 | -0.10635 |
| C | -4.01322 | 0.986233 | -0.70334 |
| C | -4.48975 | -0.81725 | 0.835222 |
| C | -2.69037 | 0.484281 | -0.06605 |
| C | -3.09683 | -0.97511 | 0.280634 |
| C | -0.88972 | -1.79182 | -0.06027 |
| C | -1.52734 | 0.430577 | -1.08133 |
| C | -0.36017 | -0.47573 | -0.59109 |
| C | -0.38411 | -2.78986 | -0.79111 |
| C | 0.464854 | -2.19212 | -1.85737 |
| O | 0.419575 | -0.83048 | -1.7355 |
| O | 1.077585 | -2.75197 | -2.72182 |
| C | -1.98839 | -1.77817 | 0.964224 |
| C | -5.01765 | -1.40515 | 1.903272 |
| H | -4.79443 | 1.986626 | 1.157231 |
| H | -5.78316 | 2.26171 | -0.34481 |
| H | -5.96491 | -0.1902 | -0.72012 |
| H | -4.00572 | 1.222681 | -1.76419 |
| C | -2.35243 | 1.305649 | 1.187396 |
| H | -2.31633 | -2.79599 | 1.199969 |
| H | -6.03947 | -1.2078 | 2.212751 |
| H | -4.44649 | -2.1111 | 2.50068 |
| H | -2.32495 | 2.372755 | 0.943241 |
| H | -3.1075 | 1.151862 | 1.963637 |
| H | -1.39074 | 1.045735 | 1.626334 |
| H | -3.25122 | -1.45612 | -0.69947 |
| H | -1.67777 | -1.30053 | 1.903495 |
| H | -1.91891 | -0.11365 | -1.95173 |
| C | 1.646109 | -2.70462 | 1.847561 |
| H | 1.480309 | -3.61711 | 1.265444 |
| H | 2.267859 | -2.97806 | 2.705023 |
| H | 0.676726 | -2.34376 | 2.201622 |
| C | -0.56847 | -4.26782 | -0.72624 |
| H | -1.08014 | -4.62969 | -1.62387 |
| H | 0.406784 | -4.76506 | -0.70029 |
| H | -1.14324 | -4.56706 | 0.153161 |

**31** (with b3lyp/6-31G(d,p)-LANL2DZ)

| C | 0.410191 | 1.645221 | -0.97668 |
| --- | --- | --- | --- |
| C | 0.099735 | 2.851769 | -0.09758 |
| C | 1.448571 | 3.587326 | -0.05004 |
| C | 2.449688 | 2.797205 | -0.88753 |
| H | 1.739238 | 4.034562 | 0.894542 |
| H | 3.484566 | 2.666765 | -0.58389 |
| C | -1.06141 | 3.759525 | -0.57862 |
| H | -2.02442 | 3.242763 | -0.59394 |
| H | -1.15381 | 4.622418 | 0.087896 |
| H | -0.8603 | 4.126133 | -1.58815 |
| C | 1.979637 | 4.170864 | -1.33236 |
| H | 1.338411 | 4.228854 | -2.20701 |
| H | 2.685202 | 4.991988 | -1.24111 |
| C | -0.29916 | 2.187178 | 1.213344 |
| C | -0.52682 | 0.501905 | -1.22101 |
| H | -0.86342 | 0.528 | -2.2627 |
| C | -1.73394 | 0.596649 | -0.30033 |
| C | -2.99392 | 0.243387 | -0.64096 |
| O | -4.71636 | 1.51616 | 0.446842 |
| C | -3.45433 | -0.29609 | -1.9674 |
| H | -2.67089 | -0.30655 | -2.72385 |
| H | -3.80727 | -1.32673 | -1.84639 |
| H | -4.30213 | 0.296703 | -2.32976 |
| C | -1.49115 | 1.183446 | 1.061053 |
| O | -2.19389 | 0.960907 | 2.030456 |
| C | 2.672024 | -1.32653 | -0.31592 |
| C | 4.117472 | -0.74738 | -0.28114 |
| C | 4.449557 | -0.3605 | 1.154576 |
| H | 4.457609 | -0.12261 | -1.09776 |
| H | 4.996194 | 0.548382 | 1.39302 |
| C | 2.71819 | -2.87478 | -0.30462 |
| H | 1.737533 | -3.31673 | -0.48736 |
| H | 3.393952 | -3.23209 | -1.08816 |
| H | 3.075896 | -3.26596 | 0.651537 |
| C | 5.195152 | -1.48591 | 0.47682 |
| H | 5.034544 | -2.50266 | 0.815436 |
| H | 6.220395 | -1.28001 | 0.180961 |
| C | 1.76489 | -0.89241 | -1.53224 |
| C | -0.03473 | -1.67416 | 0.166249 |
| C | -0.92386 | -2.65921 | -0.04676 |
| C | -1.22854 | -2.7035 | -1.49218 |
| O | -1.9642 | -3.44346 | -2.10187 |
| H | 1.866794 | -1.66596 | -2.29928 |
| C | -1.55373 | -3.6243 | 0.903678 |
| H | -2.54531 | -3.26273 | 1.201637 |
| H | -1.69192 | -4.59778 | 0.425586 |
| H | -0.95822 | -3.74925 | 1.812078 |
| C | 0.226514 | -0.93296 | -1.14078 |
| O | -0.48605 | -1.72742 | -2.12099 |
| C | 2.081935 | -0.74057 | 1.010869 |
| H | 1.889747 | 0.315997 | 0.78542 |
| C | 0.746937 | -1.36983 | 1.414451 |
| H | 0.19847 | -0.69987 | 2.088535 |
| H | 0.898437 | -2.3016 | 1.972198 |
| C | 1.683105 | 1.62381 | -1.39828 |
| C | 2.142143 | 0.452851 | -2.21433 |
| H | 1.635667 | 0.477233 | -3.18943 |
| H | 3.208953 | 0.490284 | -2.43811 |
| C | 3.239971 | -0.70489 | 1.982795 |
| C | 3.199875 | -0.89525 | 3.301908 |
| H | 4.095002 | -0.82365 | 3.912598 |
| H | 2.271078 | -1.10895 | 3.82233 |
| O | 0.234756 | 2.34739 | 2.289099 |
| C | -4.14161 | 0.457639 | 0.318626 |
| O | -4.51579 | -0.69815 | 0.893841 |
| C | -5.61339 | -0.5907 | 1.821544 |
| H | -5.81753 | -1.60835 | 2.152207 |
| H | -5.32489 | 0.038431 | 2.666485 |
| H | -6.48946 | -0.15939 | 1.332483 |

**Zn(BH_4_)_2_** (with b3lyp/6-31G(d,p)-LANL2DZ)

| H | -1.61198 | 0.488045 | -1.05166 |
| --- | --- | --- | --- |
| B | -2.0739 | -0.03164 | -0.00955 |
| H | -3.257 | 0.094063 | -0.02168 |
| H | -1.62556 | 0.582434 | 0.987063 |
| H | -1.73804 | -1.21594 | 0.048041 |
| Zn | -0.02271 | 0.013251 | 0.005652 |
| H | 2.796594 | 0.024509 | 1.003783 |
| H | 1.487464 | 1.05715 | -0.04119 |
| H | 2.717918 | -0.09726 | -1.07131 |
| H | 1.445647 | -1.06926 | 0.085527 |
| B | 2.167159 | -0.02062 | -0.01208 |

**Int-α-OH-1** (with b3lyp/6-31G(d,p)-LANL2DZ)

| C | 0.513085 | -1.22422 | 1.776493 |
| --- | --- | --- | --- |
| C | -0.92563 | -1.50652 | 2.210893 |
| C | -0.96165 | -3.03145 | 2.359669 |
| C | 0.40487 | -3.56213 | 1.944903 |
| H | -1.87489 | -3.54364 | 2.077689 |
| H | 0.529203 | -4.47706 | 1.372764 |
| C | -1.41875 | -0.75876 | 3.477967 |
| H | -1.34692 | 0.32784 | 3.379116 |
| H | -2.46413 | -1.01094 | 3.676322 |
| H | -0.81581 | -1.06398 | 4.336676 |
| C | -0.0623 | -3.65367 | 3.389632 |
| H | 0.439665 | -3.02853 | 4.122497 |
| H | -0.35005 | -4.63428 | 3.756956 |
| C | -1.70531 | -1.00707 | 1.025973 |
| C | 1.019733 | 0.170021 | 1.529051 |
| H | 1.363712 | 0.582509 | 2.48393 |
| C | -0.12116 | 1.065657 | 1.028527 |
| C | -0.12631 | 2.42194 | 1.135805 |
| O | -2.21047 | 3.586144 | 1.324655 |
| C | 0.896025 | 3.254778 | 1.851148 |
| H | 1.687859 | 2.67201 | 2.318491 |
| H | 1.349878 | 3.968541 | 1.153978 |
| H | 0.383542 | 3.848491 | 2.617963 |
| C | -1.33851 | 0.408801 | 0.516173 |
| O | -2.16251 | 0.942375 | -0.2313 |
| C | 2.364067 | -1.491 | -1.37878 |
| C | 1.743824 | -2.88851 | -1.67532 |
| C | 0.593162 | -2.69811 | -2.65621 |
| H | 1.640766 | -3.59913 | -0.86422 |
| H | -0.33762 | -3.25024 | -2.55468 |
| C | 3.567883 | -1.20237 | -2.30899 |
| H | 4.045967 | -0.25206 | -2.05624 |
| H | 4.321453 | -1.99025 | -2.20795 |
| H | 3.273739 | -1.14805 | -3.35997 |
| C | 1.828883 | -3.46177 | -3.0707 |
| H | 2.499496 | -3.0306 | -3.80458 |
| H | 1.721958 | -4.53974 | -3.15823 |
| C | 2.855667 | -1.21076 | 0.100711 |
| C | 2.36649 | 1.18218 | -0.52145 |
| C | 3.324877 | 2.100722 | -0.31232 |
| C | 3.977977 | 1.810319 | 0.985328 |
| O | 4.878352 | 2.393239 | 1.537237 |
| H | 3.938188 | -1.06989 | 0.042259 |
| C | 3.786877 | 3.240254 | -1.16219 |
| H | 4.876919 | 3.226141 | -1.26031 |
| H | 3.338288 | 3.211891 | -2.1579 |
| H | 3.52977 | 4.200878 | -0.70033 |
| C | 2.357748 | 0.18862 | 0.636499 |
| O | 3.383073 | 0.688854 | 1.529234 |
| C | 1.159139 | -0.56015 | -1.73932 |
| H | 0.438753 | -0.72526 | -0.92355 |
| C | 1.517422 | 0.92526 | -1.73761 |
| H | 0.609091 | 1.54079 | -1.71993 |
| H | 2.06485 | 1.207456 | -2.64387 |
| C | 1.212556 | -2.35956 | 1.600442 |
| C | 2.635353 | -2.3276 | 1.140003 |
| H | 3.290259 | -2.12804 | 1.999243 |
| H | 2.954761 | -3.29341 | 0.738755 |
| C | 0.510565 | -1.21948 | -2.93556 |
| C | -0.04989 | -0.62202 | -3.98719 |
| H | -0.51616 | -1.19353 | -4.78338 |
| H | -0.09407 | 0.459192 | -4.07746 |
| O | -2.60664 | -1.59807 | 0.447742 |
| C | -1.28168 | 3.25593 | 0.62386 |
| O | -1.0632 | 3.651419 | -0.63578 |
| C | -2.12568 | 4.426593 | -1.23922 |
| H | -1.7324 | 4.760143 | -2.1982 |
| H | -3.00221 | 3.791903 | -1.38441 |
| H | -2.38576 | 5.275438 | -0.60444 |
| H | -5.22996 | -0.71104 | 1.983796 |
| B | -5.55492 | -0.2467 | 0.920325 |
| H | -6.67207 | 0.177805 | 0.805318 |
| H | -5.44153 | -1.16781 | 0.067741 |
| H | -4.79333 | 0.728711 | 0.691888 |
| Zn | -4.01332 | -0.23594 | -0.74433 |
| H | -3.07305 | 0.287492 | -3.65434 |
| H | -3.54233 | -1.09919 | -2.34077 |
| H | -4.36577 | 0.831174 | -2.2699 |
| H | -4.96917 | -0.52387 | -3.58237 |
| B | -3.99633 | -0.12271 | -3.00275 |

**TS-α-OH** (with b3lyp/6-31G(d,p)-LANL2DZ)

| C | 0.305377 | -0.85657 | 1.938771 |
| --- | --- | --- | --- |
| C | -1.15739 | -0.8217 | 2.394411 |
| C | -1.41659 | -2.27871 | 2.804367 |
| C | -0.15267 | -3.07759 | 2.526687 |
| H | -2.39343 | -2.69431 | 2.585003 |
| H | -0.17639 | -4.08558 | 2.122194 |
| C | -1.43721 | 0.186645 | 3.528497 |
| H | -1.2924 | 1.221736 | 3.207362 |
| H | -2.47121 | 0.089232 | 3.868627 |
| H | -0.77219 | -0.00565 | 4.373912 |
| C | -0.6246 | -2.84281 | 3.952518 |
| H | -0.03278 | -2.18442 | 4.581799 |
| H | -1.06194 | -3.68813 | 4.476134 |
| C | -1.88031 | -0.44791 | 1.093992 |
| C | 1.025138 | 0.37366 | 1.455058 |
| H | 1.468147 | 0.880158 | 2.318885 |
| C | 0.01549 | 1.344286 | 0.825464 |
| C | 0.158917 | 2.689426 | 0.782686 |
| O | -2.05756 | 3.637405 | 0.733098 |
| C | 1.325815 | 3.466814 | 1.325372 |
| H | 2.05453 | 2.844587 | 1.842996 |
| H | 1.831594 | 3.985332 | 0.503737 |
| H | 0.971039 | 4.241668 | 2.015328 |
| C | -1.23117 | 0.726774 | 0.308827 |
| O | -1.76435 | 1.043607 | -0.7578 |
| C | 2.034833 | -2.01699 | -1.06711 |
| C | 1.261597 | -3.36803 | -1.06872 |
| C | 0.073531 | -3.2411 | -2.01374 |
| H | 1.141869 | -3.90493 | -0.13607 |
| H | -0.89692 | -3.66088 | -1.76386 |
| C | 3.227239 | -2.05274 | -2.05317 |
| H | 3.819113 | -1.13513 | -1.9976 |
| H | 3.890326 | -2.89021 | -1.81289 |
| H | 2.901433 | -2.16657 | -3.0902 |
| C | 1.203351 | -4.19193 | -2.3339 |
| H | 1.861753 | -3.97338 | -3.16674 |
| H | 0.990599 | -5.2508 | -2.21294 |
| C | 2.601477 | -1.50944 | 0.322993 |
| C | 2.374546 | 0.753453 | -0.78648 |
| C | 3.432143 | 1.582107 | -0.79662 |
| C | 4.114574 | 1.483693 | 0.514291 |
| O | 5.099298 | 2.058385 | 0.909866 |
| H | 3.691639 | -1.53878 | 0.242571 |
| C | 3.966693 | 2.470344 | -1.87377 |
| H | 5.051593 | 2.355114 | -1.95898 |
| H | 3.507249 | 2.255709 | -2.84172 |
| H | 3.782082 | 3.525311 | -1.63868 |
| C | 2.311203 | 0.026922 | 0.552382 |
| O | 3.433447 | 0.568668 | 1.291261 |
| C | 0.92064 | -1.04472 | -1.5741 |
| H | 0.238858 | -0.96007 | -0.71763 |
| C | 1.439573 | 0.355923 | -1.89638 |
| H | 0.603873 | 1.061963 | -1.97976 |
| H | 1.965308 | 0.380183 | -2.85769 |
| C | 0.82967 | -2.09065 | 1.995114 |
| C | 2.244354 | -2.34268 | 1.571515 |
| H | 2.927753 | -2.0509 | 2.380748 |
| H | 2.434628 | -3.40378 | 1.389104 |
| C | 0.116052 | -1.84325 | -2.57303 |
| C | -0.47701 | -1.39401 | -3.68127 |
| H | -1.06426 | -2.04879 | -4.31765 |
| H | -0.41092 | -0.35272 | -3.98388 |
| O | -2.42277 | -1.32173 | 0.343267 |
| C | -0.95194 | 3.563562 | 0.246542 |
| O | -0.5165 | 4.302012 | -0.78857 |
| C | -1.49514 | 5.195133 | -1.36155 |
| H | -0.9755 | 5.721574 | -2.16107 |
| H | -2.33821 | 4.625197 | -1.75797 |
| H | -1.85908 | 5.895796 | -0.60704 |
| H | -3.00493 | 0.468621 | 1.62871 |
| B | -4.28365 | 0.650172 | 1.359165 |
| H | -4.73314 | 1.48919 | 2.083281 |
| H | -4.70043 | -0.47781 | 1.445714 |
| H | -4.23267 | 1.122309 | 0.208894 |
| Zn | -3.60443 | -0.24818 | -0.93739 |
| H | -4.1785 | -1.39497 | -3.66424 |
| H | -4.74319 | -1.46139 | -1.77592 |
| H | -3.99725 | 0.300195 | -2.67395 |
| H | -5.82969 | -0.3489 | -2.9851 |
| B | -4.70778 | -0.73708 | -2.81091 |

**Int-α-OH-2** (with b3lyp/6-31G(d,p)-LANL2DZ)

| C | 0.098387 | -0.44079 | 2.028508 |
| --- | --- | --- | --- |
| C | -1.23013 | 0.197129 | 2.408002 |
| C | -1.96292 | -0.96779 | 3.099884 |
| C | -1.06355 | -2.19057 | 3.056454 |
| H | -3.02804 | -1.06329 | 2.92401 |
| H | -1.44766 | -3.18923 | 2.865117 |
| C | -1.08478 | 1.424412 | 3.336097 |
| H | -0.62877 | 2.276746 | 2.824143 |
| H | -2.07044 | 1.74341 | 3.687757 |
| H | -0.47103 | 1.188149 | 4.209178 |
| C | -1.38507 | -1.51718 | 4.379581 |
| H | -0.585 | -0.98785 | 4.889166 |
| H | -2.07367 | -2.03058 | 5.045284 |
| C | -1.93052 | 0.640829 | 1.078343 |
| C | 1.177163 | 0.327808 | 1.31331 |
| H | 1.838898 | 0.777955 | 2.060232 |
| C | 0.525848 | 1.499932 | 0.551944 |
| C | 1.095801 | 2.713049 | 0.381372 |
| O | -0.96623 | 3.895383 | -0.00885 |
| C | 2.505298 | 3.079958 | 0.760502 |
| H | 3.016554 | 2.284762 | 1.301808 |
| H | 3.084884 | 3.303374 | -0.1417 |
| H | 2.52034 | 3.988921 | 1.37064 |
| C | -0.87473 | 1.21184 | 0.08973 |
| O | -1.16459 | 1.235749 | -1.11367 |
| C | 1.09568 | -2.74592 | -0.59519 |
| C | -0.07339 | -3.71943 | -0.27358 |
| C | -1.29043 | -3.32397 | -1.10229 |
| H | -0.21826 | -4.05387 | 0.74556 |
| H | -2.29783 | -3.35494 | -0.69811 |
| C | 2.110201 | -3.39091 | -1.56912 |
| H | 2.973623 | -2.74091 | -1.73267 |
| H | 2.479881 | -4.33484 | -1.15535 |
| H | 1.674414 | -3.60291 | -2.54922 |
| C | -0.57709 | -4.63347 | -1.36164 |
| H | -0.01887 | -4.75964 | -2.28185 |
| H | -1.10325 | -5.52944 | -1.04485 |
| C | 1.924433 | -2.17836 | 0.632687 |
| C | 2.359334 | -0.26419 | -0.97819 |
| C | 3.618526 | 0.147129 | -1.19919 |
| C | 4.35294 | 0.100342 | 0.086479 |
| O | 5.503139 | 0.379539 | 0.321513 |
| H | 2.928729 | -2.60453 | 0.555838 |
| C | 4.303069 | 0.566935 | -2.46008 |
| H | 5.288568 | 0.097011 | -2.53316 |
| H | 3.717187 | 0.308857 | -3.34592 |
| H | 4.471077 | 1.650338 | -2.47064 |
| C | 2.187001 | -0.62184 | 0.49172 |
| O | 3.490195 | -0.3547 | 1.062719 |
| C | 0.325561 | -1.56098 | -1.25894 |
| H | -0.13977 | -1.06802 | -0.39697 |
| C | 1.241234 | -0.54904 | -1.9433 |
| H | 0.688164 | 0.367009 | -2.1779 |
| H | 1.642826 | -0.93295 | -2.88792 |
| C | 0.179598 | -1.73487 | 2.367628 |
| C | 1.405843 | -2.534 | 2.042037 |
| H | 2.204249 | -2.30742 | 2.761975 |
| H | 1.232715 | -3.61074 | 2.12351 |
| C | -0.89603 | -2.1348 | -1.92707 |
| C | -1.57195 | -1.61022 | -2.969 |
| H | -2.45549 | -2.09287 | -3.37567 |
| H | -1.18509 | -0.75824 | -3.52268 |
| O | -2.67032 | -0.31992 | 0.46156 |
| C | 0.237969 | 3.823276 | -0.15723 |
| O | 0.968645 | 4.756597 | -0.79346 |
| C | 0.221013 | 5.871793 | -1.31661 |
| H | 0.958386 | 6.5239 | -1.78328 |
| H | -0.51143 | 5.527282 | -2.0501 |
| H | -0.30195 | 6.391518 | -0.51059 |
| H | -2.54651 | 1.562566 | 1.33656 |
| B | -4.0919 | 2.586564 | 1.41532 |
| H | -3.55092 | 3.537599 | 1.890974 |
| H | -4.63079 | 1.78624 | 2.118043 |
| H | -4.21181 | 2.50605 | 0.229623 |
| Zn | -2.9555 | -0.02628 | -1.42328 |
| H | -4.54924 | -0.33596 | -4.00051 |
| H | -4.55316 | -0.79231 | -2.07963 |
| H | -3.67092 | 0.973378 | -2.82331 |
| H | -5.61947 | 0.775022 | -2.62023 |
| B | -4.63188 | 0.155637 | -2.90616 |

**Zn-α-OH** (with b3lyp/6-31G(d,p)-LANL2DZ)

| C | -0.05888 | -0.68938 | 2.007905 |
| --- | --- | --- | --- |
| C | -0.95403 | 0.425605 | 2.532273 |
| C | -2.11729 | -0.36524 | 3.163721 |
| C | -1.86877 | -1.84593 | 2.933378 |
| H | -3.11861 | 0.040591 | 3.078473 |
| H | -2.66885 | -2.53519 | 2.677498 |
| C | -0.27059 | 1.357752 | 3.562131 |
| H | 0.498769 | 1.987007 | 3.105772 |
| H | -1.01609 | 2.022995 | 4.009476 |
| H | 0.194721 | 0.780964 | 4.36505 |
| C | -1.8124 | -1.2652 | 4.335029 |
| H | -0.84401 | -1.2149 | 4.824139 |
| H | -2.63942 | -1.48936 | 5.003099 |
| C | -1.37803 | 1.303283 | 1.316053 |
| C | 1.238474 | -0.40712 | 1.29786 |
| H | 2.050262 | -0.42049 | 2.032143 |
| C | 1.186079 | 1.025924 | 0.739003 |
| C | 2.237515 | 1.87034 | 0.672878 |
| O | 0.918747 | 3.879954 | 0.710721 |
| C | 3.67094 | 1.514737 | 0.956821 |
| H | 3.787088 | 0.495962 | 1.324984 |
| H | 4.262164 | 1.615331 | 0.04011 |
| H | 4.1082 | 2.206176 | 1.68471 |
| C | -0.19116 | 1.47396 | 0.342449 |
| O | -0.43019 | 1.816877 | -0.82072 |
| C | -0.31748 | -2.80411 | -0.91094 |
| C | -1.79742 | -3.14412 | -0.57687 |
| C | -2.70569 | -2.12588 | -1.2554 |
| H | -2.04754 | -3.49204 | 0.416812 |
| H | -3.58477 | -1.7178 | -0.76598 |
| C | 0.233279 | -3.71803 | -2.03075 |
| H | 1.293241 | -3.52425 | -2.21631 |
| H | 0.134056 | -4.76947 | -1.74173 |
| H | -0.29288 | -3.58041 | -2.97936 |
| C | -2.71797 | -3.5755 | -1.69084 |
| H | -2.32251 | -3.84084 | -2.66456 |
| H | -3.59561 | -4.14871 | -1.40588 |
| C | 0.723488 | -2.85487 | 0.285815 |
| C | 1.957232 | -1.15707 | -1.14186 |
| C | 3.258825 | -1.33854 | -1.41914 |
| C | 3.918761 | -1.88418 | -0.2105 |
| O | 5.073347 | -2.19047 | -0.04043 |
| H | 1.411082 | -3.67685 | 0.068138 |
| C | 4.029877 | -1.11439 | -2.67998 |
| H | 4.666814 | -1.97759 | -2.89637 |
| H | 3.370765 | -0.93382 | -3.53282 |
| H | 4.697512 | -0.2506 | -2.57973 |
| C | 1.673415 | -1.58916 | 0.291464 |
| O | 2.965671 | -2.02328 | 0.77834 |
| C | -0.45326 | -1.32346 | -1.39 |
| H | -0.60987 | -0.78199 | -0.44966 |
| C | 0.813382 | -0.77444 | -2.04185 |
| H | 0.74911 | 0.314373 | -2.14789 |
| H | 0.968838 | -1.1843 | -3.04628 |
| C | -0.56951 | -1.9129 | 2.202435 |
| C | 0.145954 | -3.12735 | 1.691192 |
| H | 0.981331 | -3.38313 | 2.357193 |
| H | -0.49947 | -4.01008 | 1.672705 |
| C | -1.81779 | -1.18276 | -2.01065 |
| C | -2.18997 | -0.3137 | -2.97043 |
| H | -3.20758 | -0.29708 | -3.35079 |
| H | -1.46184 | 0.29841 | -3.49621 |
| O | -2.41732 | 0.78606 | 0.521092 |
| C | 1.946457 | 3.317108 | 0.383327 |
| O | 2.970135 | 3.924649 | -0.23592 |
| C | 2.770393 | 5.321946 | -0.53435 |
| H | 3.68737 | 5.648515 | -1.02311 |
| H | 1.910962 | 5.447556 | -1.19637 |
| H | 2.5983 | 5.885735 | 0.385185 |
| H | -1.63925 | 2.315177 | 1.670449 |
| B | -3.91323 | 1.193086 | 0.717248 |
| H | -3.97134 | 2.26476 | 1.277864 |
| H | -4.55924 | 0.312138 | 1.23104 |
| H | -4.31708 | 1.30549 | -0.47706 |
| Zn | -2.57578 | 1.745366 | -1.32307 |
| H | -1.93689 | 3.962848 | -3.19667 |
| H | -3.08162 | 2.356822 | -3.00066 |
| H | -2.71528 | 3.647881 | -1.39162 |
| H | -3.9735 | 4.080953 | -2.83725 |
| B | -2.91476 | 3.555689 | -2.62574 |

**Int-β-OH-1** (with b3lyp/6-31G(d,p)-LANL2DZ)

| C | -0.56511 | -0.98462 | -1.90838 |
| --- | --- | --- | --- |
| C | 0.820263 | -1.08679 | -2.54823 |
| C | 0.934199 | -2.57235 | -2.91203 |
| C | -0.31651 | -3.26649 | -2.38968 |
| H | 1.909783 | -3.03808 | -2.82817 |
| H | -0.29095 | -4.25104 | -1.93195 |
| C | 1.092616 | -0.13826 | -3.74402 |
| H | 1.001773 | 0.916734 | -3.47075 |
| H | 2.103855 | -0.30207 | -4.12669 |
| H | 0.379993 | -0.34892 | -4.54538 |
| C | -0.06184 | -3.1373 | -3.88445 |
| H | -0.70789 | -2.47229 | -4.45022 |
| H | 0.236137 | -4.03566 | -4.41693 |
| C | 1.722185 | -0.70443 | -1.40409 |
| C | -1.12593 | 0.328874 | -1.43614 |
| H | -1.54457 | 0.85348 | -2.30204 |
| C | 0.016658 | 1.194872 | -0.88971 |
| C | 0.006165 | 2.55273 | -0.85013 |
| O | 2.092171 | 3.636989 | -1.23332 |
| C | -1.09433 | 3.442021 | -1.35184 |
| H | -1.9194 | 2.899307 | -1.81067 |
| H | -1.49002 | 4.042301 | -0.52411 |
| H | -0.67748 | 4.146047 | -2.08172 |
| C | 1.274169 | 0.502474 | -0.54107 |
| O | 2.057955 | 0.866204 | 0.33642 |
| C | -2.12245 | -1.66981 | 1.388882 |
| C | -1.31389 | -2.99441 | 1.52625 |
| C | -0.15161 | -2.74183 | 2.481198 |
| H | -1.15388 | -3.60347 | 0.644226 |
| H | 0.83994 | -3.14532 | 2.294175 |
| C | -3.29318 | -1.6101 | 2.399654 |
| H | -3.88651 | -0.70166 | 2.260856 |
| H | -3.95983 | -2.4674 | 2.260563 |
| H | -2.94487 | -1.61568 | 3.435231 |
| C | -1.25703 | -3.69791 | 2.862119 |
| H | -1.94762 | -3.43185 | 3.653978 |
| H | -1.00222 | -4.75416 | 2.843122 |
| C | -2.73435 | -1.33059 | -0.03181 |
| C | -2.4588 | 1.053406 | 0.735394 |
| C | -3.50986 | 1.883725 | 0.627554 |
| C | -4.19122 | 1.613802 | -0.66032 |
| O | -5.16714 | 2.140166 | -1.13538 |
| H | -3.8178 | -1.29423 | 0.107993 |
| C | -4.0392 | 2.915683 | 1.570356 |
| H | -5.11745 | 2.789015 | 1.709299 |
| H | -3.5459 | 2.867193 | 2.543986 |
| H | -3.89528 | 3.924208 | 1.16491 |
| C | -2.40645 | 0.144128 | -0.48725 |
| O | -3.52062 | 0.58972 | -1.29929 |
| C | -1.01981 | -0.62971 | 1.774034 |
| H | -0.32288 | -0.65529 | 0.923781 |
| C | -1.5353 | 0.802781 | 1.897354 |
| H | -0.69533 | 1.509397 | 1.89381 |
| H | -2.07015 | 0.958658 | 2.841065 |
| C | -1.15017 | -2.18989 | -1.78641 |
| C | -2.50275 | -2.34625 | -1.16852 |
| H | -3.26629 | -2.17474 | -1.93985 |
| H | -2.66688 | -3.36606 | -0.80899 |
| C | -0.24607 | -1.29474 | 2.888958 |
| C | 0.273512 | -0.71787 | 3.973427 |
| H | 0.83439 | -1.29144 | 4.704641 |
| H | 0.175751 | 0.348215 | 4.159047 |
| O | 2.773816 | -1.25211 | -1.10216 |
| C | 1.235653 | 3.332083 | -0.43392 |
| O | 1.192264 | 3.693229 | 0.85013 |
| C | 2.363034 | 4.403486 | 1.328793 |
| H | 2.162262 | 4.611444 | 2.378574 |
| H | 3.247298 | 3.773361 | 1.214803 |
| H | 2.497359 | 5.329867 | 0.766759 |
| H | 3.132585 | -3.17391 | 1.640773 |
| B | 3.805753 | -2.22835 | 1.965661 |
| H | 4.39667 | -2.29086 | 3.009385 |
| H | 3.023529 | -1.24445 | 2.067589 |
| H | 4.66713 | -2.0776 | 1.063949 |
| Zn | 3.91995 | -0.38246 | 0.651144 |
| H | 6.566839 | 1.175335 | 0.502213 |
| H | 4.818167 | 1.102372 | 1.413289 |
| H | 4.994229 | 2.369909 | -0.09905 |
| H | 5.079878 | 0.452521 | -0.5661 |
| B | 5.389044 | 1.309674 | 0.310978 |

**TS-β-OH** (with b3lyp/6-31G(d,p)-LANL2DZ)

| C | -0.23587 | -1.19096 | -1.7324 |
| --- | --- | --- | --- |
| C | 1.212235 | -1.36701 | -2.16422 |
| C | 1.349023 | -2.89201 | -2.30392 |
| C | 0.020482 | -3.51262 | -1.9002 |
| H | 2.293552 | -3.33955 | -2.01483 |
| H | -0.04245 | -4.42213 | -1.30927 |
| C | 1.592582 | -0.61405 | -3.46705 |
| H | 1.469499 | 0.469152 | -3.3691 |
| H | 2.638871 | -0.81079 | -3.71087 |
| H | 0.963871 | -0.95513 | -4.29258 |
| C | 0.505948 | -3.59292 | -3.33664 |
| H | -0.0289 | -3.01655 | -4.08592 |
| H | 0.871747 | -4.55341 | -3.68847 |
| C | 2.082141 | -0.77166 | -1.05916 |
| C | -0.82047 | 0.17353 | -1.50199 |
| H | -1.06847 | 0.60779 | -2.47712 |
| C | 0.2413 | 1.086028 | -0.86836 |
| C | 0.224331 | 2.443005 | -0.95341 |
| O | 2.310205 | 3.631547 | -0.97572 |
| C | -0.76374 | 3.268969 | -1.72693 |
| H | -1.48765 | 2.674856 | -2.28151 |
| H | -1.30636 | 3.93719 | -1.04887 |
| H | -0.21159 | 3.907645 | -2.42701 |
| C | 1.479421 | 0.471607 | -0.33981 |
| O | 2.194177 | 0.998202 | 0.521185 |
| C | -2.47904 | -1.47588 | 1.289714 |
| C | -1.8847 | -2.83676 | 1.757292 |
| C | -0.94127 | -2.55971 | 2.919944 |
| H | -1.60498 | -3.57248 | 1.012978 |
| H | 0.001717 | -3.08856 | 3.012953 |
| C | -3.82235 | -1.17926 | 2.003239 |
| H | -4.2815 | -0.26488 | 1.619353 |
| H | -4.52793 | -1.99954 | 1.836133 |
| H | -3.69986 | -1.05209 | 3.081888 |
| C | -2.21395 | -3.34399 | 3.141411 |
| H | -3.0257 | -2.90513 | 3.709097 |
| H | -2.09117 | -4.41056 | 3.310263 |
| C | -2.74142 | -1.28405 | -0.25989 |
| C | -2.45924 | 1.153243 | 0.333356 |
| C | -3.43096 | 2.008984 | -0.02667 |
| C | -3.89965 | 1.642789 | -1.38339 |
| O | -4.7539 | 2.159207 | -2.06216 |
| H | -3.82611 | -1.20369 | -0.36842 |
| C | -4.05665 | 3.146125 | 0.714652 |
| H | -5.14798 | 3.065777 | 0.687043 |
| H | -3.72817 | 3.183472 | 1.755972 |
| H | -3.80541 | 4.103703 | 0.243097 |
| C | -2.25071 | 0.128353 | -0.77591 |
| O | -3.1862 | 0.541678 | -1.80613 |
| C | -1.36963 | -0.49071 | 1.785925 |
| H | -0.51911 | -0.66938 | 1.111277 |
| C | -1.7692 | 0.980352 | 1.659524 |
| H | -0.88637 | 1.627509 | 1.732074 |
| H | -2.44292 | 1.280697 | 2.469947 |
| C | -0.87331 | -2.36006 | -1.57825 |
| C | -2.3216 | -2.42749 | -1.20688 |
| H | -2.92207 | -2.34389 | -2.12344 |
| H | -2.58567 | -3.39537 | -0.77195 |
| C | -0.92174 | -1.06633 | 3.11224 |
| C | -0.54543 | -0.39397 | 4.201366 |
| H | -0.22452 | -0.90848 | 5.102205 |
| H | -0.53009 | 0.69208 | 4.228917 |
| O | 3.348295 | -0.73327 | -1.14455 |
| C | 1.323052 | 3.298671 | -0.3603 |
| O | 0.98772 | 3.723256 | 0.866392 |
| C | 1.968376 | 4.558983 | 1.520424 |
| H | 1.506695 | 4.870381 | 2.456362 |
| H | 2.87668 | 3.983543 | 1.711642 |
| H | 2.209821 | 5.42286 | 0.89801 |
| H | 1.714912 | -1.76144 | 0.086904 |
| B | 2.436349 | -2.28829 | 1.060555 |
| H | 1.771402 | -3.04915 | 1.699508 |
| H | 2.689517 | -1.29215 | 1.755631 |
| H | 3.364791 | -2.74715 | 0.442702 |
| Zn | 3.998612 | -0.29465 | 0.741868 |
| H | 6.522951 | 0.054531 | 2.552816 |
| H | 4.796443 | 0.798312 | 2.034819 |
| H | 6.366945 | 1.308815 | 0.917585 |
| H | 5.831647 | -0.6105 | 0.855733 |
| B | 5.914696 | 0.418475 | 1.586216 |

**Zn-β-OH** (with b3lyp/6-31G(d,p)-LANL2DZ)

| C | -0.57989 | -1.19599 | -1.70593 |
| --- | --- | --- | --- |
| C | 0.850103 | -1.67332 | -1.93325 |
| C | 0.674256 | -3.19046 | -2.11205 |
| C | -0.79388 | -3.52239 | -1.87975 |
| H | 1.465235 | -3.83611 | -1.7453 |
| H | -1.12002 | -4.4093 | -1.34311 |
| C | 1.616577 | -1.03307 | -3.10602 |
| H | 1.678336 | 0.05703 | -3.02377 |
| H | 2.637345 | -1.42349 | -3.13105 |
| H | 1.134392 | -1.26934 | -4.05762 |
| C | -0.17144 | -3.67915 | -3.25632 |
| H | -0.48574 | -2.98573 | -4.03112 |
| H | 0.020963 | -4.68933 | -3.60636 |
| C | 1.536307 | -1.34848 | -0.58174 |
| C | -0.90291 | 0.252945 | -1.46387 |
| H | -1.0859 | 0.725974 | -2.4346 |
| C | 0.313006 | 0.965647 | -0.85416 |
| C | 0.530988 | 2.301731 | -0.99837 |
| O | 2.749405 | 3.152251 | -1.22633 |
| C | -0.32846 | 3.255119 | -1.77865 |
| H | -1.1758 | 2.783192 | -2.27173 |
| H | -0.70451 | 4.044205 | -1.11684 |
| H | 0.297681 | 3.748632 | -2.53159 |
| C | 1.408582 | 0.152348 | -0.27889 |
| O | 2.313019 | 0.687198 | 0.39319 |
| C | -2.70554 | -1.18907 | 1.281738 |
| C | -2.2683 | -2.6486 | 1.602736 |
| C | -1.17996 | -2.59716 | 2.67018 |
| H | -2.18097 | -3.35844 | 0.789586 |
| H | -0.31407 | -3.2532 | 2.630377 |
| C | -3.94088 | -0.77338 | 2.116285 |
| H | -4.28469 | 0.22785 | 1.84202 |
| H | -4.76904 | -1.46764 | 1.940264 |
| H | -3.73122 | -0.76206 | 3.189124 |
| C | -2.52443 | -3.21539 | 2.978049 |
| H | -3.19517 | -2.71252 | 3.664643 |
| H | -2.54604 | -4.2987 | 3.061067 |
| C | -3.03562 | -0.84737 | -0.22976 |
| C | -2.30362 | 1.46481 | 0.442068 |
| C | -3.11666 | 2.492305 | 0.144751 |
| C | -3.67158 | 2.271799 | -1.21136 |
| O | -4.43883 | 2.956073 | -1.84352 |
| H | -4.08889 | -0.55479 | -0.25141 |
| C | -3.51523 | 3.687688 | 0.94871 |
| H | -4.60161 | 3.818554 | 0.924725 |
| H | -3.18817 | 3.606137 | 1.988117 |
| H | -3.08317 | 4.603114 | 0.52728 |
| C | -2.30583 | 0.464889 | -0.71028 |
| O | -3.1678 | 1.084044 | -1.69834 |
| C | -1.43742 | -0.40088 | 1.752453 |
| H | -0.68436 | -0.63914 | 0.991232 |
| C | -1.61984 | 1.115905 | 1.737401 |
| H | -0.64911 | 1.622241 | 1.814621 |
| H | -2.22086 | 1.461552 | 2.586106 |
| C | -1.45356 | -2.21038 | -1.61451 |
| C | -2.89162 | -1.98087 | -1.26552 |
| H | -3.45077 | -1.68937 | -2.16482 |
| H | -3.37124 | -2.89345 | -0.8991 |
| C | -0.94864 | -1.1402 | 2.977456 |
| C | -0.38388 | -0.62047 | 4.068848 |
| H | -0.04415 | -1.24957 | 4.886168 |
| H | -0.23204 | 0.449506 | 4.181187 |
| O | 2.889968 | -1.72884 | -0.51232 |
| C | 1.788216 | 2.986293 | -0.50902 |
| O | 1.634073 | 3.490038 | 0.721722 |
| C | 2.792801 | 4.174542 | 1.252865 |
| H | 2.487468 | 4.542638 | 2.231383 |
| H | 3.628969 | 3.477647 | 1.342709 |
| H | 3.079027 | 5.000664 | 0.598732 |
| H | 0.971294 | -1.87949 | 0.200299 |
| B | 3.320327 | -2.7045 | 0.607446 |
| H | 4.309328 | -3.28246 | 0.238556 |
| H | 2.41187 | -3.39426 | 0.998792 |
| H | 3.634493 | -1.97414 | 1.630161 |
| Zn | 4.055215 | -0.4259 | 0.688094 |
| H | 5.823998 | 1.958179 | 0.683376 |
| H | 5.647813 | 0.123792 | -0.03628 |
| H | 6.903134 | 0.36992 | 1.454369 |
| H | 5.005844 | 0.630812 | 1.913725 |
| B | 5.876699 | 0.805569 | 1.015228 |

**31** (with M06-2X/6-31G(d,p)-LANL2DZ)

| C | 0.499259 | 1.569203 | -1.03763 |
| --- | --- | --- | --- |
| C | 0.319368 | 2.720433 | -0.06567 |
| C | 1.726464 | 3.316147 | 0.008034 |
| C | 2.635066 | 2.508015 | -0.90464 |
| H | 2.073743 | 3.667273 | 0.973354 |
| H | 3.654967 | 2.256815 | -0.63001 |
| C | -0.76401 | 3.74605 | -0.4553 |
| H | -1.76566 | 3.305854 | -0.47823 |
| H | -0.76954 | 4.568614 | 0.264878 |
| H | -0.55139 | 4.152333 | -1.44636 |
| C | 2.279795 | 3.935889 | -1.23982 |
| H | 1.621534 | 4.095246 | -2.08803 |
| H | 3.051161 | 4.687614 | -1.11299 |
| C | -0.11968 | 2.01137 | 1.199969 |
| C | -0.50537 | 0.491859 | -1.28719 |
| H | -0.82086 | 0.515236 | -2.33609 |
| C | -1.70548 | 0.659598 | -0.38492 |
| C | -2.97422 | 0.376198 | -0.71911 |
| O | -4.51034 | 1.77107 | 0.481868 |
| C | -3.46625 | -0.18428 | -2.02065 |
| H | -2.67205 | -0.31525 | -2.75465 |
| H | -3.91052 | -1.16887 | -1.83714 |
| H | -4.24816 | 0.462766 | -2.4313 |
| C | -1.40468 | 1.143203 | 1.006737 |
| O | -2.09347 | 0.893834 | 1.968732 |
| C | 2.565921 | -1.38854 | -0.32715 |
| C | 4.021194 | -0.85491 | -0.36055 |
| C | 4.398391 | -0.40678 | 1.038587 |
| H | 4.36156 | -0.28205 | -1.21387 |
| H | 4.974989 | 0.495911 | 1.216023 |
| C | 2.55171 | -2.92309 | -0.1856 |
| H | 1.550693 | -3.33252 | -0.3405 |
| H | 3.216536 | -3.37081 | -0.93036 |
| H | 2.886118 | -3.24072 | 0.806546 |
| C | 5.083976 | -1.58706 | 0.410439 |
| H | 4.884049 | -2.57456 | 0.808288 |
| H | 6.107513 | -1.43705 | 0.083483 |
| C | 1.666424 | -1.02687 | -1.55683 |
| C | -0.13398 | -1.59888 | 0.215871 |
| C | -1.12313 | -2.48921 | 0.088529 |
| C | -1.46859 | -2.58653 | -1.34751 |
| O | -2.30284 | -3.26351 | -1.88515 |
| H | 1.729467 | -1.85903 | -2.26619 |
| C | -1.84128 | -3.30561 | 1.106581 |
| H | -2.80936 | -2.8417 | 1.323945 |
| H | -2.03374 | -4.31063 | 0.723944 |
| H | -1.27652 | -3.36986 | 2.038981 |
| C | 0.150564 | -0.96777 | -1.14228 |
| O | -0.63547 | -1.75745 | -2.04735 |
| C | 2.023067 | -0.70662 | 0.962727 |
| H | 1.865426 | 0.346287 | 0.698606 |
| C | 0.696679 | -1.2868 | 1.42894 |
| H | 0.189934 | -0.59628 | 2.115206 |
| H | 0.854942 | -2.21834 | 1.987609 |
| C | 1.752088 | 1.456322 | -1.4859 |
| C | 2.076599 | 0.255581 | -2.3203 |
| H | 1.484218 | 0.289894 | -3.24416 |
| H | 3.120686 | 0.215254 | -2.63005 |
| C | 3.198391 | -0.67633 | 1.902996 |
| C | 3.173657 | -0.83623 | 3.222163 |
| H | 4.081131 | -0.78493 | 3.815109 |
| H | 2.240988 | -1.00234 | 3.753016 |
| O | 0.444653 | 2.030895 | 2.26426 |
| C | -4.08051 | 0.667742 | 0.261138 |
| O | -4.57487 | -0.4614 | 0.778431 |
| C | -5.59567 | -0.26351 | 1.760947 |
| H | -5.89593 | -1.25905 | 2.081627 |
| H | -5.1915 | 0.309262 | 2.598289 |
| H | -6.44035 | 0.276466 | 1.329148 |

**Zn(BH_4_)_2_** (with M06-2X/6-31G(d,p)-LANL2DZ)

| H | -1.61746 | -1.20129 | -0.17247 |
| --- | --- | --- | --- |
| B | -2.04926 | -0.05303 | 0.003094 |
| H | -3.23458 | -0.01333 | -0.00606 |
| H | -1.60528 | 0.682153 | -0.91024 |
| H | -1.61722 | 0.374898 | 1.093911 |
| Zn | -0.00023 | 0.027859 | -0.00141 |
| H | 3.235001 | -0.04752 | 0.002031 |
| H | 1.616236 | -0.10851 | -1.16541 |
| H | 1.604286 | -1.01714 | 0.642271 |
| H | 1.626321 | 1.004296 | 0.530757 |
| B | 2.049197 | -0.04884 | 0.002388 |

**Int-α-OH-1** with M06-2X/6-31G(d,p)-LANL2DZ)

| C | 0.697924 | -1.24807 | 1.753212 |
| --- | --- | --- | --- |
| C | -0.73729 | -1.47532 | 2.21709 |
| C | -0.83002 | -2.99037 | 2.345647 |
| C | 0.488058 | -3.57384 | 1.873932 |
| H | -1.77439 | -3.46251 | 2.100442 |
| H | 0.543759 | -4.48694 | 1.290479 |
| C | -1.15894 | -0.71035 | 3.487666 |
| H | -1.08063 | 0.373961 | 3.3646 |
| H | -2.19691 | -0.94368 | 3.735258 |
| H | -0.51479 | -1.01369 | 4.316037 |
| C | 0.085103 | -3.64917 | 3.328398 |
| H | 0.644511 | -3.03577 | 4.027616 |
| H | -0.22709 | -4.61455 | 3.710082 |
| C | -1.52224 | -0.94129 | 1.056379 |
| C | 1.255048 | 0.118668 | 1.468569 |
| H | 1.737093 | 0.500638 | 2.375237 |
| C | 0.12823 | 1.074861 | 1.09319 |
| C | 0.203961 | 2.415647 | 1.194272 |
| O | -1.89376 | 3.515575 | 1.533069 |
| C | 1.361383 | 3.193084 | 1.737676 |
| H | 2.176296 | 2.560015 | 2.089087 |
| H | 1.745741 | 3.855476 | 0.953487 |
| H | 1.014479 | 3.829927 | 2.558393 |
| C | -1.11967 | 0.476673 | 0.575654 |
| O | -1.86091 | 1.015222 | -0.23496 |
| C | 1.962069 | -1.61853 | -1.50481 |
| C | 1.241676 | -2.98585 | -1.59336 |
| C | -0.13767 | -2.7762 | -2.19178 |
| H | 1.371095 | -3.70382 | -0.79348 |
| H | -1.00916 | -3.30368 | -1.81644 |
| C | 2.912412 | -1.39594 | -2.69353 |
| H | 3.484146 | -0.47109 | -2.56817 |
| H | 3.621847 | -2.2253 | -2.77141 |
| H | 2.367617 | -1.31938 | -3.63846 |
| C | 0.90719 | -3.54985 | -2.94558 |
| H | 1.333029 | -3.11118 | -3.83987 |
| H | 0.768245 | -4.62418 | -3.00092 |
| C | 2.775348 | -1.33128 | -0.1872 |
| C | 2.25293 | 1.056001 | -0.76348 |
| C | 3.241889 | 1.953701 | -0.74518 |
| C | 4.101451 | 1.66566 | 0.428654 |
| O | 5.079163 | 2.246707 | 0.807285 |

‘
