# Supplementary material for: Total syntheses of shizukaols A and E
Source: Nat Commun. 2018 Oct 2;9:4040. doi: 10.1038/s41467-018-06245-7 (PMC6168560; doi:10.1038/s41467-018-06245-7)
Supplement: Supplementary file 2 — Supplementary Information [file 41467_2018_6245_MOESM2_ESM.pdf]

nature COMMUNICATIONS

Supporting Information for

Total Syntheses of Shizukaols A and E

Wu *et al*

Total Syntheses of Shizukaols A and E

Jian-Li Wu[+,1], Yin-Suo Lu[+,1,4], Bencan Tang*[,‡,3], Xiao-Shui Peng*[,1,2]

Supplementary Figures

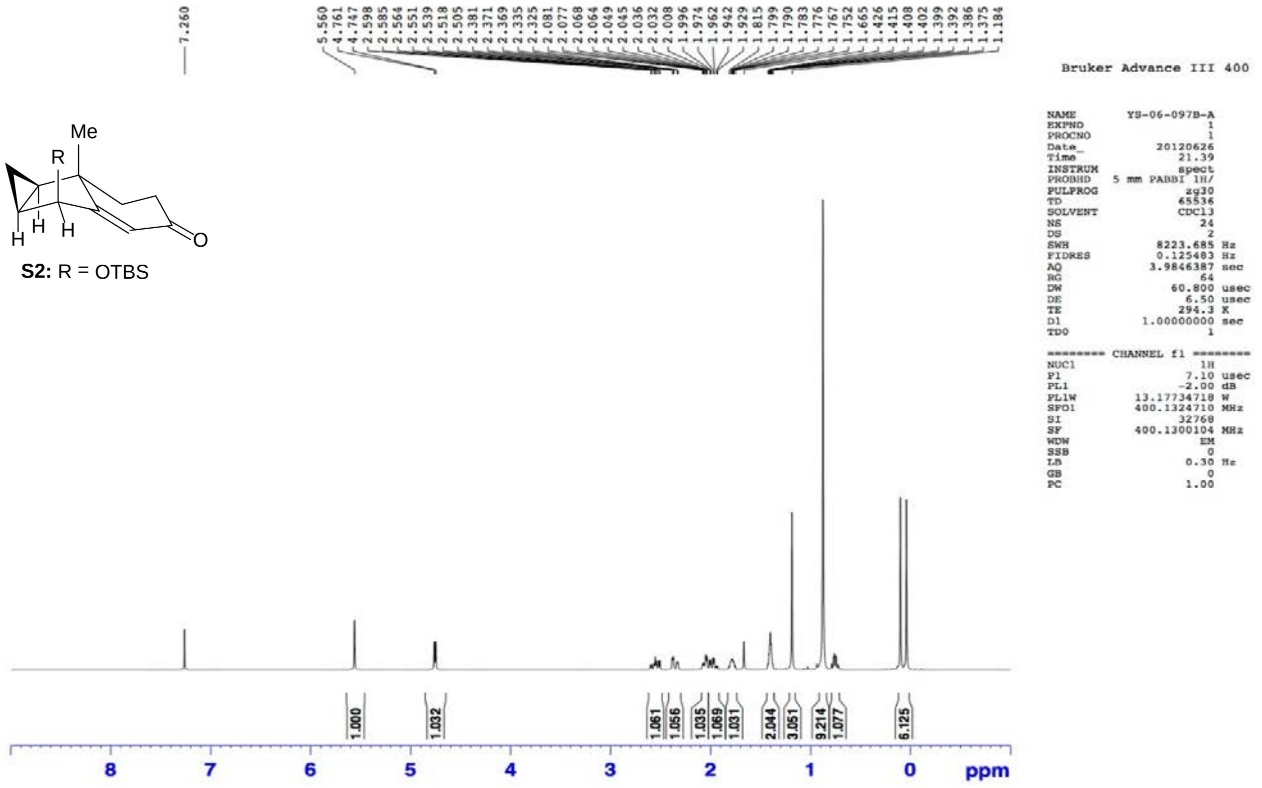

Supplementary Figure 1 ^{1}H NMR spectrum of Compound **S2** in $CDCl_3$

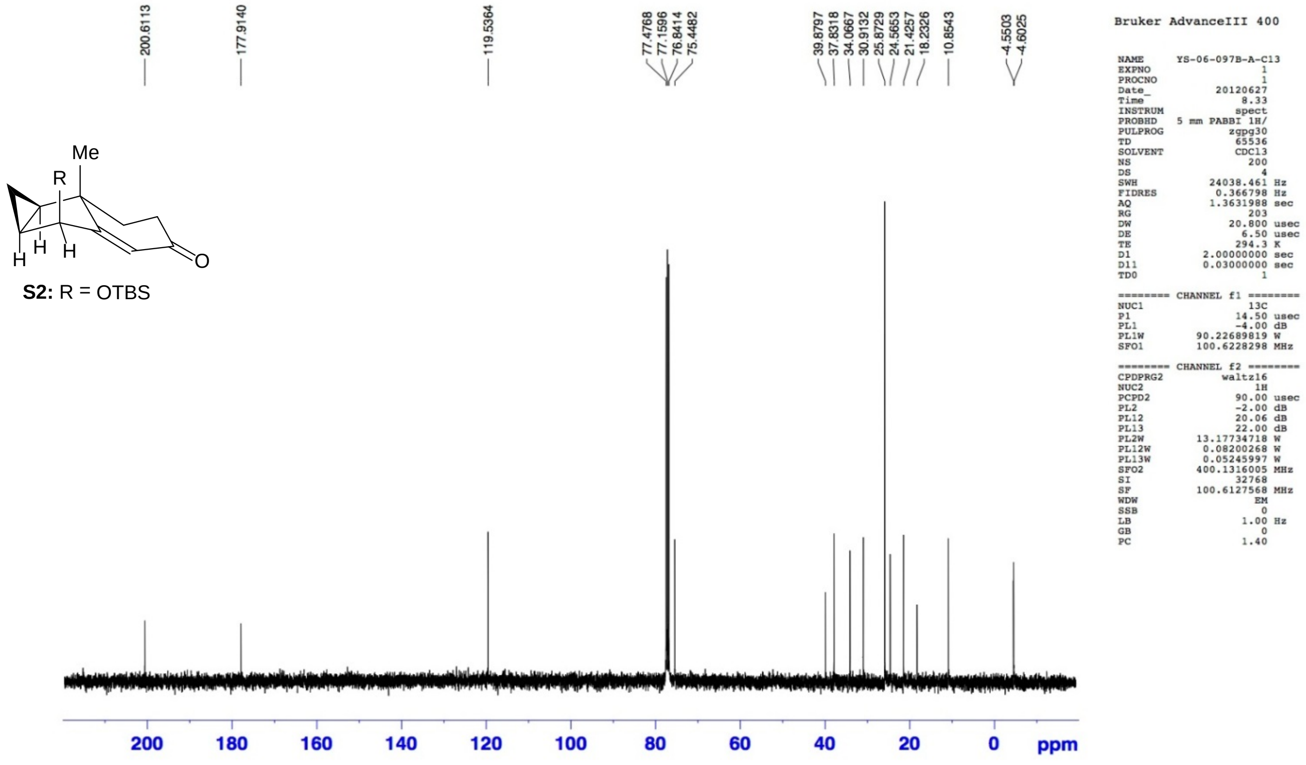

Supplementary Figure 2 ^{13}C NMR spectrum of Compound **S2** in $CDCl_3$

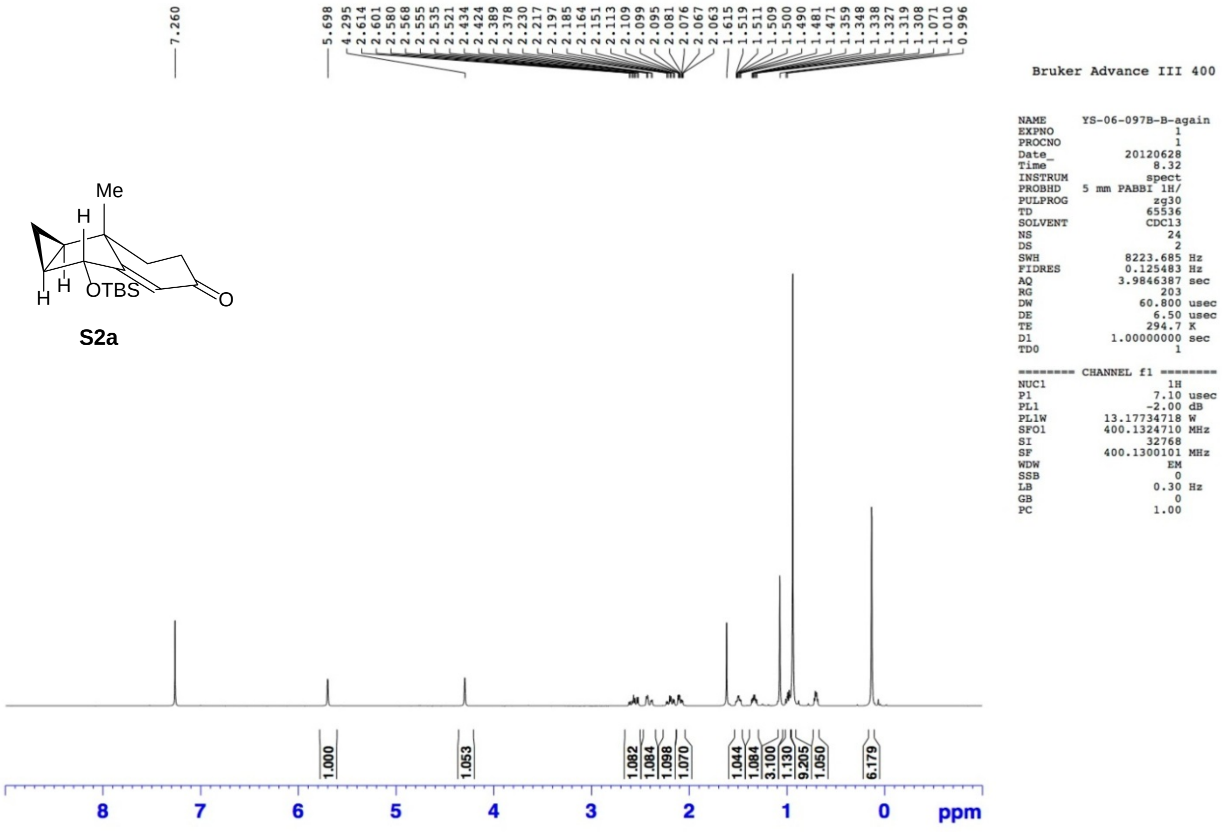

Supplementary Figure 3 ^{1}H NMR spectrum of Compound **S2a** in $CDCl_3$

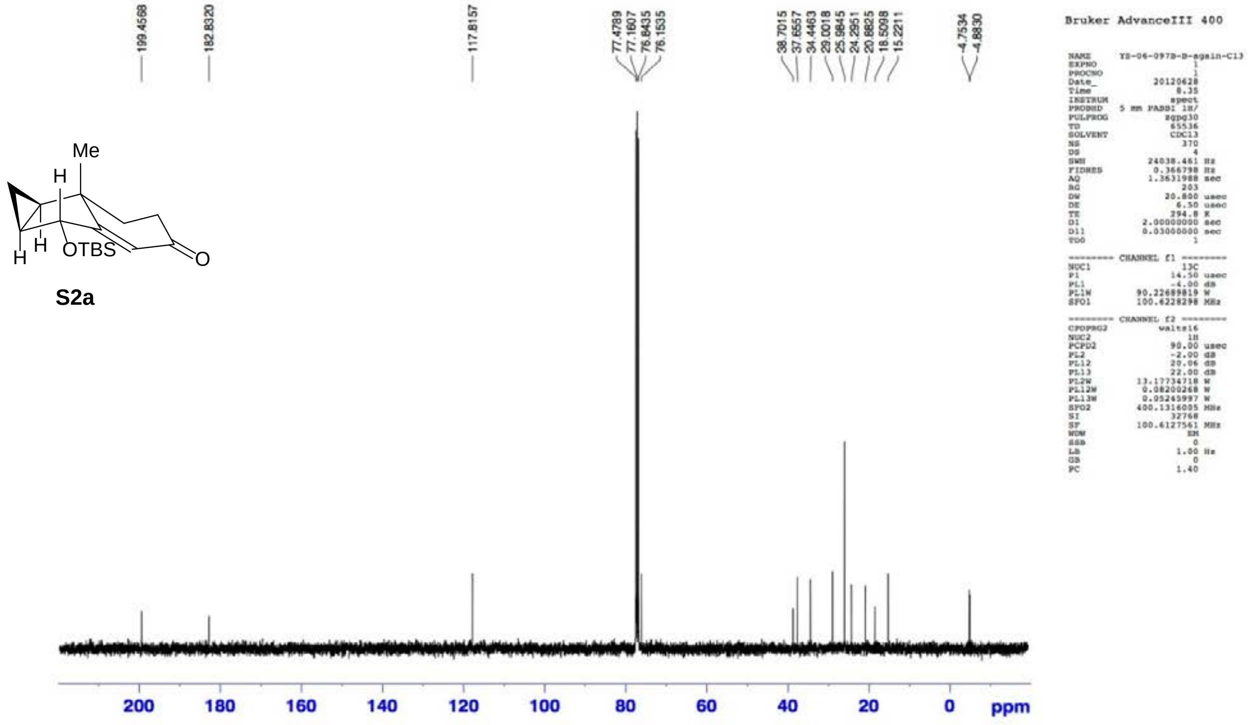

Supplementary Figure 4 ^{13}C NMR spectrum of Compound **S2a** in $CDCl_3$

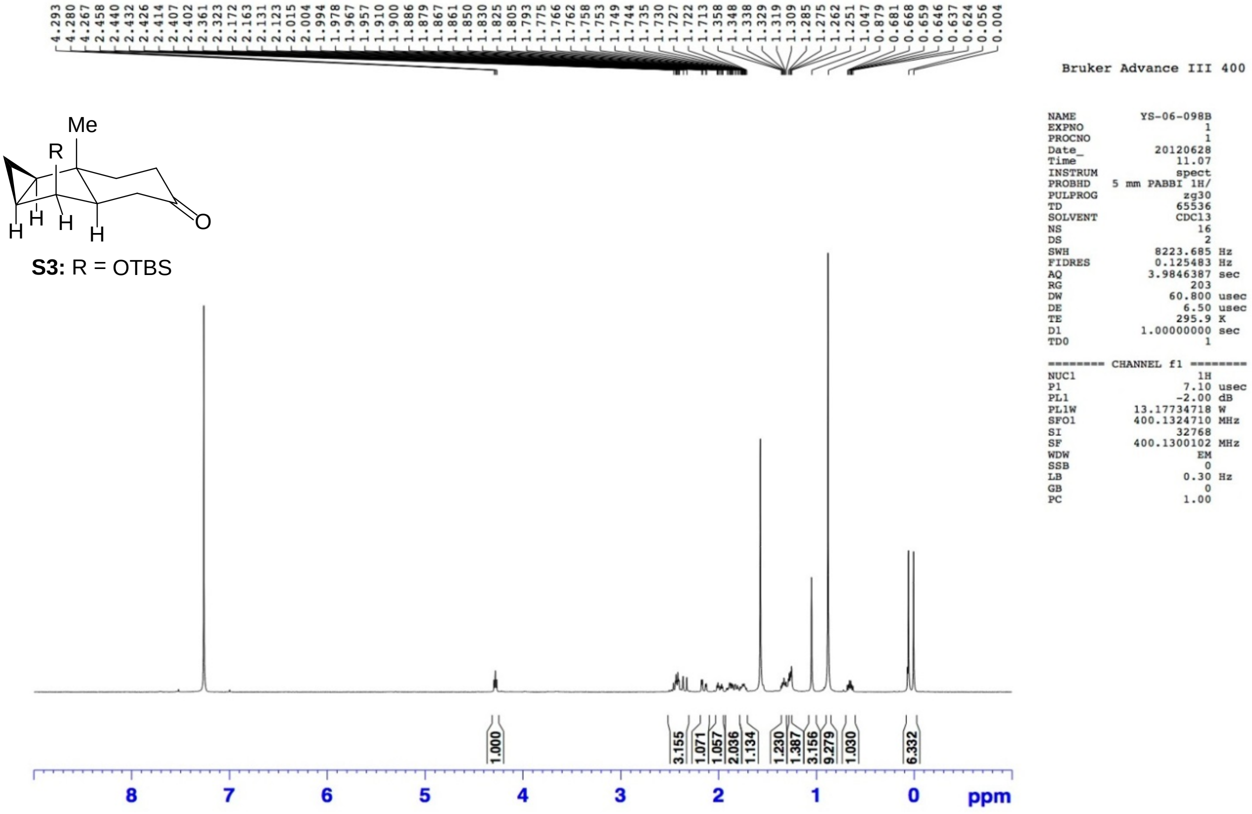

Supplementary Figure 5 ^{1}H NMR spectrum of Compound **S3** in $CDCl_3$

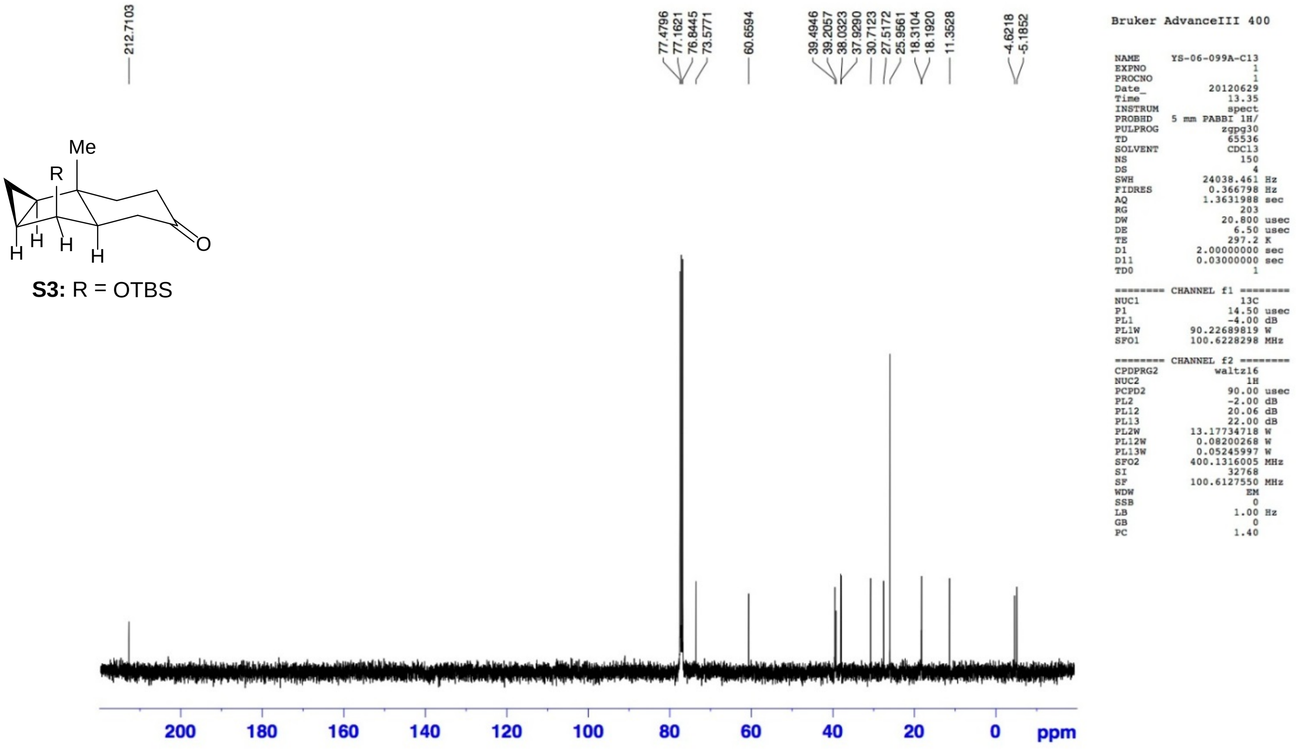

Supplementary Figure 6 ^{13}C NMR spectrum of Compound **S3** in $CDCl_3$

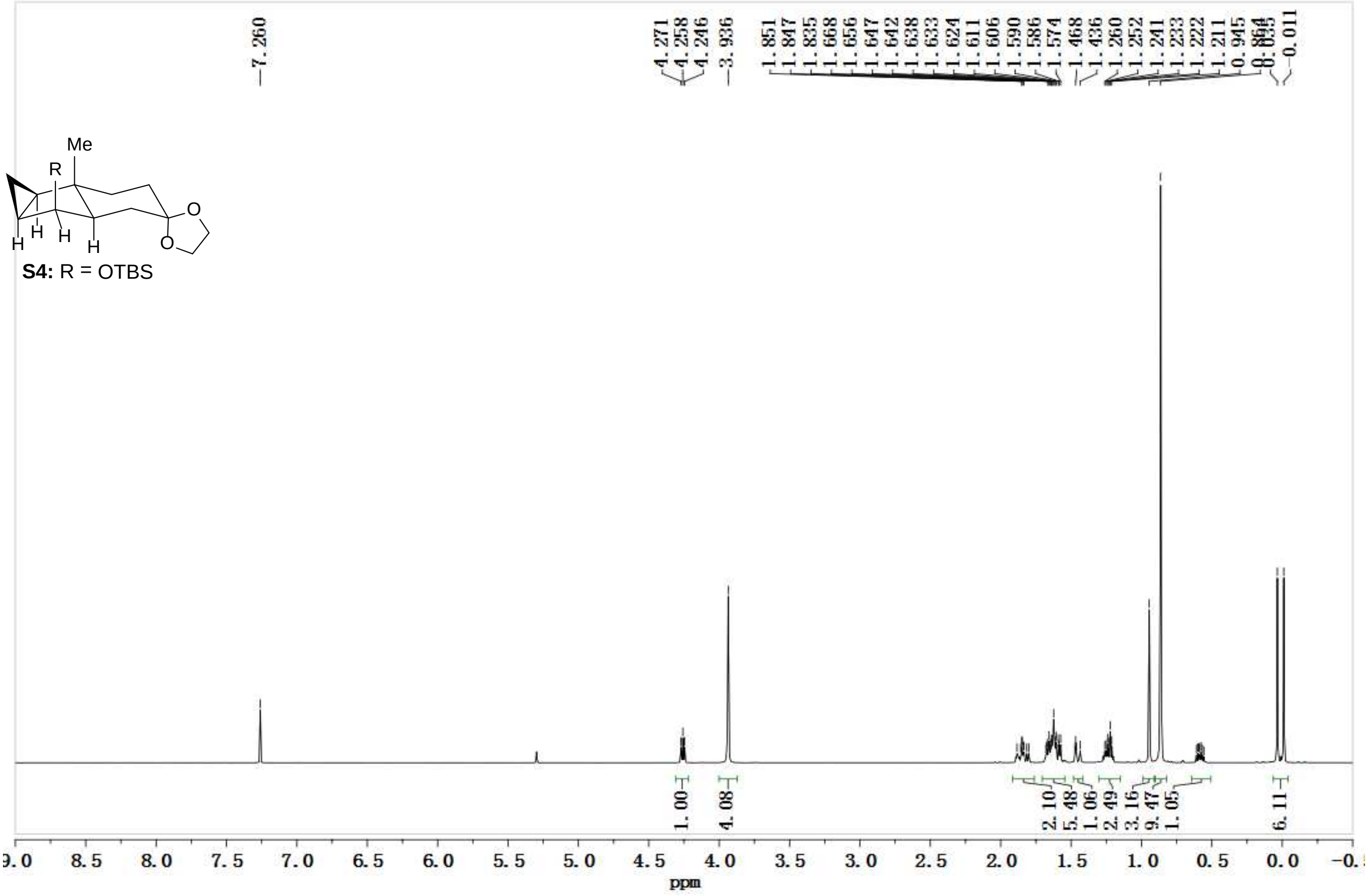

Supplementary Figure 7 ^{1}H NMR spectrum of Compound **S4** in $CDCl_3$

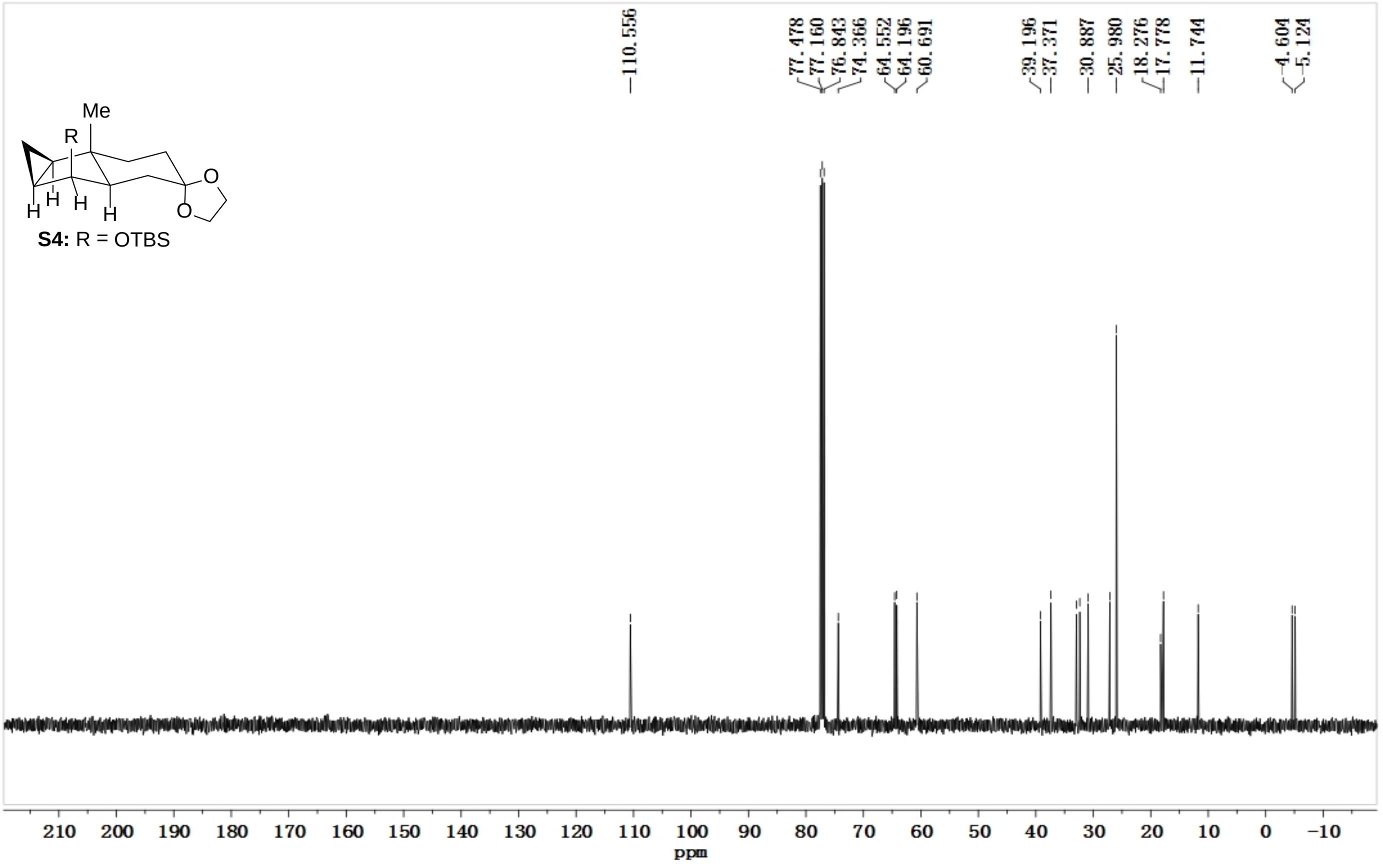

Supplementary Figure 8 ^{13}C NMR spectrum of Compound **S4** in $CDCl_3$

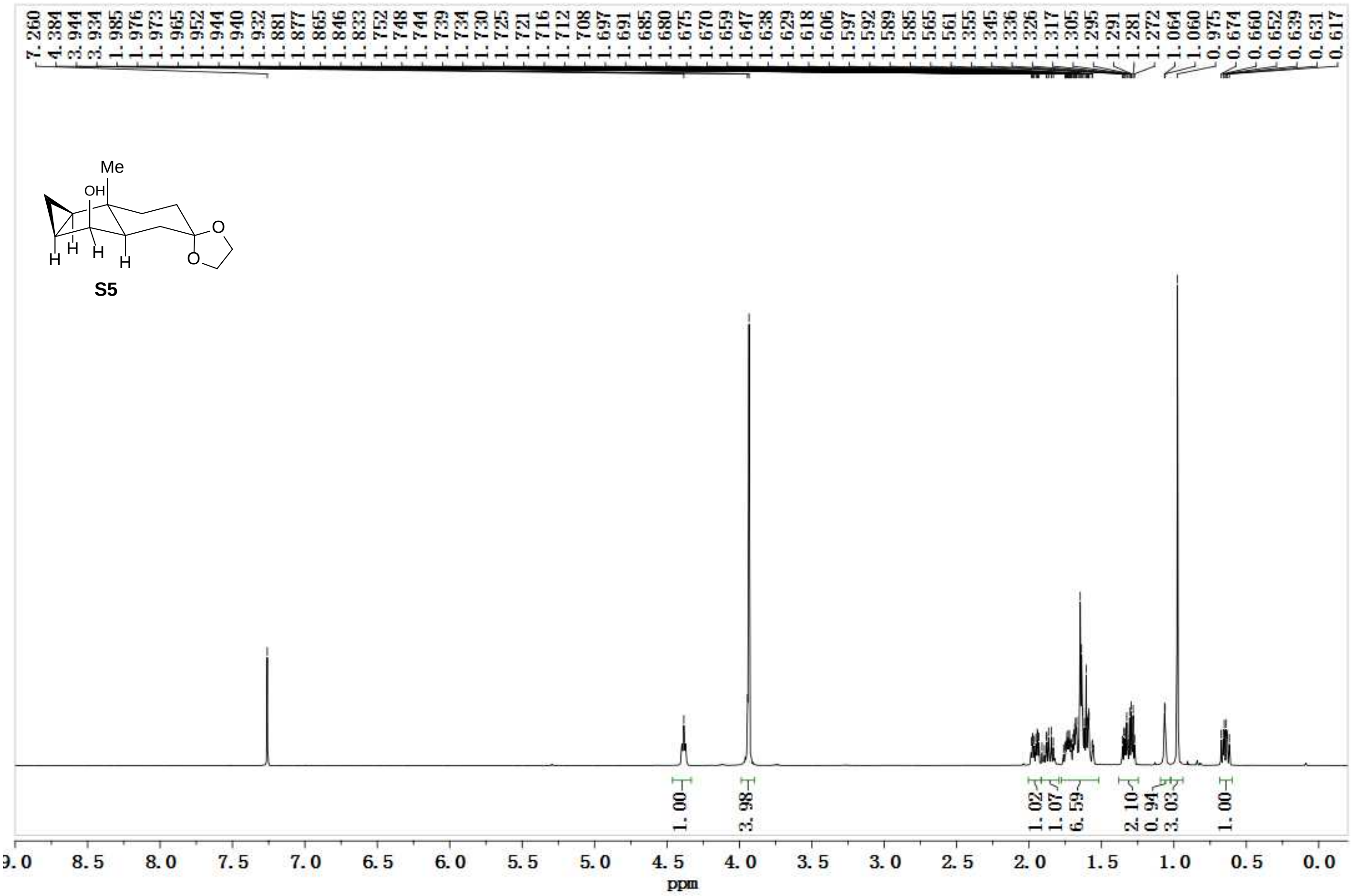

Supplementary Figure 9 ^{1}H NMR spectrum of Compound **S5** in $CDCl_3$

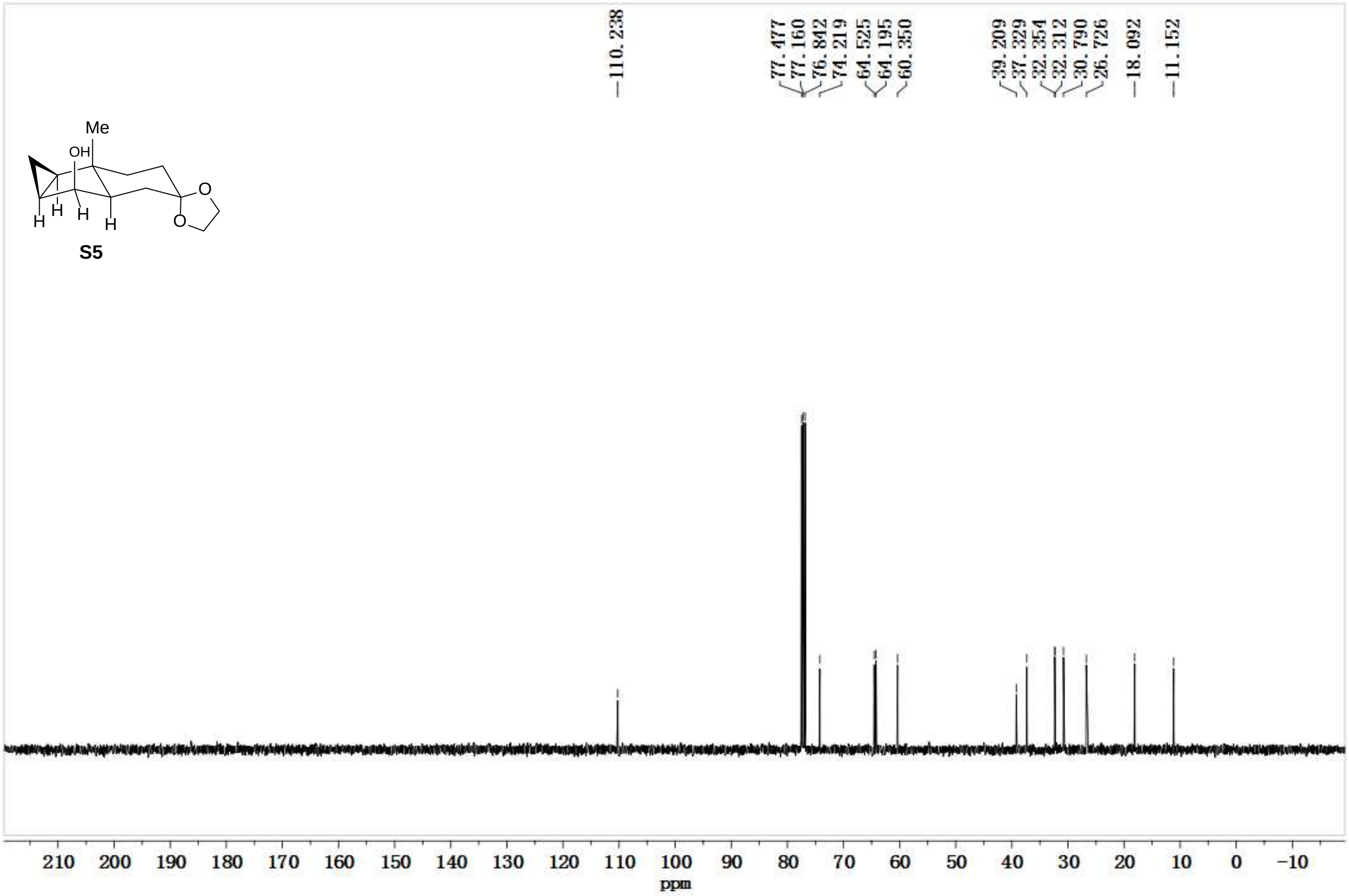

Supplementary Figure 10 ^{13}C NMR spectrum of Compound **S5** in $CDCl_3$

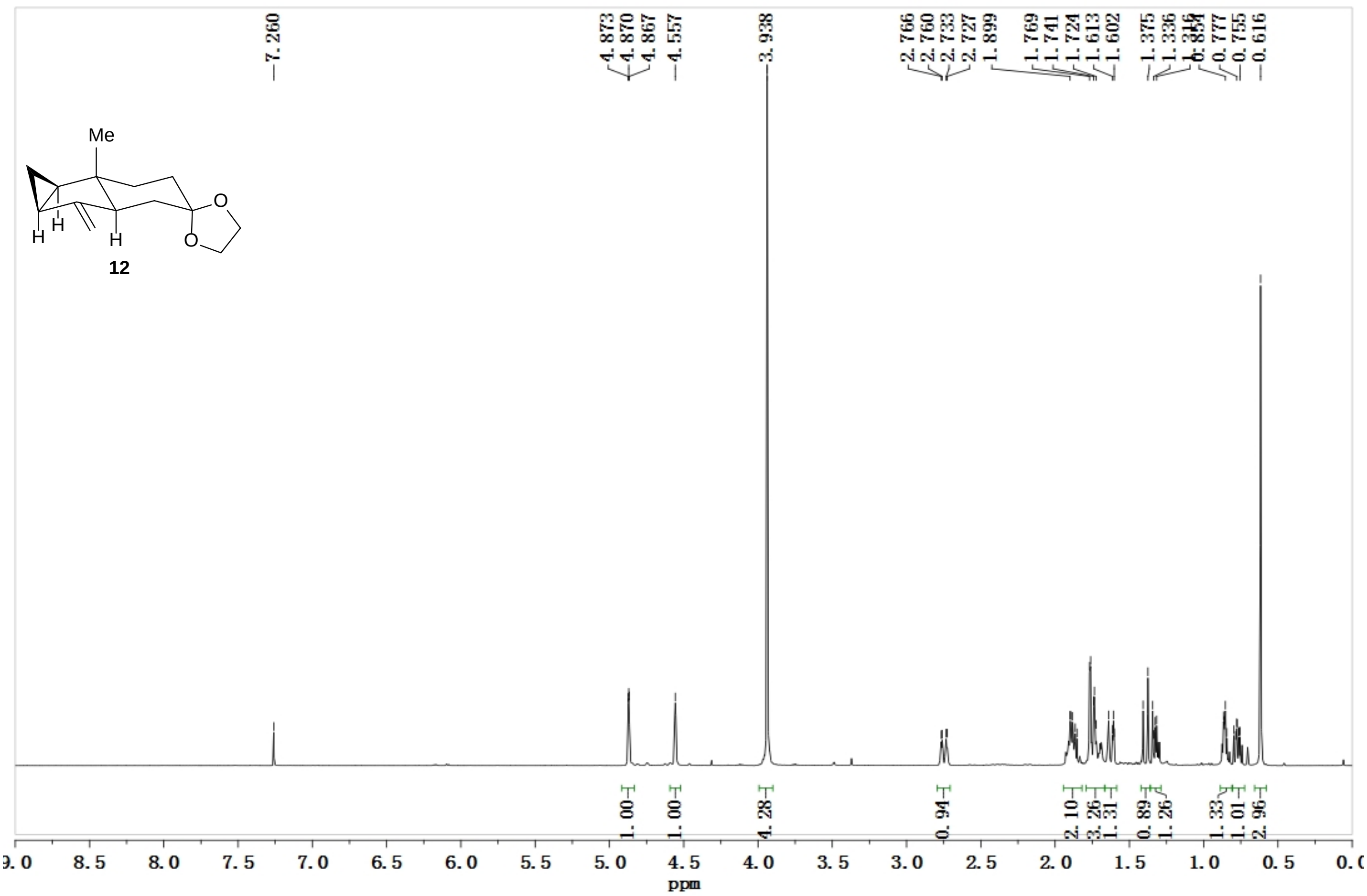

Supplementary Figure 11 ^{1}H NMR spectrum of Compound **12** in $CDCl_3$

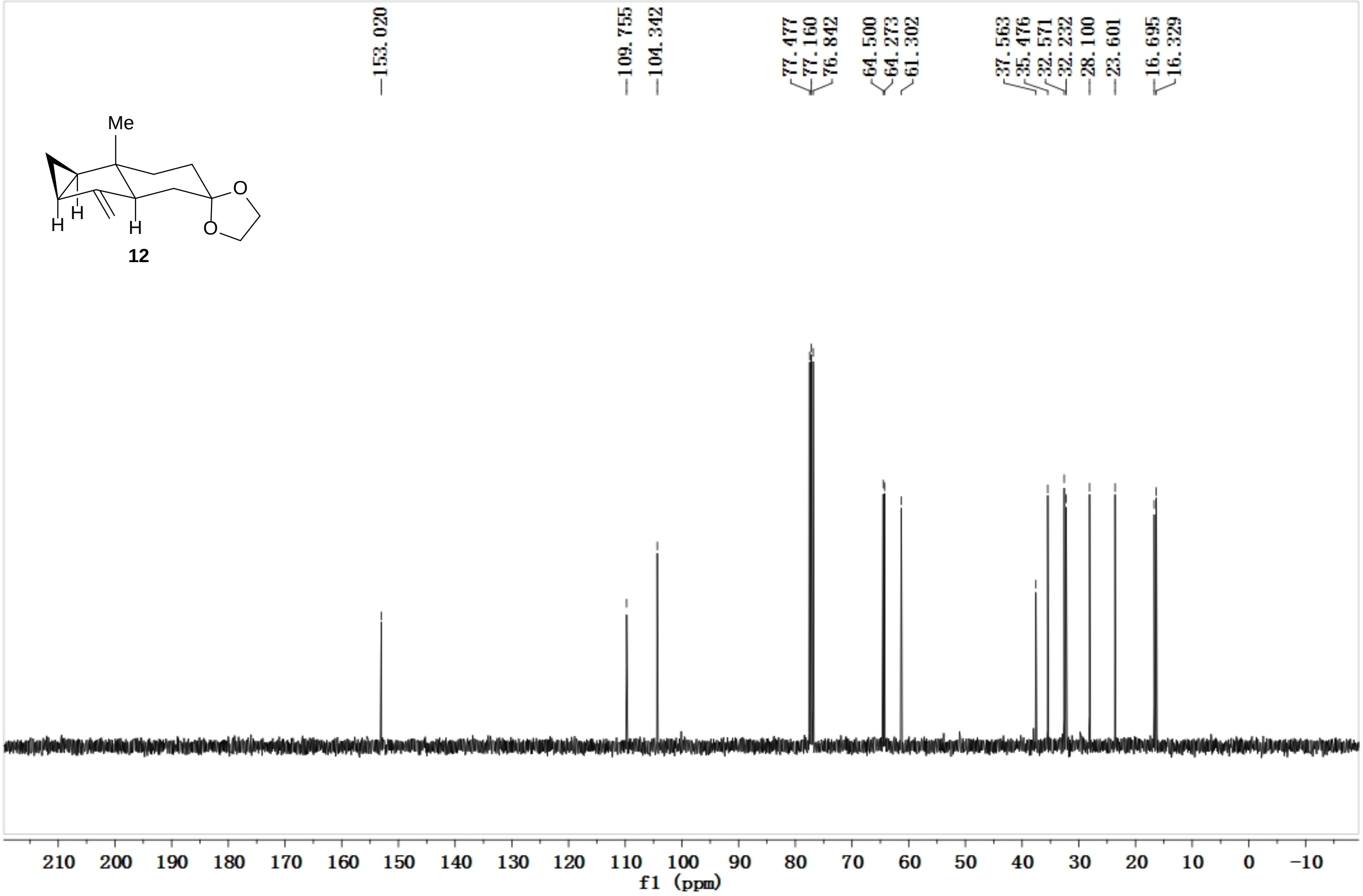

Supplementary Figure 12 ^{13}C NMR spectrum of Compound **12** in $CDCl_3$

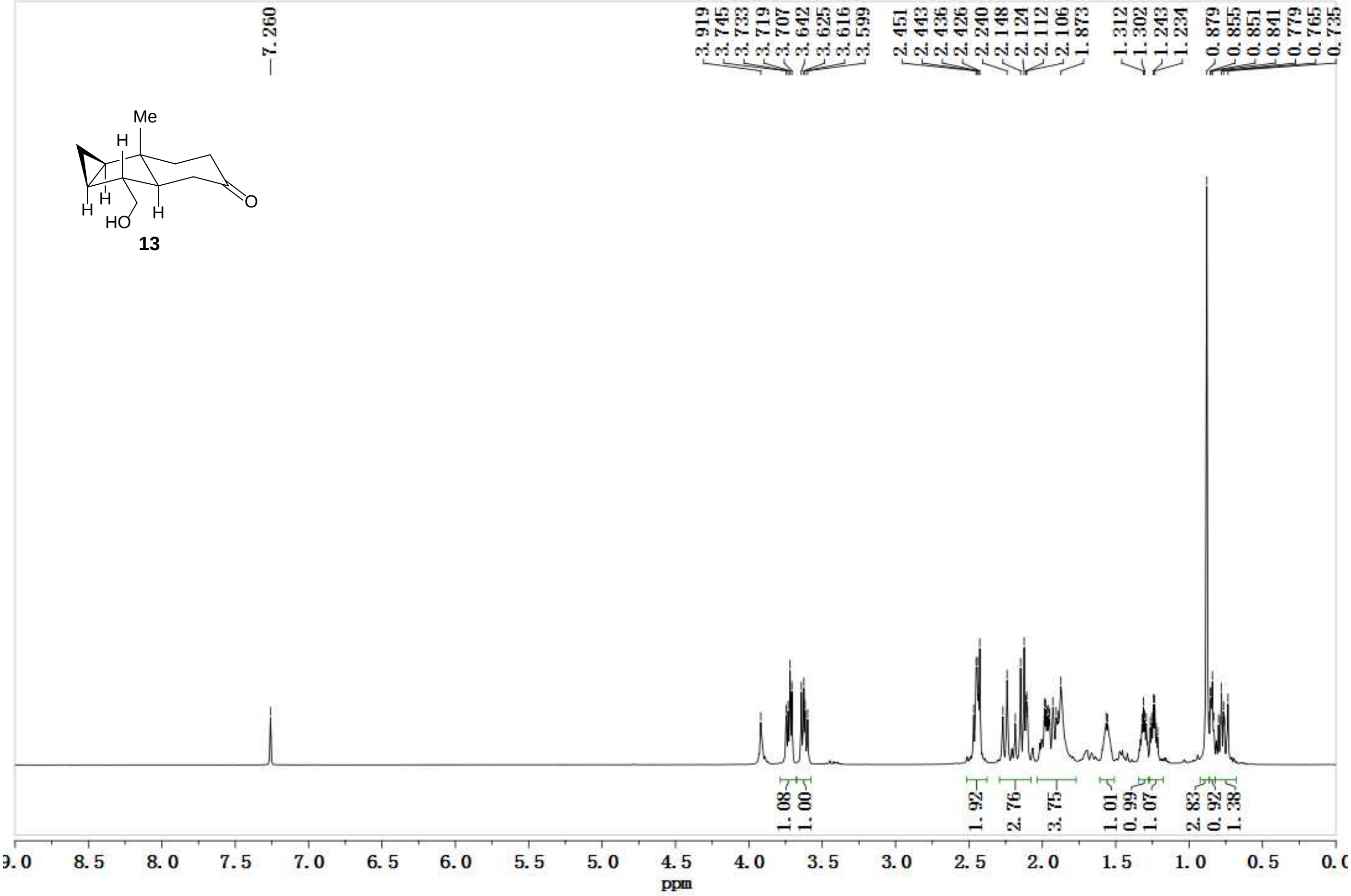

Supplementary Figure 13 ^{1}H NMR spectrum of Compound **13** in $CDCl_3$

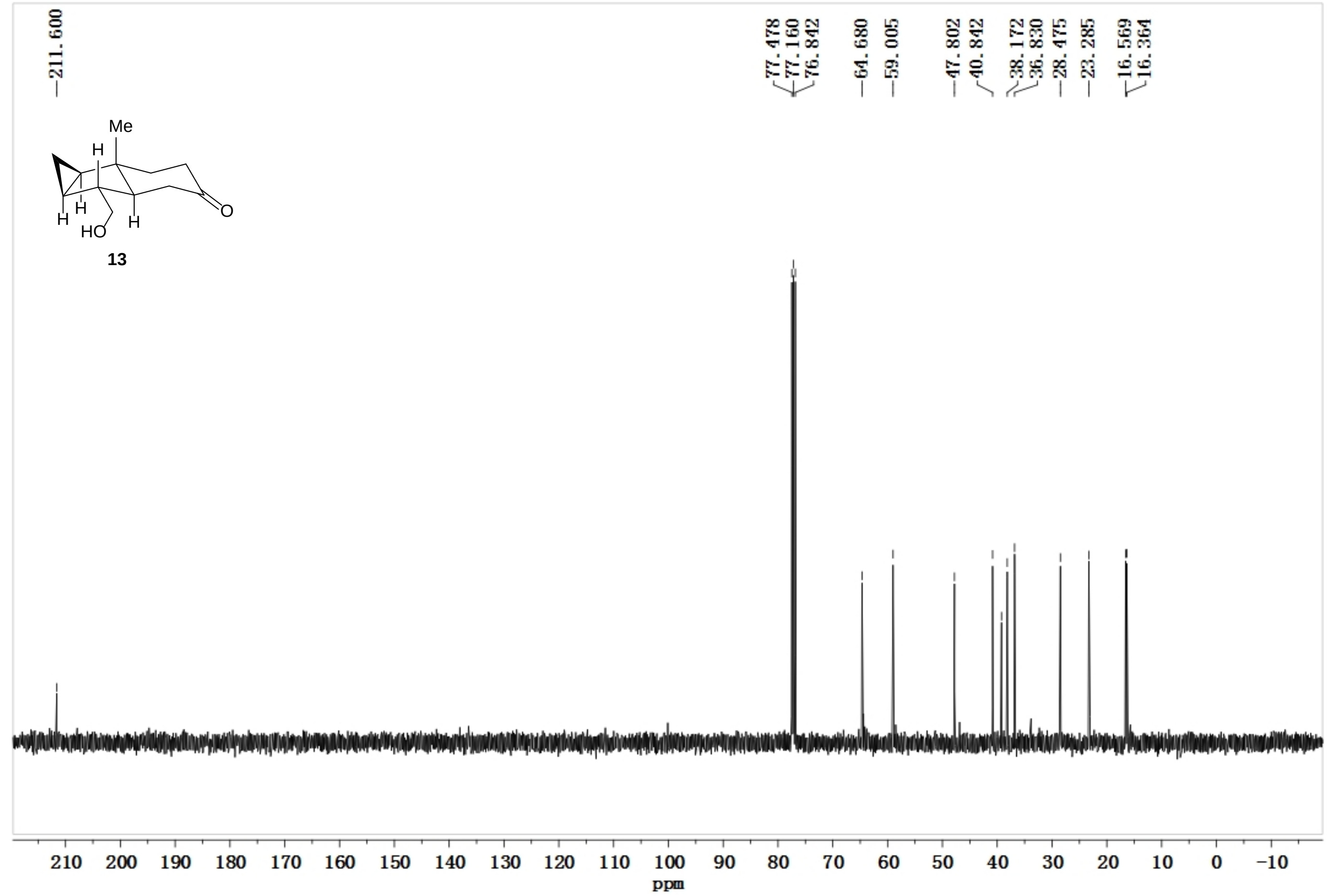

Supplementary Figure 14 ^{13}C NMR spectrum of Compound **13** in $CDCl_3$

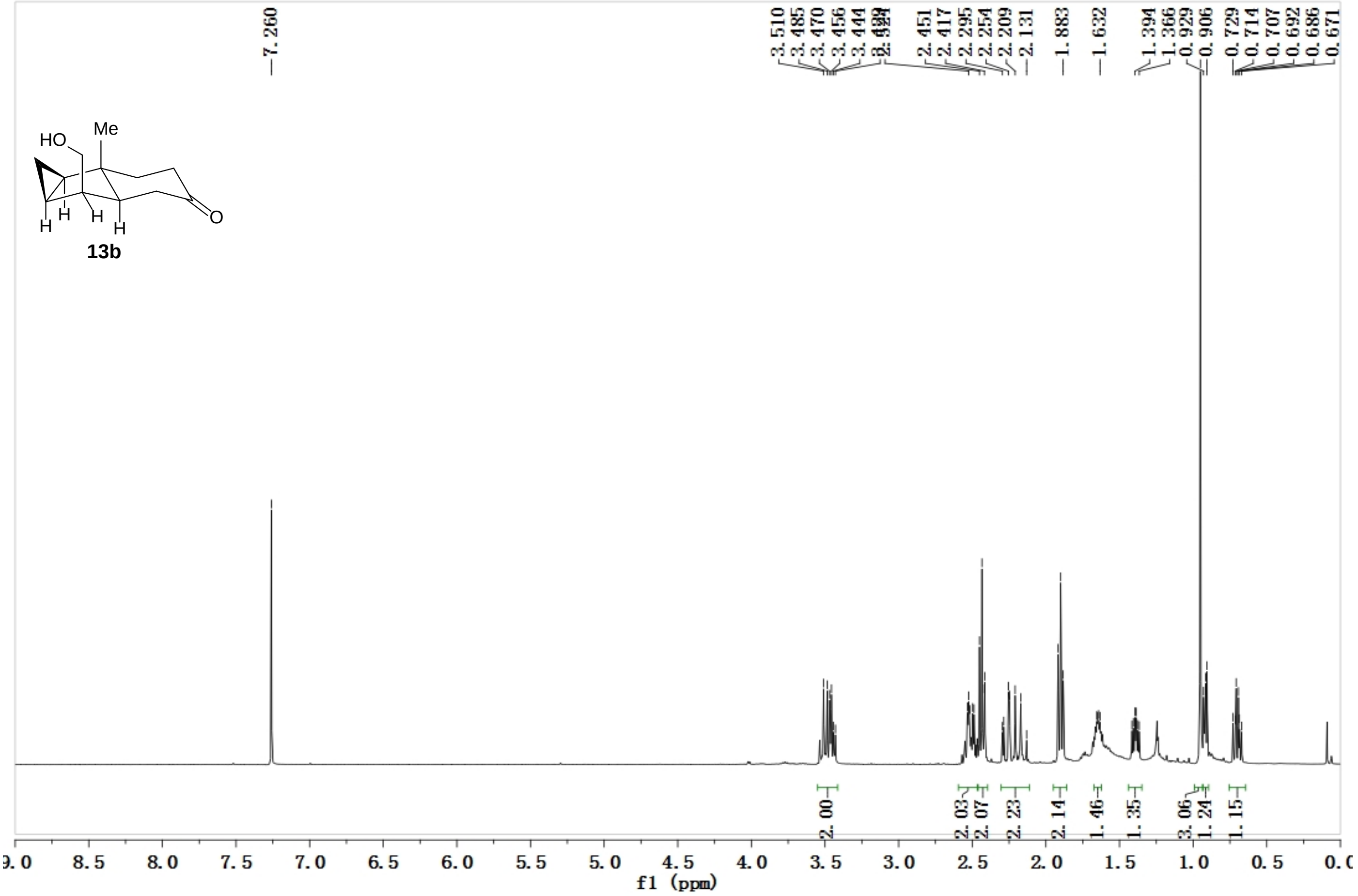

Supplementary Figure 15 ^{1}H NMR spectrum of Compound **13b** in $CDCl_3$

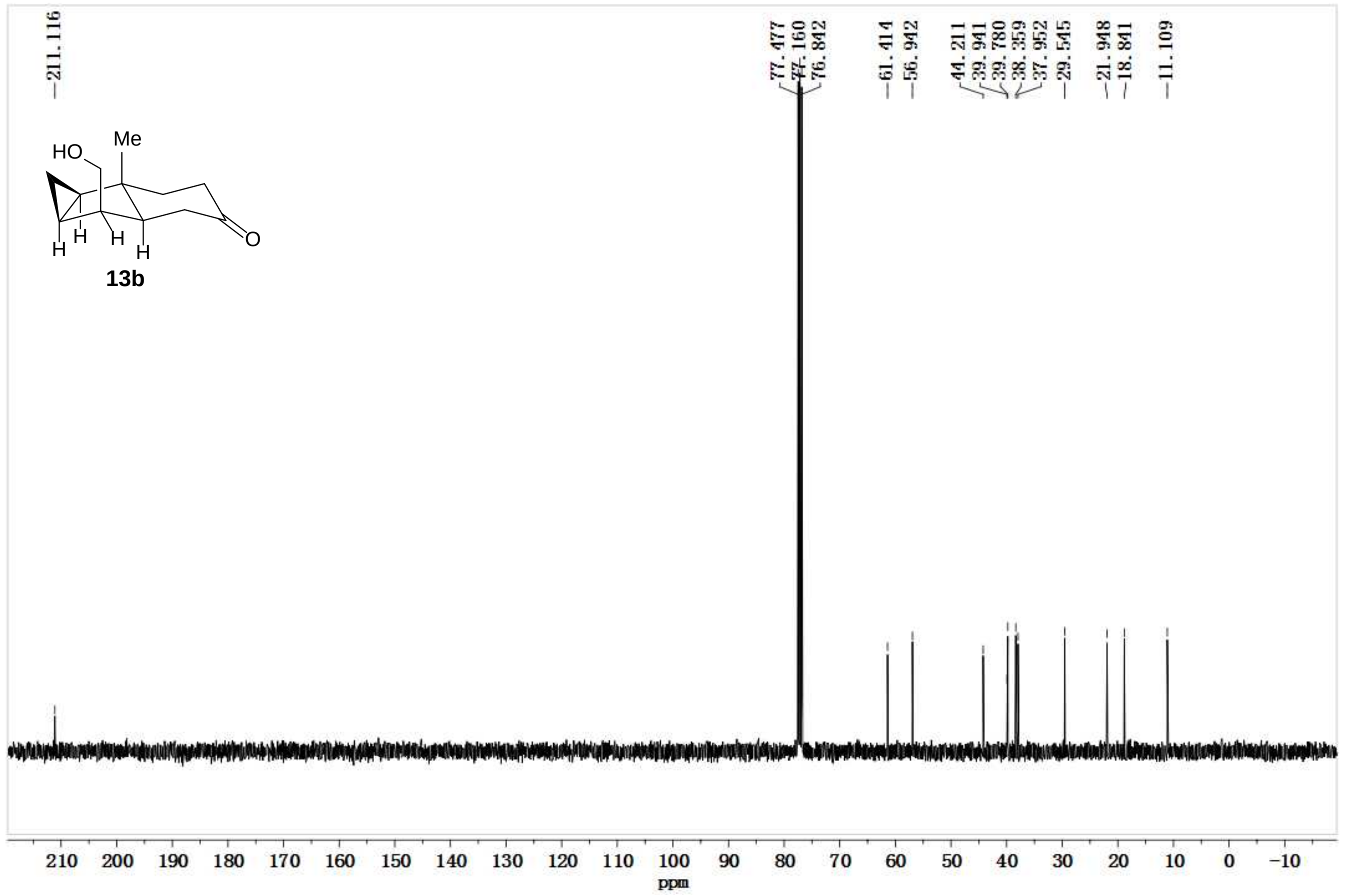

Supplementary Figure 16 ^{13}C NMR spectrum of Compound **13b** in $CDCl_3$

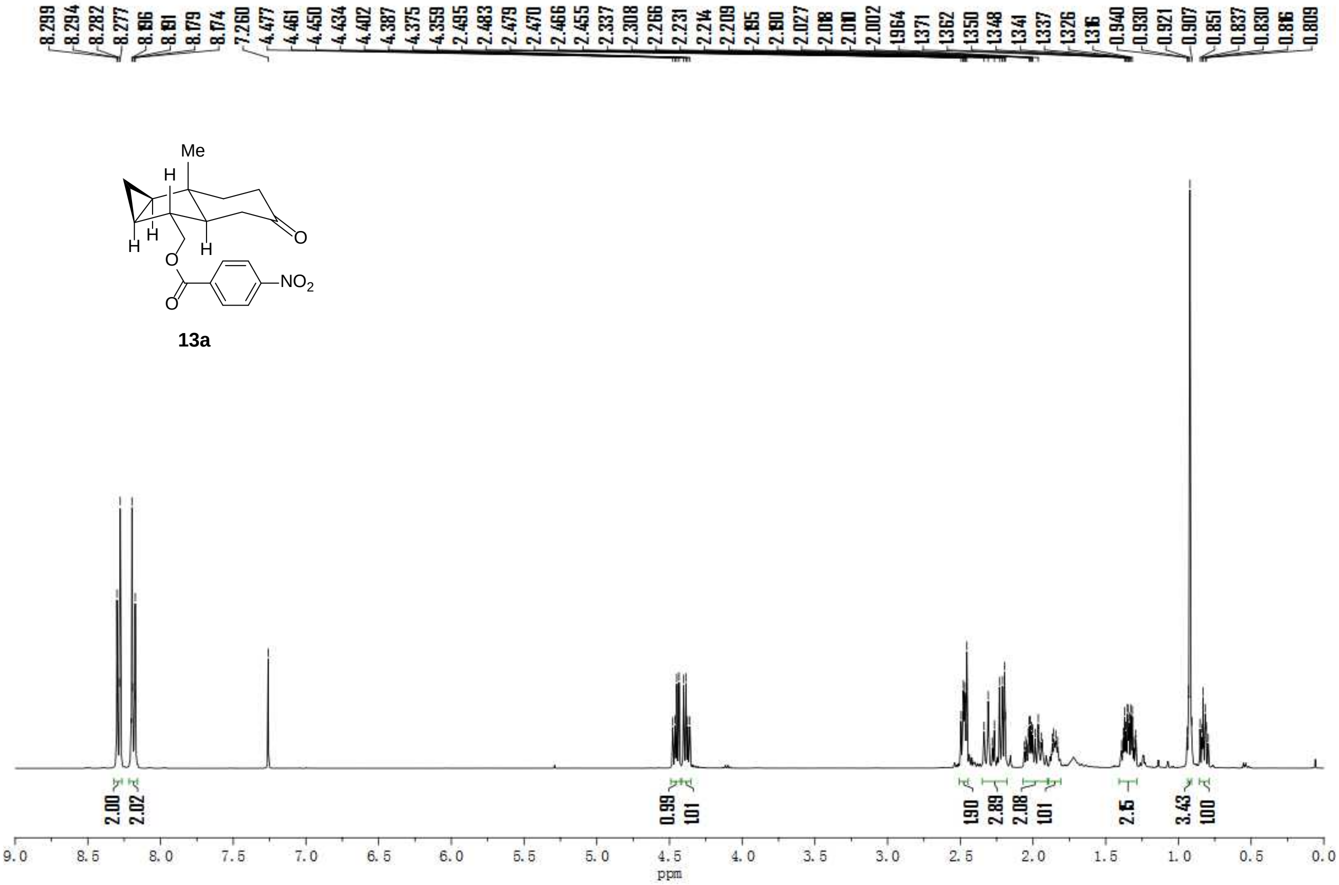

Supplementary Figure 17 ^{1}H NMR spectrum of Compound **13a** in $CDCl_3$

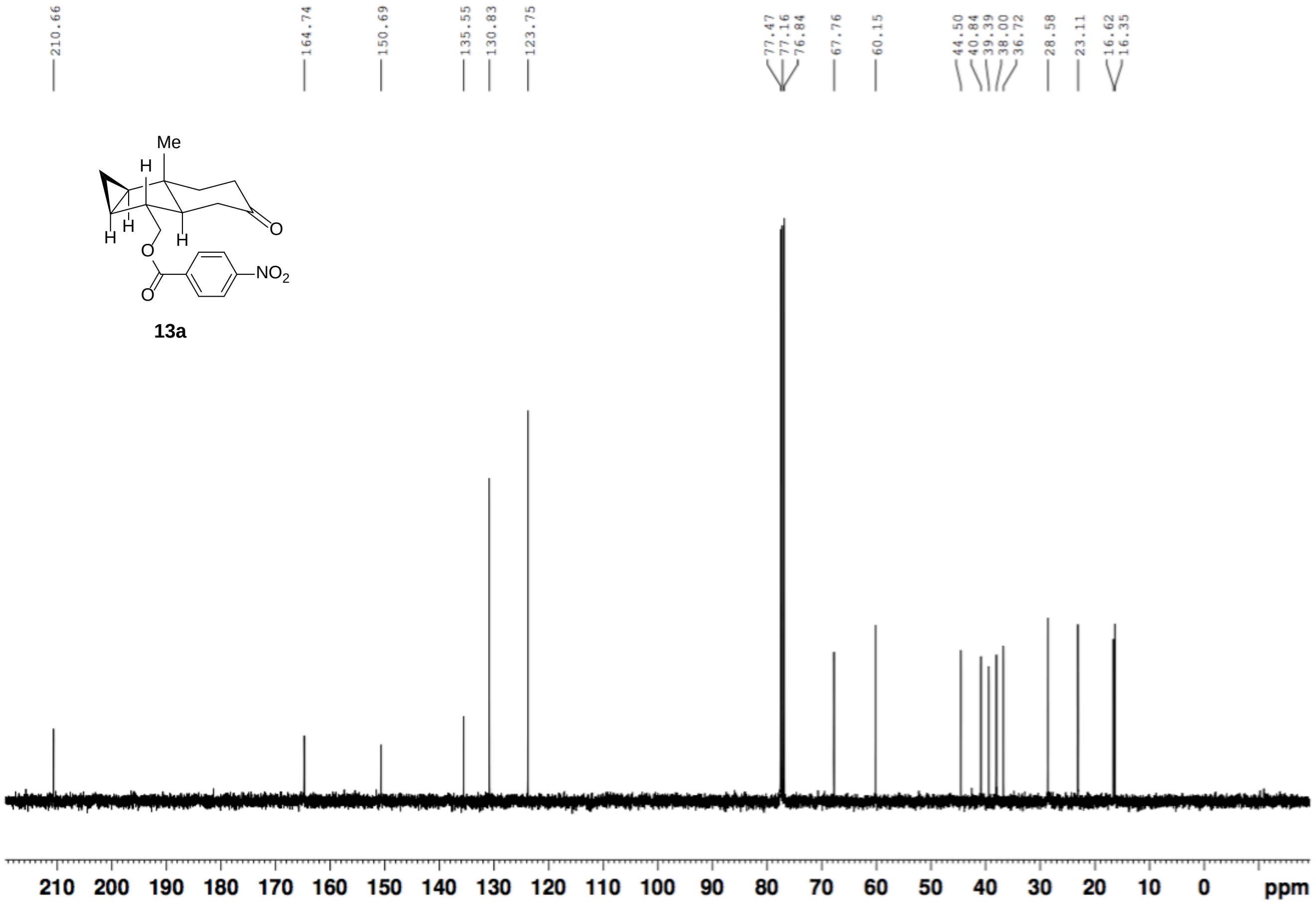

Supplementary Figure 18 ^{13}C NMR spectrum of Compound **13a** in $CDCl_3$

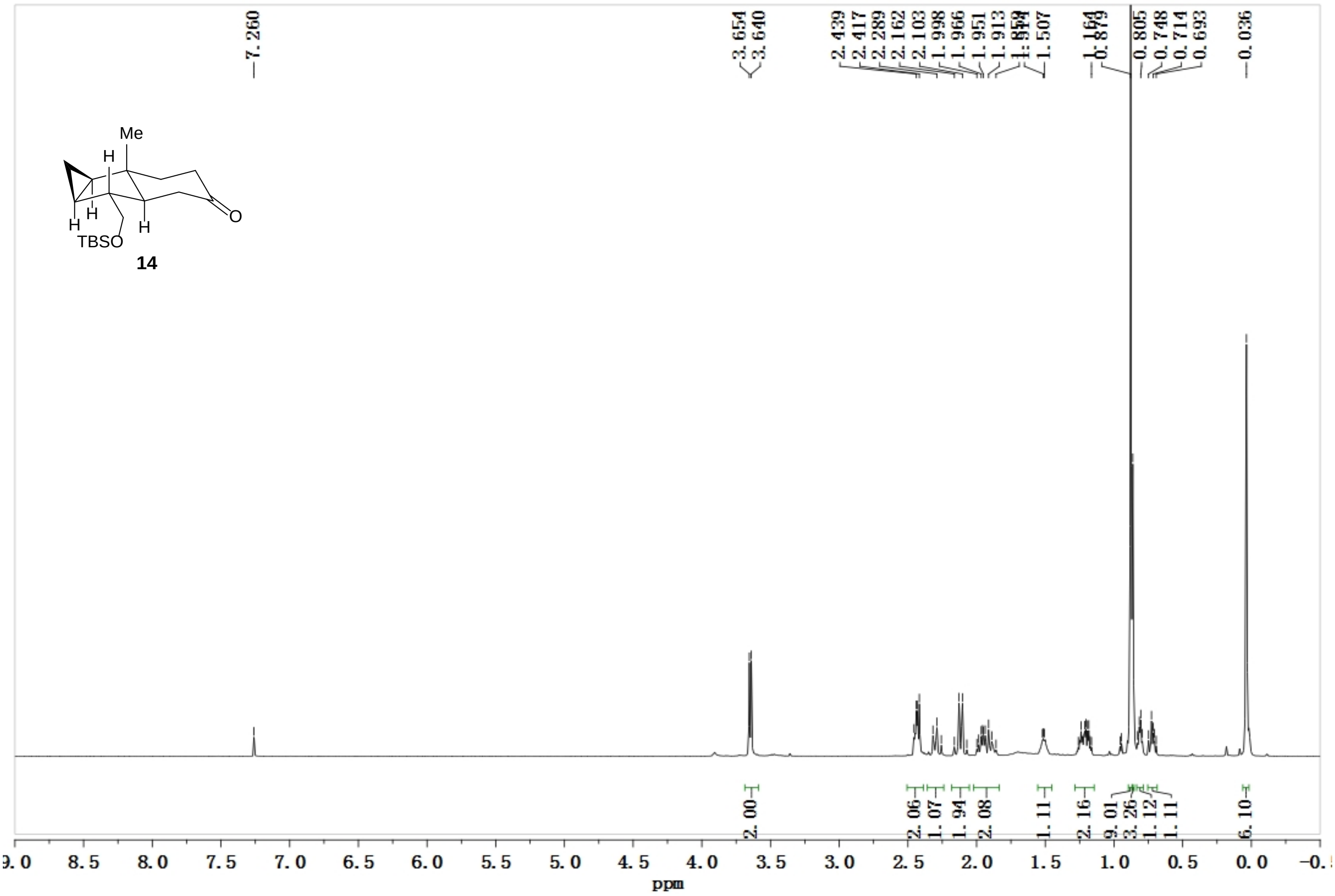

Supplementary Figure 19 ^{1}H NMR spectrum of Compound **14** in $CDCl_3$

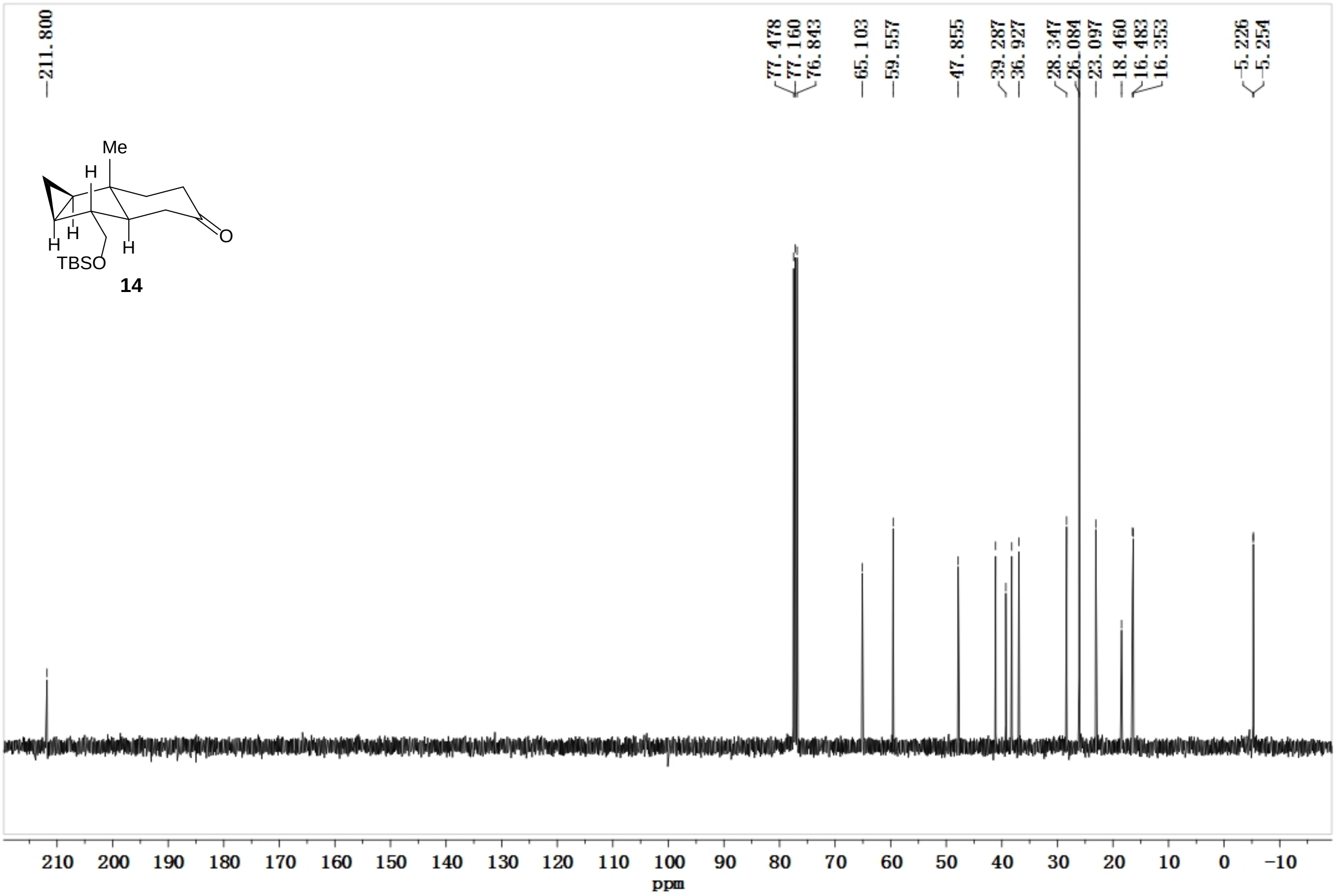

Supplementary Figure 20 ^{13}C NMR spectrum of Compound **14** in $CDCl_3$

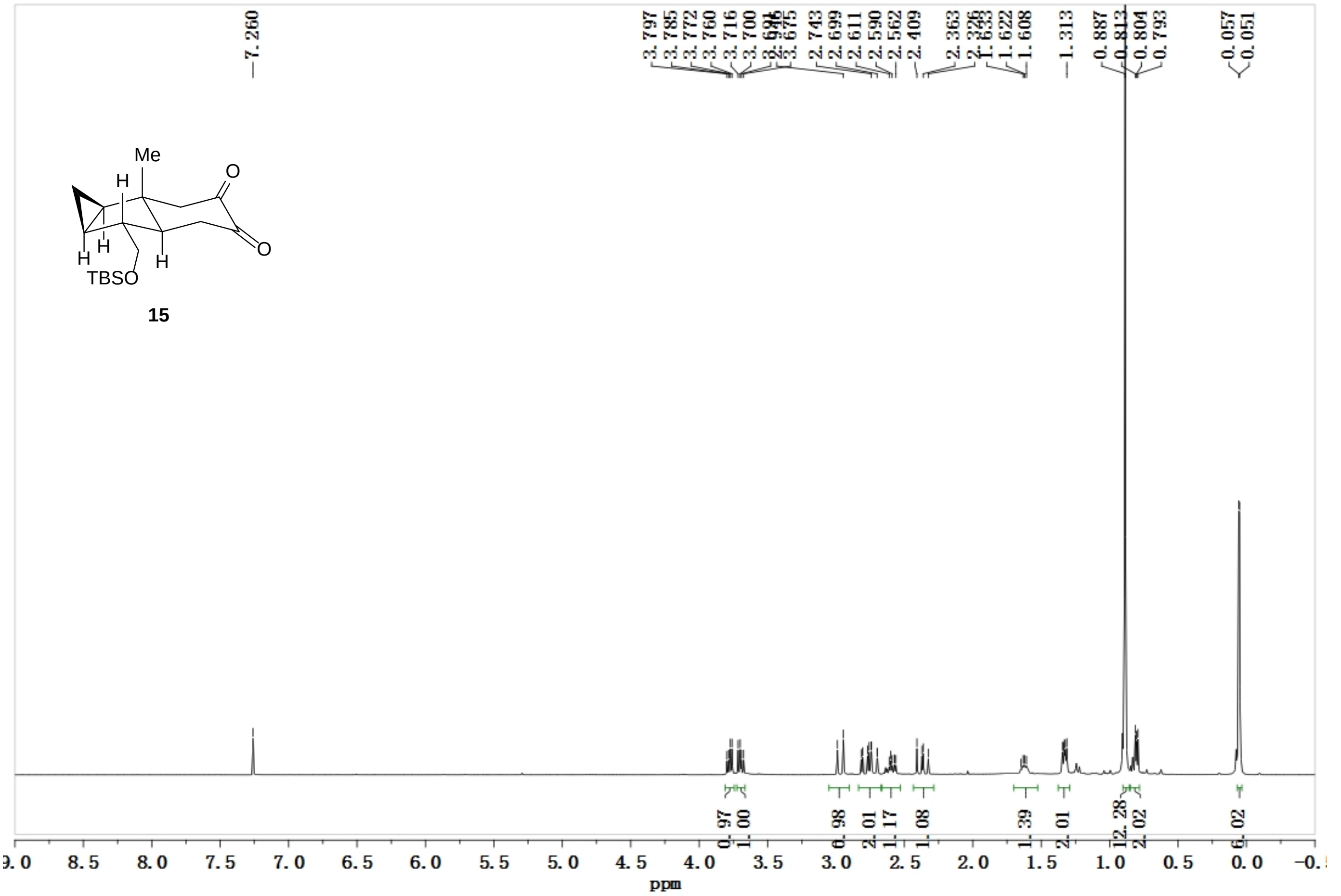

Supplementary Figure 21 ^{1}H NMR spectrum of Compound **15** in $CDCl_3$

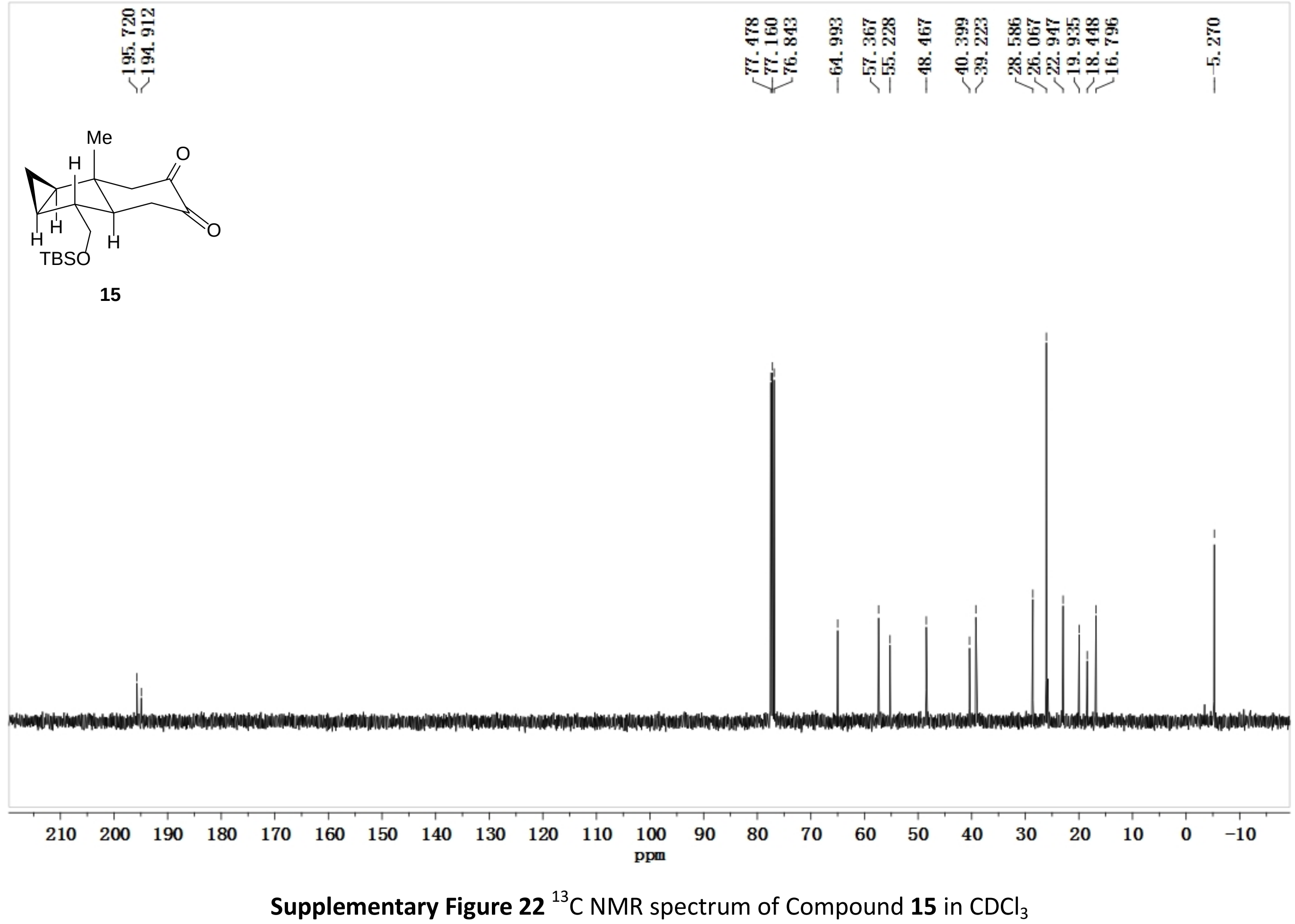

Supplementary Figure 22 ^{13}C NMR spectrum of Compound **15** in $CDCl_3$

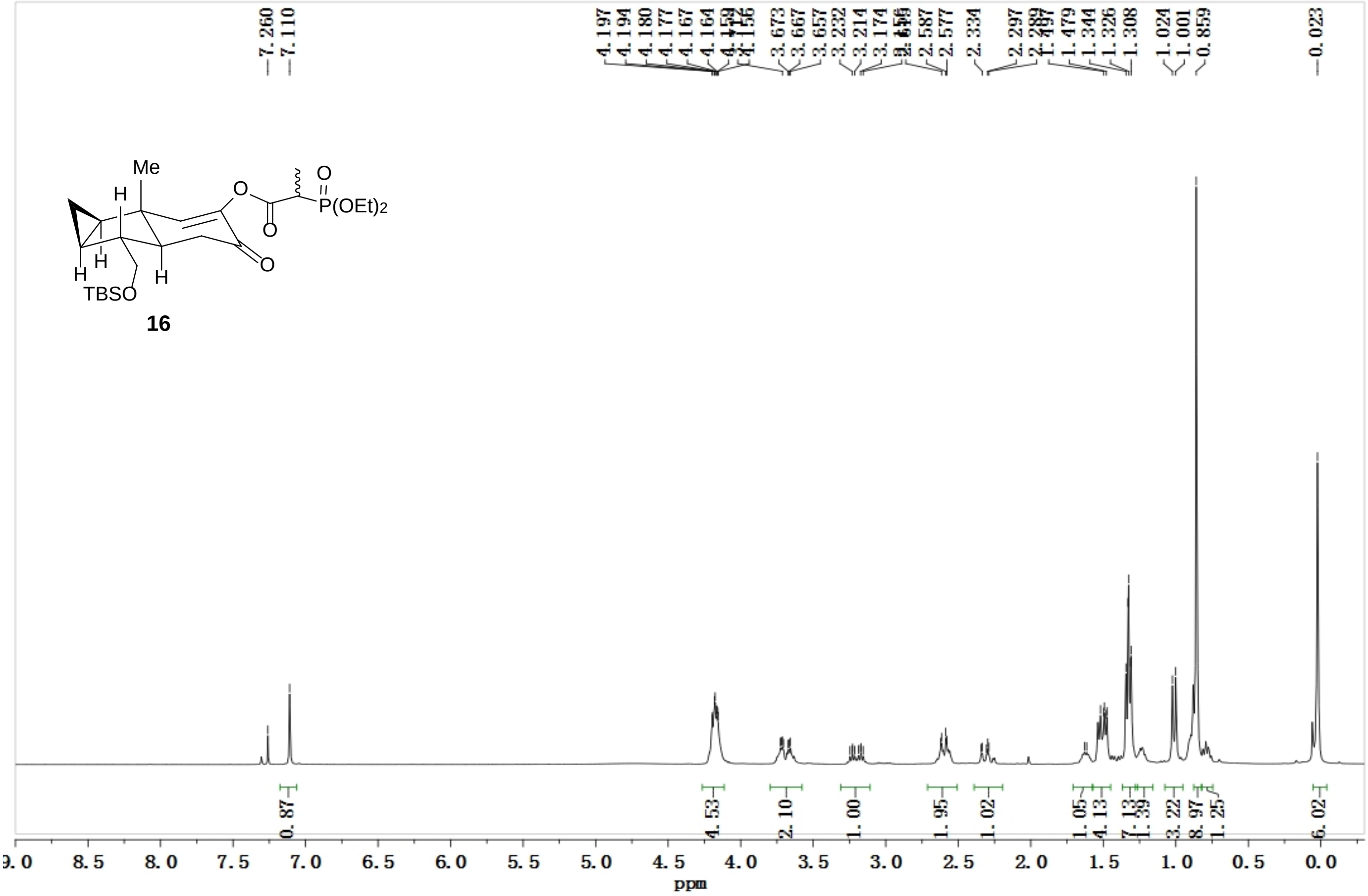

Supplementary Figure 23 ^{1}H NMR spectrum of Compound **16** in $CDCl_3$

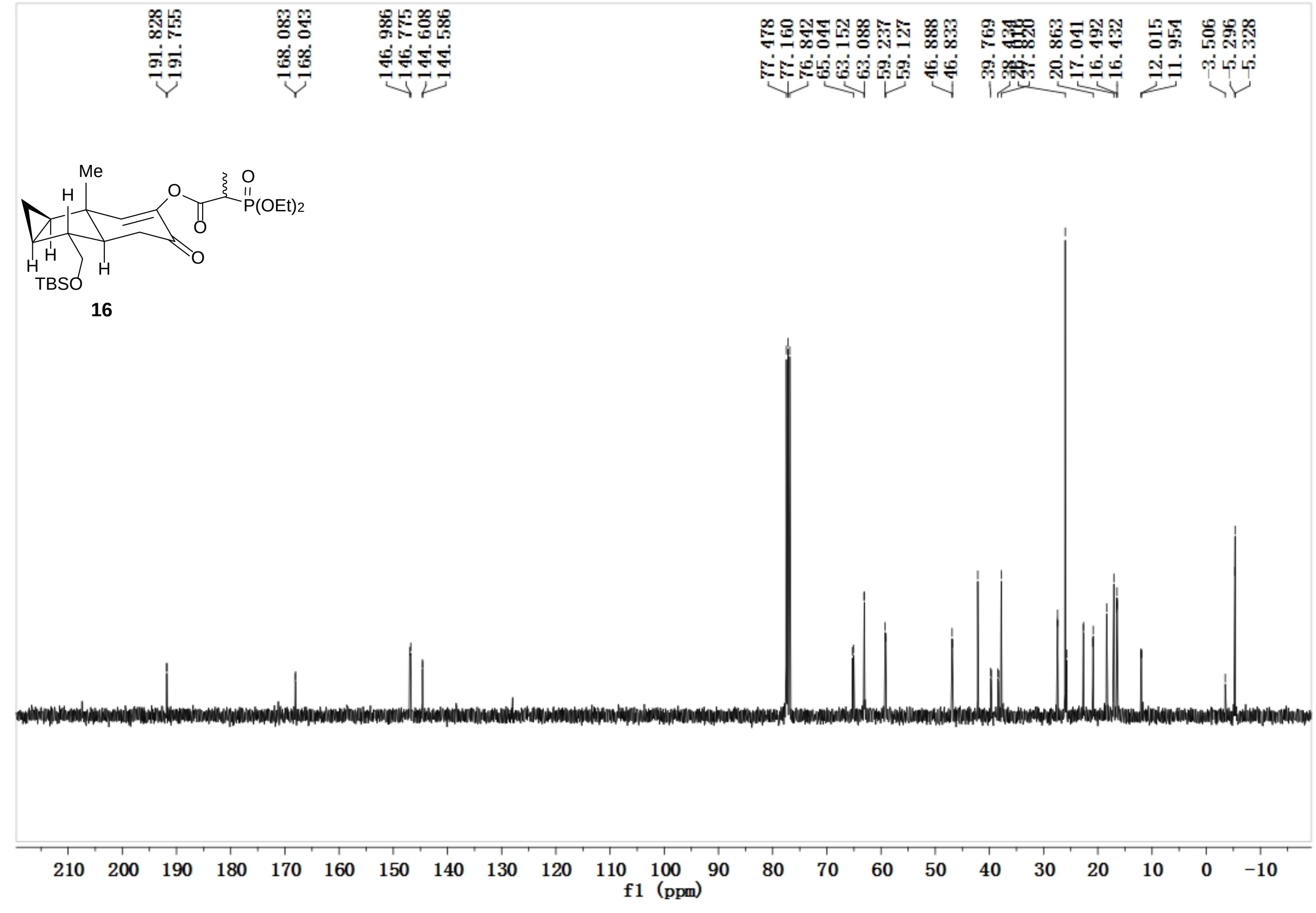

Supplementary Figure 24 ^{13}C NMR spectrum of Compound **16** in $CDCl_3$

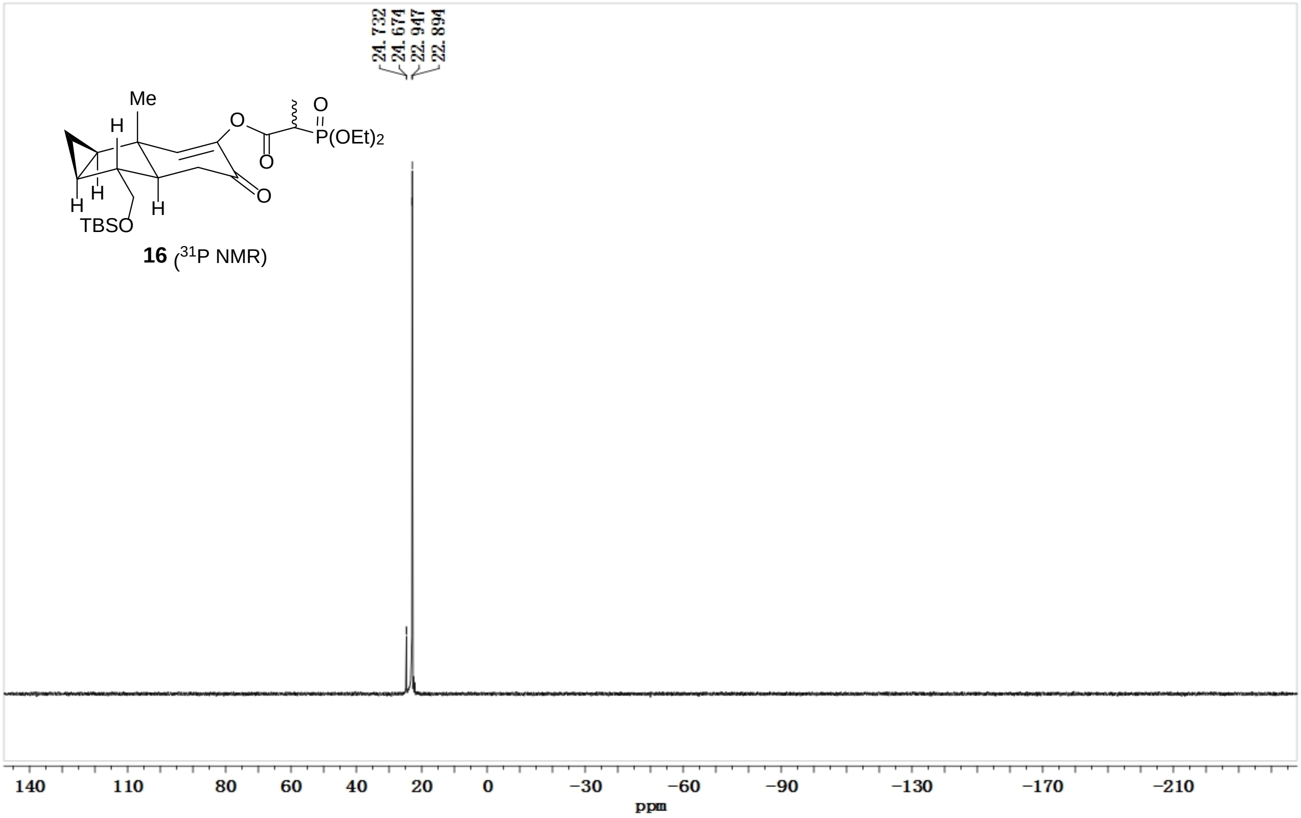

Supplementary Figure 25 ^{31}P NMR spectrum of Compound **16** in $CDCl_3$

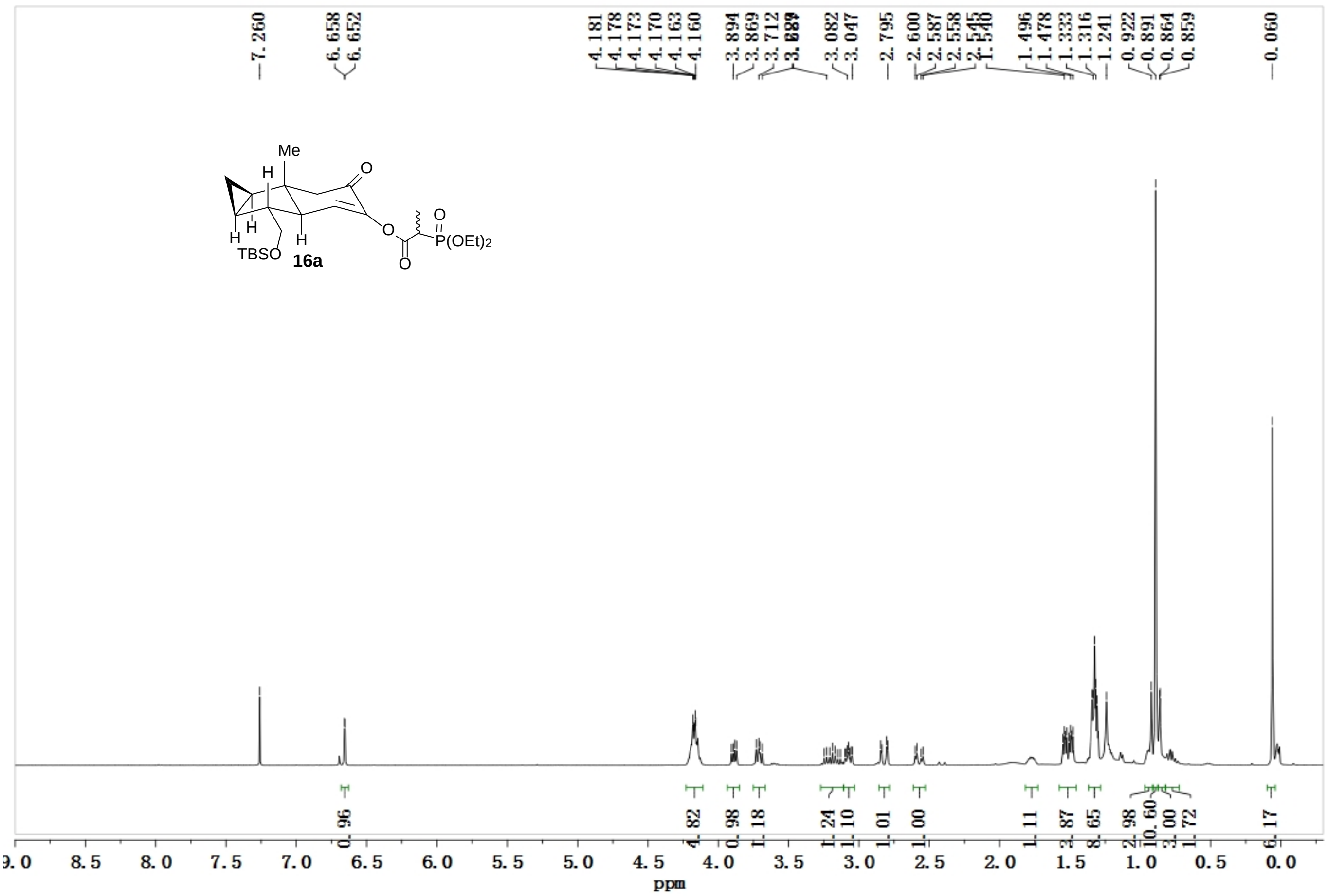

Supplementary Figure 26 ^{1}H NMR spectrum of Compound **16a** in $CDCl_3$

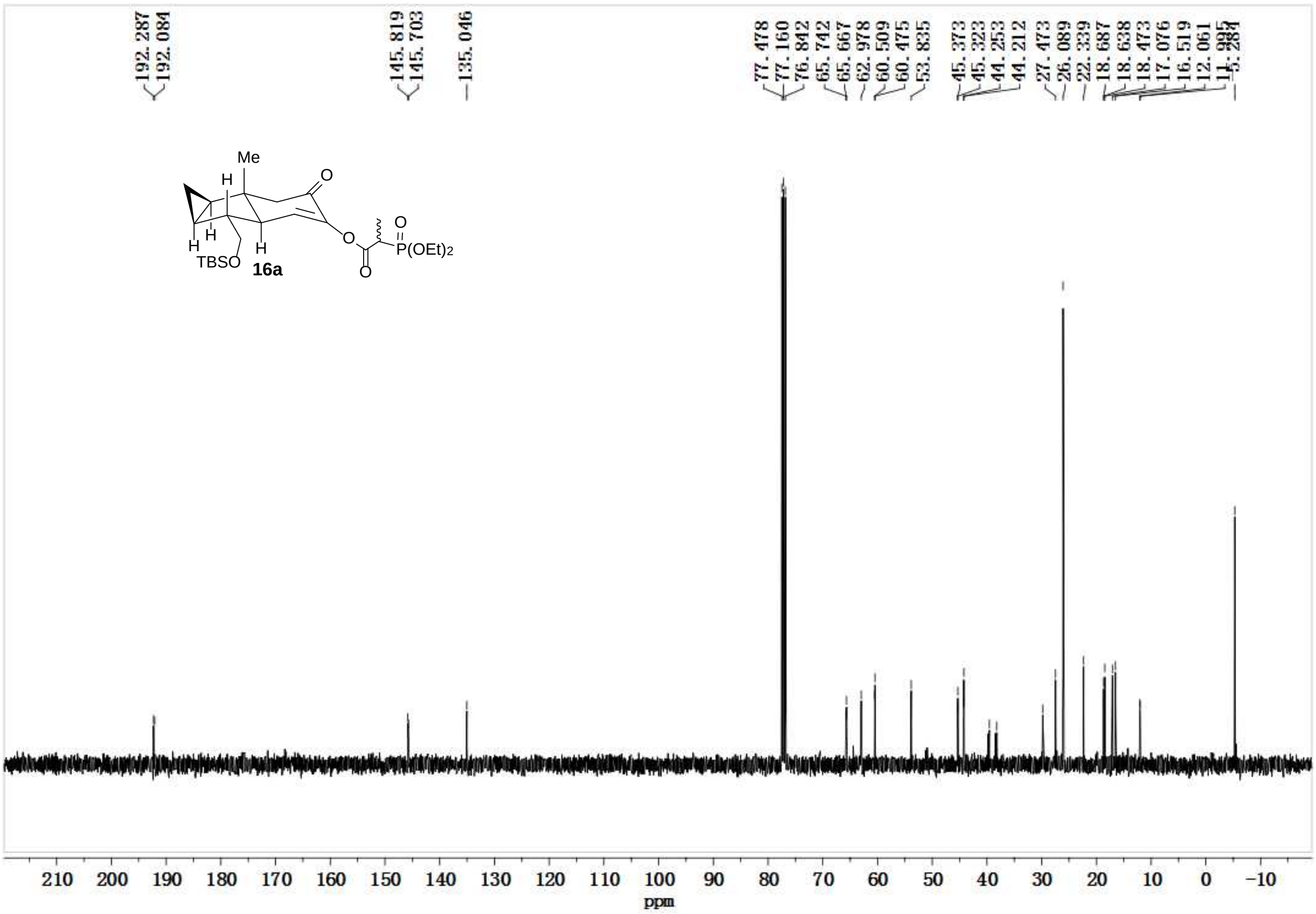

Supplementary Figure 27 ^{13}C NMR spectrum of Compound **16a** in $CDCl_3$

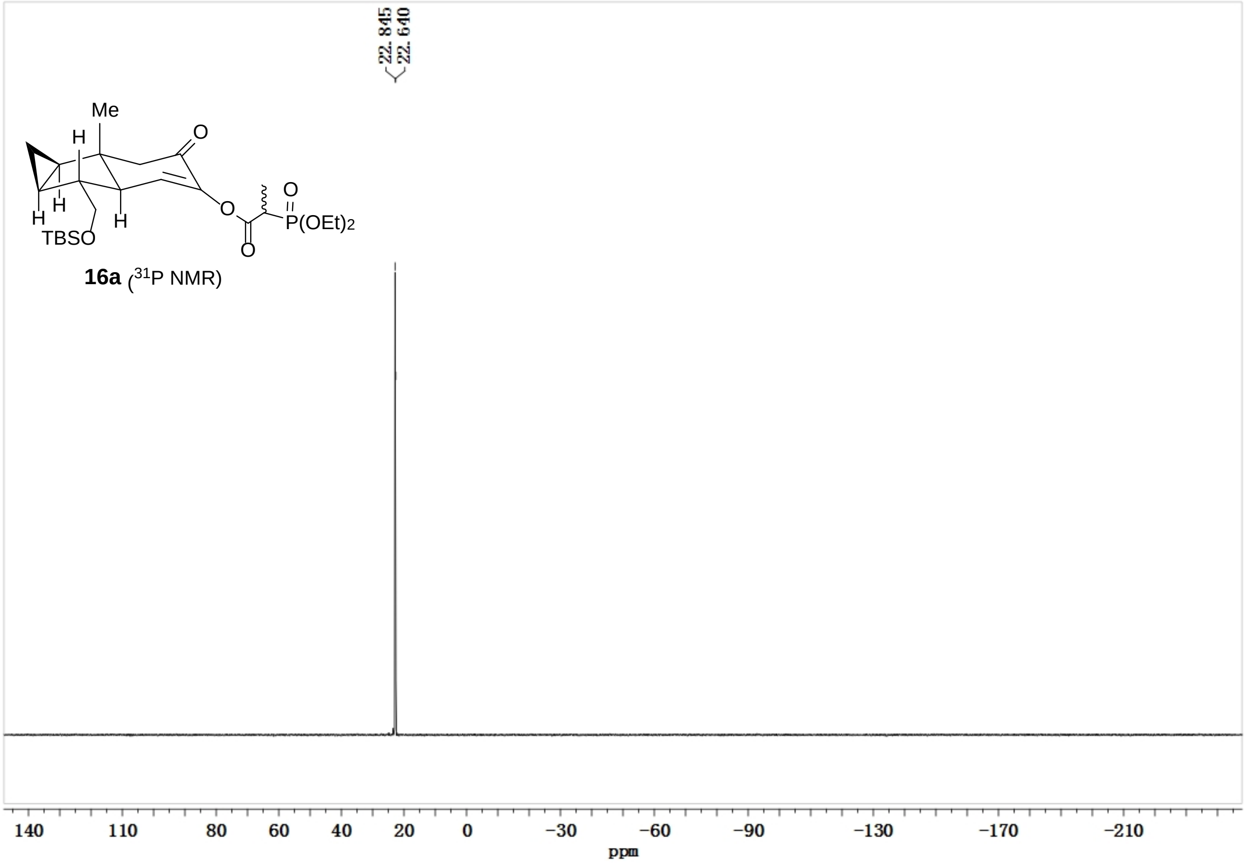

Supplementary Figure 28 ^{31}P NMR spectrum of Compound **16a** in $CDCl_3$

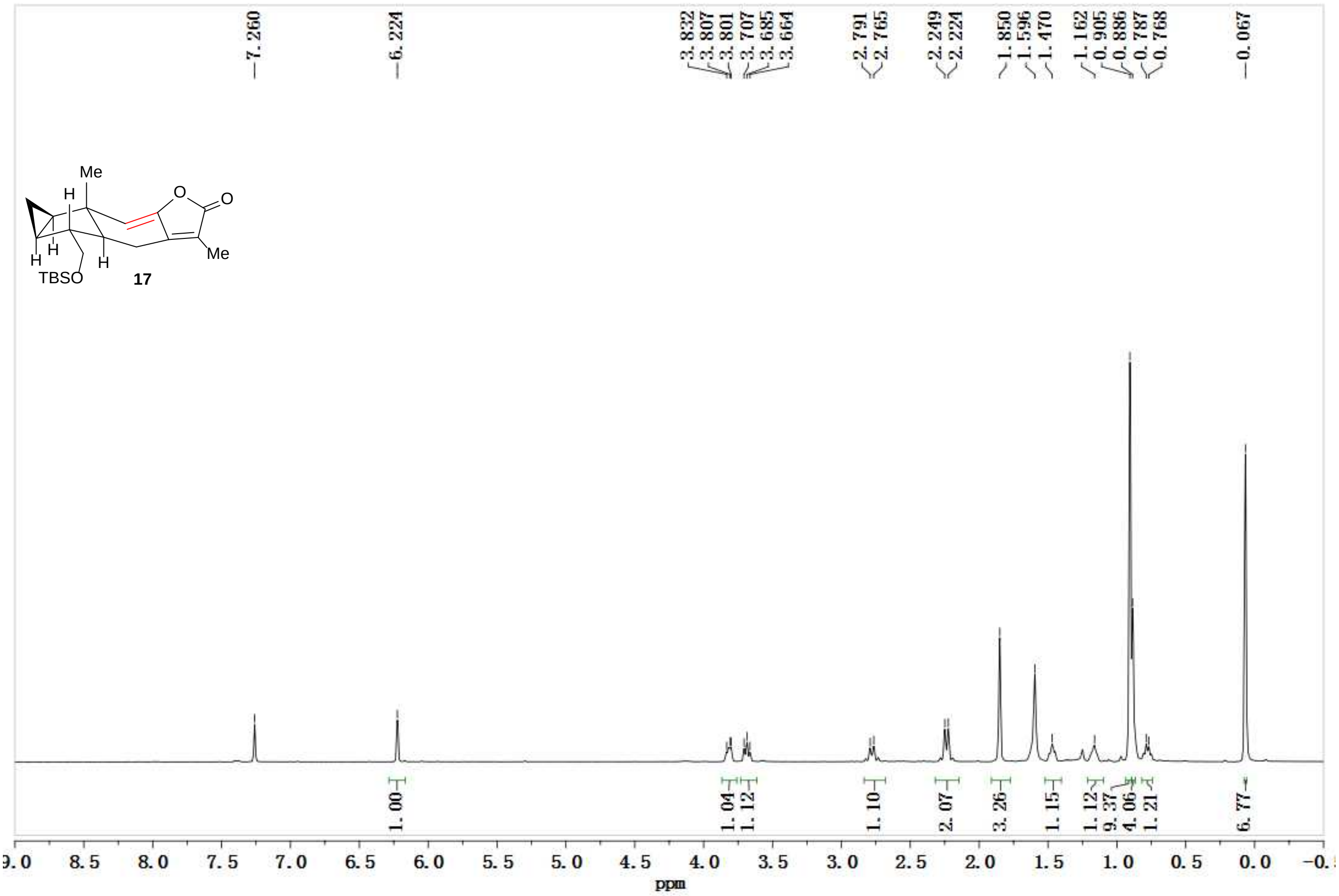

Supplementary Figure 29 ^{1}H NMR spectrum of Compound **17** in $CDCl_3$

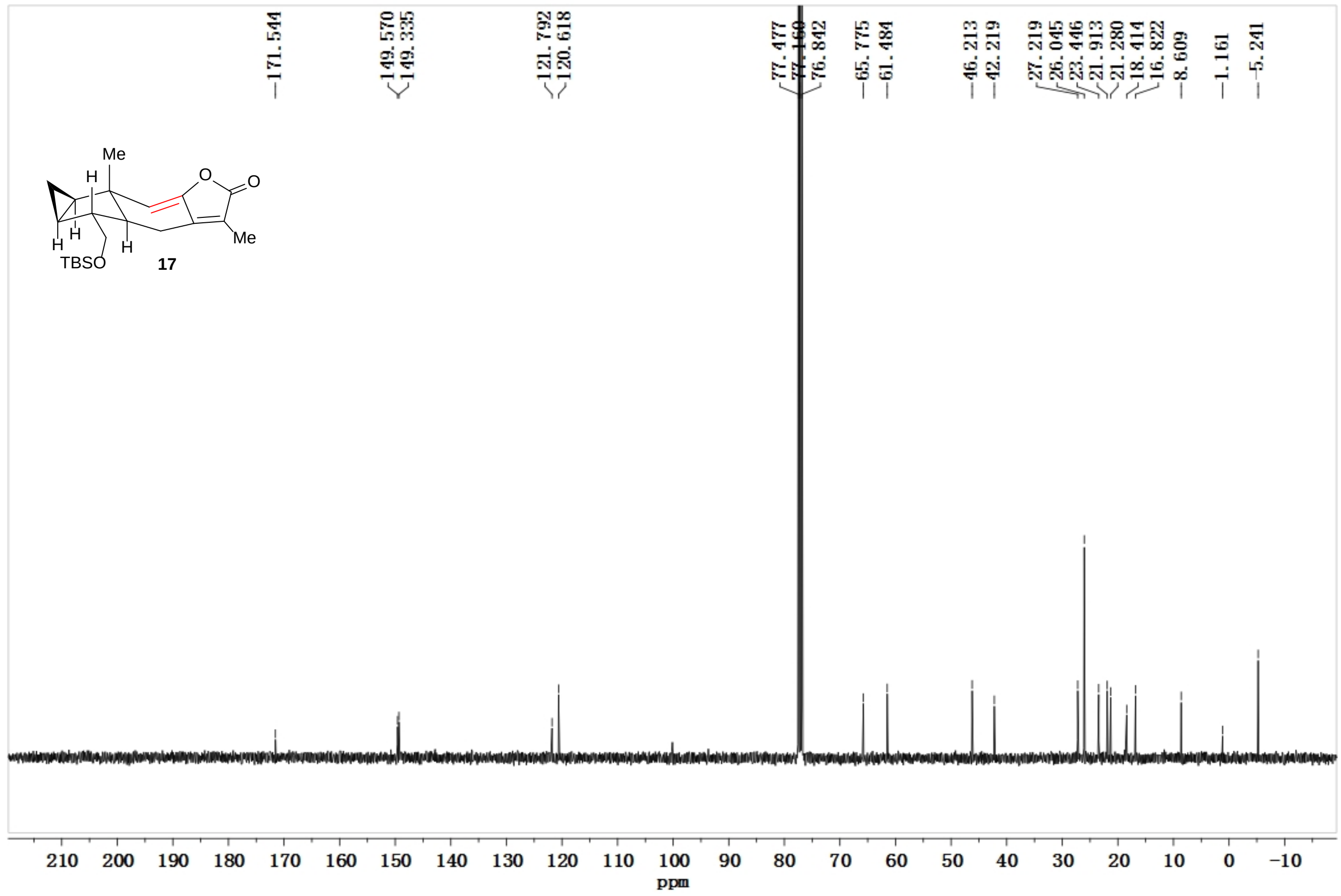

Supplementary Figure 30 ^{13}C NMR spectrum of Compound **17** in $CDCl_3$

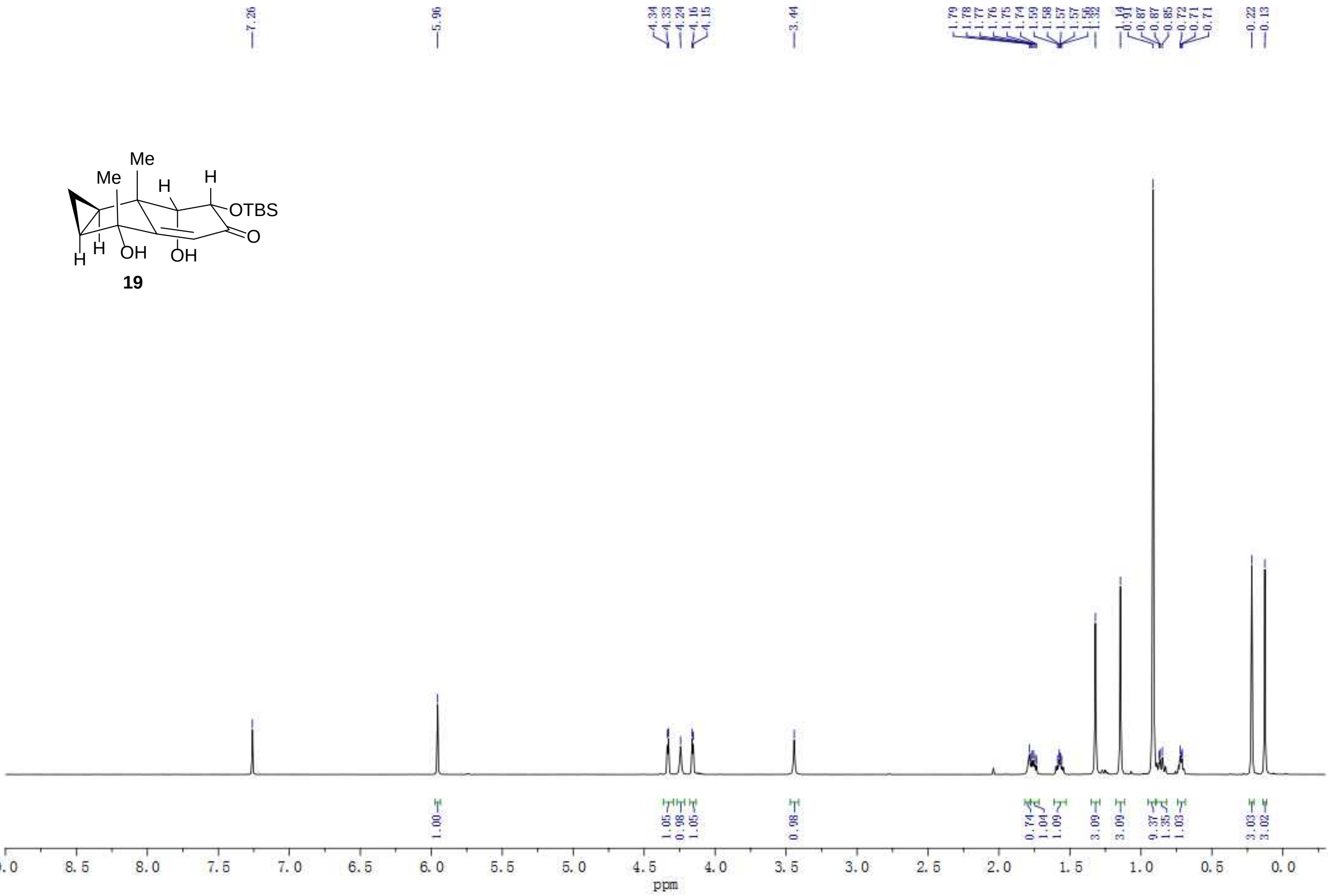

Supplementary Figure 31 ^{1}H NMR spectrum of Compound **19** in $CDCl_3$

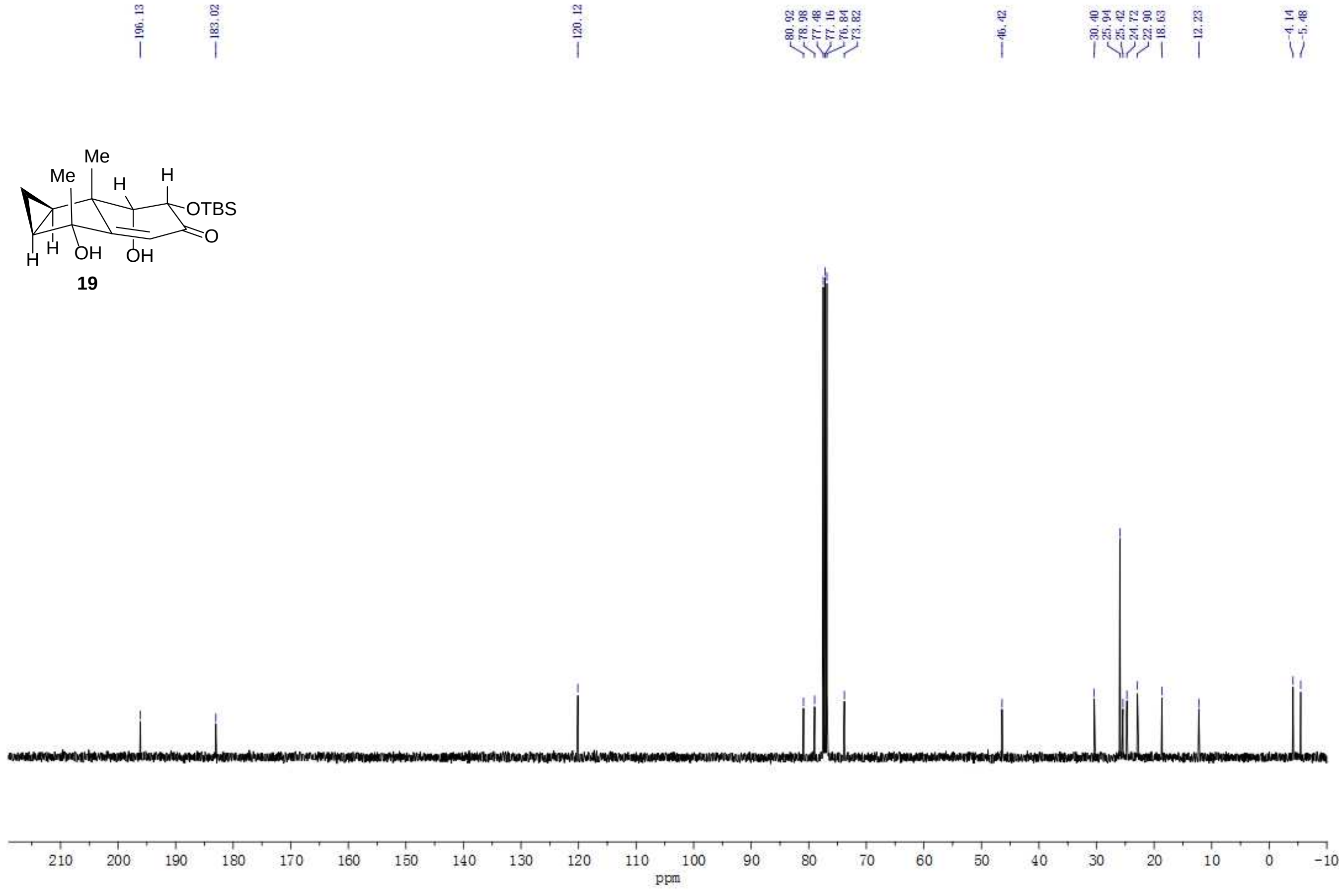

Supplementary Figure 32 ^{13}C NMR spectrum of Compound **19** in $CDCl_3$

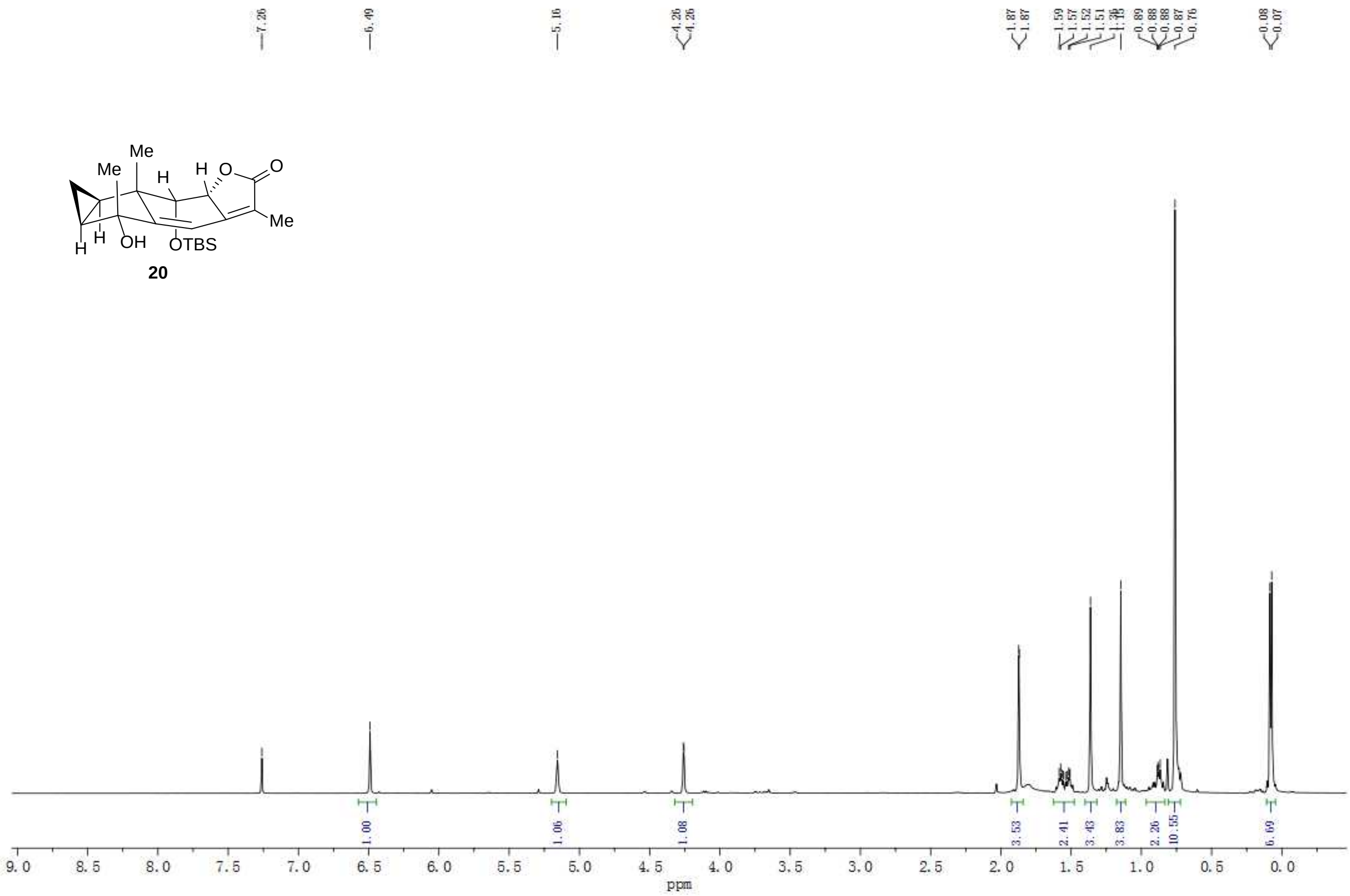

Supplementary Figure 33 ^{1}H NMR spectrum of Compound **20** in $CDCl_3$

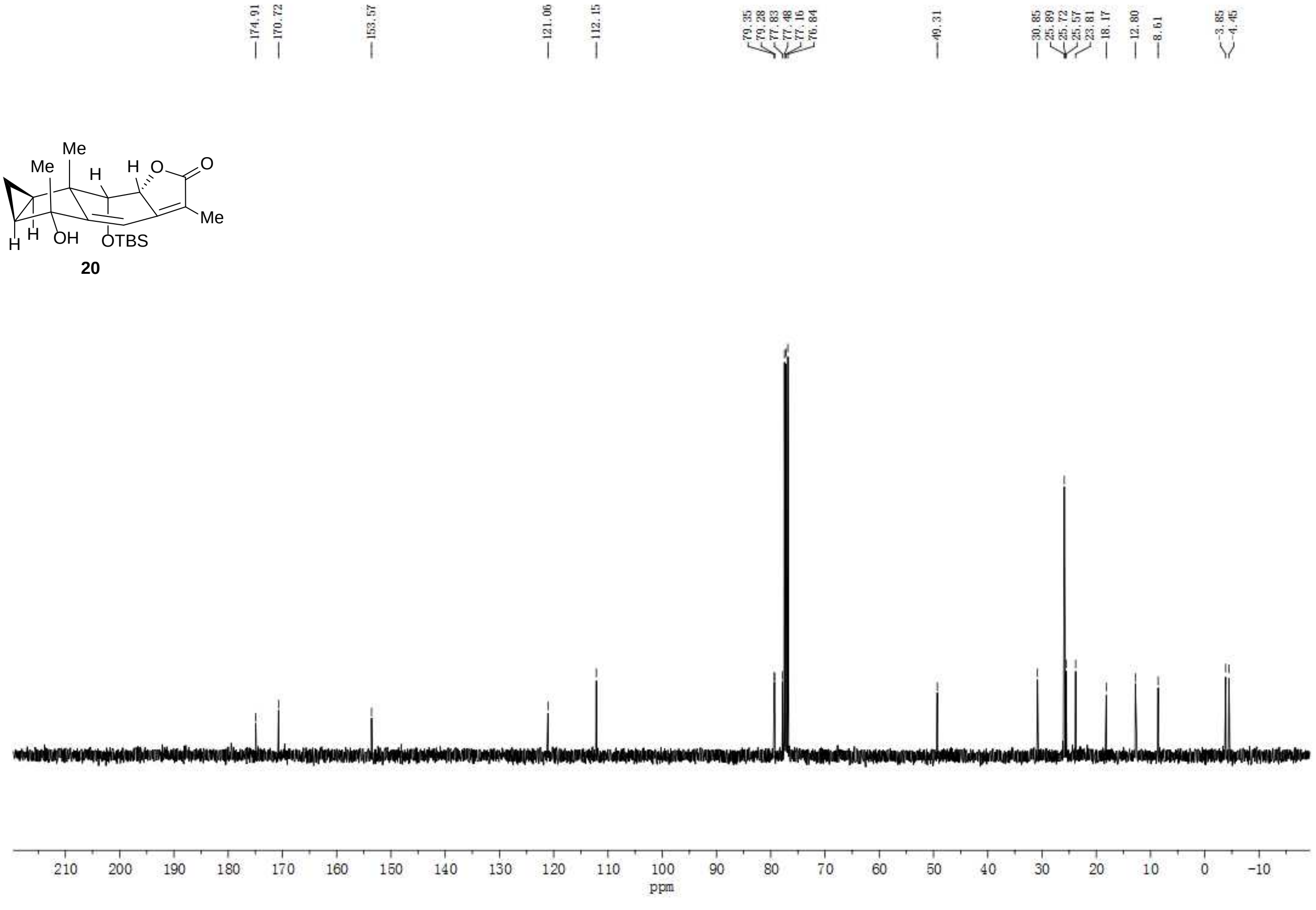

Supplementary Figure 34 ^{13}C NMR spectrum of Compound **20** in $CDCl_3$

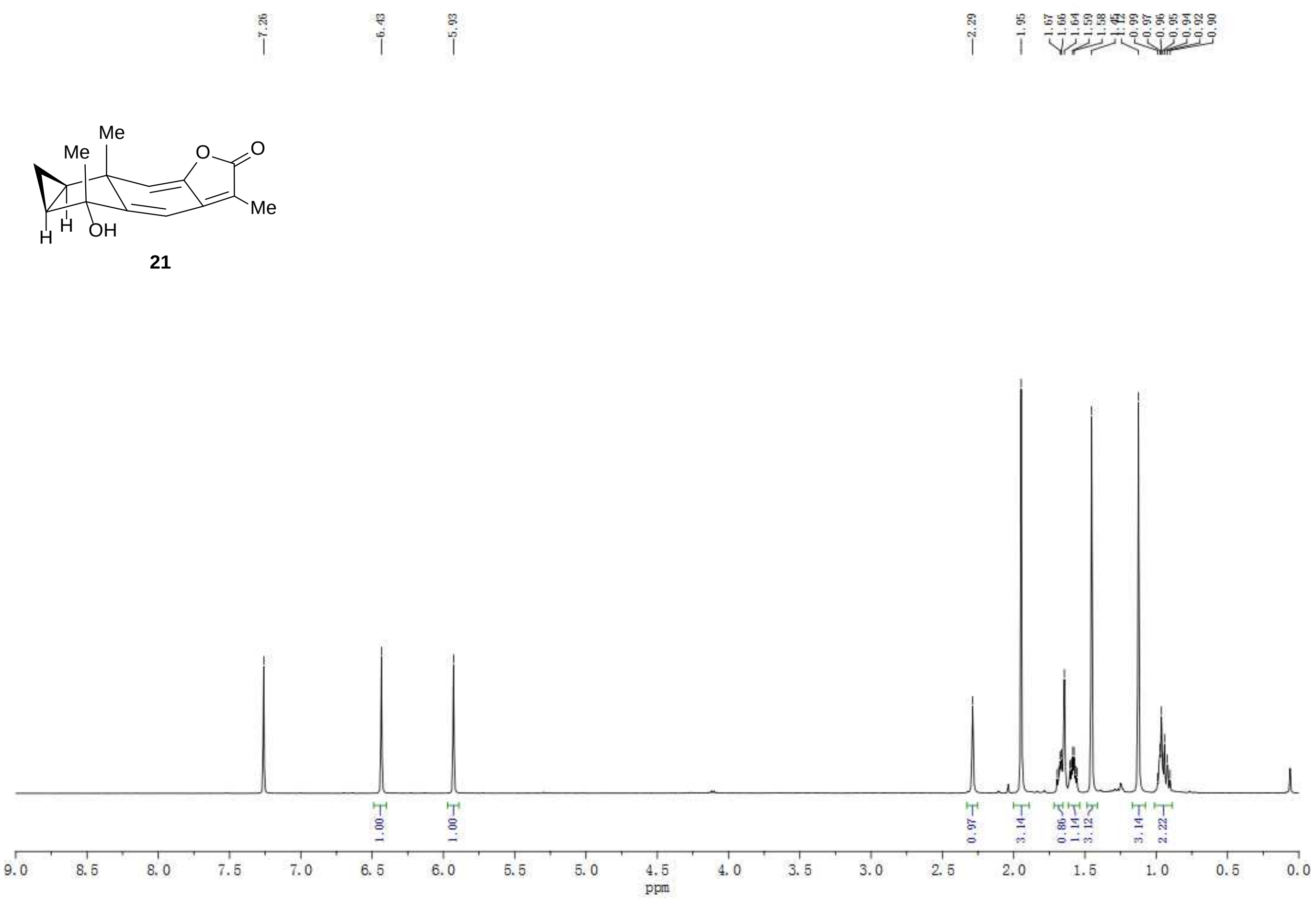

Supplementary Figure 35 ^{1}H NMR spectrum of Compound **21** in $CDCl_3$

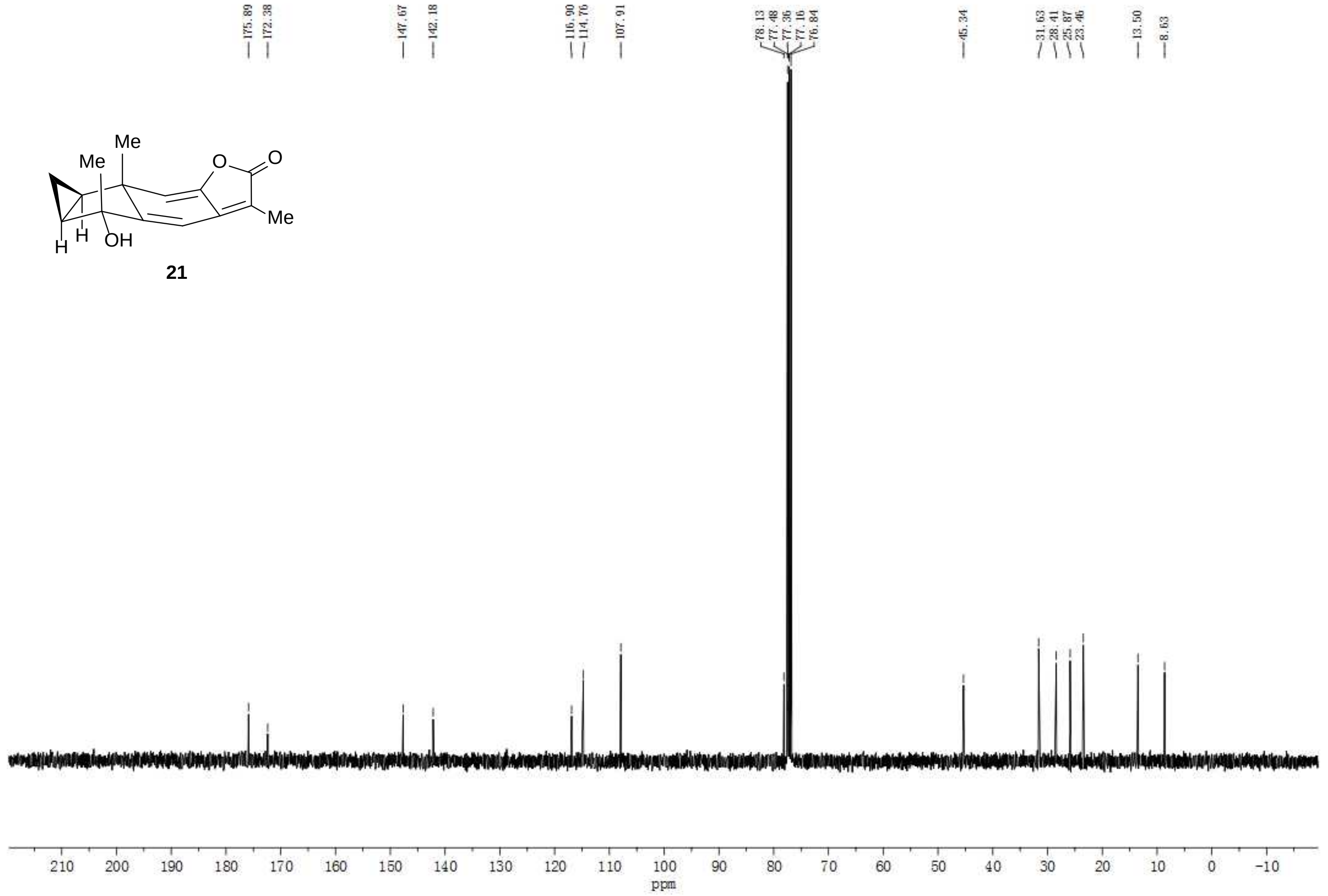

Supplementary Figure 36 ^{13}C NMR spectrum of Compound **21** in $CDCl_3$

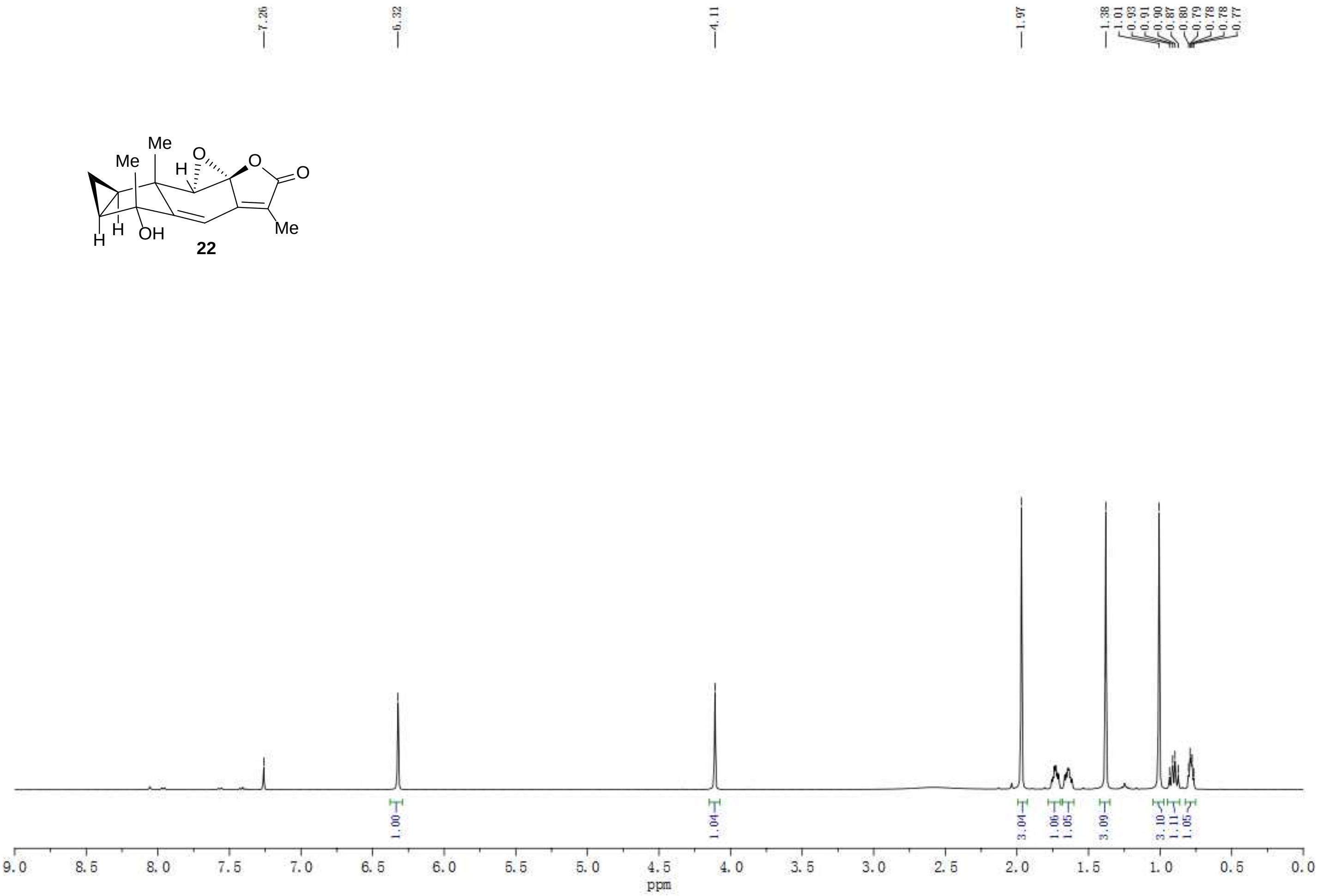

Supplementary Figure 37 ^{1}H NMR spectrum of Compound **22** in $CDCl_3$

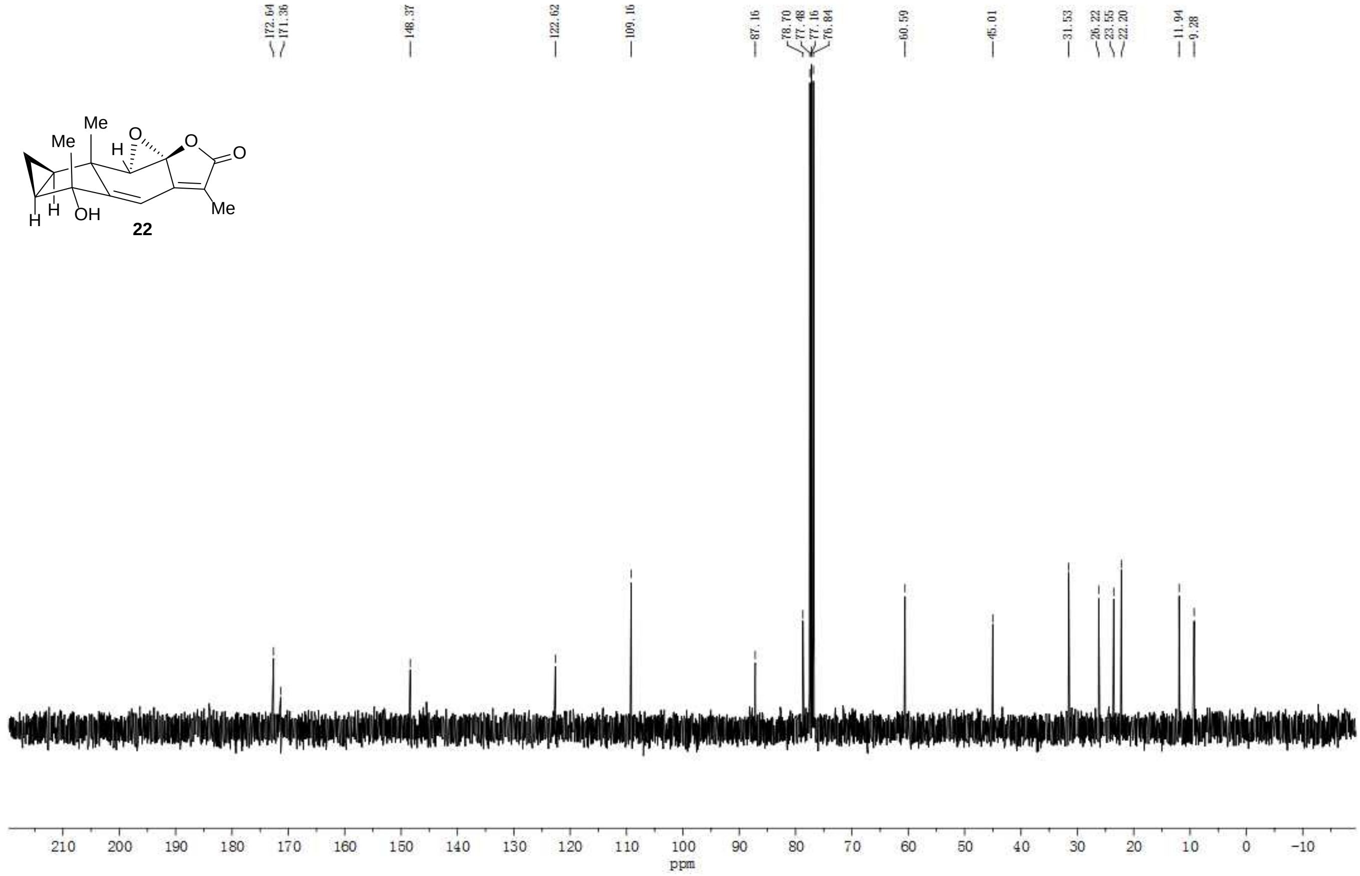

Supplementary Figure 38 ^{13}C NMR spectrum of Compound **22** in $CDCl_3$

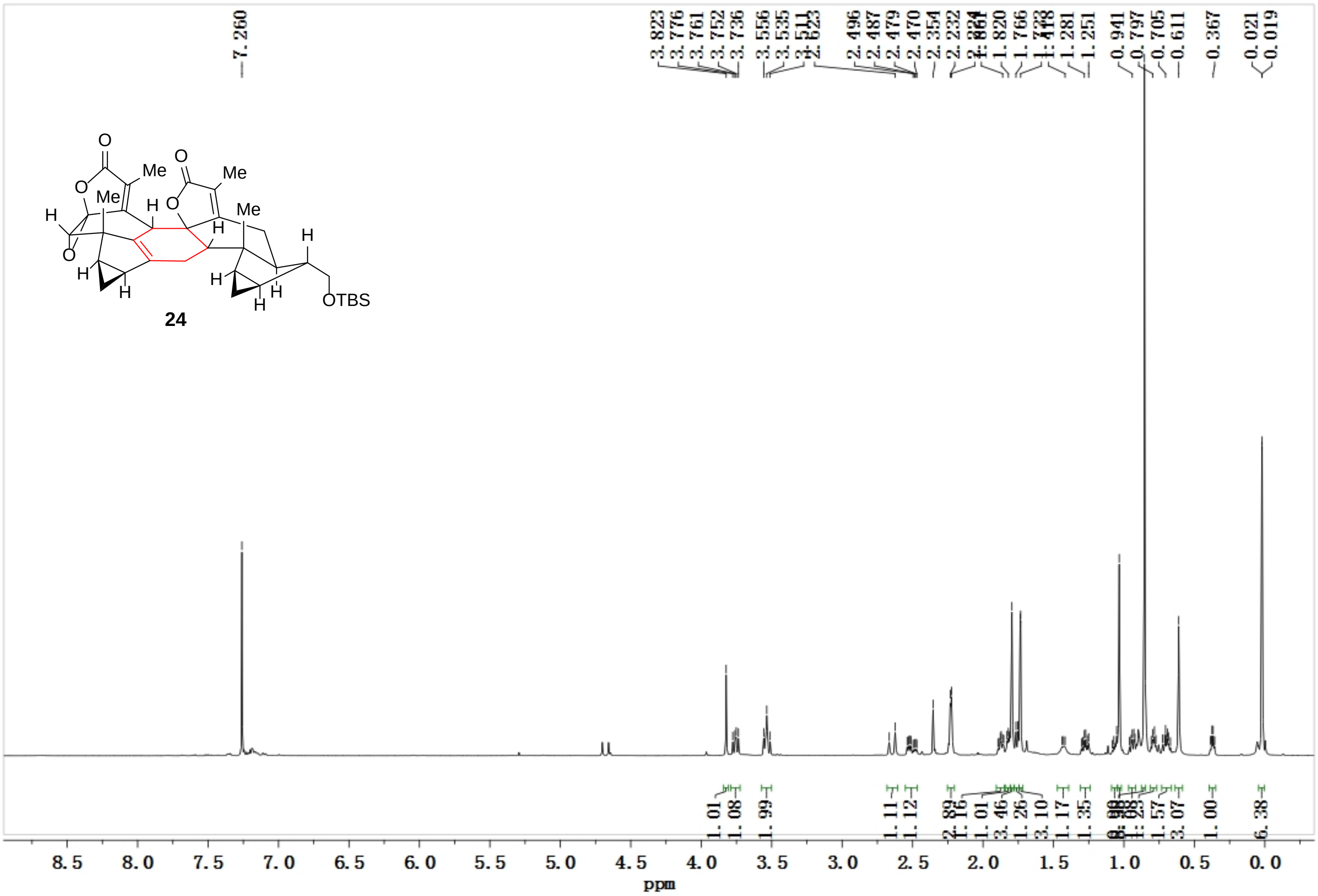

Supplementary Figure 39 ^{1}H NMR spectrum of Compound **24** in $CDCl_3$

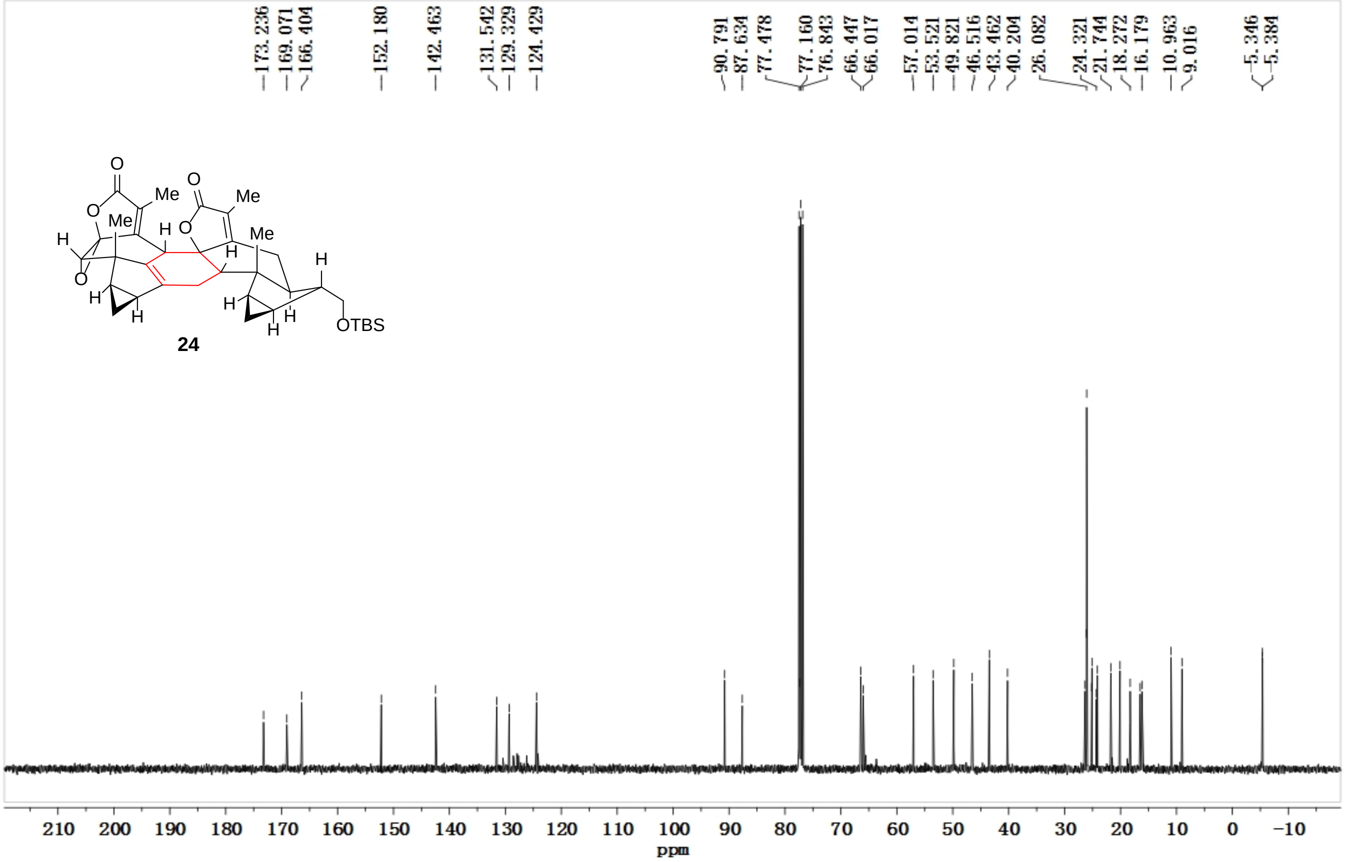

Supplementary Figure 40 ^{13}C NMR spectrum of Compound **24** in $CDCl_3$

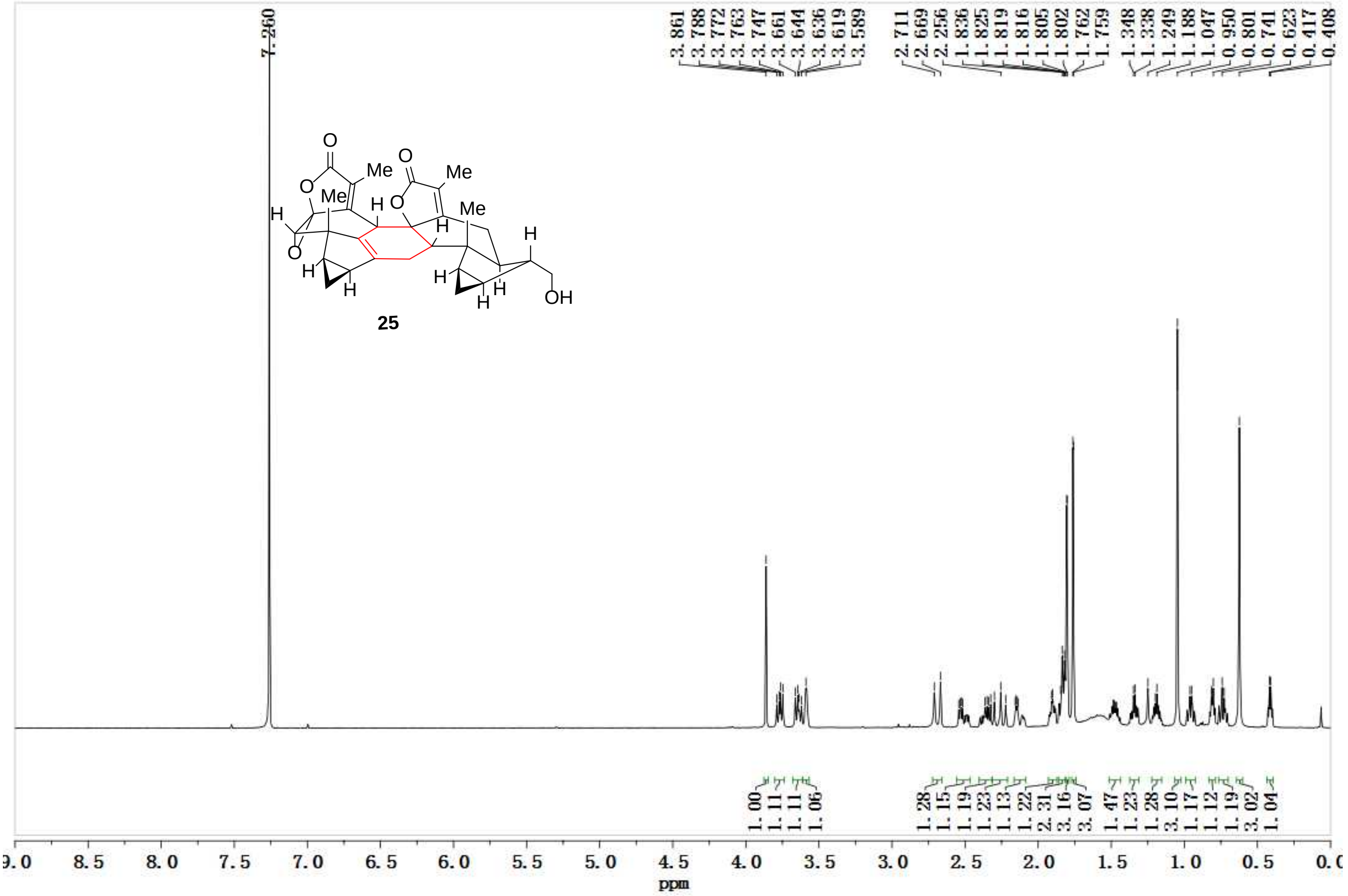

Supplementary Figure 41 ^{1}H NMR spectrum of Compound **25** in $CDCl_3$

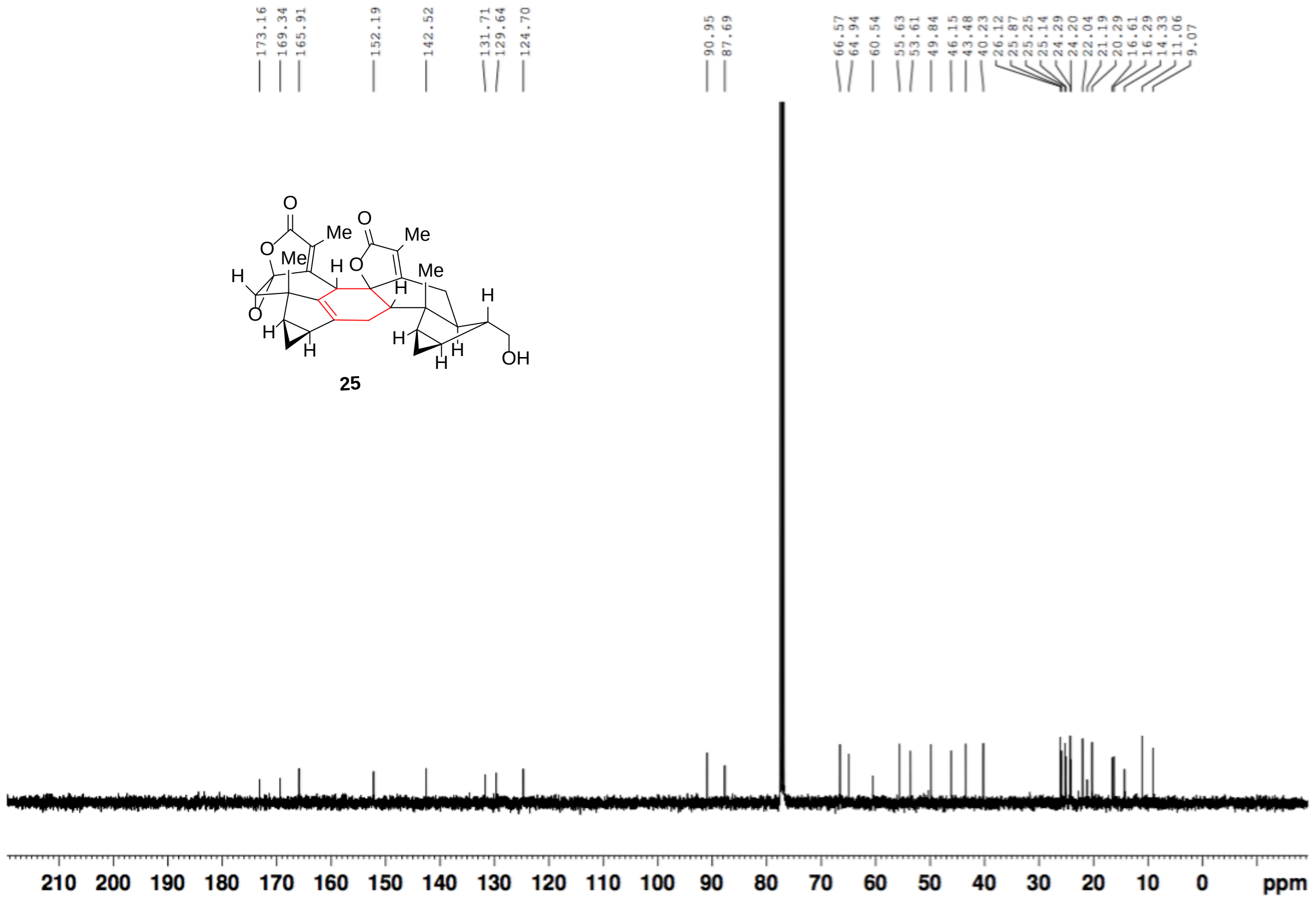

Supplementary Figure 42 ^{13}C NMR spectrum of Compound **25** in $CDCl_3$

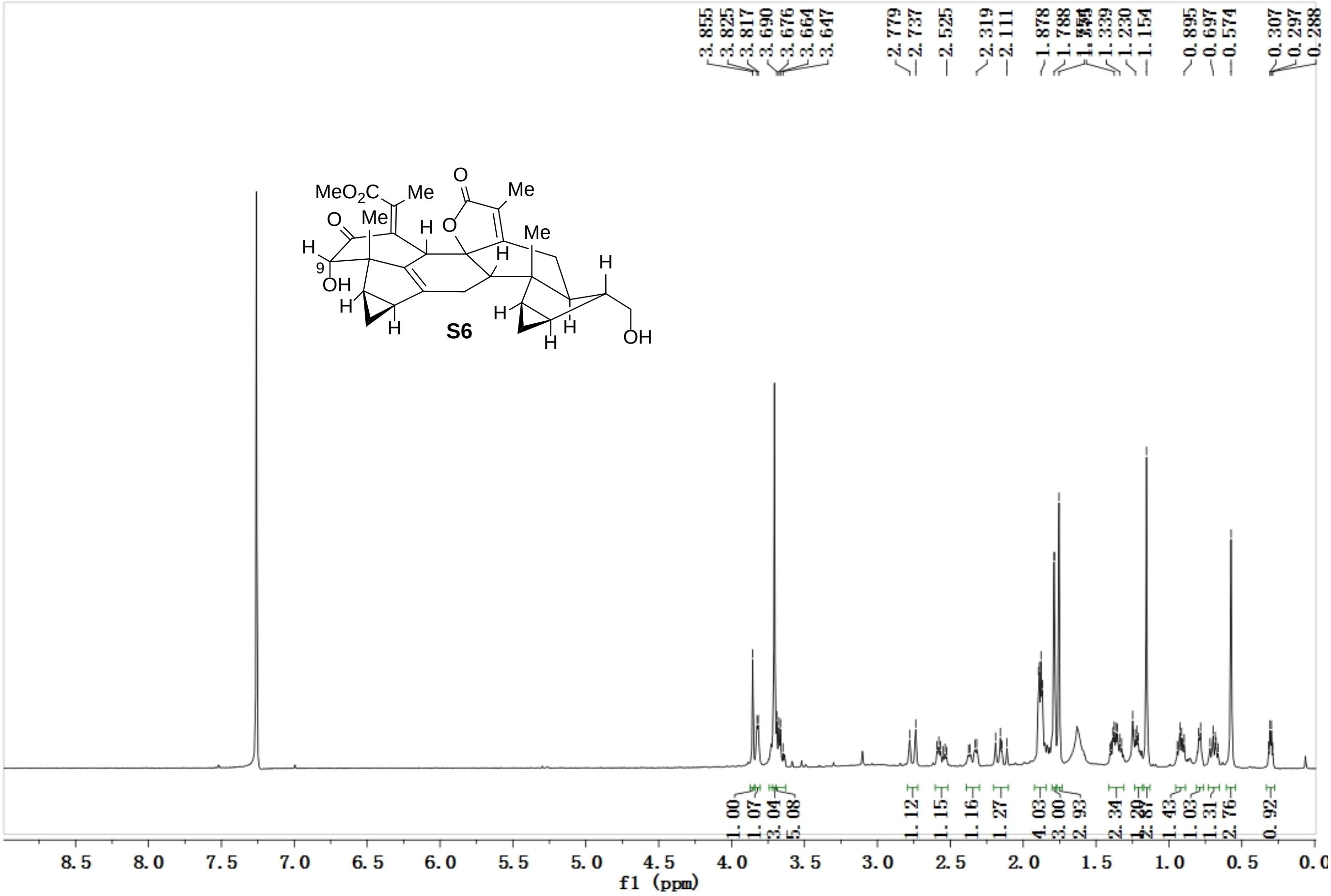

Supplementary Figure 43 ^{1}H NMR spectrum of Compound **S6** in $CDCl_3$

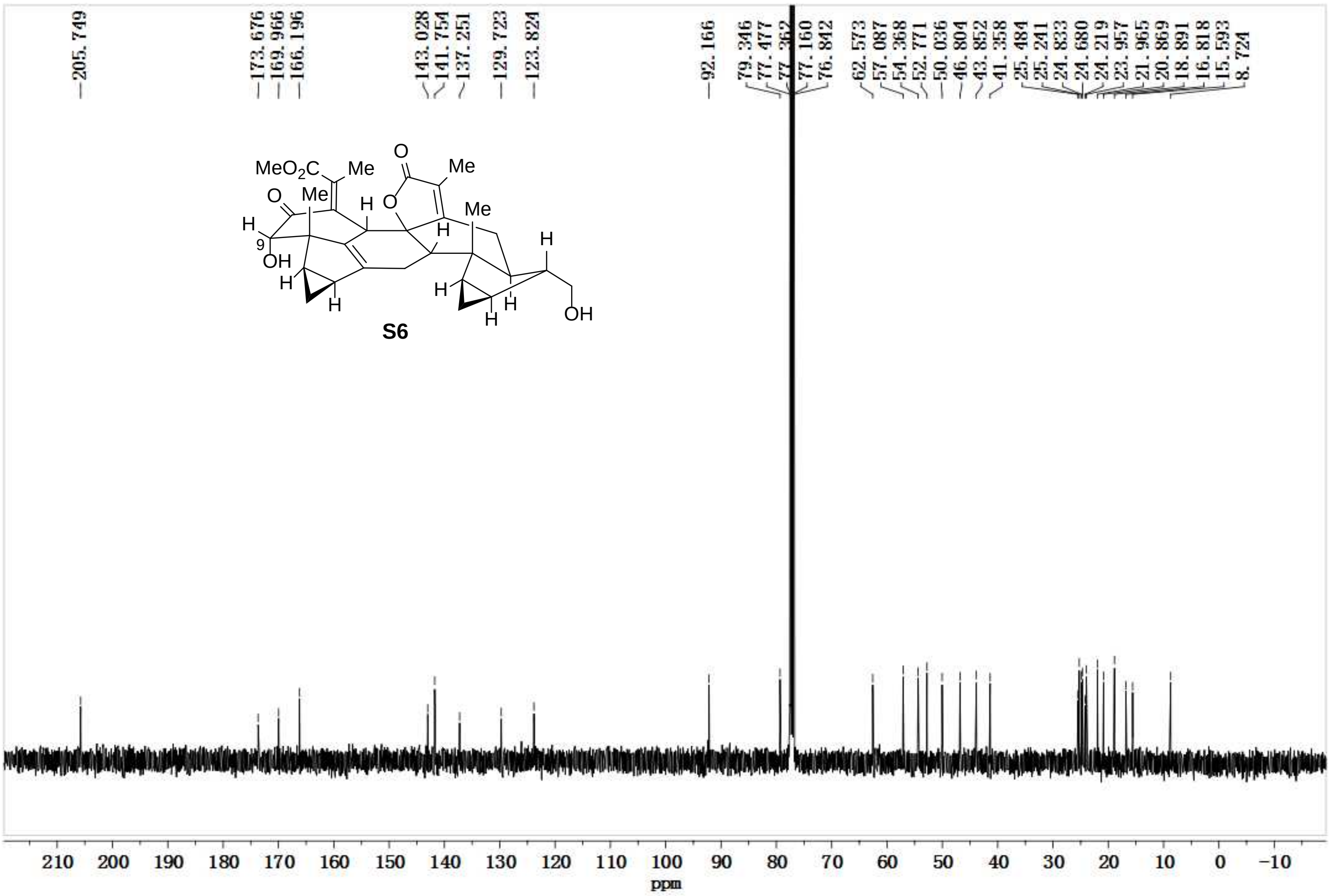

Supplementary Figure 44 ^{13}C NMR spectrum of Compound **S6** in $CDCl_3$

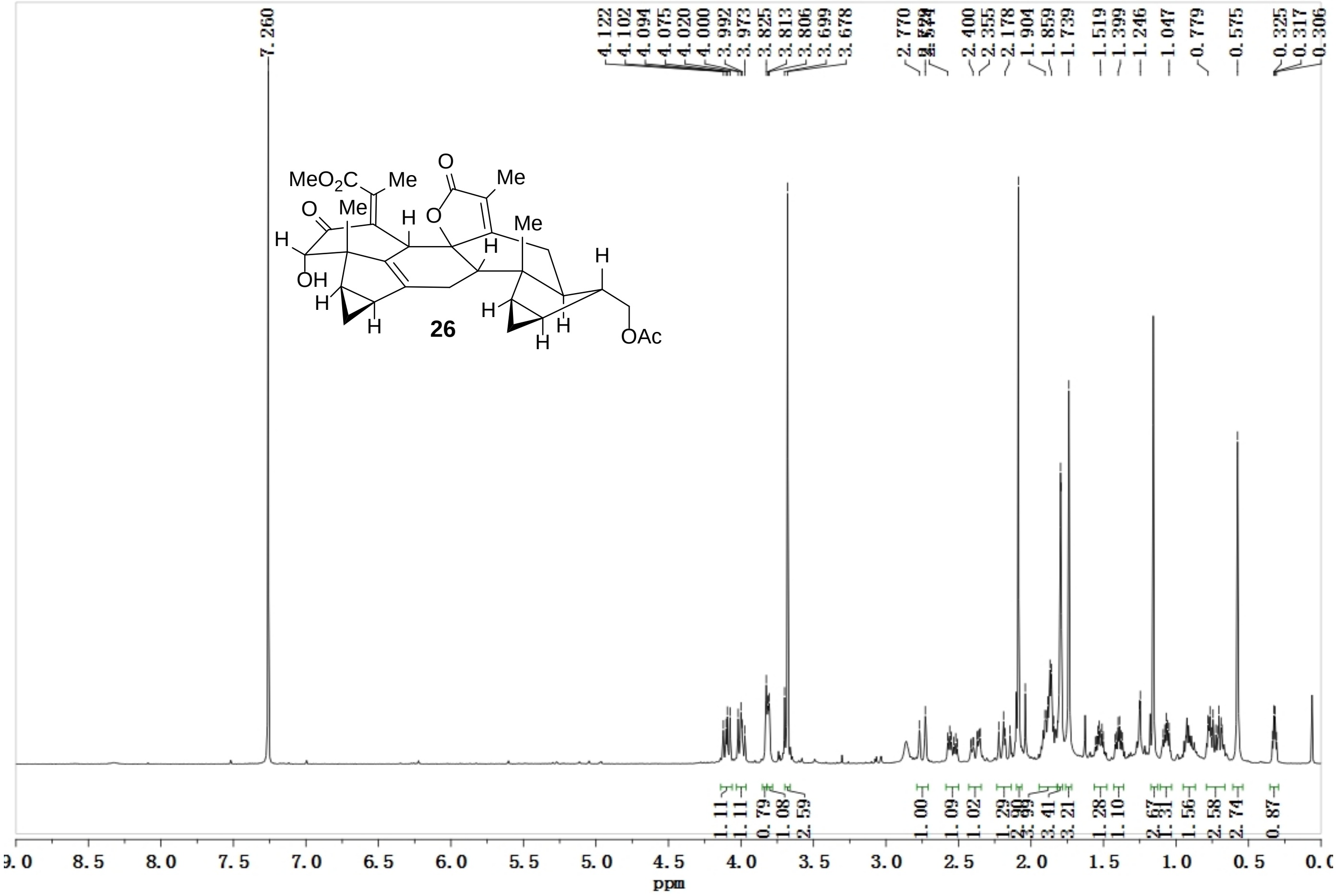

Supplementary Figure 45 ^{1}H NMR spectrum of Compound **26** in $CDCl_3$

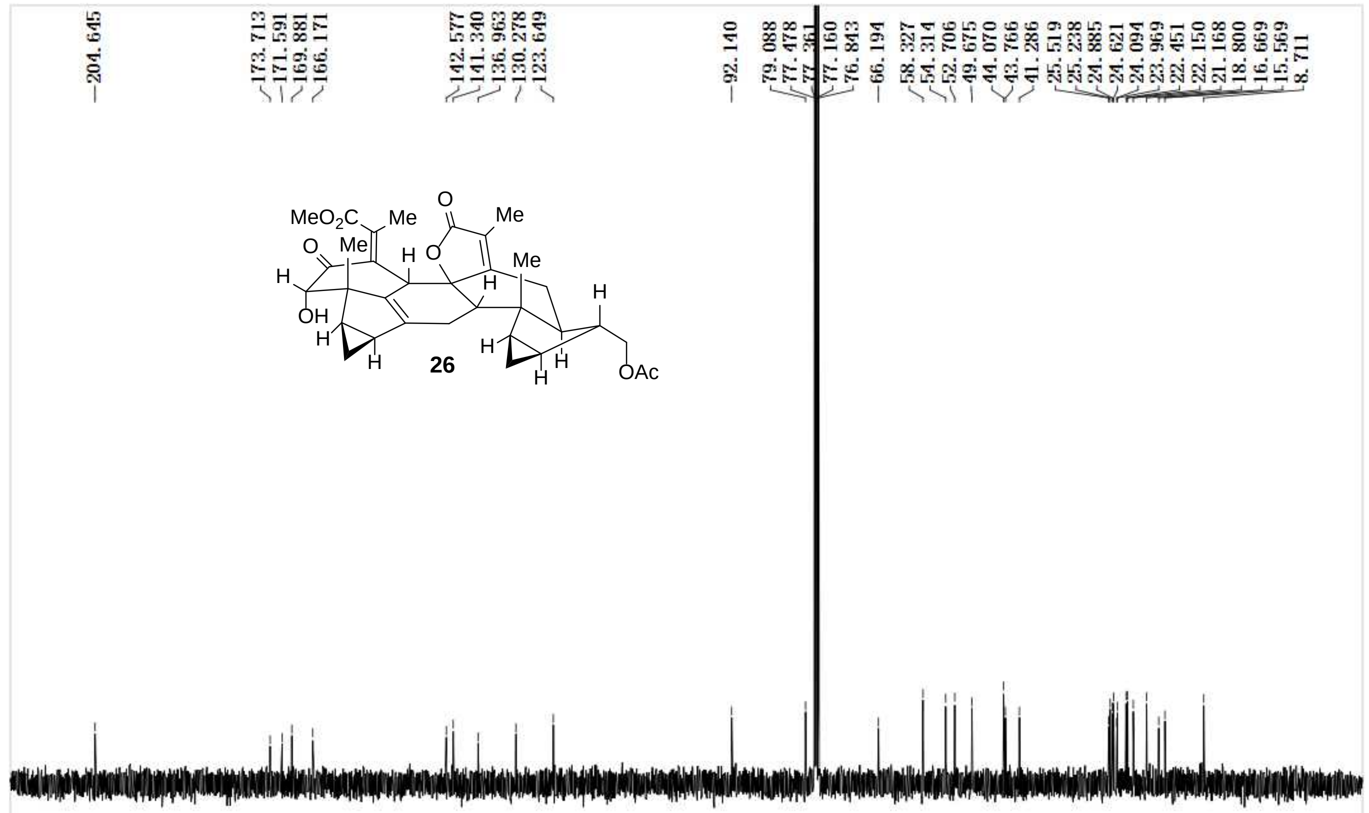

Supplementary Figure 46 ^{13}C NMR spectrum of Compound **26** in $CDCl_3$

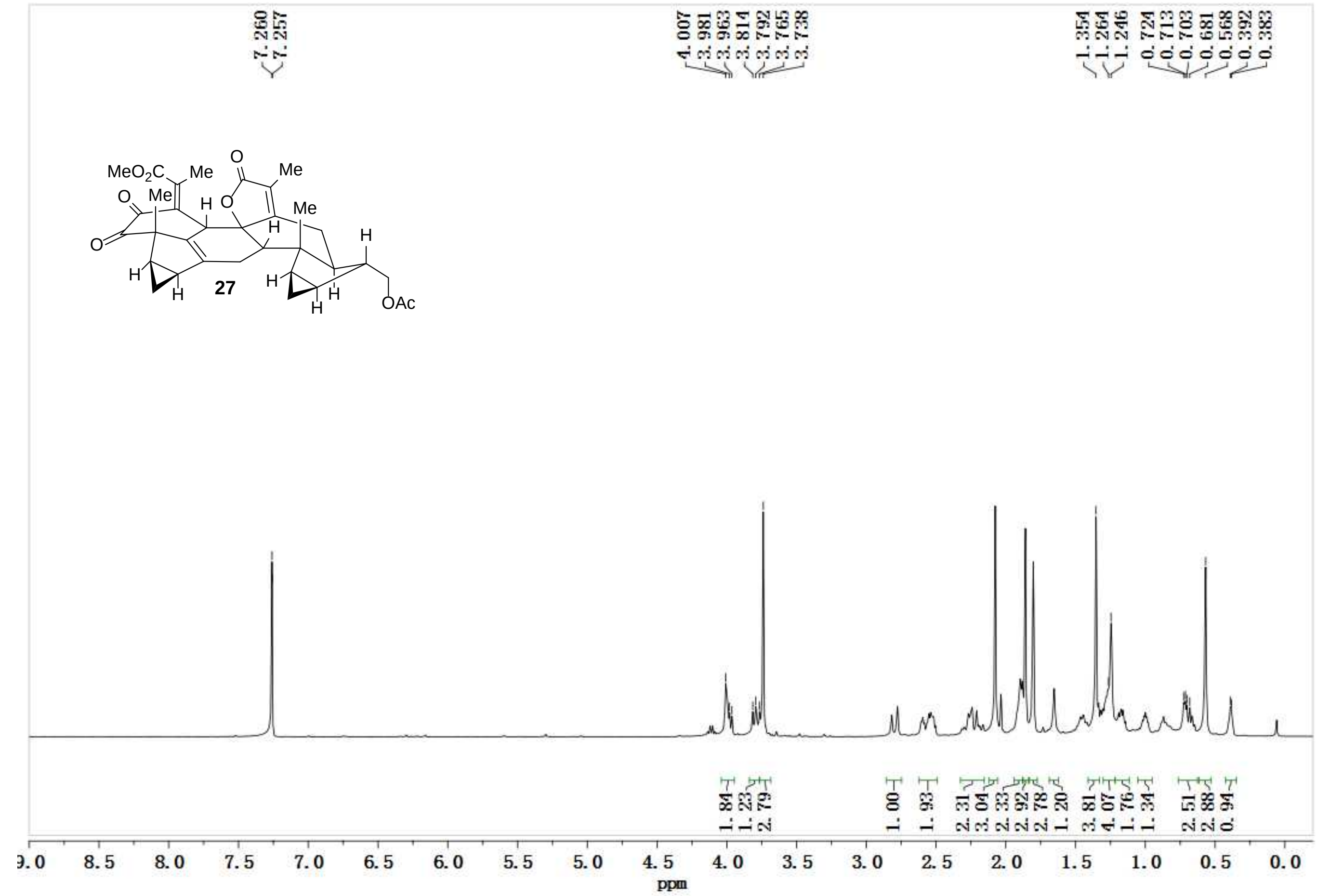

Supplementary Figure 47 ^{1}H NMR spectrum of Compound **27** in $CDCl_3$

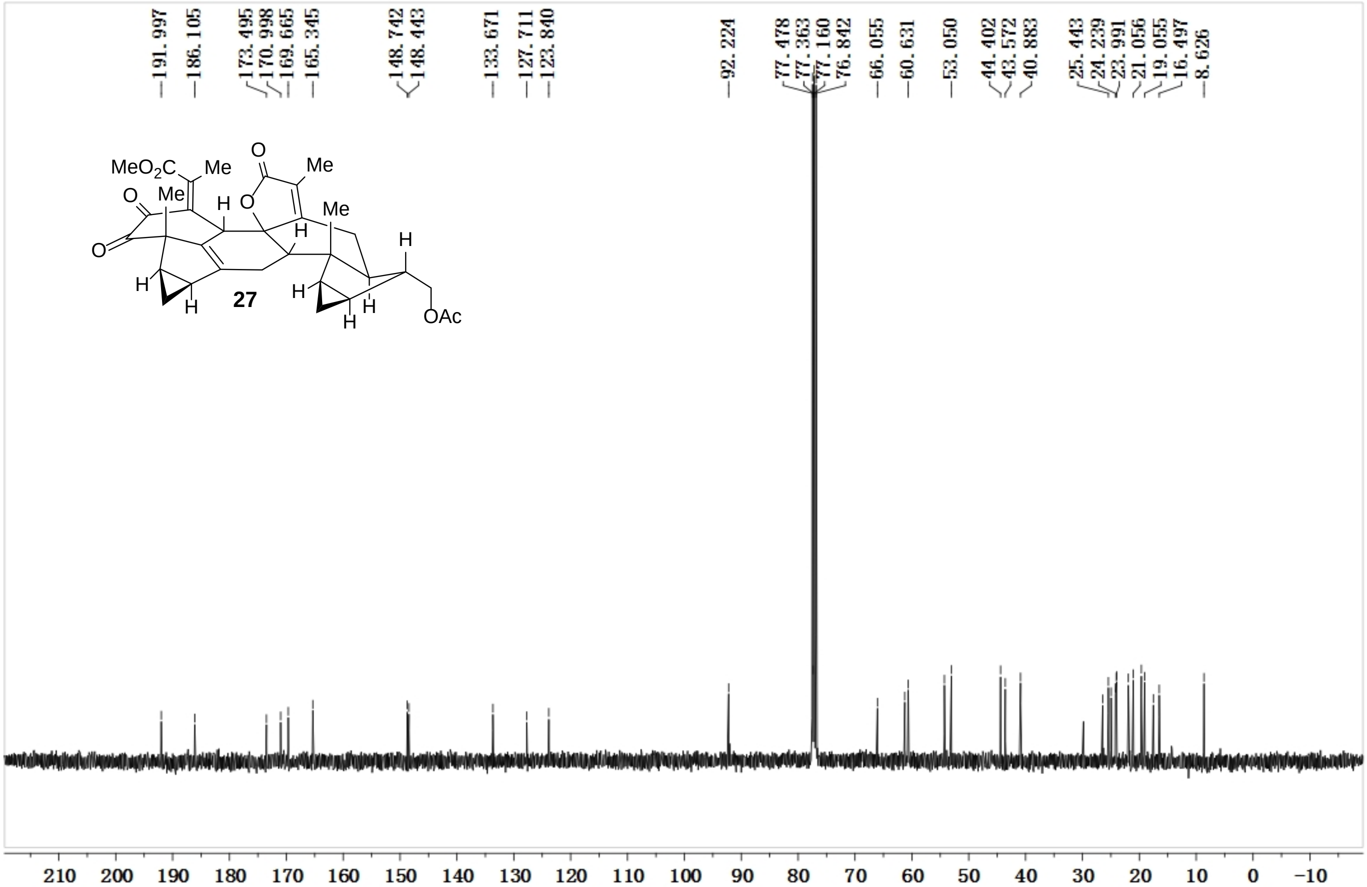

Supplementary Figure 48 ^{13}C NMR spectrum of Compound **27** in $CDCl_3$

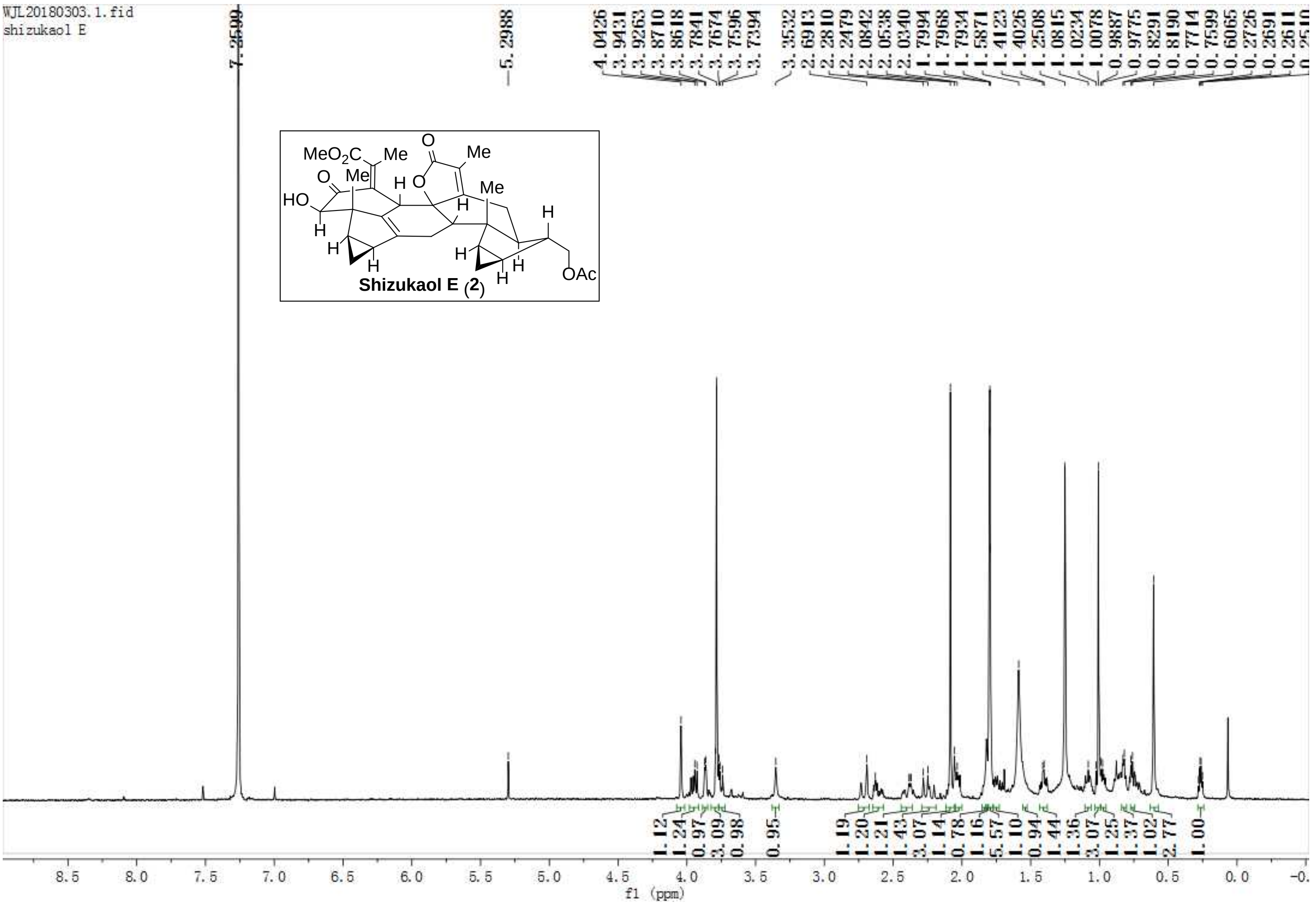

Supplementary Figure 49 ^{1}H NMR spectrum of shizukaol E (**2**) in $CDCl_3$

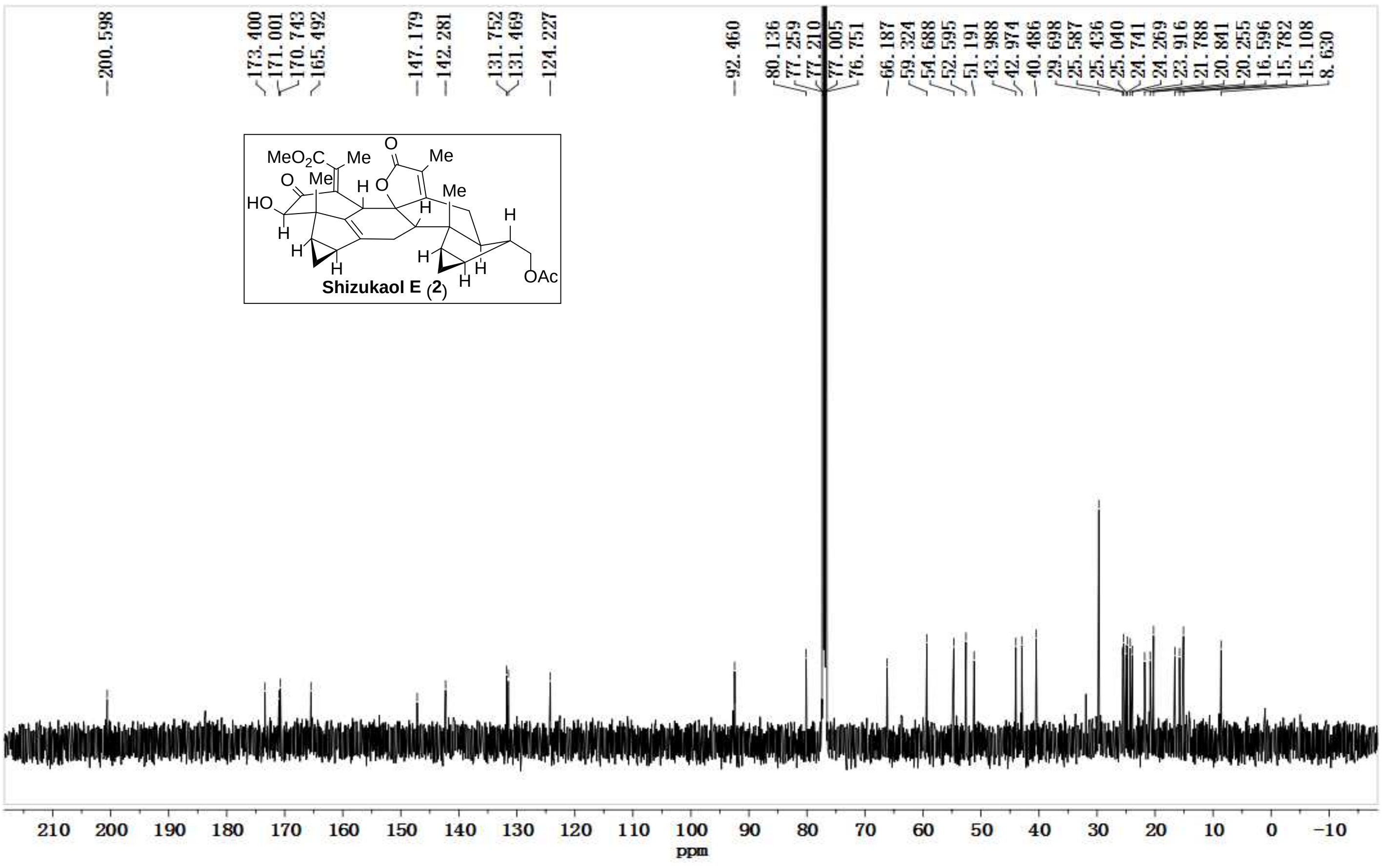

Supplementary Figure 50 ^{13}C NMR spectrum of shizukaol E (**2**) in $CDCl_3$

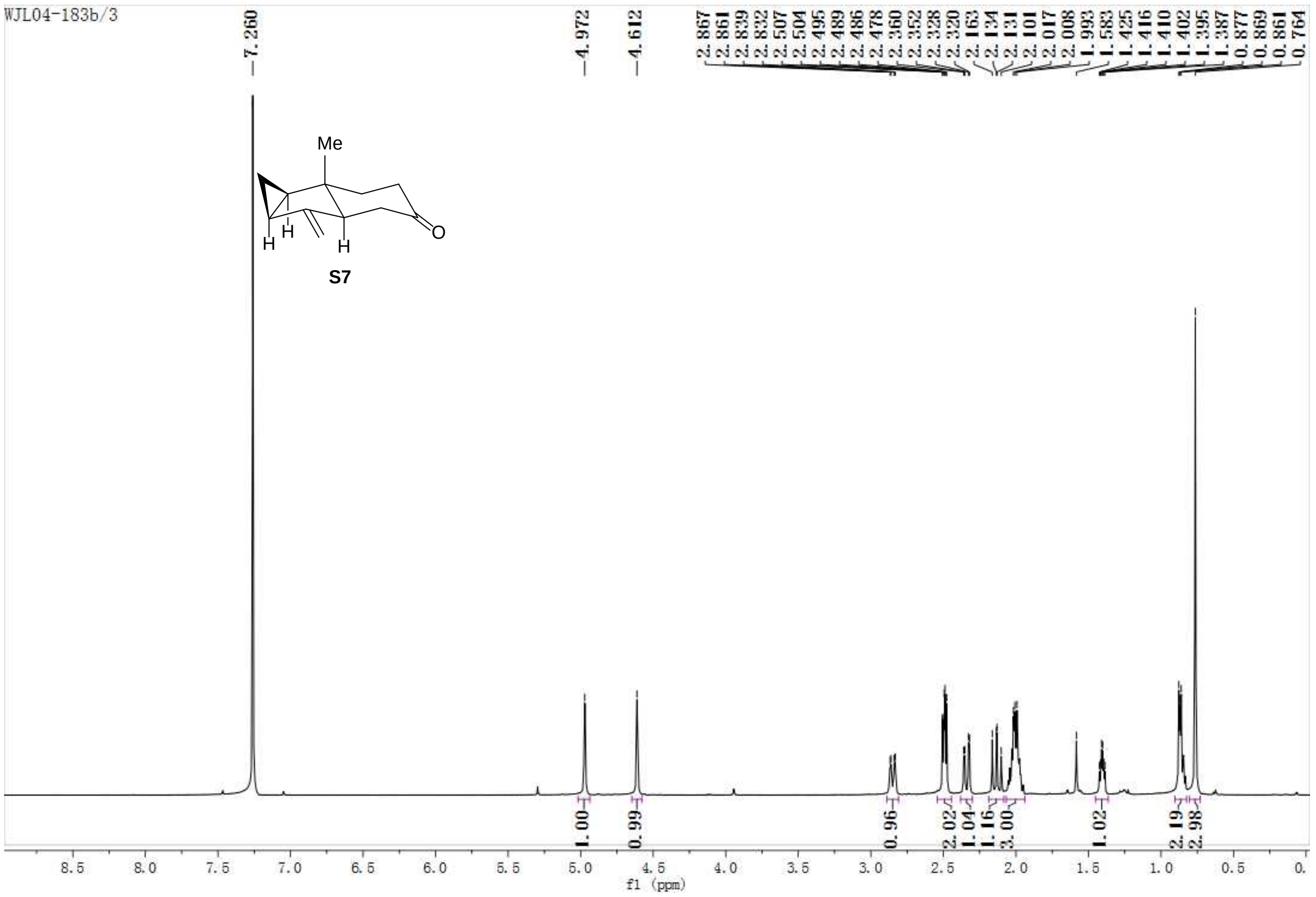

Supplementary Figure 51 ^{1}H NMR spectrum of Compound **S7** in $CDCl_3$

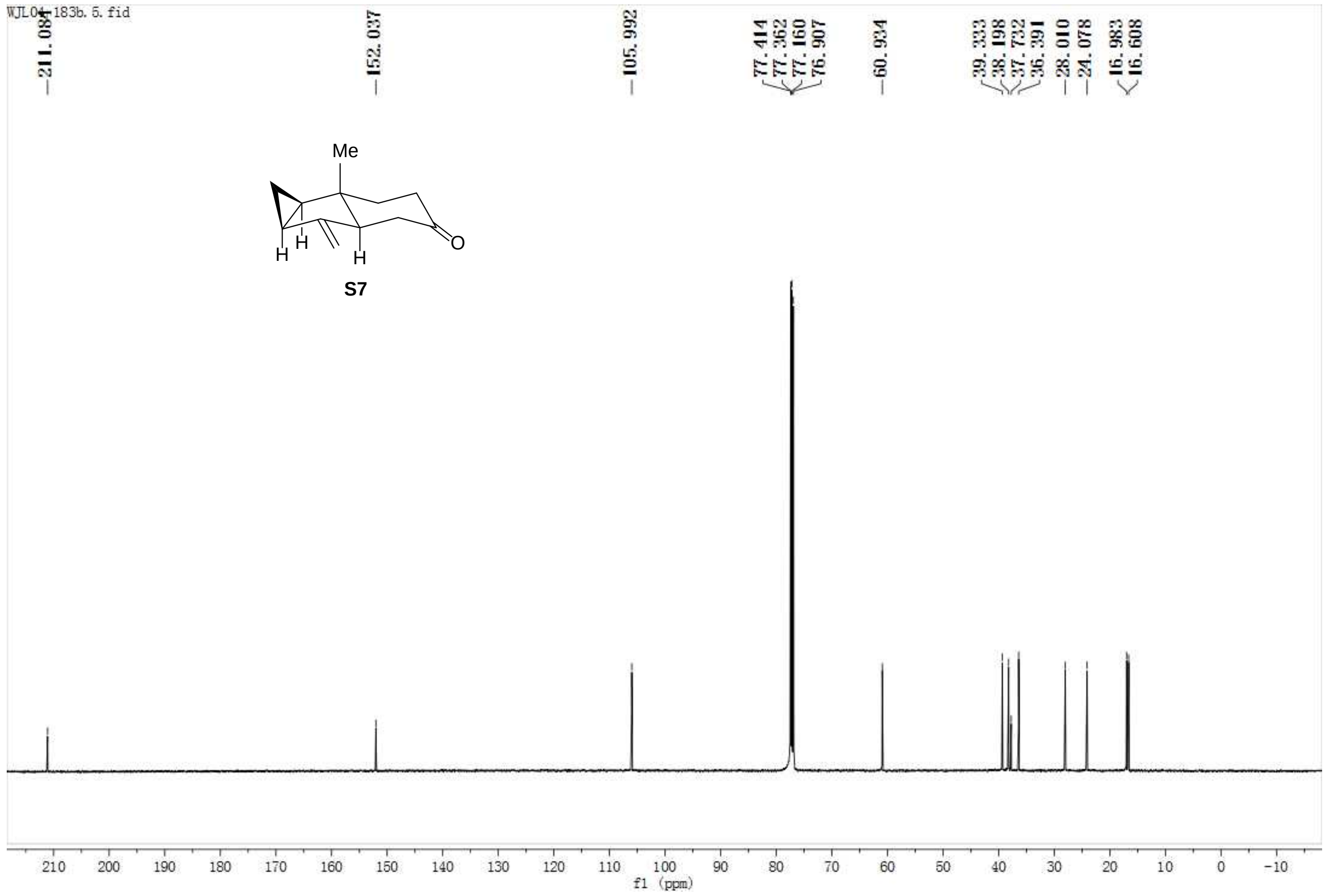

Supplementary Figure 52 ^{13}C NMR spectrum of Compound **S7** in $CDCl_3$

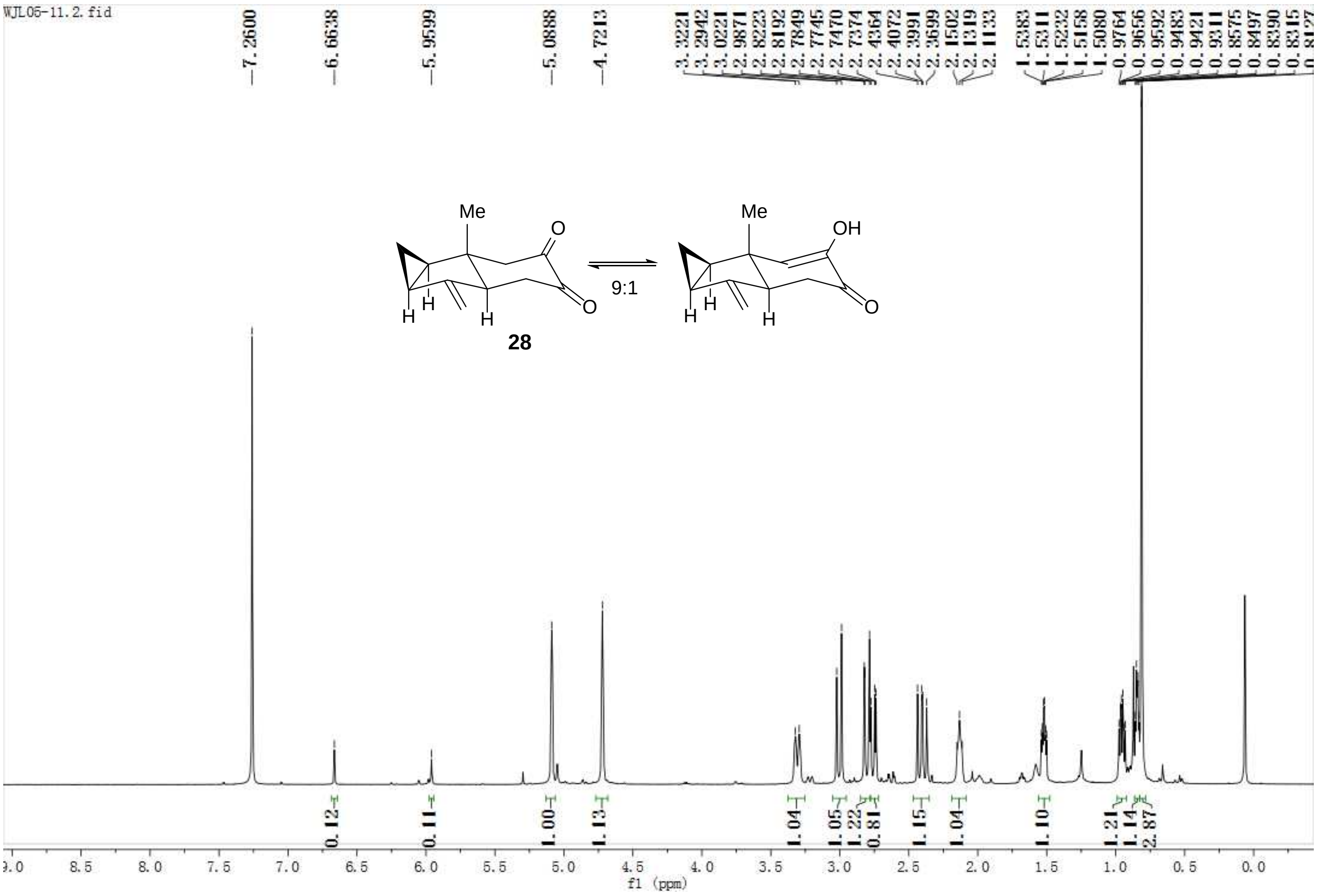

Supplementary Figure 53 ^{1}H NMR spectrum of Compound **28** in $CDCl_3$

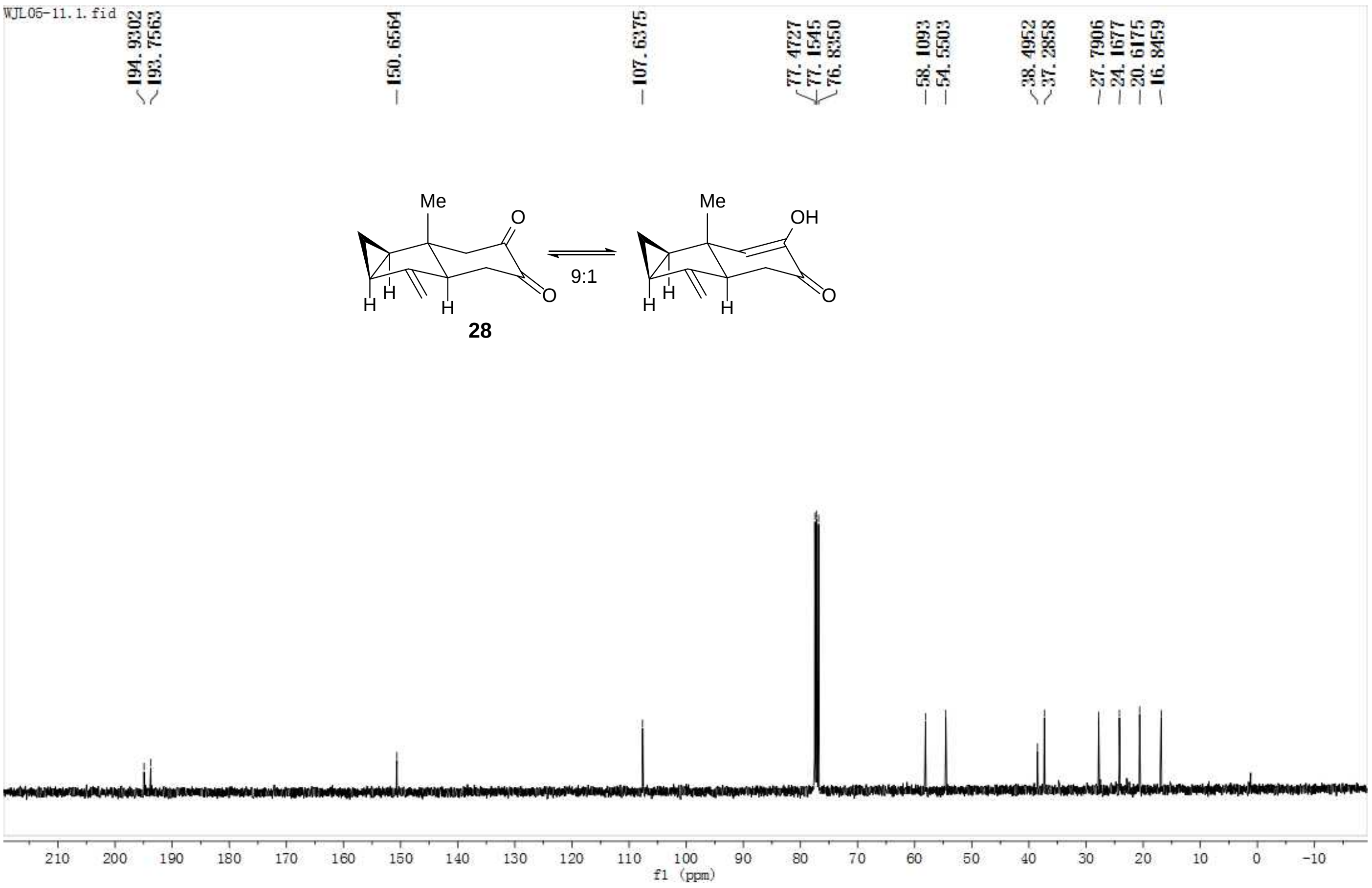

Supplementary Figure 54 ^{13}C NMR spectrum of Compound **28** in $CDCl_3$

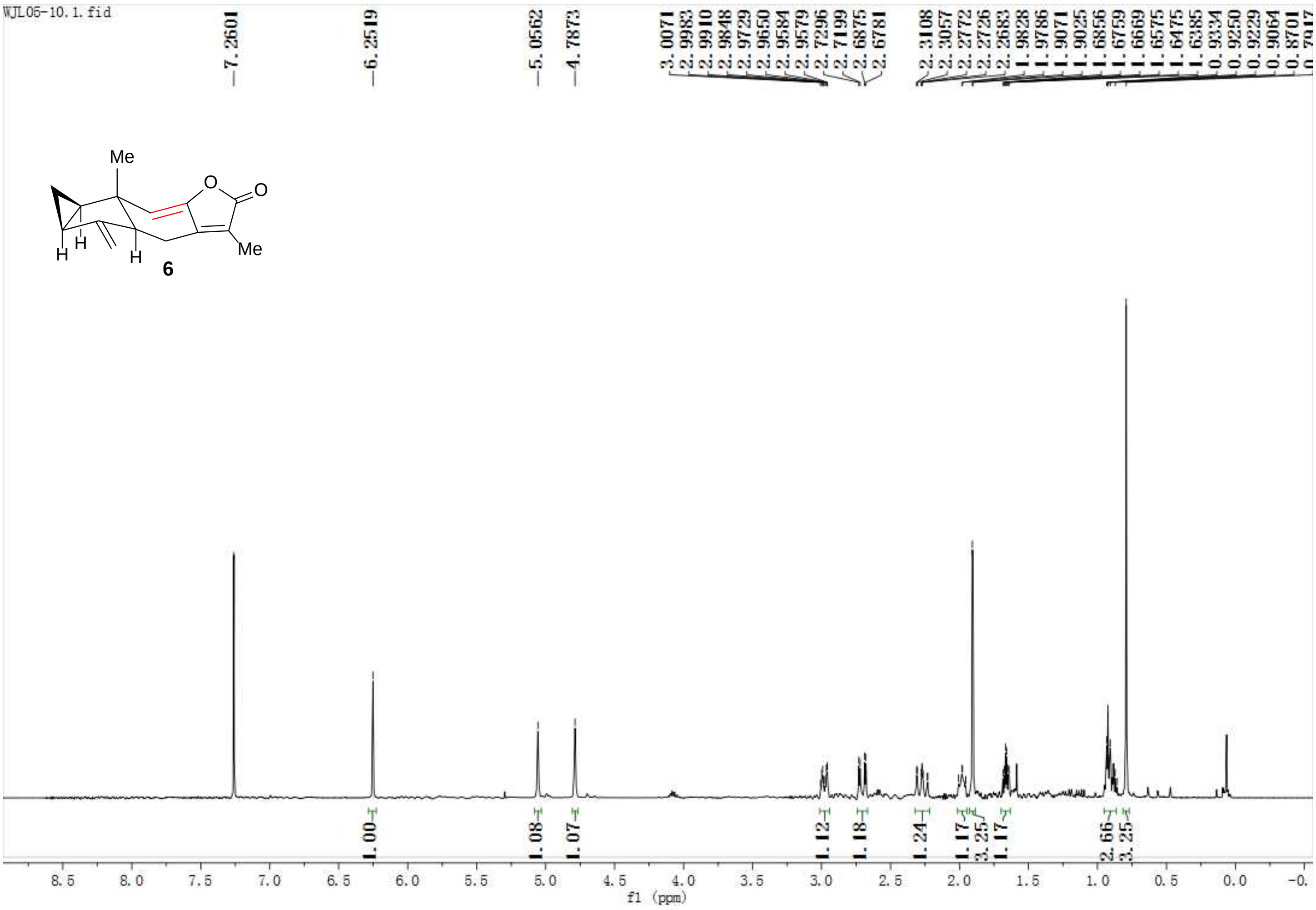

Supplementary Figure 55 ^{1}H NMR spectrum of Compound **6** in $CDCl_3$

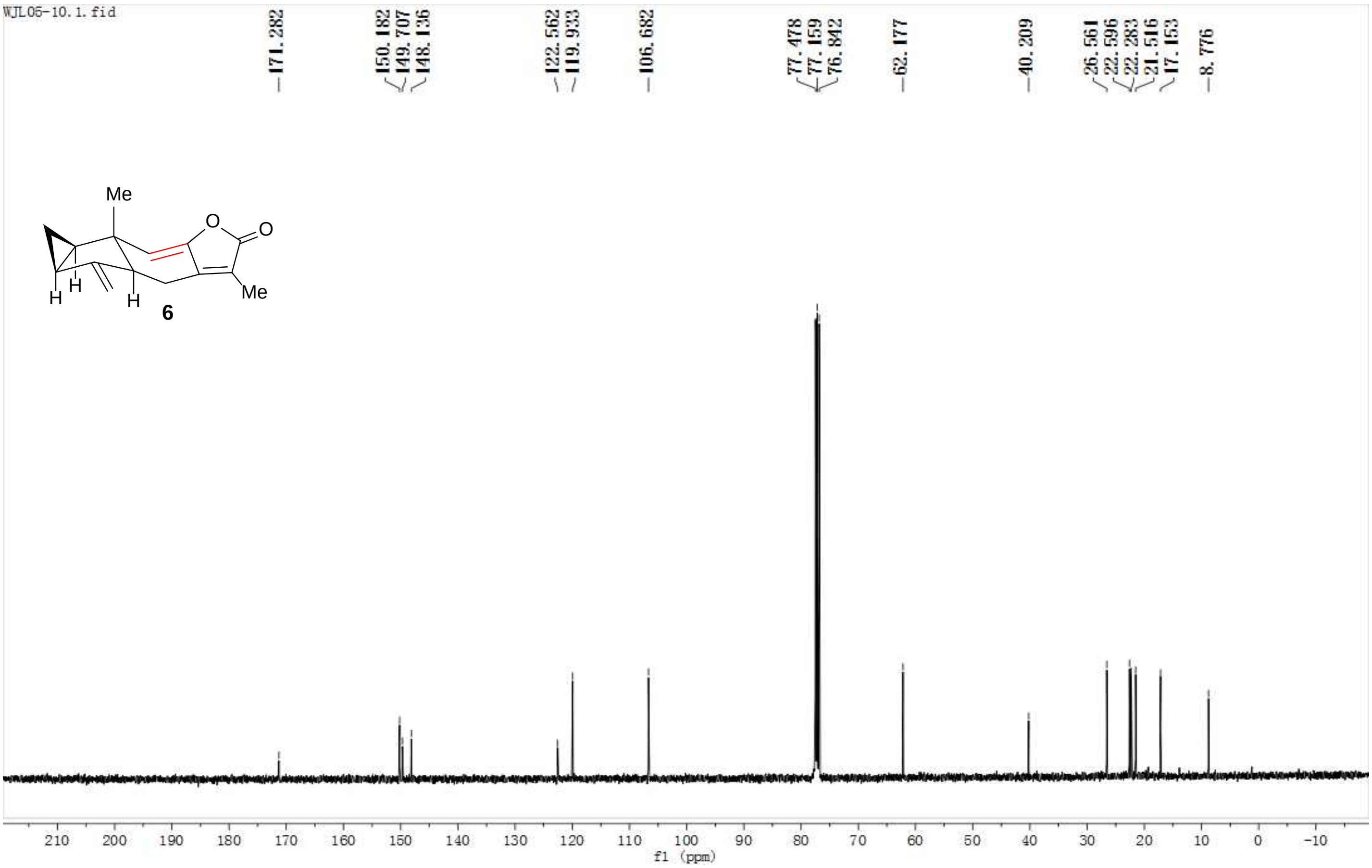

Supplementary Figure 56 ^{13}C NMR spectrum of Compound **6** in $CDCl_3$

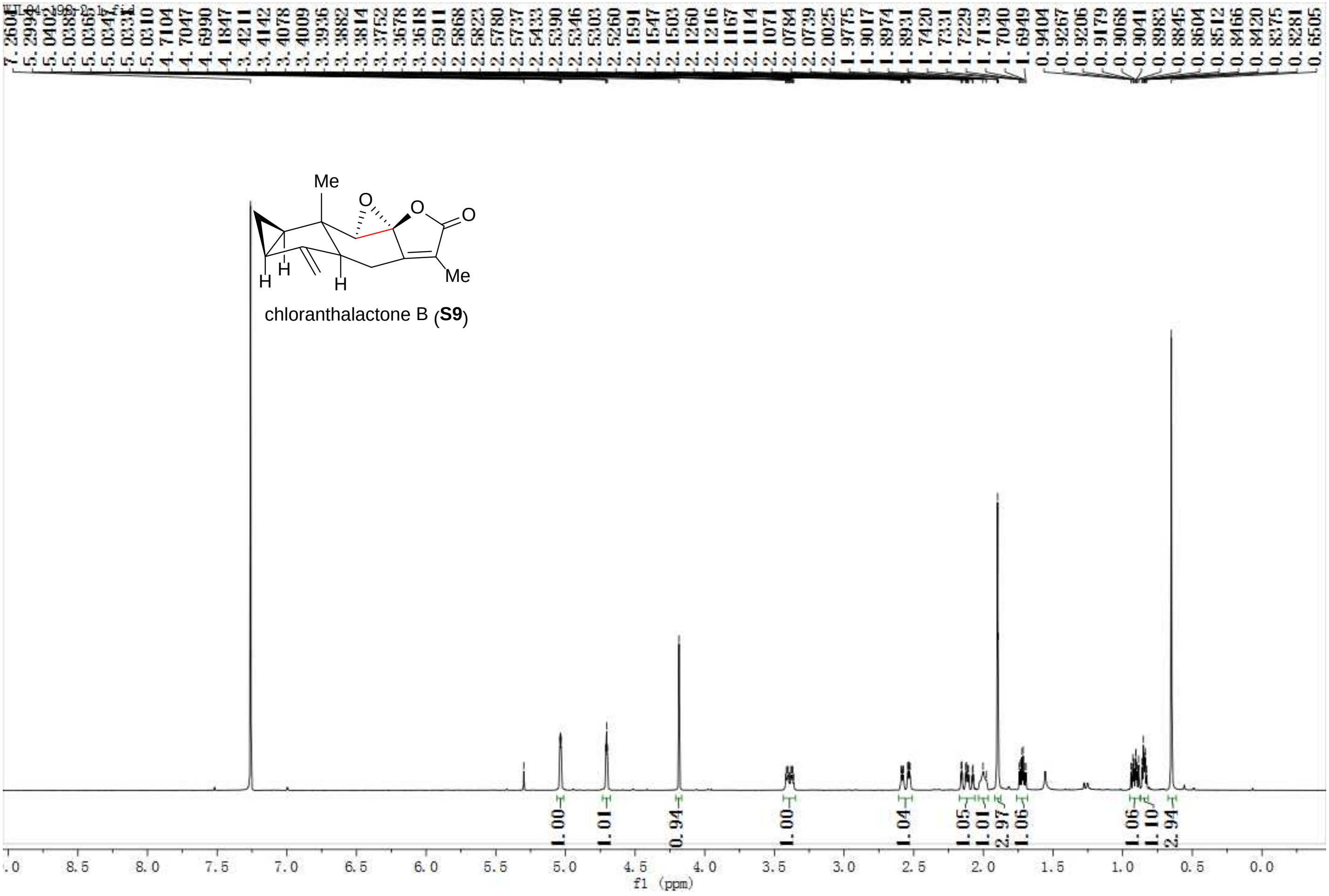

Supplementary Figure 57 ^{1}H NMR spectrum of Compound **S9** in $CDCl_3$

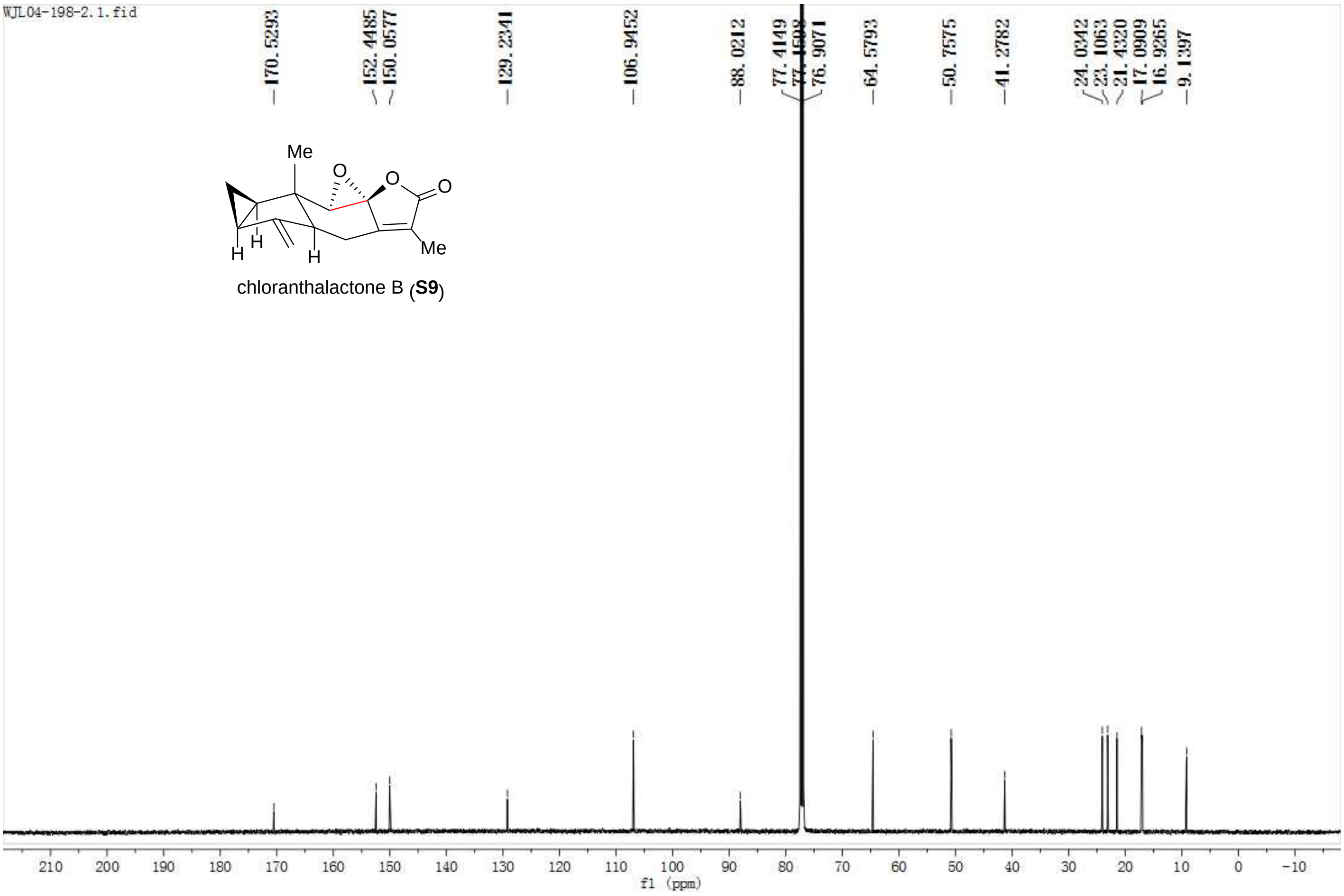

Supplementary Figure 58 ^{13}C NMR spectrum of Compound **S9** in $CDCl_3$

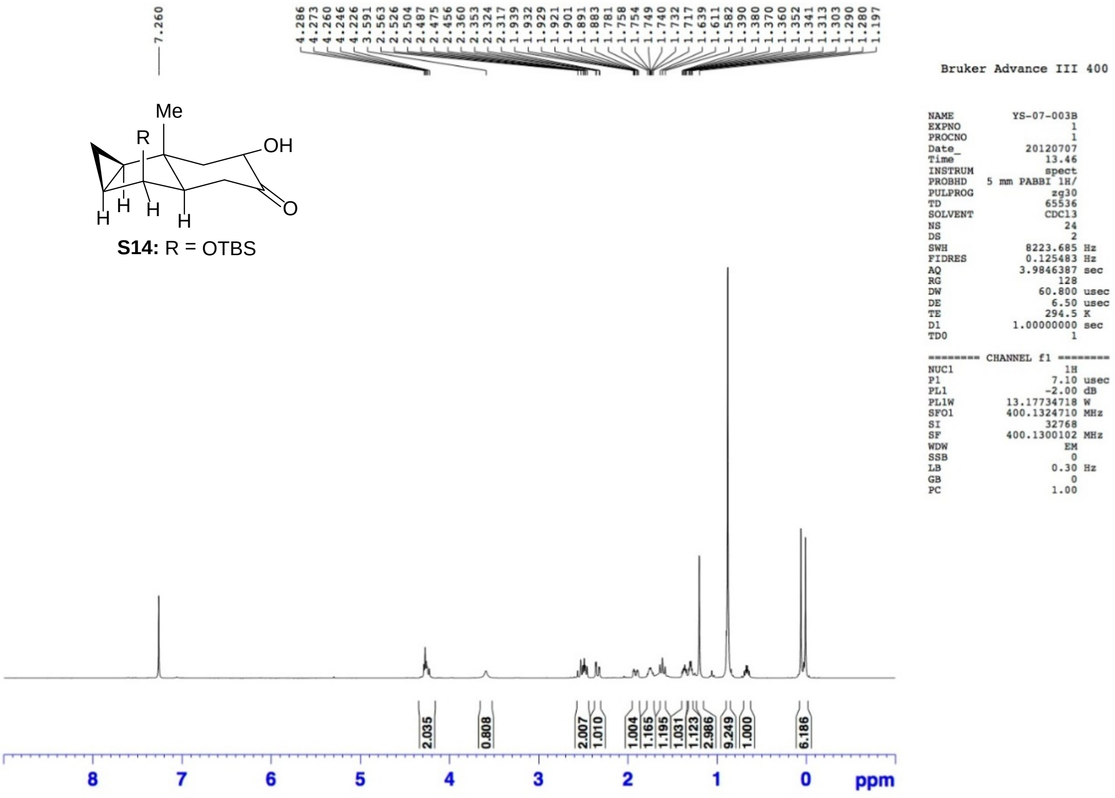

Supplementary Figure 59 ^{1}H NMR spectrum of Compound **S14** in $CDCl_3$

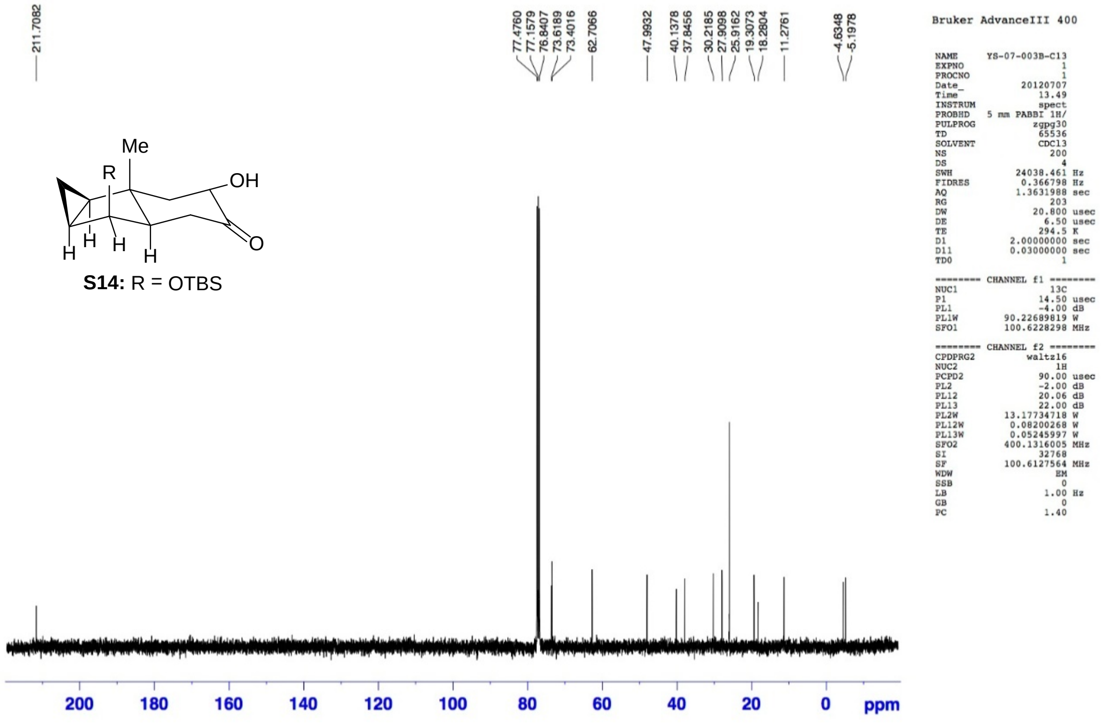

Supplementary Figure 60 ^{13}C NMR spectrum of Compound **S14** in $CDCl_3$

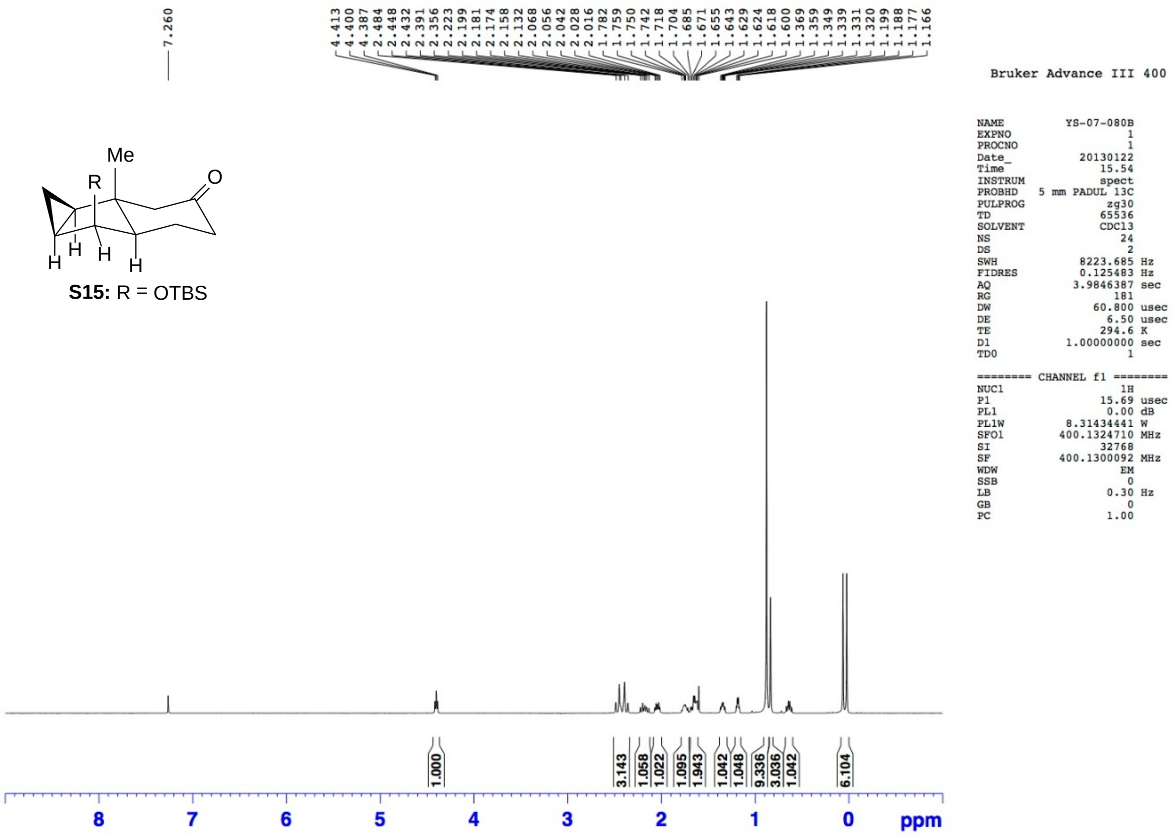

Supplementary Figure 61 ^{1}H NMR spectrum of Compound **S15** in $CDCl_3$

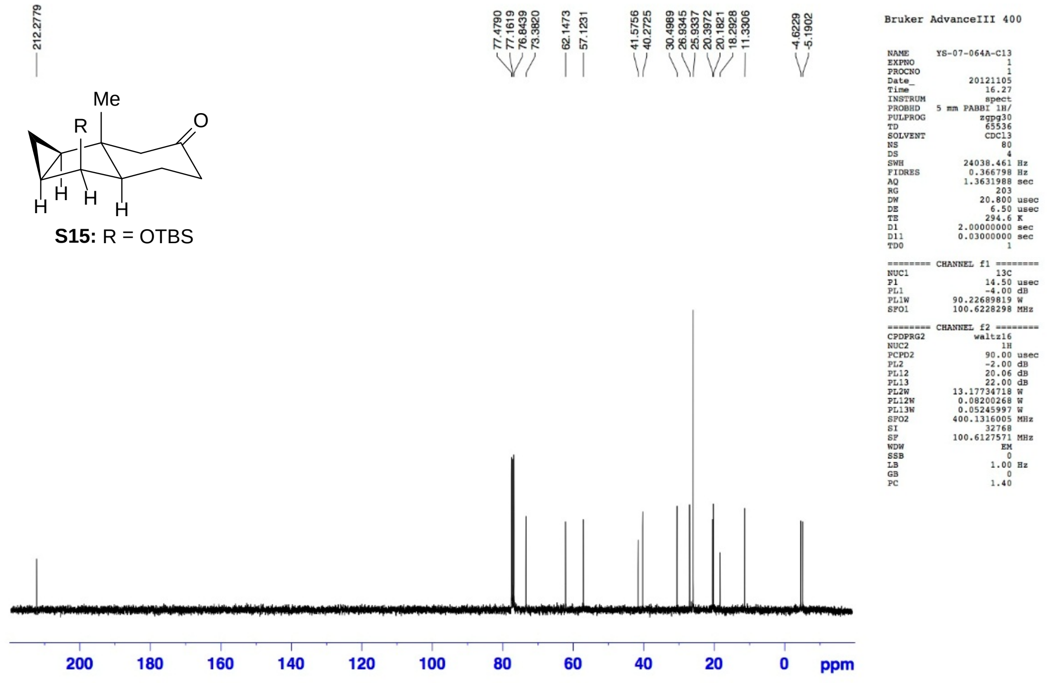

Supplementary Figure 62 ^{13}C NMR spectrum of Compound **S15** in $CDCl_3$

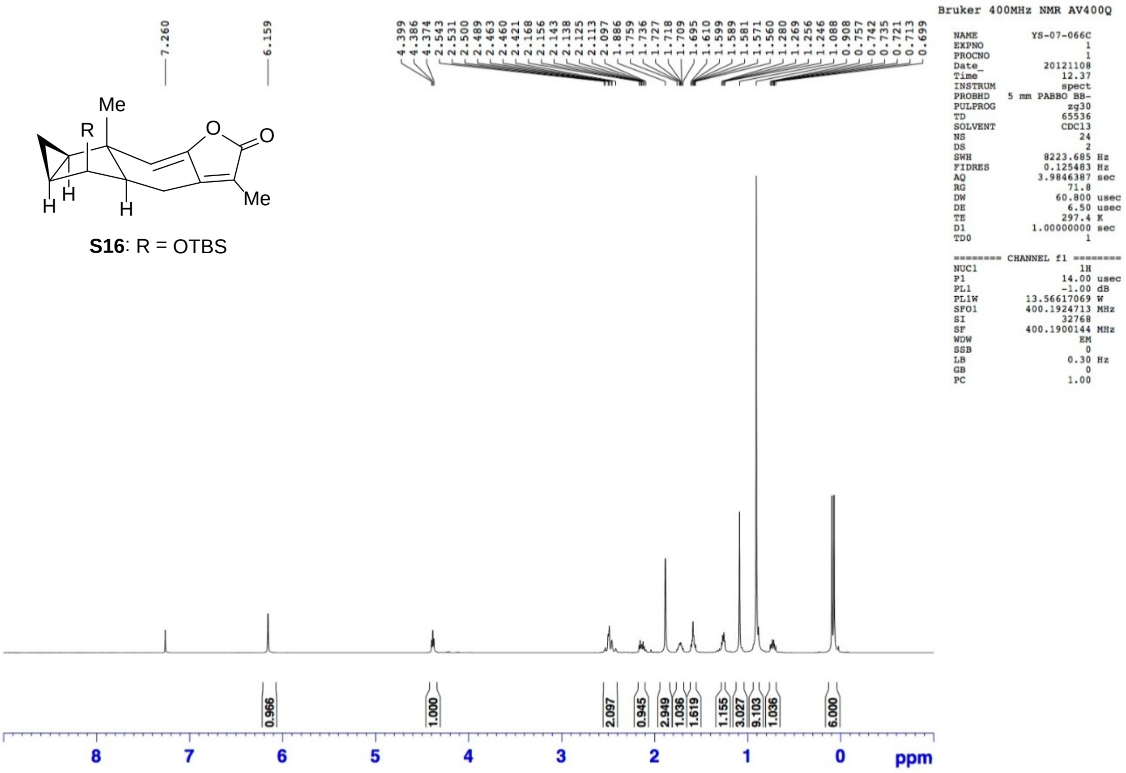

Supplementary Figure 63 ^{1}H NMR spectrum of Compound **S16** in $CDCl_3$

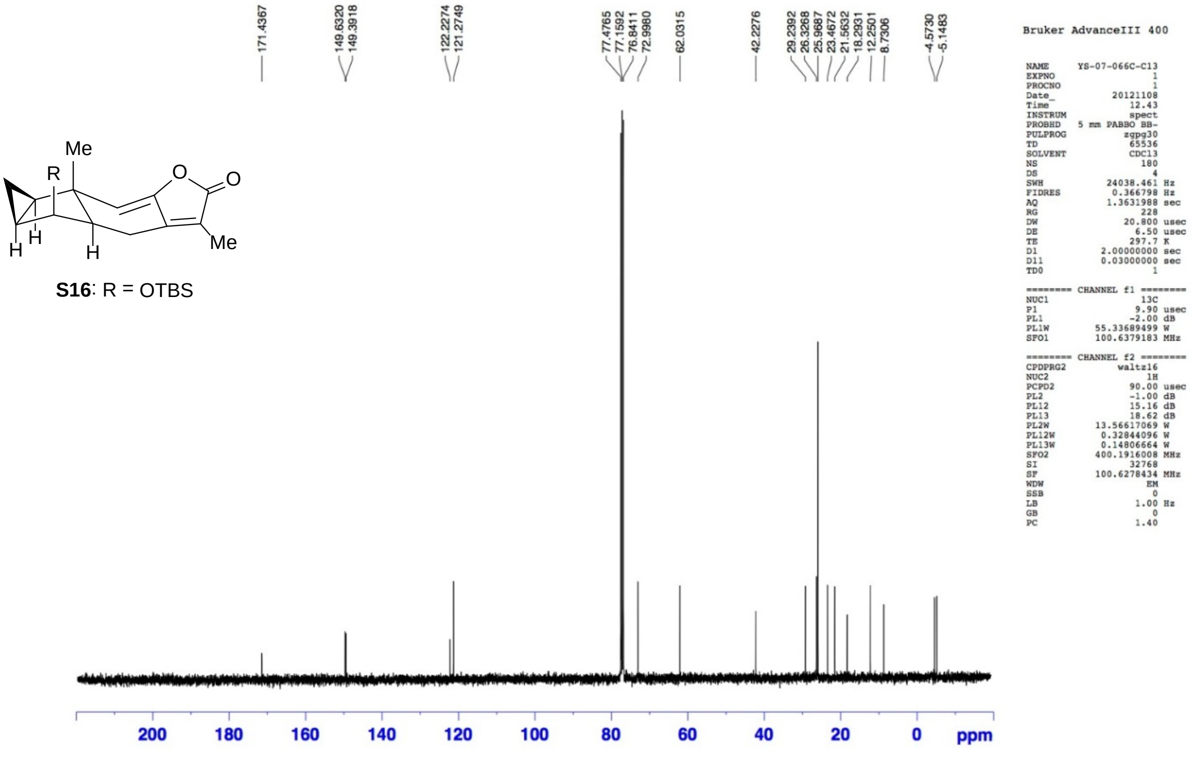

Supplementary Figure 64 ^{13}C NMR spectrum of Compound **S16** in $CDCl_3$

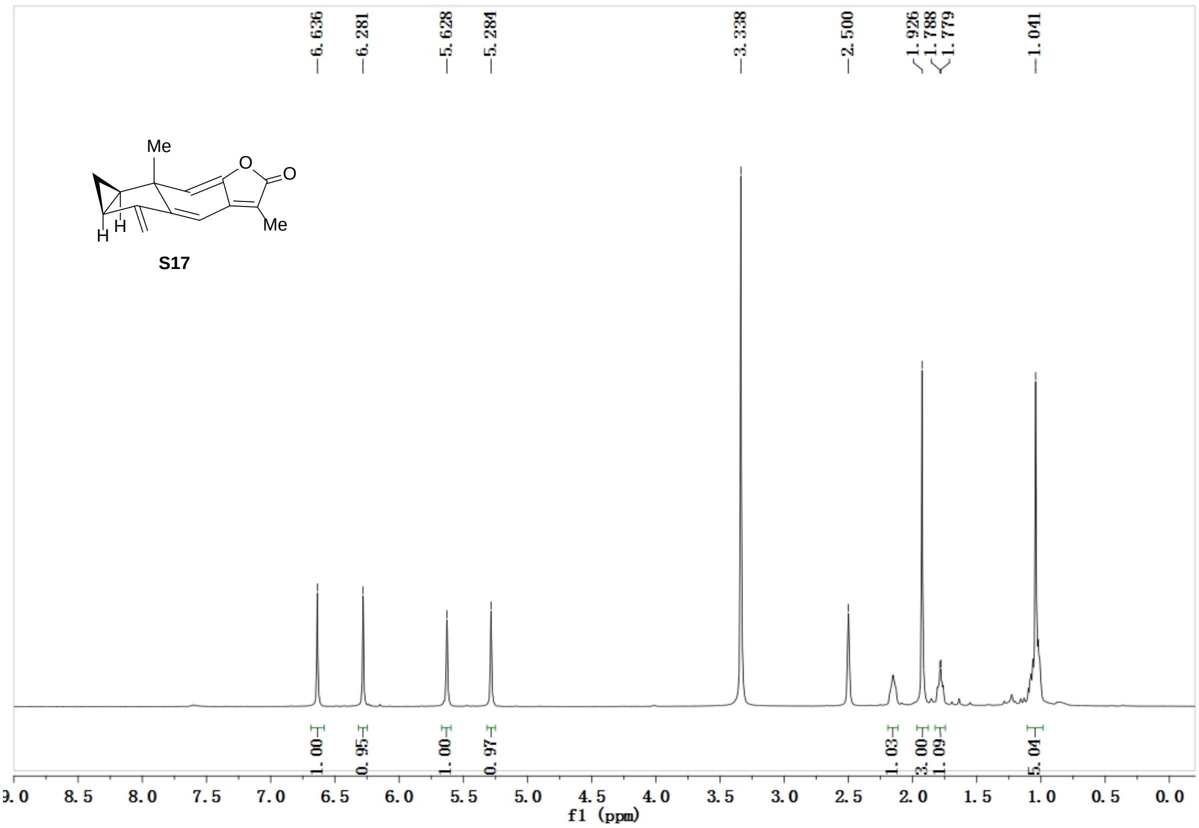

Supplementary Figure 65 ^{1}H NMR spectrum of Compound **S17** in DMSO-d6

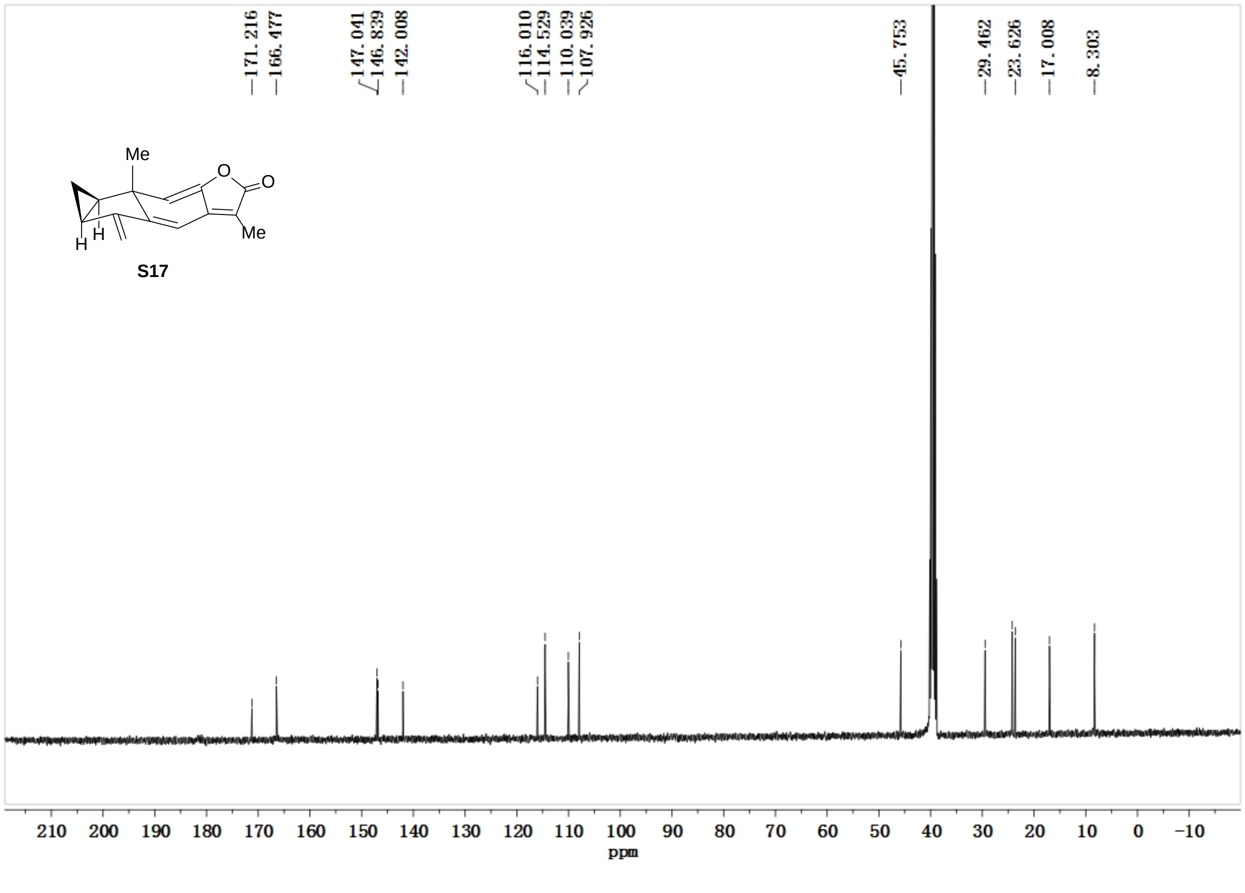

Supplementary Figure 66 ^{13}C NMR spectrum of Compound **S17** in DMSO-d6

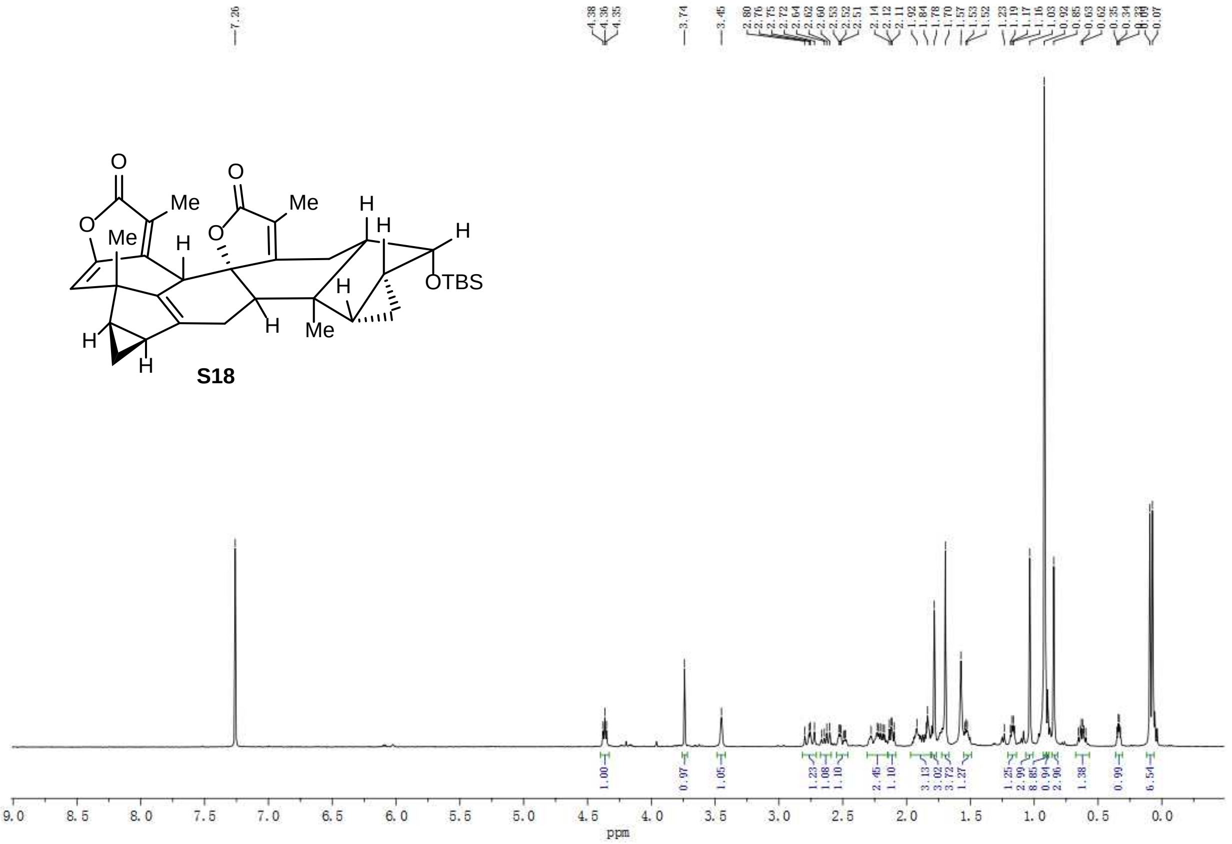

Supplementary Figure 67 ^{1}H NMR spectrum of Compound **S18** in $CDCl_3$

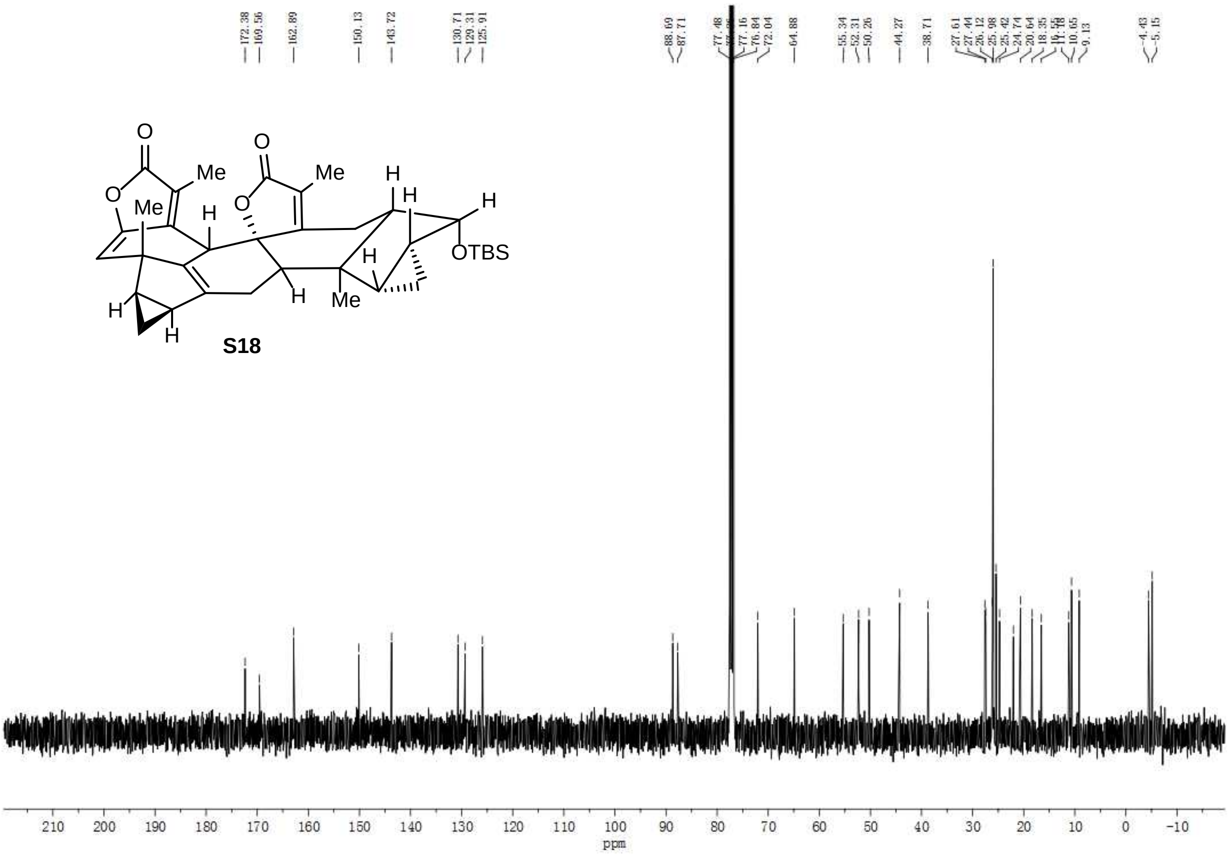

Supplementary Figure 68 ^{13}C NMR spectrum of Compound **S18** in $CDCl_3$

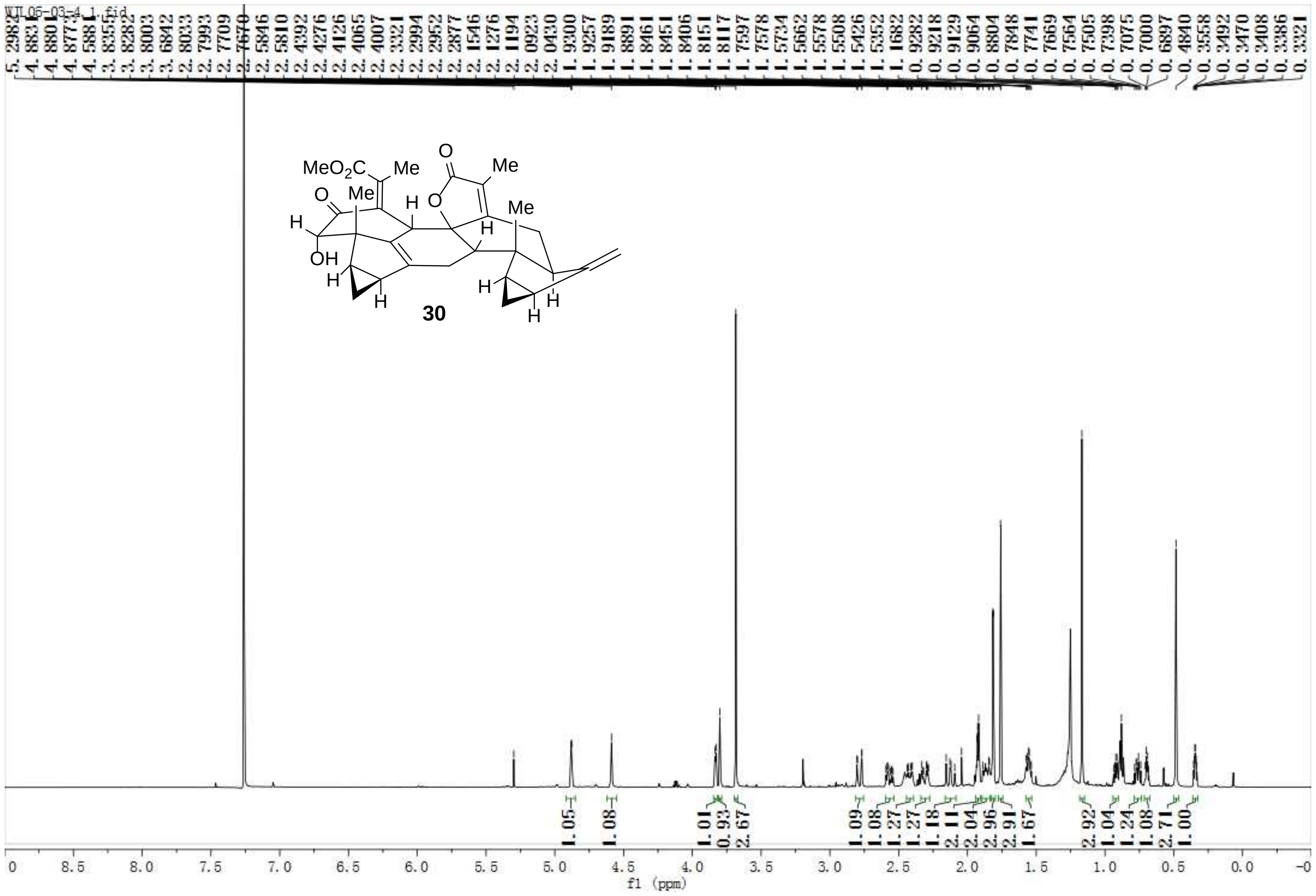

Supplementary Figure 69 ^{1}H NMR spectrum of Compound **30** in $CDCl_3$

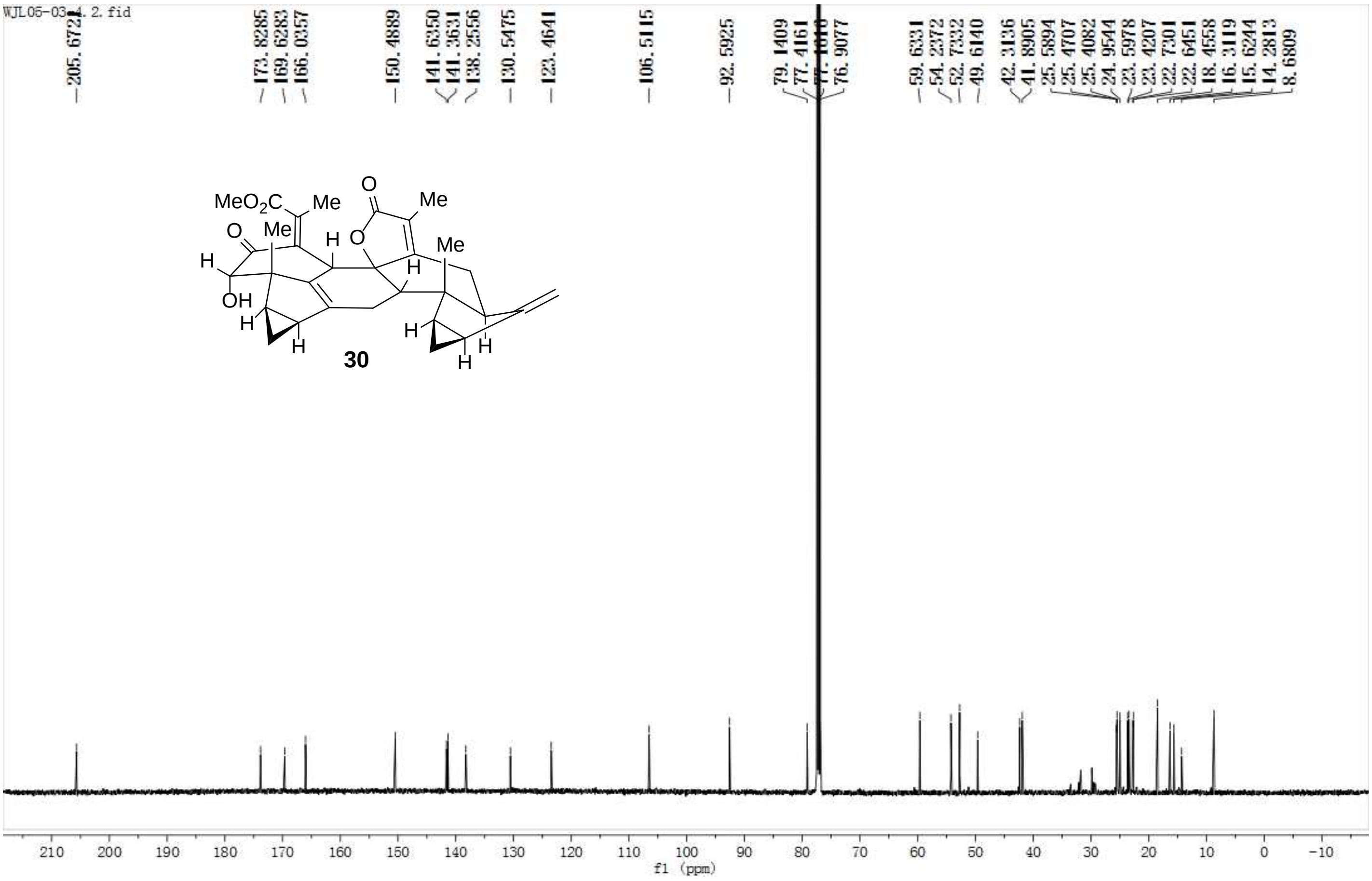

Supplementary Figure 70 ^{13}C NMR spectrum of Compound **30** in $CDCl_3$

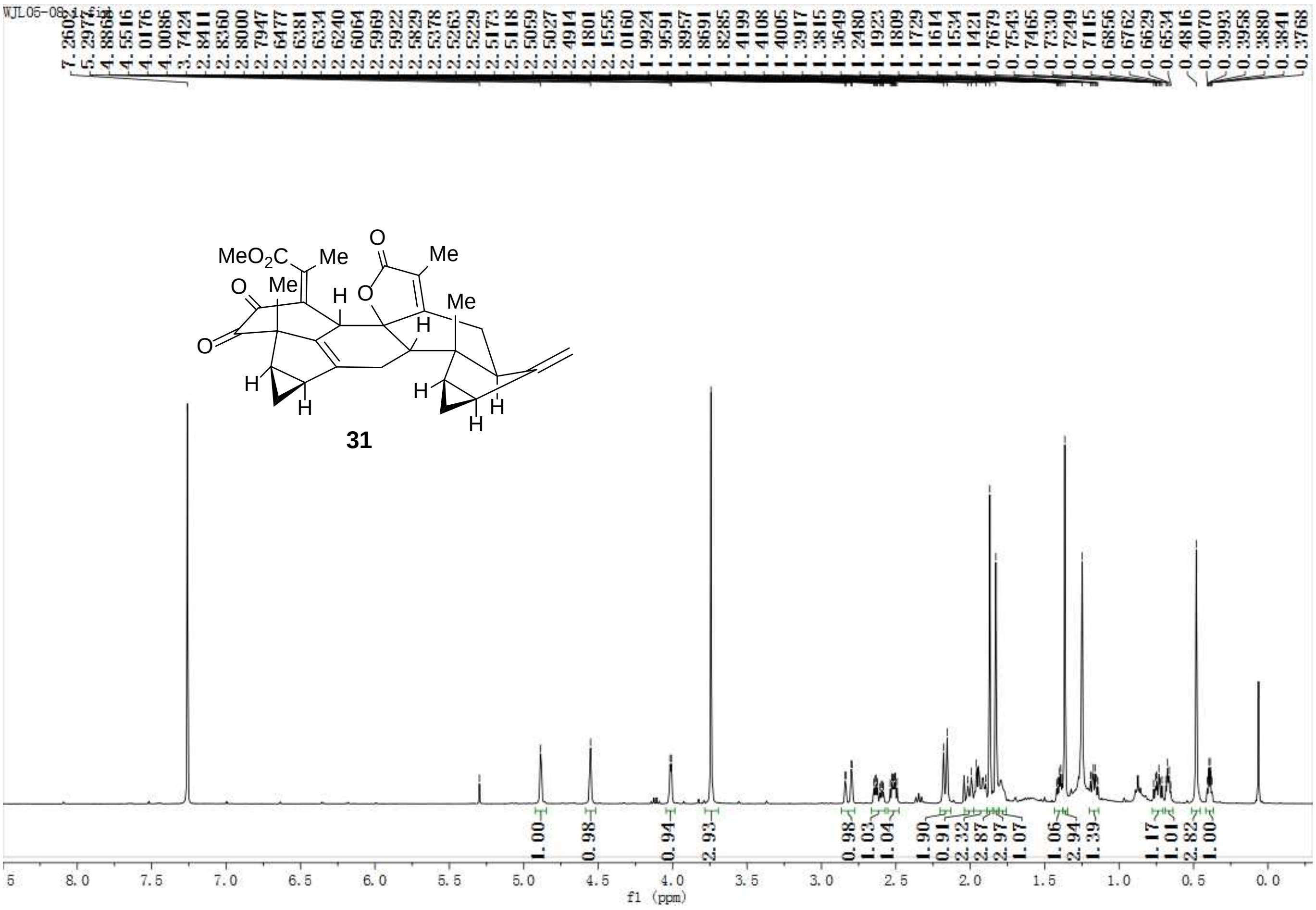

Supplementary Figure 71 ^{1}H NMR spectrum of Compound **31** in $CDCl_3$

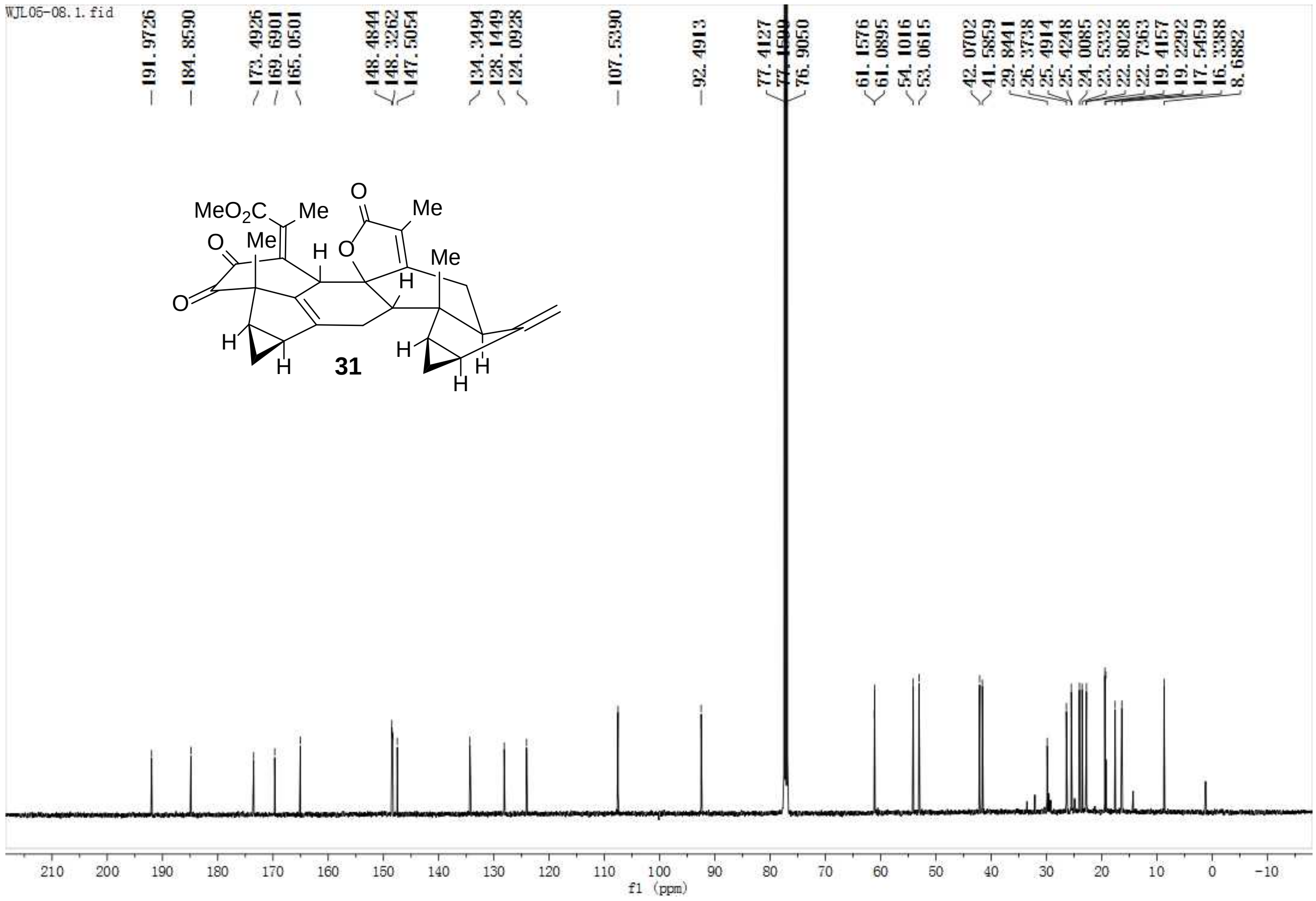

Supplementary Figure 72 ^{13}C NMR spectrum of Compound **31** in $CDCl_3$

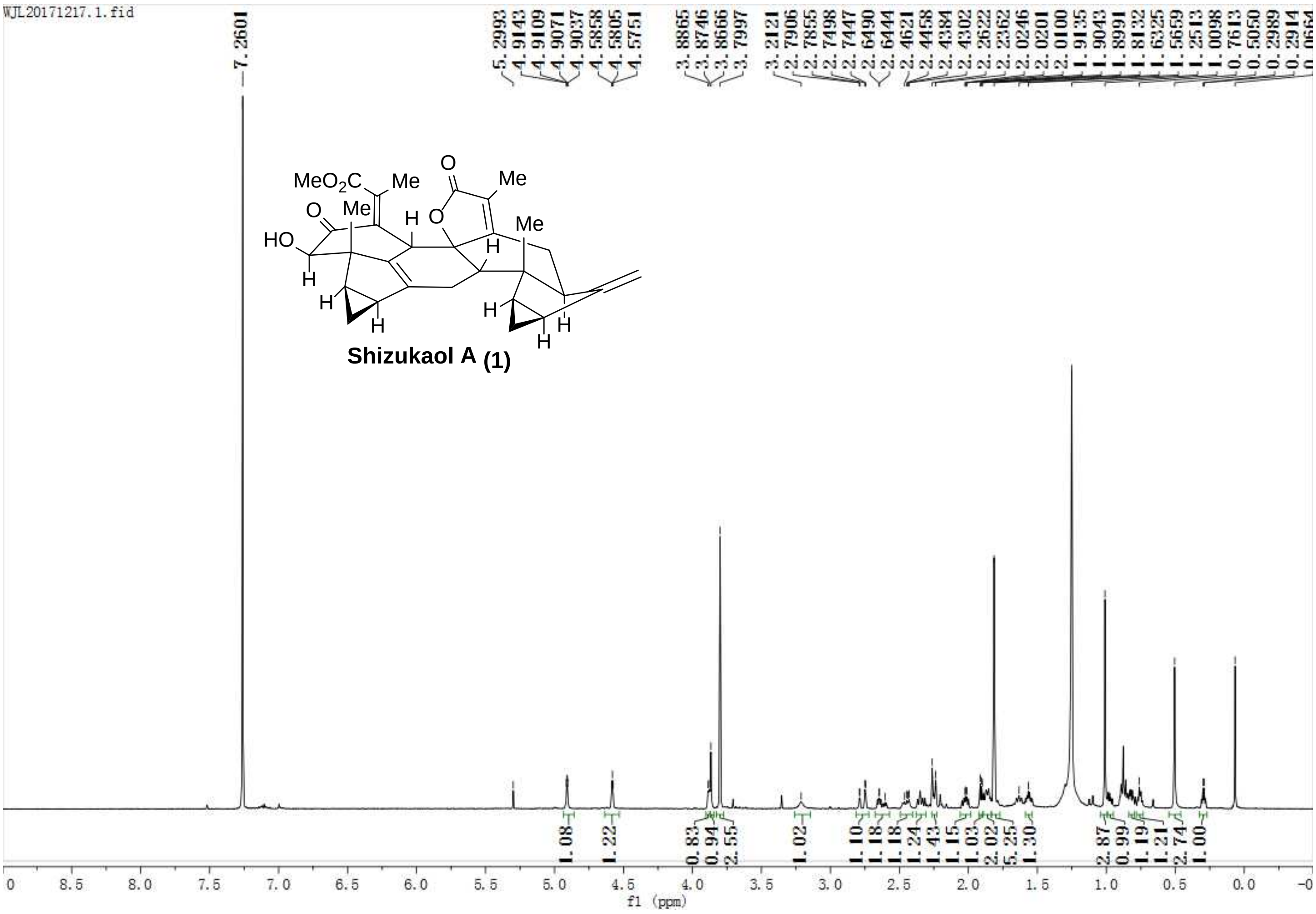

Supplementary Figure 73 ^{1}H NMR spectrum of shizukaol A (**1**) in $CDCl_3$

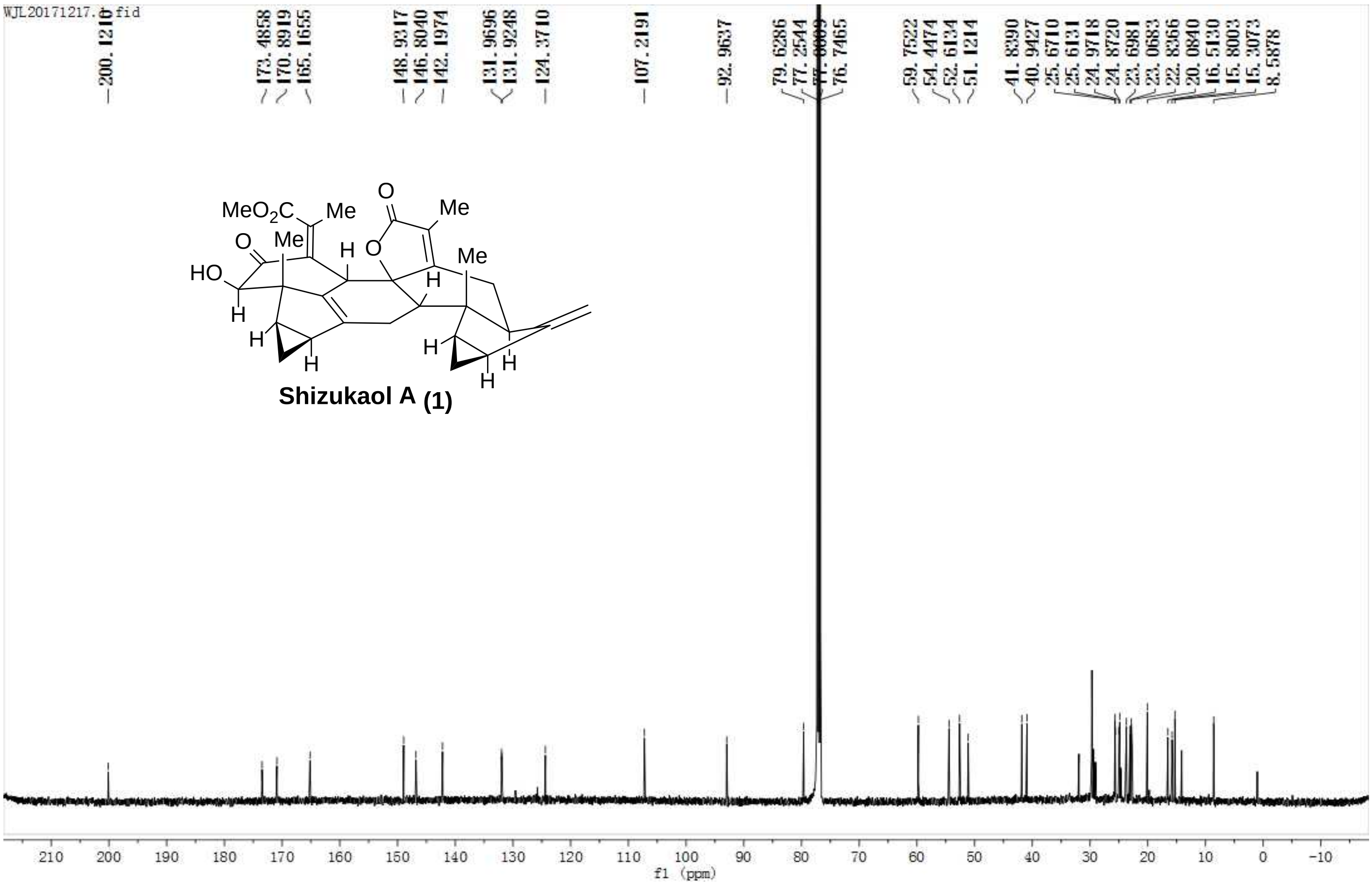

Supplementary Figure 74 ^{13}C NMR spectrum of shizukaol A (**1**) in $CDCl_3$

Supplementary Methods

General Information

All reagents and solvents were reagent grade. Further purification and drying following the guidelines of Perrin and Armarego were used when necessary. Organic solvents were concentrated under reduced pressure on a rotary evaporator in a water bath no more than 40 ℃ unless otherwise specified. Thin-layer chromatography (TLC) was performed on E. Merck silica gel 60 F254 (0.25 mm thickness) coated on aluminum plates. Chromatographic purification of products was performed on Macherey Nagel Kieselgel 60 M (230 – 400 mesh). Visualization of the developed chromatogram was performed by acidic ceric ammonium molybdate and subsequent heating.

Melting points were measured with a Stuart Melting Point Apparatus (SMP40) in Celsius degrees and were uncorrected. Nuclear magnetic resonance (NMR) spectra were recorded with a Bruker ADVANCE-III NMR spectrometer at 400.13 MHz (^{1}H) or at 100.6 MHz (^{13}C). All NMR measurements were carried out in $CDCl_3$ and internally referenced to residual solvent signals (referenced at δ 7.26 ppm in ^{1}H, and δ 77.16 ppm for central line of the triplet in ^{13}C). Data for ^{1}H NMR are reported as follows: chemical shift (δ ppm), multiplicity (s = singlet, d = doublet, t = triplet, q = quartet, brs = broad singlet, dd = doublet of doublets, dt = doublet of triplets, td = triplet of doublets, ddd = doublet of doublets of doublets, m = multiplet), integration, coupling constant (Hz) and assignment. Data for ^{13}C NMR are reported in terms of chemical shift. Mass spectrometry (MS) and high-resolution mass spectrometry (HRMS) were measured on a ThermoFinnigan MAT 95XL. Elemental analyses were carried out by Shanghai Institute of Organic Chemistry, the Chinese Academy of Science, PRC. Selected crystals for X-ray analyses were used for intensity data collection on either a Bruker AXS Kappa Apex II Duo diffractometer or Bruker D8 Venture X-Ray Diffractometer at 173K using frames of oscillation range 0.3°, with 2° < θ < 28°. Infrared spectra (IR) were recorded on a Nicolet 420 FT-IR spectrometer as thin film on potassium bromide discs.

Experimental procedures and characterization data

Procedure for the preparation of (±)-(1a*R*,1b*S*,6*S*,6a*S*)-6-((*tert*-butyldimethylsilyl)oxy)-1b-methyl-1,1a,2,3,6,6a-hexahydrocyclopropa[a]inden-4(1b*H*)-one (S2):

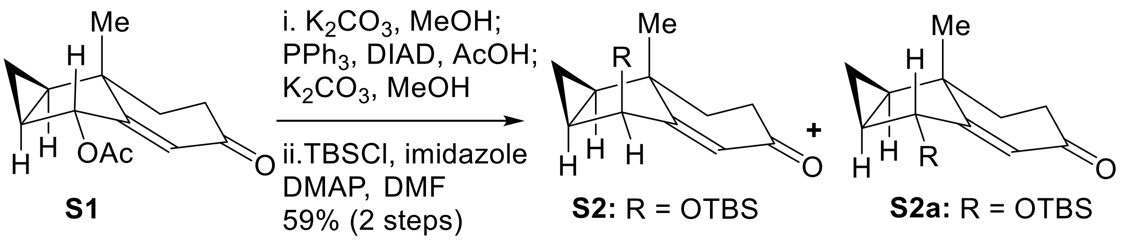

To a solution of compound **S1** (237.0 mg, 1.08 mmol, 1.00 equiv.) in MeOH (16.0 mL) was added K_2CO_3 (223.0 mg, 1.62 mmol, 1.50 equiv.). The mixture was stirred at this temperature for 1 h. After evaporation of MeOH *in vacuo*, the residue was diluted with EtOAc (200 mL), washed with water (40 mL × 2), and then brine (40 mL). The organic layer was dried over anhydrous Na_2SO_4, filtered and concentrated for next step without further purification.

To a solution of the crude product and PPh_3 (594.0 mg, 2.27 mmol, 2.10 equiv.) in THF (18.0 mL) was added AcOH (133 μL, 2.27 mmol, 2.10 equiv.) and DIAD (577 μL, 2.92 mmol, 2.70 equiv.) at 0 ^{o}C under Argon atmosphere. The mixture was stirred at this temperature for about 3 h and saturated aq. NH_4Cl was added. After evaporation of THF *in vacuo*, the residue was diluted with EtOAc (200 mL), washed with water (40 mL × 2), and then brine (40 mL). The organic layer was dried over anhydrous Na_2SO_4, filtered and concentrated for next step without further purification.

To a solution of the crude product above in MeOH (16.0 mL) was added K_2CO_3 (223.0 mg, 1.62 mmol, 1.50 equiv.) at room temperature. The mixture was stirred at this temperature for 1 h. After evaporation of MeOH *in vacuo*, the residue was diluted with EtOAc (200 mL), washed with water (40 mL × 2), and then brine (40 mL). The organic layer was dried over anhydrous Na_2SO_4, filtered and concentrated for next step without further purification. Then, to a solution of this crude product and TBSCl (324.0 mg, 2.16 mmol, 2.00 equiv.), and imidazole (220.0 mg, 3.24 mmol, 3.00 equiv.) in DMF (1.5 mL) was added DMAP (40.0 mg, 0.32 mmol, 0.30 equiv.). The mixture was stirred at 20 ^{o}C for about 1 h. The reaction was diluted EtOAc (100 mL), washed with water (10 mL × 2), and then brine (10 mL). The organic layer was dried over anhydrous Na_2SO_4, filtered and concentrated. The residue was purified by column chromatography on silica gel (hexane/EtOAc 10:1) to give compound **S2** (184.0 mg, 59%) as a white solid and its diastereomer **S2a** (22.0 mg, 7%) as a colorless oil.

S2: R_f = 0.70 (hexane/EtOAc 4:1); m.p.: 60.1 – 61.2 ^{o}C; IR (film): ν = 2953, 2929, 1672, 1663, 1081, 1034 cm^{-1}; ^{1}H NMR (400 MHz, $CDCl_3$) δ 5.56 (s, 1H), 4.75 (d, J = 6.0 Hz, 1H), 2.55 (ddd, J = 5.2, 12.8, 18.4 Hz, 1H), 2.35 (dd, J = 4.0, 18.4 Hz, 1H), 2.06 (ddd, J = 1.6, 5.2, 12.8 Hz, 1H), 1.97 (td, J = 4.8, 13.6 Hz, 1H), 1.82 – 1.75 (m, 1H), 1.43 – 1.38 (m, 2H), 1.18 (s, 3H), 0.88 (s, 9H), 0.75 (ddd, J = 2.4,

9.2, 18.4 Hz, 1H), 0.10 (s, 3H), 0.04 ppm (s, 3H); ^{13}C NMR (100 MHz, $CDCl_3$) δ 200.6, 177.9, 119.5, 75.4, 39.9, 37.8, 34.1, 30.9, 25.9, 24.6, 21.4, 18.2, 10.9, – 4.6, – 4.6 ppm; HRMS (ESI) *m/z* calcd for $C_{17}H_{28}O_2SiNa$ $[M+Na]^+$ 315.1751, found 315.1765.

S2a: R_f = 0.65 (hexane/EtOAc 4:1); IR (film): ν = 2954, 2930, 1675, 1258, 1137 cm^{-1}; ^{1}H NMR (400 MHz, $CDCl_3$) δ 5.70 (s, 1H), 4.30 (s, 1H), 2.57 (ddd, *J* = 5.2, 13.2, 18.4 Hz, 1H), 2.41 (dd, *J* = 4.8, 18.0 Hz, 1H), 2.19 (td, *J* = 5.2, 13.2 Hz, 1H), 2.09 (ddd, *J* = 1.6, 5.6, 13.2 Hz, 1H), 1.50 (td, *J* = 4.0, 7.6 Hz, 1H), 1.33 (td, *J* = 4.4, 8.4 Hz, 1H), 1.07 (s, 3H), 0.98 (td, *J* = 6.0, 8.8 Hz, 1H), 0.94 (s, 9H), 0.70 (dd, *J* = 4.0, 9.6 Hz, 1H), 0.13 (s, 3H), 0.13 ppm (s, 3H); ^{13}C NMR (100 MHz, $CDCl_3$) δ 199.5, 182.8, 117.8, 76.2, 38.7, 37.7, 34.4, 29.0, 26.0, 24.3, 20.9, 18.5, 15.2, – 4.8, – 4.9 ppm; HRMS (ESI) *m/z* calcd for $C_{17}H_{28}O_2SiNa$ $[M+Na]^+$ 315.1751, found 315.1745.

Procedure for the preparation of (±)-(1a*R*,1b*S*,5a*R*,6*S*,6a*S*)-6-((*tert*-butyldimethylsilyl)oxy)-1b-methyloctahydrocyclopropa[a]inden-4(1b*H*)-one (S3):

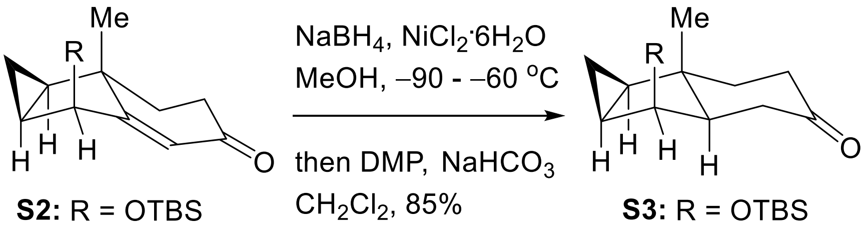

To a solution of compound **S2** (15.7 mg, 0.054 mmol, 1.00 equiv.) and $NiCl_2$•$6H_2O$ (76.8 mg, 0.32 mmol, 5.93 equiv.) in MeOH (2.0 mL) was added $NaBH_4$ (36.6 mg, 0.97 mmol, 18.0 equiv.) at –90 °C. The reaction mixture was warmed up to –60 °C slowly, and silica gel was added to the reaction mixture, which was then filtered and concentrated under reduced pressure. To a solution of this crude product and $NaHCO_3$ (67.8 mg, 0.81 mmol, 15.0 equiv.) in dry CH_2Cl_2 (2.0 mL) was added Dess-Martin periodinane (34.2 mg, 0.081 mmol, 1.50 equiv.) at 0 °C under Argon atmosphere. The reaction was kept stirring at 20 °C for about 0.5 h, cooled to 0 °C, and quenched by saturated aq. $Na_2S_2O_3$ (5 mL). The mixture was diluted with CH_2Cl_2 (50 mL), washed with water (10 mL × 2) and then brine (10 mL). The organic layer was dried over Na_2SO_4, filtered and concentrated. The residue was purified by column chromatography on silica gel (hexane/EtOAc 15:1) to give compound **S3** (13.3 mg, 85%) as a colorless oil.

S3: R_f = 0.40 (hexane/EtOAc 8:1); ^{1}H NMR (400 MHz, $CDCl_3$) δ 4.28 (t, *J* = 5.2 Hz, 1H), 2.43 – 2.32 (m, 3H), 2.15 (dd, *J* = 3.6, 16.0 Hz, 1H), 1.99 (dt, *J* = 4.0, 14.8 Hz, 1H), 1.92 – 1.79 (m, 2H), 1.78

– 1.71 (m, 1H), 1.33 (td, J = 4.0, 8.0 Hz, 1H), 1.27 (dd, J = 4.0, 9.2 Hz, 1H), 1.05 (s, 3H), 0.88 (s, 9H), 0.65 (td, J = 5.2, 8.8 Hz, 1H), 0.06 (s, 3H), 0.00 ppm (s, 3H); ^{13}C NMR (100 MHz, $CDCl_3$) δ 212.7, 73.6, 60.7, 39.5, 39.2, 38.0, 37.9, 30.7, 27.5, 26.0, 18.3, 18.2, 11.4, – 4.6, – 5.2 ppm.

Procedure for the preparation of (±)-(1a*R*,1b*S*,5a*S*,6a*S*)-1b-methyl-6-methyleneoctahydro-1*H*-spiro[cyclopropa[a]indene-4,2'-[1,3]dioxolane] (12):

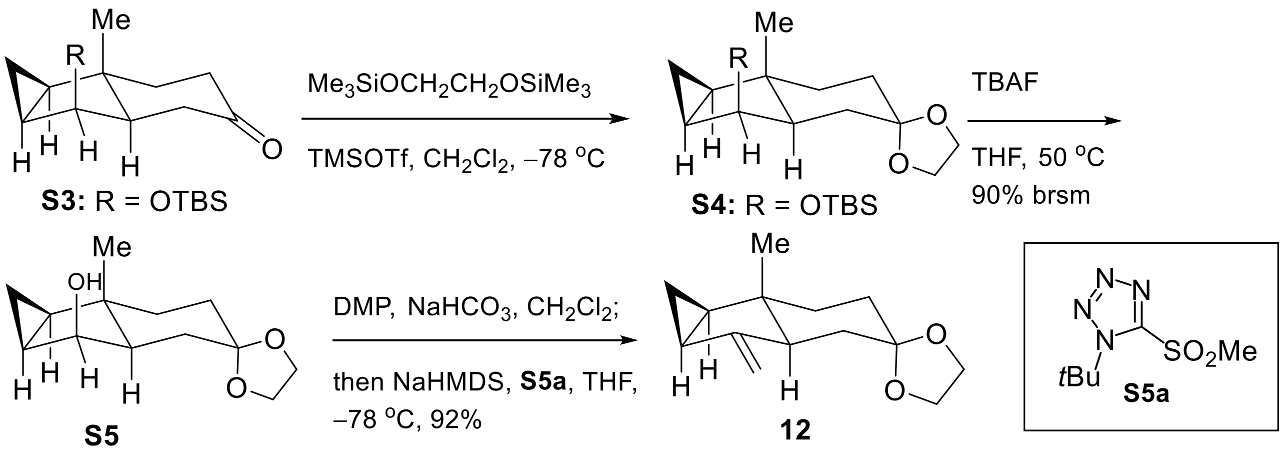

To a solution of compound **S3** (3.92 g, 13.3 mmol, 1.00 equiv.) in anhydrous CH_2Cl_2 (67 mL) was added TMSOTf (240 µL, 1.33 mmol, 0.10 equiv.) and $(TMSOCH_2)_2$ (3.92 mL, 16.0 mmol, 1.20 equiv.) at –78 °C under argon atmosphere. The reaction was stirred at this temperature for 40 min before triethylamine (1.5 mL) was added. The mixture was diluted with CH_2Cl_2 (200 mL) and washed with aqueous $NaHCO_3$ (80 mL) and then brine (80 mL). The organic layer was dried over anhydrous Na_2SO_4, filtered and concentrated. The residue was purified by column chromatography on silica gel (200 g, hexane/EtOAc 15:1) to give compound **S4** (4.79 g, quant.) as a colorless oil.

S4: R_f = 0.70 (hexane/EtOAc 8:1); IR (film): v = 2939, 1454, 1375, 1249, 1043, 837, 774 cm^{-1}; ^{1}H NMR (400 MHz, $CDCl_3$) δ 4.26 (t, J = 5.1 Hz, 1H), 3.94 (s, 4H), 1.84 (ddd, J = 15.4, 13.6, 8.3 Hz, 2H), 1.70 – 1.55 (m, 5H), 1.45 (dt, J = 12.8, 2.2 Hz, 1H), 1.27 – 1.20 (m, 2H), 0.94 (s, 3H), 0.86 (s, 9H), 0.62 – 0.53 (m, 1H), 0.01 ppm (d, J = 18.4 Hz, 6H); ^{13}C NMR (100 MHz, $CDCl_3$) δ 110.6, 74.4, 64.6, 64.2, 60.7, 39.2, 37.4, 32.9, 32.3, 30.9, 27.1, 26.0, 18.3, 17.8, 11.8, – 4.6, – 5.1 ppm; HRMS (ESI) m/z calcd for $C_{19}H_{34}O_3Si$ [M+H]$^+$ 339.2350, found 339.2344; Anal. Calcd for $C_{19}H_{34}O_3Si$: C, 67.41; H, 10.12; found: C, 67.29; H, 10.10.

To a solution of compound **S4** obtained above in THF (47 mL) was added TBAF (20 mL, 1.0 M in

THF, 20 mmol, 1.50 equiv.) and the reaction was stirred at 50 ℃ for 24 h. THF was evaporated and the residue was diluted with EtOAc (500 mL). The organic layer was washed with water (100 mL × 2), brine (100 mL), then dried over Na_2SO_4, filtered and concentrated. The residue was purified by column chromatography on silica gel (60 g, hexane/EtOAc 3:2) to afford compound **S5** (2.51 g, 84% for 2 steps, 90% brsm) as a colorless oil after recovering compound **S4** (290 mg, 0.86 mmol).

S5: R_f = 0.50 (hexane/EtOAc 1:1); IR (film): v = 3462, 2926, 1443, 1349, 1292, 1245, 1080, 773, 496 cm^{-1}; ^1H NMR (400 MHz, $CDCl_3$) δ 4.38 (t, J = 4.5 Hz, 1H), 3.94 (d, J = 4.2 Hz, 4H), 1.96 (ddd, J = 13.0, 4.7, 3.3 Hz, 1H), 1.91–1.83 (m, 1H), 1.76 – 1.71 (m, 1H), 1.70 – 1.67(m, 1H), 1.65 – 1.55 (m, 5H), 1.31 (ddd, J = 9.3, 7.9, 3.8 Hz, 2H), 1.06 (d, J = 1.6 Hz, 1H), 0.98 (s, 3H), 0.65 ppm (td, J = 8.5, 5.4 Hz, 1H); ^{13}C NMR (100 MHz, $CDCl_3$) δ 110.2, 74.2, 64.5, 64.2 60.3, 39.2, 37.3, 32.4, 32.3, 30.8, 26.7 18.1, 11.1 ppm; HRMS (ESI) *m/z* calcd for $C_{13}H_{20}O_3$ $[M+H]^+$ 225.1485, found 225.1483; Anal. Calcd for $C_{13}H_{20}O_3$: C, 69.61; H, 8.99; found: C, 69.12; H, 8.97.

To a solution of compound **S5** (1.63 g, 7.27 mmol, 1.00 equiv.) and $NaHCO_3$ (2.44 g, 29.1 mmol, 4.00 equiv.) in CH_2Cl_2 (73 mL) was added Dess-Martin periodinane (4.0 g, 9.45 mmol, 1.30 equiv.). The reaction was stirred at room temperature for 20 min and quenched with saturated aqueous $Na_2S_2O_3$ (20 mL). The mixture was diluted with CH_2Cl_2 (200 mL) and washed with water (100 mL × 2), brine (100 mL), then dried over Na_2SO_4, filtered and evaporated to dryness. As the ketone is unstable, it was used in the next step without purification.

To a solution of *J-K* reagent **S5a** (3.57 g, 17.5 mmol, 2.41 equiv.) in anhydrous THF (120 mL) under Argon atmosphere was added NaHMDS (8.0 mL, 2 M in THF, 16.0 mmol, 2.20 equiv.) at –78 ℃. After addition, the solution was stirred for 30 min at this temperature. Then a solution of crude ketone obtained above in THF (20 mL) was added slowly. After addition, the reaction was stirred for 100 min and saturated aq. NH_4Cl (30 mL) was added. The mixture was extracted with CH_2Cl_2 (100 mL × 2), washed with water (100 mL) and brine (100 mL). The organic layer was dried over Na_2SO_4, filtered and concentrated. The residue was purified by column chromatography on silica gel (80 g, hexane/EtOAc 14:1) to give compound **12** (1.48 g, 92% for 2 steps) as a colorless oil.

12: R_f = 0.75 (hexane/EtOAc 10:1); IR (film): v = 3072, 2943, 1661, 1449, 1354, 1284, 1100, 1043, 949, 883, 485 cm^{-1}; ^1H NMR (400 MHz, $CDCl_3$) δ 4.87 (t, J = 1.2 Hz, 1H), 4.56 (s, 1H), 3.94 (s, 4H), 2.75 (dd, J = 13.1, 2.4 Hz, 1H), 1.94 – 1.82 (m, 2H), 1.79 – 1.67 (m, 3H), 1.62 (dd, J = 8.7, 6.4 Hz, 1H), 1.39 (d, J = 13.0 Hz, 1H), 1.33 (dd, J = 7.4, 3.2 Hz, 1H), 0.89 – 0.81 (m, 1H), 0.81 – 0.72 (m, 1H), 0.62 ppm (s, 3H); ^{13}C NMR (100 MHz, $CDCl_3$) δ 153.0, 109.8, 104.3, 64.5, 64.3, 61.3, 37.6, 35.5, 32.6, 32.2, 28.1,

23.6, 16.7, 16.3 ppm; HRMS (ESI) *m/z* calcd for $C_{14}H_{20}O_2$ $[M+H]^+$ 221.15361, found 221.15360; Anal. Calcd for $C_{14}H_{20}O_2$: C, 76.33; H, 9.15; found: C, 76.10; H, 9.21.

Procedure for the preparation of (±)-(1a*R*,1b*S*,5a*S*,6*R*,6a*S*)-6-(hydroxymethyl)-1b-methyloctahydrocyclopropa[a]inden-4(1b*H*)-one (13):[1]

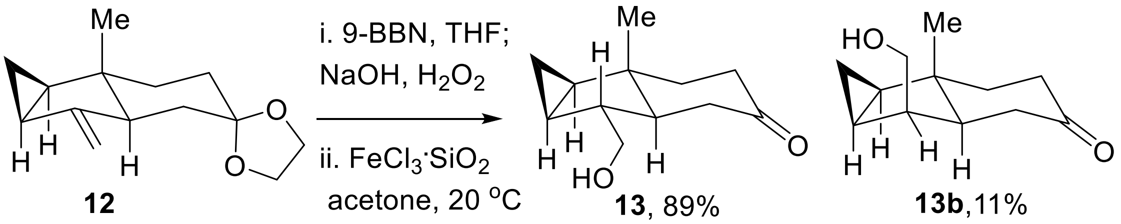

To a solution of compound **12** (5.80 g, 26.3 mmol, 1.00 equiv.) in THF (250 mL) was added 9-BBN (105.3 mL, 0.5 M in THF, 52.7 mmol, 2.00 equiv.) slowly through dropping funnel at 0 ℃. After addition, the reaction was warmed up to room temperature and stirred overnight. The solution was cooled down again using ice bath. Aqueous solution NaOH (43 mL, 3 M, 129 mmol, 4.90 equiv.) and H_2O_2 (36.8 mL, 35%) was added slowly successively. Then the ice bath was removed and the mixture was stirred for about 40 min. The reaction mixture was extracted with CH_2Cl_2 (100 mL × 2), washed with water (100 mL) and brine (100 mL). The organic layer was dried over Na_2SO_4, filtered and concentrated. The residue was purified by column chromatography on silica gel (150 g, hexane/EtOAc 3:2) to give the inseparable isomers as a colorless oil: R_f = 0.40 (hexane/EtOAc 1:1), which were dissolved in acetone (130 mL) and $FeCl_3{\cdot}SiO_2$ (2.63 g) was added in one portion. The reaction was stirred at 20 ℃ for 3 h before the solvent was evaporated. The residue was purified by column chromatography on silica gel (150 g, hexane/EtOAc 1:1) to give compound **13** (4.56 g, 89%) as a colorless oil, and compound **13b** (560 mg, 11%) as a colorless oil.

13: R_f = 0.35 (hexane/EtOAc 1:1); IR (film): ν = 3407, 2926, 1699, 1417, 1055 cm^{-1}; ^1H NMR (400 MHz, $CDCl_3$) δ 3.73 (dd, *J* = 10.5, 5.0 Hz, 1H), 3.62 (dd, *J* = 10.5, 6.8 Hz, 1H), 2.53 – 2.37 (m, 2H), 2.27 – 2.07 (m, 3H), 2.02 – 1.80 (m, 3H), 1.60 – 1.50 (m, 1H), 1.35 – 1.20 (m, 2H), 0.88 (s, 3H), 0.86 – 0.73 ppm (m, 2H); ^{13}C NMR (100 MHz, $CDCl_3$) δ 211.6, 64.7, 59.0, 47.8, 40.8, 39.2, 38.2, 36.8, 28.5, 23.3, 16.6, 16.4 ppm; HRMS (ESI) *m/z* calcd for $C_{12}H_{18}O_2$ $[M+Na]^+$ 217.1199, found 217.1198; Anal. Calcd for $C_{12}H_{18}O_2$: C, 74.19; H, 9.34; found: C, 73.65; H, 9.34.

13b: R_f = 0.30 (hexane/EtOAc 1:1); IR (film): ν = 3742, 3445, 2931, 1699, 1052, 521 cm^{-1}; ^1H NMR

(400 MHz, $CDCl_3$) δ 3.54 – 3.42 (m, 2H), 2.57 – 2.46 (m, 2H), 2.43 (q, *J* = 6.7 Hz, 2H), 2.30 – 2.12 (m, 2H), 1.90 (t, *J* = 6.8 Hz, 2H), 1.68 – 1.62 (m, 1H), 1.42 – 1.36 (m, 1H), 0.95 (s, 3H), 0.93 – 0.90 (m, 1H), 0.70 ppm (td, *J* = 8.7, 6.0 Hz, 1H); ^{13}C NMR (100 MHz, $CDCl_3$) δ 211.1, 61.4, 56.9, 44.2, 39.9, 39.8, 38.4, 38.0, 29.6, 21.9, 18.8, 11.1 ppm; HRMS (ESI) *m/z* calcd for $C_{12}H_{18}O_2$ $[M+Na]^+$ 217.1199, found 217.1198.

Procedure for the preparation of (±)-((1a*R*,1b*S*,5a*S*,6*R*,6a*S*)-1b-methyl-4-oxodecahydrocyclopropa[a]inden-6-yl)methyl 4-nitrobenzoate (13a):

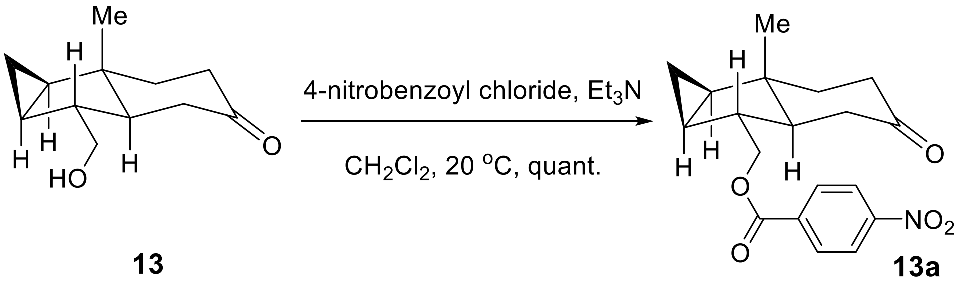

13 **13a**

To a solution of compound **13** (24.7 mg, 0.13 mmol, 1.00 equiv.) in CH_2Cl_2 (2.0 mL) was added triethylamine (70 μL, 0.51 mmol, 3.92 equiv.) and 4-nitrobenzoyl chloride (47 mg, 0.25 mmol, 1.92 equiv.) at 20 °C. The reaction was stirred at this temperature for 1 h and excess chloride was quenched with water (0.5 mL). The mixture was diluted with EtOAc (50 mL). The organic layer was washed with water (20 mL), brine (20 mL) then dried over Na_2SO_4, filtered and concentrated. The residue was purified by column chromatography on silica gel (12 g, hexane/EtOAc 6:1) to give compound **13a** (43.8 mg, quant.) as a white solid, whose single crystal was obtained from a CH_2Cl_2 solution.

13a: R_f = 0.20 (hexane/EtOAc 6:1); m.p. = 129.3–133.4 °C; IR (film): *v* = 2945, 1716, 1528, 1347, 1275, 1110, 1058, 859, 718 cm^{-1}; ^{1}H NMR (400 MHz, $CDCl_3$) δ 8.32 – 8.27 (m, 2H), 8.22 – 8.16 (m, 2H), 4.46 (dd, *J* = 11.0, 6.4 Hz, 1H), 4.38 (dd, *J* = 11.0, 6.2 Hz, 1H), 2.51 – 2.45 (m, 2H), 2.35 – 2.18 (m, 3H), 2.07 – 1.90 (m, 2H), 1.87 – 1.82 (m, 1H), 1.41 – 1.29 (m, 2H), 0.92 (s, 3H), 0.82 ppm (td, *J* = 8.4, 5.7 Hz, 1H); ^{13}C NMR (100 MHz, $CDCl_3$) δ 210.7, 164.7, 150.7, 135.6, 130.8, 123.7, 67.8, 60.1, 44.5, 40.8, 39.4, 38.0, 36.7, 28.6, 23.1, 16.6, 16.3 ppm; HRMS (ESI) *m/z* calcd for $C_{19}H_{21}O_5N$ $[M+Na]^+$ 366.13119, found 366.13119.

Procedure for the preparation of (±)-(1a*R*,1b*S*,5a*S*,6*R*,6a*S*)-6-(((*tert*-butyldimethylsilyl)-oxy)methyl)-1b-methyloctahydrocyclopropa[a]inden-4(1b*H*)-one（14）：

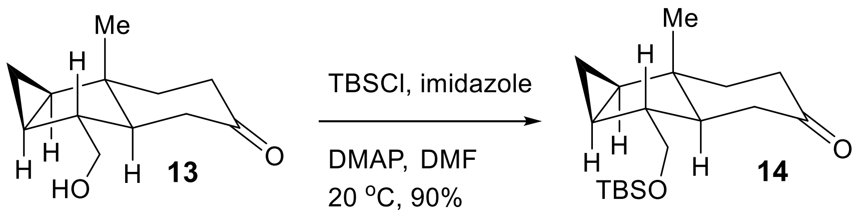

To a solution of compound **13** (4.56 g, 23.5 mmol, 1.00 equiv.), imidazole (4.79 g, 70.4 mmol, 3.00 equiv.) and DMAP (143 mg, 1.17 mmol, 0.050 equiv.) in DMF (30 mL) was added TBSCl (7.08 g, 47.0 mmol, 2.00 equiv.) under argon atmosphere. The reaction was stirred overnight at room temperature and excess reagent was quenched with water. DMF was evaporated using membrane pump. The residue was extracted with EtOAc (150 mL × 3). The organic layer was washed with water (100 mL × 2), brine (100 mL) and then dried over Na_2SO_4, filtered and concentrated. The residue was purified by column chromatography on silica gel (160 g, hexane/EtOAc 12:1) to give compound **14** (6.55 g, 90%) as a colorless oil.

14: R_f = 0.60 (hexane/EtOAc 10:1); IR (film): v = 2940, 2873, 1709, 1462, 1401, 1251, 1080, 841, 778 cm^{-1}; ^1H NMR (400 MHz, $CDCl_3$) δ 3.65 (d, J = 5.8 Hz, 2H), 2.46 – 2.42 (m, 2H), 2.29 (dd, J = 24.3 Hz, 12.6Hz, 1H), 2.12 (dd, J = 23.6 Hz, 13.6 Hz, 2H), 2.00 – 1.86 (m, 2H), 1.55 – 1.45 (m, 1H), 1.27 – 1.16 (m, 2H), 0.88 (s, 9H), 0.86 (s, 3H), 0.81 (q, J = 4.3 Hz, 1H), 0.75 – 0.69 (m, 1H), 0.04 ppm (s, 6H); ^{13}CNMR (100 MHz, $CDCl_3$) δ 211.8, 65.1, 59. 6, 47.9, 41.1, 39.3, 38.2, 36.9, 28.3, 26.1, 23.1, 18.5, 16.5, 16.4, – 5.2, – 5.3 ppm; HRMS (ESI) m/z calcd for $C_{18}H_{32}O_2Si$ $[M+Na]^+$ 331.2064, found 331.2061; Anal. Calcd for $C_{18}H_{32}O_2Si$: C, 70.07; H, 10.45; found: C, 69.84; H, 10.38.

Procedure for the preparation of (±)-(1a*R*,1b*S*,5a*S*,6*R*,6a*S*)-6-(((*tert*-butyldimethylsilyl)oxy)methyl) -1b-methyloctahydrocyclopropa[a]indene-3,4-dione (15):

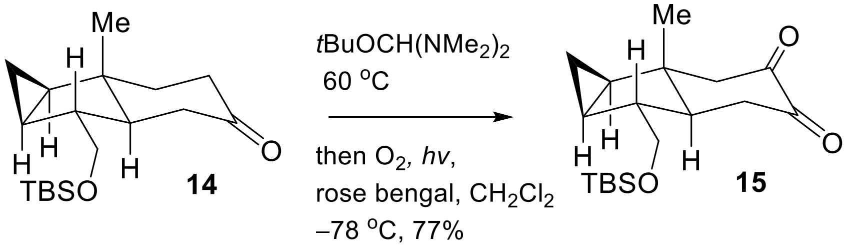

A solution of compound **14** (6.55g, 21.2 mmol, 1.00 equiv.) and *t*-butoxybis(dimethyl-amino)methane (Bredereck’s reagent) (5.7 mL, 27.6 mmol, 1.30 equiv.) was heated at 60 °C with stirring for 17 h. Then the flask was charged with O_2 and equipped with an O_2 balloon. Rose bengal (36 mg, 0.037 mmol, 0.0017 equiv.) and CH_2Cl_2 (250 mL) was added. The solution

was cooled to –78 °C and was photooxygenated with a Sylvania FMH 500 lamp as a light source. After 1 h, the reaction was complete monitored by TLC and the irradiation was stopped. The reaction mixture was warmed up to room temperature, concentrated *in vacuo*. The residue was purified by column chromatography on silica gel (160 g, hexane/EtOAc 6:1) to give compound **15** (5.29 g, 77%) as a yellow solid.

15: R_f = 0.50 (hexane/EtOAc 4:1); m.p.: 124.7 – 126.5 °C; IR (film): ν = 2945, 2870, 1720, 1462, 1401, 1254, 1095, 842, 779 cm^{-1}; ^1H NMR (400 MHz, $CDCl_3$) δ 3.78 (dd, *J* = 10.1, 5.0 Hz, 1H), 3.70 (dd, *J* = 10.1, 6.5 Hz, 1H), 2.97 (d, *J* = 17.5 Hz, 1H), 2.75 (ddd, *J* = 18.4, 18.0, 2.8 Hz, 2H), 2.64 – 2.56 (m, 1H), 2.37 (dd, *J* = 18.4, 14.8 Hz, 1H), 1.65 – 1.58 (m, 1H), 1.34 – 1.30 (m, 2H), 0.89 (s, 12H), 0.85 – 0.79 (m, 2H), 0.05 ppm (d, *J* = 2.4 Hz, 6H); ^{13}C NMR (100 MHz, $CDCl_3$) δ 195.7, 194.9, 65.0, 57.4, 55.2, 48.5, 40.4, 39.2, 28.6, 26.1, 22.9, 19.9, 18.4, 16.8, – 5.3 ppm; HRMS (ESI) *m/z* calcd for $C_{18}H_{30}O_3Si$ $[M+Na+CH_3OH]^+$ 377.2119, found 377.2114.

Procedure for the preparation of (±)-(1a*R*,1b*S*,5a*S*,6*R*,6a*S*)-6-(((*tert*-butyldimethylsilyl)oxy)-methyl)-1b-methyl-4-oxo-1,1a,1b,4,5,5a,6,6a-octahydrocyclopropa[a]inden-3-yl 2-(diethoxyph-osphoryl)propanoate (16):

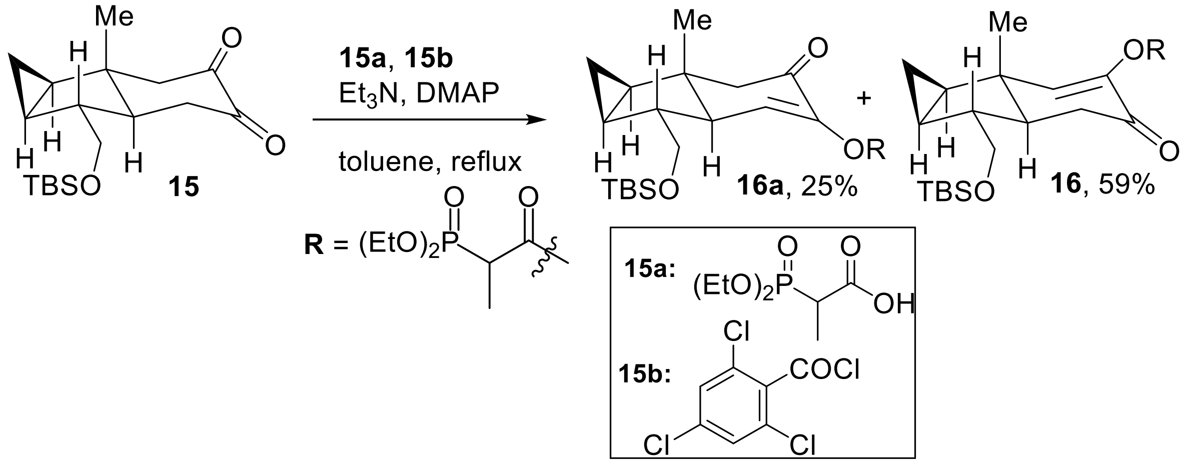

To a solution of 2-(diethylphosphono)propionic acid (**15a**) (330 mg, 1.57 mmol, 1.58 equiv.) in anhydrous toluene (3.0 mL) was added 2,4,6-trichlorobenzoyl chloride (**15b**) (0.20 mL, 1.28 mmol, 1.29 equiv.) and Et_3N (0.25 mL, 1.79 mmol, 1.80 equiv.) successively. The mixture was stirred at 20 °C for 30 min. Then compound **15** (320 mg, 0.99 mmol, 1.00 equiv.) in toluene (8.0 mL) and DMAP (122 mg, 1.0 mmol, 1.01 equiv.) was added. The reaction was heated at 120 °C for 1.5 h before being diluted with EtOAc (100 mL). The organic layer was washed with water (30 mL × 2) and brine (30 mL), then dried over Na_2SO_4, filtered and concentrated. The residue was purified by column chromatography on silica

gel (15 g, hexane/EtOAc 3:2) to give compound **16** (299 mg, 59%) as a colorless oil, together with compound **16a** (130 mg, 25.5%) as a colorless oil.

16: R_f = 0.35 (hexane/EtOAc 1:1); IR (film): ν = 3739, 2943, 1754, 1694, 1460, 1390, 1310, 1242, 1050, 839, 784 cm^{-1}; ^1H NMR (400 MHz, $CDCl_3$) δ 7.11 (s, 1H), 4.25 – 4.15(m, 4H), 3.80 – 3.62 (m, 2H), 3.26 – 3.14 (m, 1H), 2.65 – 2.55 (m, 2H), 2.35 – 2.24 (m, 1H), 1.66 – 1.58 (m, 1H), 1.55 – 1.45 (m, 4H), 1.35 – 1.30 (m, 7H), 1.27 – 1.20 (m, 1H), 1.01 (d, J = 9.1 Hz, 3H), 0.86 (s, 9H), 0.82 – 0.74 (m, 1H), 0.02 ppm (s, 6H); ^{13}C NMR (100 MHz, $CDCl_3$) δ 191.83, 191.75, 168.1, 168.0, 147.0, 146.8, 144.61, 144.59, 65.3, 65.0, 63.2, 63.1, 59.2, 59.1, 46.9, 46.8, 42.2, 39.8, 39.6, 38.4, 38.3, 37.8, 27.4, 27.4, 26.0, 25.8, 22.7, 22.6, 20.9, 20.9, 18.4, 17.0, 16.5, 16.5, 16.5, 16.4, 12.03, 12.02, 11.97, 11.95, – 3.5, – 5.30, – 5.33 ppm; ^{31}P NMR (162 MHz, $CDCl_3$) δ 24.73, 24.67, 22.95, 22.89 ppm; HRMS (ESI) *m/z* calcd for $C_{25}H_{43}O_7PSi$ $[M+Na]^+$ 537.2408, found 537.2405.

16a: R_f = 0.40 (hexane/EtOAc 1:1); IR (film): ν = 3461, 3215, 2937, 1758, 1693, 1460, 1387, 1311, 1244, 1127, 1050, 967, 838, 788 cm^{-1}; ^1H NMR (400 MHz, $CDCl_3$) δ 6.65 (d, J = 2.7 Hz, 1H), 4.23 – 4.11 (m, 4H), 3.89 (dd, J = 10.0, 4.9 Hz, 1H), 3.71 (dd, J = 10.0, 7.3 Hz, 1H), 3.19 (ddd, J = 23.3, 15.9, 7.3 Hz, 1H), 3.11 – 3.03 (m, 1H), 2.82 (dd, J = 16.6, 3.4 Hz, 1H), 2.57 (dd, J = 16.7, 5.1 Hz, 1H), 1.78 (dt, J = 7.2, 5.5 Hz, 1H), 1.51 (ddd, J = 17.7, 7.2, 4.2 Hz, 4H), 1.37 – 1.28 (m, 9H), 0.92 (s, 3H), 0.89 (s, 10H), 0.86 (d, J = 1.9 Hz, 3H), 0.82 – 0.72 (m, 2H), 0.06 ppm (s, 6H); ^{13}C NMR (100 MHz, $CDCl_3$) δ 192.3, 192.1, 145.8, 145.7, 135.11, 135.05, 65.7, 65.7, 63.1, 63.04, 63.00, 62.98, 62.93, 60.51, 60.48, 53.85, 53.83, 45.4, 45.3, 44.3, 44.2, 39.8, 39.5, 38.5, 38.2, 29.8, 27.5, 27.5, 26.09, 26.05, 26.0, 22.3, 18.7, 18.6, 18.5, 17.1, 16.6, 16.5, 12.06, 12.00, – 5.3 ppm; ^{31}P NMR (162 MHz, $CDCl_3$) δ 22.85, 22.64 ppm; HRMS (ESI) *m/z* calcd for $C_{25}H_{43}O_7PSi$ $[M+Na]^+$ 537.2408, found 537.2405.

Hydrolysis of compound 16a to recover 15:

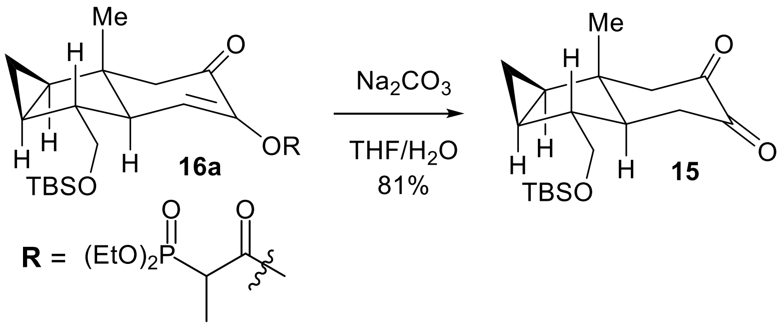

To a solution of compound **16a** (627 mg, 1.22 mmol, 1.00 equiv.) in THF (24.4 mL) was added aqueous Na_2CO_3 (24.4 mL, 0.1 M, 2.44 mmol, 2.00 equiv.). The reaction was stirred at 40 °C overnight

before being extracted with CH_2Cl_2 (40 mL × 2). The organic layer was washed with water (30 mL) and brine (30 mL), then dried over Na_2SO_4, filtered and concentrated. The residue was purified by column chromatography on silica gel (15 g, hexane/EtOAc 6:1) to give compound **15** (320 mg, 81%) as a yellow solid.

Procedure for the preparation of (±)- 15*O*-*tert*-butyldimethylsilyl shizukanolide C (17):

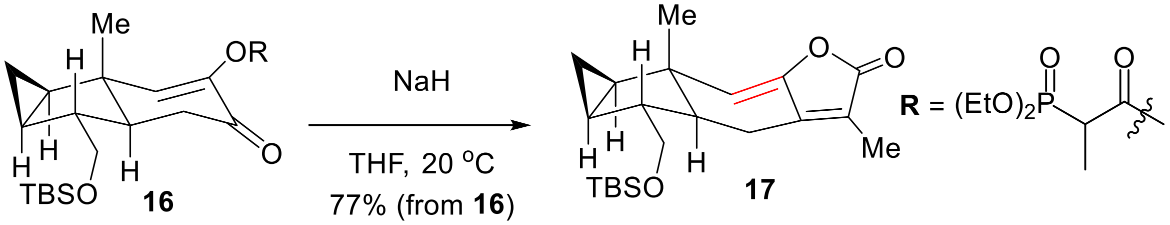

Compound **16** (1.85 g, 3.60 mmol, 1.00 equiv.) was dissolved in anhydrous THF (72 mL) and the solution was cooled down with ice bath. NaH (216 mg, 60% in mineral oil, 5.40 mmol, 1.50 equiv.) was added. After no bubbles produced, the reaction was warmed to room temperature and stirred overnight. The reaction was cooled down again with ice bath and the excess NaH was quenched with saturated aqueous NH_4Cl (30 mL) carefully. THF was evaporated and the residue was diluted with EtOAc (200 mL). The organic layer was washed with water (50 mL) and brine (50 mL), then dried over Na_2SO_4, filtered and concentrated. The residue was purified by column chromatography on silica gel (50 g, hexane/EtOAc 12:1) to give compound **17** (1.00 g, 77%) as a pale-yellow oil.

17: R_f = 0.40 (hexane/EtOAc 8:1); IR (film): ν = 3407, 2935, 1754, 1389, 1060 cm^{-1}; ^1H NMR (400 MHz, $CDCl_3$) δ 6.22 (s, 1H), 3.83 – 3.80 (m, 1H), 3.69 (t, J = 8.5 Hz, 1H), 2.78 (q, J = 12.6 Hz, 1H), 2.24 (dd, J = 23.2, 13.2 Hz, 2H), 1.85 (s, 3H), 1.51 – 1.43 (m, 1H), 1.20 – 1.12 (m, 1H), 0.91 (s, 9H), 0.89 (s, 4H), 0.78 (q, J = 7.6 Hz, 1H), 0.07 ppm (s, 6H); ^{13}C NMR (100 MHz, $CDCl_3$) δ 171.5, 149.6, 149.3, 121.8, 120.6, 65.8, 61.5, 46.2, 42.2, 27.2, 26.0, 23.4, 21.9, 21.3, 18.4, 16.8, 8.6, – 5.2 ppm; HRMS (ESI) *m/z* calcd for $C_{21}H_{32}O_3Si$ $[M+H]^+$ 361.2194, found 361.2183; Anal. Calcd for $C_{21}H_{32}O_3Si$: C, 69.95; H, 8.95; found: C, 69.95; H, 9.08.

Procedure for the preparation of (±)-(1a*R*,1b*S*,2*S*,3*S*,6*R*,6a*S*)-3-((*tert*-butyldimethylsilyl)oxy)-2,6-dihydroxy-1b,6-dimethyl-1a,1b,2,3,6,6a-hexahydrocyclopropa[a]inden-4(1*H*)-one (19):

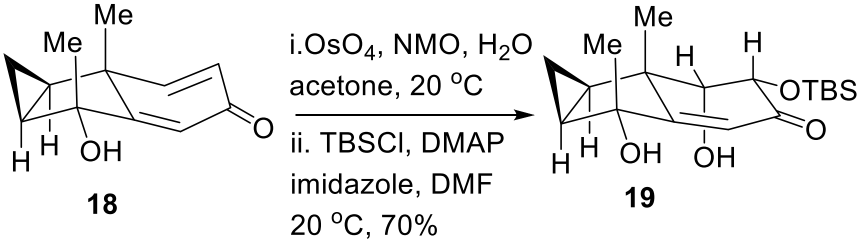

To a solution of compound **18** (4.00 g, 21.0 mmol, 1.00 equiv.) and *N*-Methylmorpholine-*N*-Oxide (3.27 g, 24.2 mmol, 1.15 equiv.) in acetone/H_2O (144/18 mL) was added OsO_4 (1.5 mL, 2.5 wt% solution in *t*BuOH) at 20 ℃. The reaction was stirred over night at this temperature. After addition of $Na_2S_2O_3{\cdot}5H_2O$ (1.80 g), the reaction was stirred for another hour and evaporated *in vacuo*, diluted with EtOAc (400 mL). The aqueous layer was extracted with EtOAc (40 mL × 5). The combined organic layers were washed with water (20 mL) and then brine (100 mL). The organic layer was dried over Na_2SO_4, filtered and concentrated. To a solution of the crude diol in DMF (52.5 mL) was added imidazole (5.72 g, 84.0 mmol, 4.00 equiv.), DMAP (513 mg, 4.2 mmol, 0.20 equiv.) and TBSCl (9.5 g, 63.0 mmol, 3.00 equiv.). The reaction was stirred at 20 ℃ overnight, then diluted with EtOAc (500 mL), and washed with water (50 mL × 3), brine (100 mL). The organic layer was dried over Na_2SO_4, filtered and concentrated. The residue was purified by column chromatography on silica gel (80 g, hexane/EtOAc 2:1) to give compound **19** (4.98 g, 70%, 74% brsm) as a white solid and compound **18** (195.4 mg).

19: R_f = 0.20 (hexane/EtOAc 2:1), m.p.: 180.9 – 189.0 ℃; IR (film): ν = 3319, 2930, 2856, 1686, 1465, 1383, 1328, 1251, 1152, 1118, 1083, 1037, 1002, 937, 863, 835, 779, 672 cm^{-1}; ^1H NMR (400 MHz, $CDCl_3$) δ 5.96 (s, 1H), 4.33 (d, *J* = 2.8 Hz, 1H), 4.24 (s, 1H), 4.16 (d, *J* = 2.8 Hz, 1H), 3.44 (s, 1H), 1.79 (s, 1H), 1.78 – 1.72 (m, 1H), 1.61 – 1.53 (m, 1H), 1.32 (s, 3H), 1.14 (s, 3H), 0.91 (s, 9H), 0.89 – 0.82 (m, 1H), 0.74 – 0.69 (m, 1H), 0.22 (s, 3H), 0.13 ppm (s, 3H); ^{13}C NMR (100 MHz, $CDCl_3$) δ 196.1, 183.0, 120.1, 80.9, 79.0, 73.8, 46.4, 30.4, 25.9, 25.4, 24.7, 22.9, 18.6, 12.2, – 4.1, – 5.5 ppm; HRMS (ESI) *m/z* calcd for $C_{18}H_{30}O_4Si$ $[M+Na]^+$ 361.1806, found 361.1803; Anal. Calcd for $C_{18}H_{30}O_4Si$: C, 63.87; H, 8.93; found: C, 63.88; H, 8.94.

Procedure for the preparation of (±)-(5*R*,5a*S*,6a*R*,6b*S*,7*S*,7a*R*)-7-((*tert*-butyldimethylsilyl)oxy)-5-hydroxy-3,5,6b-trimethyl-5a,6,6a,6b,7,7a-hexahydrocyclopropa[2,3]indeno[5,6-b]furan-2(5*H*)-one (20):[2-4]

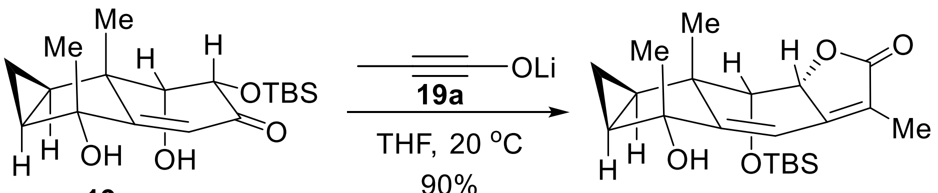

To a solution of methyl 2,2-dibromopropionate (prepared following procedures of ref. 1c) (41.8 mg, 0.17 mmol, 5.67 equiv.) in anhydrous THF (1.0 mL) was added *t*BuLi (0.52 mL, 1.3 M in pentane, 0.68 mmol, 22.7 eqiv) slowly at –78 ℃ under Argon atmosphere. The reaction was stirred at this temperature for 2 h, then 0 ℃ for 30 min. After the reaction was warmed up to 20 ℃, a solution of compound **19** (10.0 mg, 0.030 mmol, 1.00 equiv.) in anhydrous THF (1.0 mL) was added. The reaction was stirred for 30 min at this temperature and excess reagent was quenched with saturated aqueous NH_4Cl (1.0 mL). THF was evaporated away *in vacuo*. The residue was diluted with EtOAc (40 mL), washed with water (10 mL × 2) and then brine (10 mL). The organic layer was dried over Na_2SO_4, filtered and concentrated. The residue was purified by column chromatography on silica gel (4 g, hexane/EtOAc 2:1) to give compound **20** (10. 0 mg, 89%) as a white solid, whose single crystal was obtained from CH_2Cl_2 solution. **20:** R_f = 0.60 (hexane/EtOAc 2:1); m.p.: 181.6 – 184.2 ℃; IR (film): v = 3459, 2951, 1743, 1663, 1460, 1379, 1255, 1055, 843 cm^{-1}; ^1H NMR (400 MHz, $CDCl_3$) δ 6.49 (s, 1H), 5.16 (s, 1H), 4.26 (d, J = 2.1 Hz, 1H), 1.87 (d, J = 1.5 Hz, 3H), 1.62 – 1.48 (m, 2H), 1.36 (s, 3H), 1.15 (s, 3H), 0.95 – 0.84 (m, 2H), 0.76 (s, 9H), 0.08 ppm (d, J = 5.0 Hz, 6H); ^{13}C NMR (100 MHz, $CDCl_3$) δ 174.9, 170.7, 153.6, 121.1, 112.2, 79.4, 79.3, 77.8, 49.3, 30.9, 25.9, 25.7, 25.6, 23.8, 18.2, 12.8, 8.6, – 3.9, – 4.5 ppm; HRMS (ESI) *m/z* calcd for $C_{21}H_{32}O_4Si$ $[M+Na]^+$ 399.1962, found 399.1965; Anal. Calcd for $C_{21}H_{32}O_4Si$: C, 66.98; H, 8.57; found: C, 67.05; H, 8.57.

Procedure for the preparation of (±)-(5*R*,5a*S*,6a*R*,6b*R*)-5-hydroxy-3,5,6b-trimethyl-5a,6,6a,6b-tetrahydrocyclopropa[2,3]indeno[5,6-b]furan-2(5*H*)-one (21):

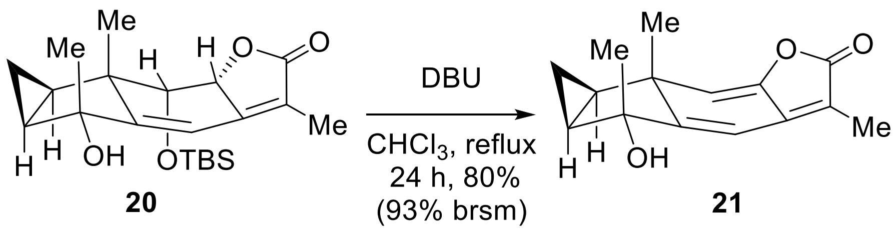

To a solution of compound **20** (81.3 mg, 0.22 mmol, 1.00 equiv.) in $CHCl_3$ (7.2 mL) was added DBU (0.161 mL, 1.08 mmol, 4.91 equiv.). Then the reaction was heated up and refluxed (75 ℃ oil bath) for 48 h. The solution was concentrated and the residue was purified by column chromatography on

silica gel (12 g, hexane/EtOAc 2:1) to give compound **21** (42.0 mg, 80%, 93% brsm) as a white solid and compound **20** (11.5 mg).

21: R_f = 0.25 (hexane/EtOAc 2:1); m.p.: 186.9 – 192.7 °C; IR (film): ν = 3440, 2924, 1750, 1638, 1448, 1383, 1261, 1053, 733 cm^{-1}; ^1H NMR (400 MHz, $CDCl_3$) δ 6.43 (s, 1H), 5.93 (s, 1H), 2.29 (s, 1H), 1.95 (s, 3H), 1.70 – 1.66 (m, 1H), 1.58 (td, J = 7.9, 4.0 Hz, 1H), 1.45 (s, 3H), 1.12 (s, 3H), 1.01 – 0.89 ppm (m, 2H); ^{13}C NMR (100 MHz, $CDCl_3$) δ 175.9, 172.4, 147.7, 142.2, 116.9, 114.8, 107.9, 78.1, 45.3, 31.6, 28.4, 25.9, 23.5, 13.5, 8.6 ppm; HRMS (ESI) *m/z* calcd for $C_{15}H_{16}O_3$ $[M+Na]^+$ 267.0992, found 267.0999; Anal. Calcd for $C_{15}H_{16}O_3$: C, 73.75; H, 6.60; found: C, 73.61; H, 6.70.

Procedure for the preparation of (±)-(1a*S*,6*R*,6a*S*,7a*R*,7b*S*,7c*S*)-6-hydroxy-4,6,7b-trimethyl-6,6a,7,7a,7b,7c-hexahydro-3*H*-cyclopropa[2,3]oxireno[2',3':4,5]indeno[5,6-b]furan-3-one (22):

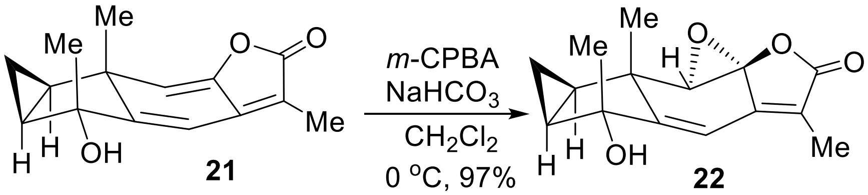

To a solution of compound **21** (70.0 mg, 0.29 mmol, 1.00 equiv.) in CH_2Cl_2 (5.7 mL) was added $NaHCO_3$ (240 mg, 2.86 mmol, 10.0 equiv.) and *m*-CPBA (282 mg, 70%, 1.15 mmol, 4.00 equiv.) at 0 °C. The reaction was stirred at this temperature for 30 min and excess reagent was quenched with saturated aqueous $Na_2S_2O_3$ (3.0 mL). The solution was diluted with EtOAc (80 mL), washed with saturated aqueous $NaHCO_3$ (10 mL), water (10 mL × 2) and brine (15 mL). The organic layer was dried over Na_2SO_4, filtered and concentrated. The residue was purified by column chromatography on silica gel (18 g, hexane/EtOAc 2:1) to give compound **22** (72.5 mg, 97%) as a white solid.

22: R_f = 0.20 (hexane/EtOAc 2:1); m.p.: 148.8 – 152.1 °C; IR (film): ν = 3423, 2979, 2930, 1772, 1677, 1446, 1386, 1256, 1058, 954, 522 cm^{-1}; ^1H NMR (400 MHz, $CDCl_3$) δ 6.32 (s, 1H), 4.11 (s, 1H), 1.97 (s, 3H), 1.73 (td, J = 7.9, 3.9 Hz, 1H), 1.68 – 1.60 (m, 1H), 1.38 (s, 3H), 1.01 (s, 3H), 0.90 (dd, J = 15.4, 8.7 Hz, 1H), 0.82 – 0.75 ppm (m, 1H); ^{13}C NMR (100 MHz, $CDCl_3$) δ 172.6, 171.4, 148.4, 122.6, 109.2, 87.2, 78.7, 60.6, 45.0, 31.5, 26.2, 23.6, 22.2, 11.9, 9.3 ppm; HRMS (ESI) *m/z* calcd for $C_{15}H_{16}O_4[M+Na]^+$ 283.0941, found 283.0929; Anal. Calcd for $C_{15}H_{16}O_4$: C, 69.22; H, 6.20; found: C, 69.23; H, 6.28.

Procedure for the preparation of (±)-(1a*S*,6a*S*,7a*R*,7b*S*,7c*S*)-4,7b-dimethyl-6-methylene-6,6a,7,7a,7b,7c-hexahydro-3*H*-cyclopropa[2,3]oxireno[2',3':4,5]indeno[5,6-b]furan-3-one (23):

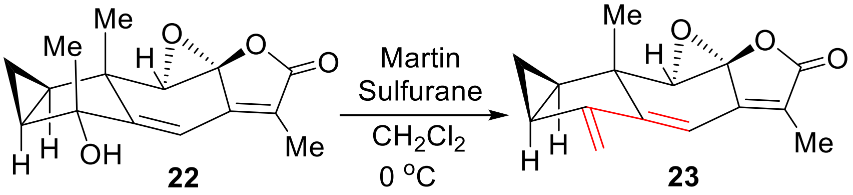

Compound **22** (467 mg, 1.8 mmol, 1.00 equiv.) was dissolved in anhydrous CH_2Cl_2 (20 mL) and the solution was cooled down to 0 ℃ under Argon atmosphere. A solution of Martin sulfurane dehydrating reagent (1.75 g, 2.6 mmol, 1.44 equiv.) in anhydrous CH_2Cl_2 (15 mL) was added. After stirring at this temperature for 5 min, saturated aqueous $NaHCO_3$ (20 mL) was added and the mixture was diluted with CH_2Cl_2 (100 mL) and washed with brine (30 mL). The organic layer was dried over Na_2SO_4, filtered and concentrated (some solvent left). The residue was purified by column chromatography on silica gel (the silica gel was rinsed with hexane containing 1% Et_3N) (40 g, hexane/EtOAc 10:1). The eluent containing product was combined, was added xylene and concentrated. The crude product **23** was stored in xylene in a solution form at –20 ℃. R_f = 0.65 (hexane/EtOAc 4:1).

Procedure for the preparation of Compound 24:

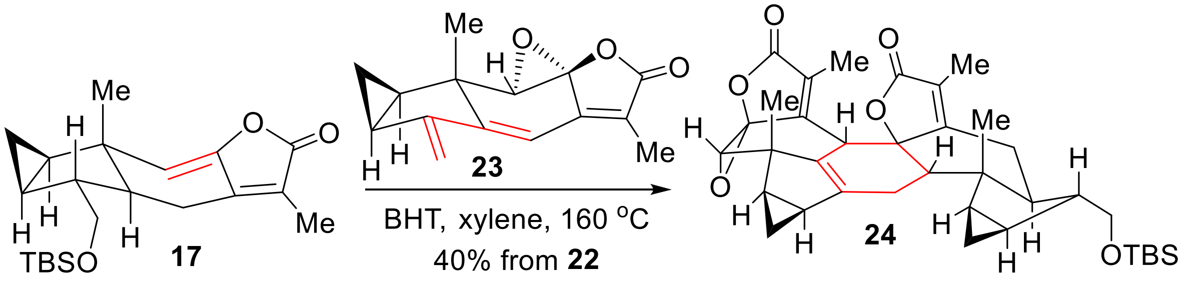

To a solution (160 ℃ oil bath) of dienophile **17** (600 mg, 1.67 mmol, 4.56 equiv.) and BHT (55 mg, 0.25 mmol, 0.69 equiv.) in refluxing xylene (10 mL) was added a solution of triene **23** (from 94 mg precursor **22**, 0.36 mmol, 1.00 equiv.) in xylene (47 mL) via syringe pump (0.30 mL/h). After the addition, the reaction was refluxed for another 10 h. Then the solvent was evaporated using membrane pump and the residue was purified by column chromatography on silica gel (20 g, hexane/EtOAc 12:1 – 7:1) to give compound **24** (86.7 mg, 40% from **22**) as a pale-yellow gum, together with the recovered compound **17** (422 mg, 70%).

24: R_f = 0.60 (hexane/EtOAc 4:1); IR (film): ν = 2923, 2863, 2401, 1758, 1641, 1448, 1254, 1089, 742 cm^{-1}; ^1H NMR (400 MHz, $CDCl_3$) δ 3.82 (s, 1H), 3.76 (dd, J = 9.8, 6.3 Hz, 1H), 3.53 (t, J = 9.1 Hz, 2H), 2.64 (d, J = 17.0 Hz, 1H), 2.50 (ddd, J = 16.8, 6.7, 3.7 Hz, 1H), 2.23 (d, J = 2.9 Hz, 3H), 1.87 (td, J = 6.9, 3.0 Hz, 1H), 1.83 – 1.81 (m, 1H), 1.80 (s, 3H), 1.76 (d, J = 5.6 Hz, 1H), 1.73 (d, J = 1.4 Hz, 3H),

1.47 – 1.39 (m, 1H), 1.28 (td, J = 8.3, 3.9 Hz, 1H), 1.08 – 1.05 (m, 1H), 1.03 (s, 3H), 0.97 – 0.92 (m, 1H), 0.85 (s, 9H), 0.79 (dd, J = 9.3, 3.9 Hz, 1H), 0.73 – 0.66 (m, 1H), 0.61 (s, 3H), 0.37 (dd, J = 7.6, 4.1 Hz, 1H), 0.02 ppm (d, J = 0.6 Hz, 6H); ^{13}C NMR (100 MHz, $CDCl_3$) δ 173.2, 169.1, 166.4, 152.2, 142.5, 131.5, 129.3, 124.4, 90.8, 87.6, 66.4, 66.0, 57.0, 53.5, 49.8, 46.5, 43.5, 40.2, 26.4, 26.08, 26.06, 25.2, 25.1, 24.3, 24.1, 21.7, 20.1, 18.3, 16.5, 16.2, 11.0, 9.0, – 5.3, – 5.4 ppm; HRMS (ESI) *m/z* calcd for $C_{36}H_{46}O_6Si$ $[M+Na]^+$ 625.2956, found 625.2954.

Procedure for the preparation of Compound 25:

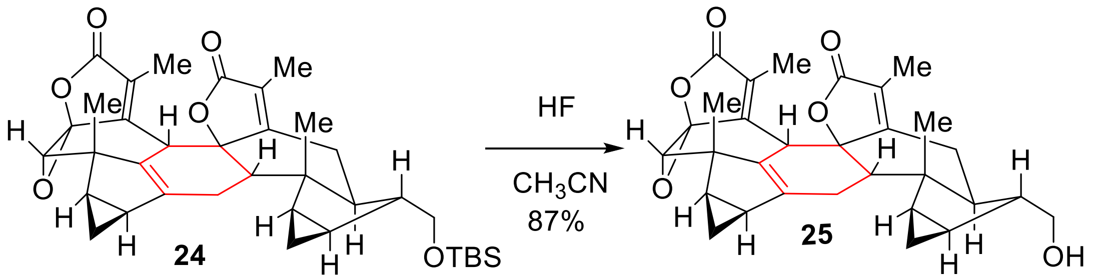

To a solution of compound **24** (40 mg, 0.066 mmol, 1.00 equiv.) in CH_3CN (4.0 mL) in a plastic tube was added aqueous HF (1.0 mL, 40%) at 20 °C. The reaction was stirred at room temperature overnight. $NaHCO_3$ (first solution then solid) was added to quench the excess acid until no bubbles produced. The mixture was extracted with EtOAc (20 mL × 3) and the combined organic layers were washed with water (20 mL) and brine (20 mL). The organic layer was dried over Na_2SO_4, filtered and evaporated. The residue was purified by column chromatography on silica gel (8 g, hexane/EtOAc 3:2 – 1:1) to give compound **25** (28 mg, 87%) as a white solid.

25: R_f = 0.15 (hexane/EtOAc 2:1); IR (film): v = 3747, 3422, 2929, 2876, 1758, 1673, 1448, 1386, 1302, 1060, 732, 663, 530 cm^{-1}; ^{1}H NMR (400 MHz, $CDCl_3$) δ 3.86 (s, 1H), 3.77 (dd, J = 10.1, 6.4 Hz, 1H), 3.64 (dd, J = 10.1, 6.8 Hz, 1H), 3.59 (s, 1H), 2.69 (d, J = 17.0 Hz, 1H), 2.51 (ddd, J = 16.8, 6.9, 3.8 Hz, 1H), 2.41 – 2.32 (m, 1H), 2.31 – 2.21 (m, 1H), 2.13 (ddd, J = 17.6, 5.5, 1.9 Hz, 1H), 1.90 (td, J = 6.9, 3.0 Hz, 1H), 1.86 – 1.82 (m, 2H), 1.80 (d, J = 1.2 Hz, 3H), 1.76 (d, J = 1.4 Hz, 3H), 1.51 – 1.44 (m, 1H), 1.34 (td, J = 8.2, 3.8 Hz, 1H), 1.22 – 1.16 (m, 1H), 1.05 (s, 3H), 0.96 (td, J = 7.8, 4.7 Hz, 1H), 0.83 – 0.79 (m, 1H), 0.73 (td, J = 8.3, 5.8 Hz, 1H), 0.62 (s, 3H), 0.41 ppm (dd, J = 7.6, 4.2 Hz, 1H); ^{13}C NMR (100 MHz, $CDCl_3$) δ 173.2, 169.3, 165.9, 152.2, 142.5, 131.7, 129.7, 124.7, 91.0, 87.7, 66.6, 64.9, 55.6, 53.6, 49.8, 46.2, 43.5, 40.2, 26.1, 25.9, 25.3, 25.2, 24.3, 24.2, 22.0, 20.3, 16.6, 16.3, 11.1, 9.1 ppm; HRMS (ESI) *m/z* calcd for $C_{30}H_{32}O_6$ $[M+Na]^+$ 511.2091, found 511.2093.

Procedure for the preparation of (±)-9-*epi*-shizukaol E (26):

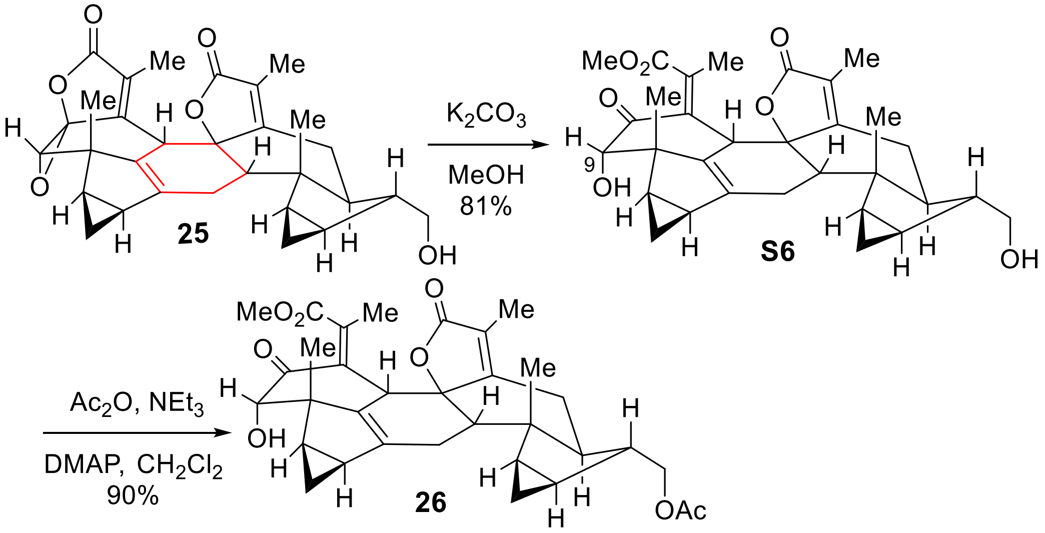

To a solution of compound **25** (8.7 mg, 0.018 mmol, 1.00 equiv.) in MeOH (2.0 mL) was added K_2CO_3 (4.9 mg, 0.036 mmol, 2.00 equiv.) at 20 °C. After stirring for 15 min at this temperature, saturated aqueous NH_4Cl (0.5 mL) was added. The organic solvent was evaporated *in vacuo* and the residue was extracted with EtOAc (15 mL × 3). The combined organic layers were washed with brine (15 mL), dried over Na_2SO_4, filtered and concentrated. The residue was purified by column chromatography on silica gel (8 g, hexane/EtOAc 2:3) to give compound **S6** (7.5 mg, 81%) as a pale-yellow gum.

S6: R_f = 0.30 (hexane/EtOAc 2:3); IR (film): ν = 3845, 3740, 3419, 2930, 2873, 1739, 1451, 1385, 1296, 1060, 732, 527 cm^{-1}; ^{1}H NMR (400 MHz, $CDCl_3$) δ 3.86 (s, 1H), 3.82 (d, *J* = 3.3 Hz, 1H),3.71 (s, 3H), 3.74 – 3.63 (m, 2H), 2.76 (d, *J* = 16.7 Hz, 1H), 2.60 – 2.52 (m, 1H), 2.35 (dd, *J* = 18.0, 4.6 Hz, 1H), 2.15 (dd, *J* = 17.8, 13.4 Hz, 1H), 1.90 – 1.85 (m, 4H), 1.79 (d, *J* = 0.9 Hz, 3H), 1.75 (s, 3H), 1.37 (ddd, *J* = 15.8, 10.1, 4.6 Hz, 2H), 1.24 – 1.18 (m, 1H), 1.15 (s, 3H), 0.95 – 0.89 (m, 1H), 0.79 (dd, *J* = 9.0 3.7 Hz, 1H), 0.73 – 0.66 (m, 1H), 0.57 (s, 3H), 0.30 ppm (dd, *J* = 7.7, 3.7 Hz, 1H); ^{13}C NMR (100 MHz, $CDCl_3$) δ 205.7, 173.7, 170.0, 166.2, 143.0, 141.8, 137.3, 129.7, 123.82, 92.2, 79.3, 62.6, 57.1, 54.4, 52.8, 50.0, 46.8, 43.9, 41.4, 25.5, 25.2, 24.8, 24.7, 24.2, 24.0, 22.0, 20.9, 18.9, 16.8, 15.6, 8.7 ppm; HRMS (ESI) *m/z* calcd for $C_{31}H_{36}O_7$ $[M+Na]^+$ 543.2353, found 543.2356.

To a solution of diol **S6** (7.0 mg, 0.013 mmol, 1.00 equiv.), triethylamine (20 µL, 0.14 mmol, 10.0 equiv.) and DMAP (trace amount) in CH_2Cl_2 (2.0 mL) was added Ac_2O (10 µL, 0.11 mmol, 8.5 equiv.) at 20 °C. The reaction was stirred for 30 min and excess anhydride was quenched with water (1.0 mL).

The mixture was diluted with EtOAc (30 mL) and washed with saturated aqueous $NaHCO_3$ (10 mL), water (10 mL) and brine (10 mL). The organic layer was dried over Na_2SO_4, filtered and concentrated. The residue was purified by column chromatography on silica gel (4 g, hexane/EtOAc 2:1) to afford compound **26** (6.8 mg, 90%) as a pale-yellow oil.

26: R_f = 0.20 (hexane/EtOAc 2:1); IR (film): ν = 3744, 3437, 2924, 1737, 1527, 1444, 1382, 1250, 1060, 502, 731 cm^{-1}; ^1H NMR (400 MHz, $CDCl_3$) δ 4.10 (dd, J = 11.1, 7.6 Hz, 1H), 4.00 (dd, J = 11.0, 7.8 Hz, 1H), 3.83 (s, 1H), 3.81 (d, J = 3.0 Hz, 1H), 3.68 (s, 3H), 2.75 (d, J = 16.6 Hz, 1H), 2.57 – 2.51 (m, 1H), 2.38 (ddd, J = 17.9, 6.4, 1.9 Hz, 1H), 2.18 (dd, J = 17.9, 13.3 Hz, 1H), 2.09 (s, 3H), 1.92 – 1.85 (m, 4H), 1.79 (d, J = 1.3 Hz, 3H), 1.74 (s, 3H), 1.53 (ddd, J = 10.9, 7.4, 3.6 Hz, 1H), 1.39 (td, J = 8.2, 3.8 Hz, 1H), 1.16 (s, 3H), 1.10 – 1.04 (m, 1H), 0.95 – 0.87 (m, 2H), 0.79 – 0.66 (m, 2H), 0.58 (s, 3H), 0.32 ppm (dd, J = 7.5, 4.3 Hz, 1H); ^{13}C NMR (100 MHz, $CDCl_3$) δ 204.6, 173.7, 171.6, 169.9, 166.2, 142.6, 141.3, 137.0, 130.3, 123.6, 92.1, 79.1, 66.2, 58.3, 54.3, 52.7, 49.7, 44.1, 43.8, 41.3, 25.5, 25.2, 24.9, 24.6, 24.1, 24.0, 22.5, 22.2, 21.2, 18.8, 16.7, 15.6, 8.7 ppm; HRMS (ESI) m/z calcd for $C_{33}H_{38}O_8$ $[M+Na]^+$ 585.24589, found 585.24588.

Total synthesis of (±)-shizukaol E (2)

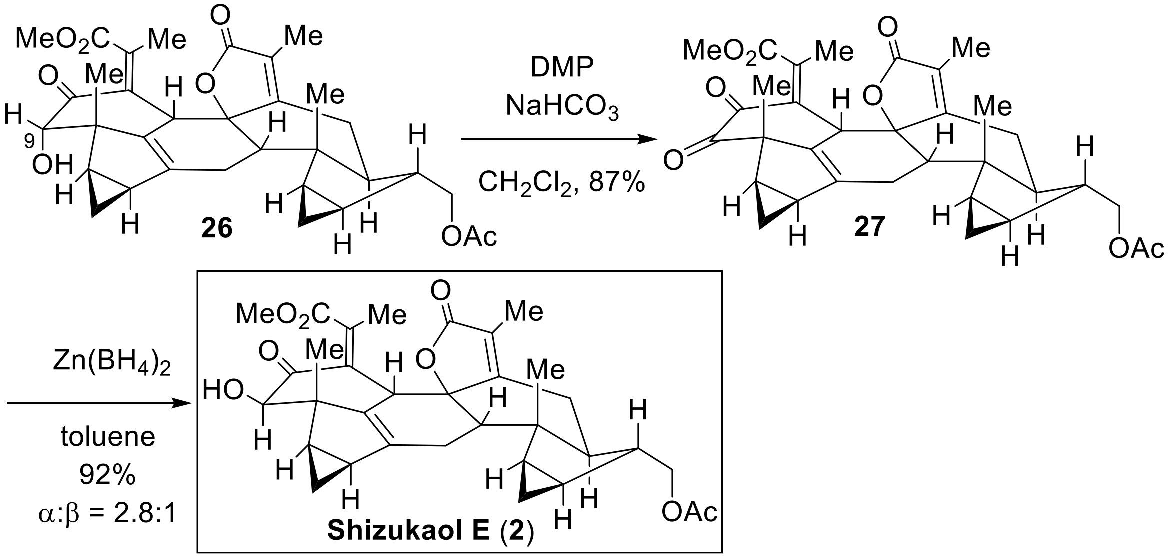

To a solution of compound **26** (6.0 mg, 0.011 mmol) and $NaHCO_3$ (4.5 mg, 0.054 mmol, 5.00 equiv.) in CH_2Cl_2 (1.0 mL) was added Dess-Martin periodinane (9.0 mg, 0.021 mmol, 2.00 equiv.). The reaction was stirred at room temperature for 20 min and saturated aqueous $Na_2S_2O_3$ (0.5 mL) was added. The mixture was diluted with CH_2Cl_2 (30 mL) and washed with water (10 mL), brine (10 mL), then dried over Na_2SO_4, filtered and evaporated. The residue was purified by column chromatography on silica gel

(6 g, hexane/EtOAc 2:1) to afford dione **27** (5.2 mg, 87%) as a yellow oil.

27: R_f = 0.30 (hexane/EtOAc 2:1); IR (film): ν = 3840, 3741, 3613, 2925, 1742, 1524, 1458, 1246, 1052, 916, 800, 726 cm^{-1}; ^{1}H NMR (400 MHz, $CDCl_3$) δ 4.01 (s, 1H), 3.97 (d, *J* = 7.1 Hz, 1H), 3.82 – 3.76 (m, 1H), 3.74 (s, 3H), 2.80 (d, *J* = 16.4 Hz, 1H), 2.61 – 2.51 (m, 2H), 2.32 – 2.18 (m, 2H), 2.08 (s, 3H); 1.93 – 1.88 (m, 2H), 1.86 (s, 3H), 1.80 (s, 3H), 1.50 – 1.42 (m, 1H), 1.35 (s, 3H), 1.32 – 1.26(m, 2H), 1.17 (dd, *J* = 12.1, 7.5 Hz, 1H), 1.03 – 0.97 (m, 1H), 0.74 – 0.64 (m, 2H), 0.57 (s, 3H), 0.39 ppm (dd, *J* = 7.6, 4.0 Hz, 1H); ^{13}C NMR (100 MHz, $CDCl_3$) δ 192.0, 186.1, 173.5, 171.0, 169.7, 165.3, 148.7, 148.4, 133.7, 127.7, 123.8, 92.2, 66.1, 61.2, 60.6, 54.3, 53.1, 44.4, 43.6, 40.9, 26.4, 25.4, 25.0, 24.2, 24.1, 24.0, 21.9, 21.1, 19.6, 19.1, 17.5, 16.5, 8.6 ppm; HRMS (ESI) *m/z* calcd for $C_{33}H_{36}O_8$ $[M+Na]^+$ 583.2302, found 583.2301.

To a solution of dione **27** (10.7 mg) in toluene (2.0 mL) was added $Zn(BH_4)_2$ (1 drop, 1.0 M in THF) at 20 °C under argon atmosphere. The reaction was stirred for about 5 min until the yellow color vanished. HOAc (0.2 mL, 1.0 M in EtOAc) was added for quenching the reaction mixture. The solvent was evaporated and the residue was purified through a flash column on silica gel (4 g, hexane/EtOAc 3:2). The isomer mixture obtained was further purified by column chromatography (very thin and long column) on silica gel (10 g, hexane/EtOAc 2:1) to obtain shizukaol E (**2**) (2.6 mg, 24%) as a colorless oil, and its epimer **26** (7.3 mg, 68%).

(±)-Shizukaol E (2): R_f = 0.20 (hexane/EtOAc 2:1); IR (film): ν = 3450, 2924, 2857, 2088, 1742, 1639, 1443, 1382, 1249, 1087, 1049, 607 cm^{-1}; ^{1}H NMR (400 MHz, $CDCl_3$) δ 4.04 (s, 1H), 3.95 (dd, *J* = 11.1, 6.8 Hz, 1H), 3.87 (d, *J* = 3.6 Hz, 1H), 3.78 (s, 3H), 3.77 (*J* = 11.1, 8.0 Hz, 1H), 3.35 (s, 1H), 2.71 (dd, *J* = 16.4, 1.6 Hz, 1H), 2.61 (ddd, *J* = 16.5, 5.7, 3.9 Hz, 1H), 2.40 (ddq, *J* = 18.1, 6.1, 2.1 Hz, 1H), 2.24 (dd, *J* = 17.8, 13.3 Hz, 1H), 2.08 (s, 3H), 2.04 (ddd, *J* = 8.2, 5.8, 4.4 Hz, 1H), 1.84 (dd, *J* = 7.3, 2.8 Hz, 1H), 1.81 (dd, *J* = 6.0, 2.0 Hz, 1H), 1.80 (s, 6H), 1.77 – 1.73 (m, 1H), 1.57 – 1.54 (m, 1H), 1.41 (ddd, *J* = 8.4, 8.2, 4.0 Hz, 1H), 1.09 (dddd, *J* = 8.1, 8.1, 3.8, 3.8 Hz, 1H), 1.01 (s, 3H), 0.98 (ddd, *J* = 7.5, 7.5, 3.4 Hz, 1H), 0.84 – 0.81 (m, 1H), 0.75 (ddd, *J* = 8.4, 8.4, 5.5 Hz, 1H), 0.61 (s, 3H), 0.27 (ddd, *J* = 4.2, 4.2, 3.1 Hz, 1H); ^{13}C NMR (125 MHz, $CDCl_3$) δ 200.6, 173.4, 171.0, 170.7, 165.5, 147.2, 142.3, 131.8, 131.5, 124.2, 92.5, 80.1, 66.2, 59.3, 54.7, 52.6, 51.2, 44.0, 43.0, 40.5, 25.6, 25.4, 25.0, 24.7, 24.3, 23.9, 21.8, 20.8, 20.3, 16.6, 15.8, 15.1, 8.6 ppm; HRMS (ESI) *m/z* calcd for $C_{33}H_{38}O_8$ $[M+Na]^+$ 585.2459, found 585.2458.

Procedures for the preparation of (±)- (1a*R*,1b*S*,5a*S*,6a*S*)-1*b*-methyl-6-methyleneocta-hydrocyclopro-pa[*a*]indene-3,4-dione (28):

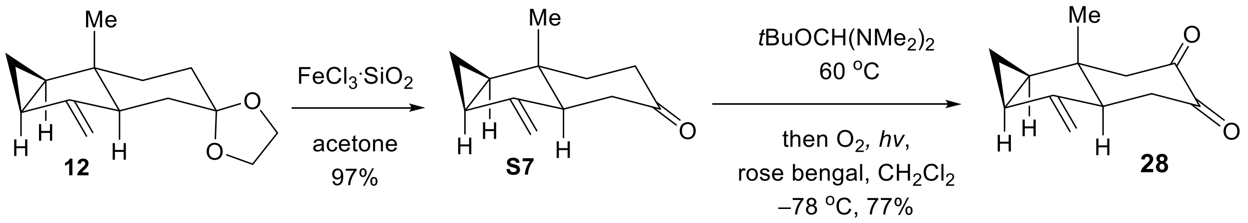

To a solution of compound **12** (500 mg, 2.27 mmol, 1.00 equiv.) in acetone (22.7 mL) was added $FeCl_3·SiO_2$ (227 mg) at 20 °C. After being stirred overnight, the solvent was evaporated *in vacuo* and the residue was purified by column chromatography on silica gel (hexane/EtOAc 12:1) to give compound **S7** (390 mg, 97%) as a colorless oil.

S7: R_f = 0.65 (hexane/EtOAc 10:1); IR (film): *ν* = 3706, 3404, 2920, 2854, 1602, 1440, 1101, 1040, 675 cm^{-1}; ^1H NMR (500 MHz, $CDCl_3$) δ 4.97 (s, 1H), 4.61 (s, 1H), 2.85 (dd, *J* = 14.3, 3.4 Hz, 1H), 2.54 – 2.45 (m, 2H), 2.34 (dd, *J* = 16.3, 3.9 Hz, 1H), 2.13 (dd, *J* = 16.2, 14.5 Hz, 1H), 2.07 – 1.94 (m, 3H), 1.41 (td, *J* = 7.3, 4.1 Hz, 1H), 0.91 – 0.82 (m, 2H), 0.76 ppm (s, 3H); ^{13}C NMR (126 MHz, $CDCl_3$) δ 211.1, 152.0, 106.0, 60.9, 39.3, 38.2, 37.7, 36.4, 28.0, 24.1, 17.0, 16.6 ppm; HRMS (ESI) *m/z* calcd for $C_{12}H_{16}O$ $[M+H]^+$ 177.12739, found 177.12742.

A mixture of compound **S7** (59 mg, 0.335 mmol, 1.00 equiv.) and *t*-butoxybis(dimethyl-amino)methane (Bredereck's reagent) (103 μL, 0.50 mmol, 1.49 equiv.) was heated at 55 °C with stirring for 11.5 h. Then the flask was charged with O_2 and equipped with an O_2 balloon. Rose Bengal (1 mg) and CH_2Cl_2 (6.7 mL) was added. The solution was cooled to –78 °C and was photooxygenated with a Sylvania FMH 500 lamp as a light source. After 1 h, the reaction was complete (TLC check) and the irradiation was stopped. The reaction mixture was warmed up to room temperature, concentrated *in vacuo*. The residue was purified by column chromatography on silica gel (hexane/EtOAc 4:1) to give compound **28** (44.6 mg, 77%) as a yellow gum.

28: R_f = 0.25 (hexane/EtOAc 4:1); IR (film): *ν* = 3076, 3015, 2958, 2920, 2863, 1718, 1662, 1408, 1385, 1092, 1038, 893, 797, 676, 614 cm^{-1}; ^1H NMR (500 MHz, $CDCl_3$) δ 5.09 (s, 1H), 4.72 (s, 1H), 3.31 (d, *J* = 13.5 Hz, 1H), 3.00 (d, *J* = 17.5 Hz, 1H), 2.80 (d, *J* = 18.7 Hz, 1H), 2.76 (dd, *J* = 13.8, 4.8 Hz, 1H), 2.40 (dd, *J* = 18.7, 14.6 Hz, 1H), 2.13 (t, *J* = 9.2 Hz, 1H), 1.52 (td, *J* = 7.5, 3.6 Hz, 1H), 0.95 (td, *J* = 8.6, 5.5 Hz, 1H), 0.84 (dd, *J* = 9.2, 3.8 Hz, 1H), 0.81 ppm (s, 3H); ^{13}C NMR (100 MHz, $CDCl_3$) δ 194.9, 193.8, 150.7, 107.6, 58.1, 54.6, 38.5, 37.3, 27.8, 24.2, 20.6, 16.9 ppm; HRMS (ESI) *m/z* calcd for $C_{12}H_{14}O_2$ $[M+Na]^+$ 213.0886, found 213.0884.

Procedures for the preparation of (±)-chloranthalactone A (6):

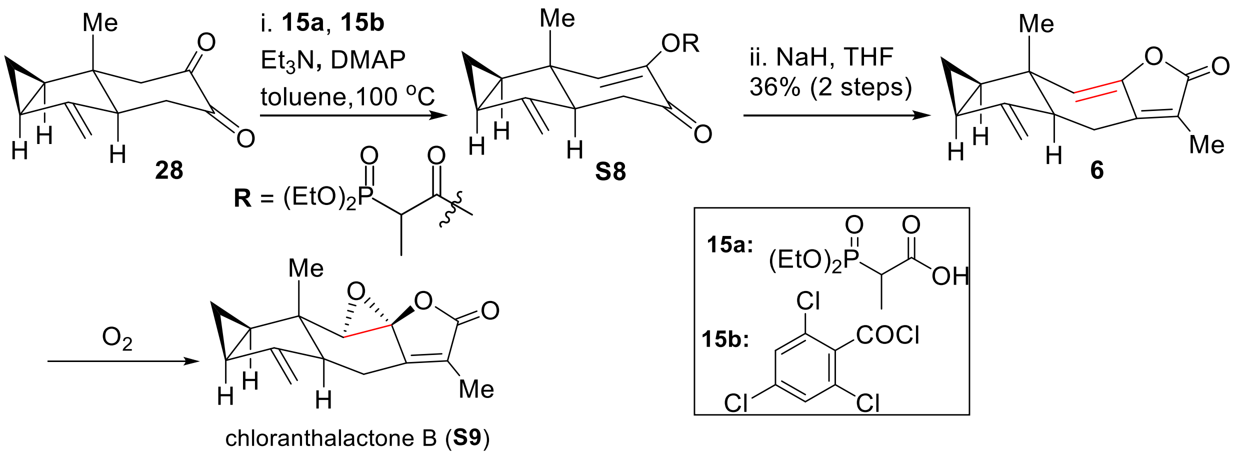

To a solution of 2-(diethylphosphono)propionic acid (**15a**) (88.5 mg, 0.42 mmol, 1.83 equiv.) in anhydrous toluene (1.0 mL) was added 2,4,6-trichlorobenzoyl chloride (**15b**) (55 μL, 0.35 mmol, 1.52 equiv.) and triethylamine (82 μL, 0.59 mmol, 2.57 equiv.) successively. The mixture was stirred at 20 °C for 30 min. Then compound **28** (44.6 mg, 0.23 mmol, 1.00 equiv.) in toluene (2.0 mL) and DMAP (31 mg, 0.26 mmol, 1.13 equiv.) was added. The reaction was heated at 100 °C for 2 h before diluted with EtOAc (50 mL). The organic layer was washed with water (20 mL × 2) and brine (20 mL), then dried over Na_2SO_4, filtered and concentrated. The residue was purified by column chromatography on silica gel (hexane/EtOAc 1:1) to give compound **S8** (52.7 mg, 59%) as a yellow oil.

The obtained product **S8** (0.14 mmol, 1.00 equiv.) was then dissolved in anhydrous THF (3.0 mL) and the solution was cooled down with ice bath. NaH (11 mg, 60% in mineral oil, 0.28 mmol, 2.00 equiv.) was added. After no bubbles produced, the reaction was warmed to room temperature and stirred for 2 h. The reaction was cooled down again with ice bath and excess base was quenched with saturated aqueous NH_4Cl (2 mL) carefully. THF was evaporated and the residue was diluted with EtOAc (50 mL). The organic layer was washed with water (20 mL) and brine (20 mL), then dried over Na_2SO_4, filtered and concentrated. The residue was purified by column chromatography on silica gel (hexane/EtOAc 15:1) to give compound **6** (19.2 mg, 61%) as a colorless oil. During the workup and purification process, part of the butenolide was oxidized by air to furnish compound **S9** as a white solid.

6: R_f = 0.55 (hexane/EtOAc 10:1); IR (film): ν = 3082, 2968, 2930, 1768, 1646, 1439, 1373, 1321, 1260, 1103, 1019, 885 cm^{-1}; ^1H NMR (400 MHz, $CDCl_3$) δ 6.25 (s, 1H), 5.06 (s, 1H), 4.79 (s, 1H), 2.98 (dq, J = 13.7, 3.0 Hz, 1H), 2.70 (dd, J = 16.8, 3.8 Hz, 1H), 2.27 (tq, J = 15.3, 2.0 Hz, 1H), 2.01 – 1.95 (m, 1H), 1.90 (d, J = 1.8 Hz, 3H), 1.66 (td, J = 7.5, 3.8 Hz, 1H), 0.95 – 0.86 (m, 2H), 0.79 ppm (s, 3H); ^{13}C NMR

(100 MHz, $CDCl_3$) δ 171.3, 150.2, 149.7, 148.1, 122.6, 119.9, 106.7, 62.2, 40.2, 26.5, 22.6, 22.3, 21.5, 17.1, 8.8 ppm; HRMS (ESI) *m/z* calcd for $C_{15}H_{16}O_2$ $[M+Na]^+$ 251.1043, found 251.1040.

S9: R_f = 0.50 (hexane/EtOAc 10:1); IR (film): *ν* = 2971, 2928, 1792, 1663, 1625, 1432, 1261, 1075, 1027, 943, 880 cm^{-1}; ^1H NMR (400 MHz, $CDCl_3$) δ 5.04 (brq, *J* = 1.2 Hz, 1H), 4.70 (t, *J* = 2.3 Hz, 1H), 4.18 (s, 1H), 3.39 (ddt, *J* = 12.7, 5.3, 2.6 Hz, 1H), 2.61 – 2.51 (m, 1H), 2.12 (ddq, *J* = 18.9, 13.2, 1.8 Hz, 1H), 2.05 – 1.97 (m, 1H), 1.90 (t, *J* = 1.7 Hz, 3H), 1.72 (td, *J* = 7.6, 3.6 Hz, 1H), 0.91 (ddd, *J* = 8.9, 7.9, 5.5 Hz, 1H), 0.84 (dt, *J* = 5.5, 3.7 Hz, 1H), 0.65 ppm (s, 3H); ^{13}C NMR (125 MHz, $CDCl_3$) δ 170.5, 152.4, 150.1, 129.2, 106.9, 88.0, 64.6, 50.8, 41.3, 24.0, 23.1, 21.43, 17.1, 16.9, 9.1 ppm; HRMS (ESI) *m/z* calcd for $C_{15}H_{16}O_3$ $[M+Na]^+$ 267.0992, found 267.0991.

Procedure for the preparation of compound (4a*R*,5*S*,5a*S*,6a*S*,6b*S*)-5-((tert-butyldimethylsilyl)oxy) -3,6b-dimethyl-4a,5,5a,6,6a,6b-hexahydrocyclopropa[2,3]indeno[5,6-*b*]furan-2(4*H*)-one (S16):

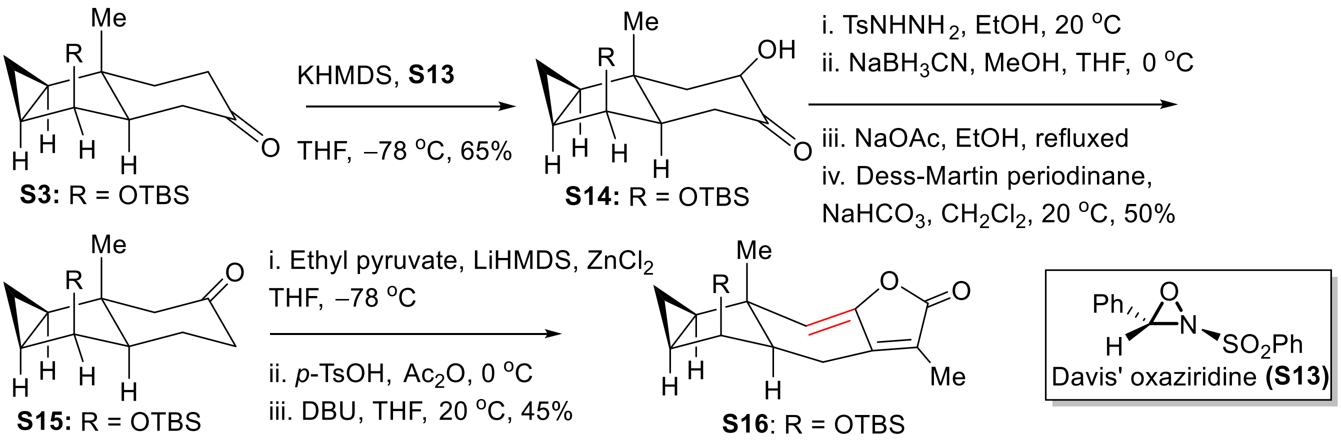

To a solution of compound **S3** (20.0 mg, 0.068 mmol) in THF (2.0 mL) was added KHMDS (0.20 mL, 0.10 mmol, 0.5 M in toluene) at –78 °C under argon atmosphere. The reaction was stirred at this temperature for 30 min, and a solution of Davis' oxaziridine **S13** (26.6 mg, 0.10 mmol) in THF (1.0 mL) was added slowly. The reaction was stirred for additional 30 min and quenched by aqueous NH_4Cl (5 mL). Evaporate THF away *in vacuo*. The residue was diluted with EtOAc (50 mL), washed with water (10 mL × 2) and then brine (10 mL). The organic layer was dried over Na_2SO_4, filtered and concentrated. The residue was purified by column chromatography on silica gel (Hexanes/EtOAc 6:1) to give compound **S14** (13.7 mg, 65%) as a white solid: R_f = 0.40 (Hexanes/EtOAc 4:1); m.p.: 46.6 – 47.8 °C; IR (film): *ν* = 2955, 2929, 1702, 1618, 1257, 1078 cm^{-1}; ^1H NMR (400 MHz, $CDCl_3$) δ 4.27 (t, *J* = 5.2 Hz, 1H), 4.26 – 4.23 (m, 1H), 2.56 – 2.46 (m, 2H), 2.34 (dd, *J* = 2.8, 14.4 Hz, 1H), 1.91 (dt, *J* = 3.6, 15.2

Hz, 1H), 1.78 – 1.72 (m, 1H), 1.61 (t, *J* = 11.2 Hz, 1H), 1.37 (td, *J* = 4.0, 8.0 Hz, 1H), 1.30 (dd, *J* = 4.0, 9.2 Hz, 1H), 1.20 (s, 3H), 0.88 (s, 9H), 0.67 (td, *J* = 5.6, 8.8 Hz, 1H), 0.06 (s, 3H), 0.01 ppm (s, 3H); ^{13}C NMR (100 MHz, $CDCl_3$) δ 211.7, 73.6, 73.4, 62.7, 48.0, 40.1, 37.8, 30.2, 27.9, 25.9, 19.3, 18.3, 11.3, –4.6, –5.2 ppm; HRMS (ESI) *m/z* calcd for $C_{17}H_{30}O_3SiNa$ $[M+Na]^+$ 333.1856, found 333.1862.

To a solution of compound **S14** (46.0 mg, 0.15 mmol) in ethanol (2.0 mL) was added tosyl hydrazide (36.0 mg, 0.19 mmol) and the solution was stirred at 20 °C for 3 h. The solution was then evaporated *in vacuo* and used for the next step without further purification. This crude mixture was dissolved in a mixture of solvents (methanol/THF 0.9 mL: 0.9 mL) and cooled to 0 °C. Hydrogen chloride solution in anhydrous methanol was carefully added to tune *p*H value to be about 3~4. To a solution of sodium cyanoborohydride (14.0 mg, 0.22 mmol) in a mixture of solvents (methanol/THF 0.5 mL: 0.5 mL) was carefully added Hydrogen chloride solution in anhydrous methanol to tune *p*H value to be about 3~4. Then the solution of sodium cyanoborohydride was added to the solution of starting material. More Hydrogen chloride solution in anhydrous methanol was added to keep the *p*H value to be about 3~4. The reaction was stirred for 30 min at 0 °C. The solvent was removed *in vacuo* and the residue was used for the next step without further purification. The residue was dissolved in anhydrous ethanol (2.5 mL). To the solution was added sodium acetate trihydrate (425.0 mg, 3.13 mmol) and then allowed to reflux for 30 min. The reaction mixture was concentrated *in vacuo*. The residue was diluted with EtOAc (50 mL), washed with water (10 mL × 2) and then brine (10 mL). The organic layer was dried over Na_2SO_4, filtered and concentrated. To a solution of the crude product and $NaHCO_3$ (124.0 mg, 1.48 mmol) in CH_2Cl_2 (5.0 mL) was added Dess-Martin periodinane (94.0 mg, 0.22 mmol) at 0 °C under argon atmosphere. The reaction was kept stirring at 20 °C for about 0.5 h, and quenched by saturated aqueous $Na_2S_2O_3$ (2 mL). The mixture was diluted with CH_2Cl_2 (50 mL), washed with water (10 mL × 2) and then brine (10 mL). The organic layer was dried over Na_2SO_4, filtered and concentrated. The residue was purified by column chromatography on silica gel (Hexanes/EtOAc 8:1) to give compound **S15** (21.8 mg, 50%) as a colorless oil: R_f = 0.80 (Hexanes/EtOAc 2:1); ^1H NMR (400 MHz, $CDCl_3$) δ 4.40 (t, *J* = 5.2 Hz, 1H), 2.48 – 2.36 (m, 3H), 2.18 (dt, *J* = 10.0, 16.8 Hz, 1H), 2.04 (dt, *J* = 5.2, 10.4 Hz, 1H), 1.78 – 1.70 (m, 1H), 1.69 – 1.60 (m, 2H), 1.34 (td, *J* = 4.0, 8.0 Hz, 1H), 1.18 (dd, *J* = 4.4, 8.8 Hz, 1H), 0.88 (s, 9H), 0.83 (s, 3H), 0.64 (td, *J* = 5.6, 8.8 Hz, 1H), 0.06 (s, 3H), 0.02 ppm (s, 3H); ^{13}C NMR (100 MHz, $CDCl_3$) δ 212.3, 73.4, 62.1, 57.1, 41.6, 40.3, 30.5, 26.9, 25.9, 20.4, 20.2, 18.3, 11.3, –4.6, –5.2 ppm.

To a solution of compound **S15** (27.0 mg, 0.091 mmol) in THF (4.0 mL) was added LiHMDS (0.18 mL, 0.18 mmol, 1 M in THF) at –78 °C under argon atmosphere. The reaction was stirred at this

temperature for about 30 min, and a solution of $ZnCl_2$ (25.0 mg, 0.18 mmol) and ethyl pyruvate (20 μL, 0.18 mmol) in THF (2.0 mL) was added. The resulting mixture was stirred at this temperature for more 30 min and then quenched using saturated aqueous NH_4Cl. Evaporate THF away *in vacuo*. The residue was diluted with EtOAc (50 mL), washed with water (10 mL × 2) and then brine (10 mL). The organic layer was dried over Na_2SO_4, filtered and concentrated. To a solution of the crude product in Ac_2O (2.5 mL) was added *p*-TSA·H_2O (8.5 mg, 0.046 mmol) at 0 ^oC. The reaction was stirred at this temperature for 9 h, before it was quenched by adding $NaHCO_3$ and water until bubbling ceased. The mixture was extracted with EtOAc (30 mL × 3). The combined organic layers were dried over Na_2SO_4, filtered and concentrated under reduced pressure. To a solution of the crude product in THF (2.0 mL) was added DBU (0.25 mL) at ^oC. The resulting mixture was allowed warm to 20 ^oC and stirred overnight, before it was quenched with saturated NH_4Cl solution (5 mL). The layers were separated and the aqueous layer was extracted with EtOAc (30 mL × 3). The combined organic layers were dried over Na_2SO_4, filtered and concentrated. The residue was purified by column chromatography on silica gel (Hexanes/EtOAc 12:1) to give compound **S16** (14.3 mg, 45%) as a white solid: R_f = 0.40 (Hexanes/EtOAc 8:1); ^{1}H NMR (400 MHz, $CDCl_3$) δ 6.16 (s, 1H), 4.39 (t, *J* = 5.2 Hz, 1H), 2.54 – 2.42 (m, 2H), 2.14 (dt, *J* = 4.8, 12.0 Hz, 1H), 1.89 (s, 3H), 1.76 – 1.70 (m, 1H), 1.61 – 1.56 (m, 1H), 1.26 (dd, *J* = 4.0, 9.2 Hz, 1H), 1.09 (s, 3H), 0.91 (s, 9H), 0.73 (td, *J* = 5.6, 8.8 Hz, 1H), 0.10 (s, 3H), 0.07 ppm (s, 3H); ^{13}C NMR (100 MHz, $CDCl_3$) δ 171.4, 149.6, 149.4, 122.2, 121.3, 73.0, 62.0, 42.2, 29.2, 26.3, 26.0, 23.5, 21.6, 18.3, 12.3, 8.7, –4.6, –5.1 ppm.

Procedure for synthesis of (5a*S*,6a*S*,6b*R*)-3,6b-dimethyl-5-methylene-5a,6,6a,6b-tetrahydrocyclopropa[2,3]indeno[5,6-*b*]furan-2(5*H*)-one (S17):

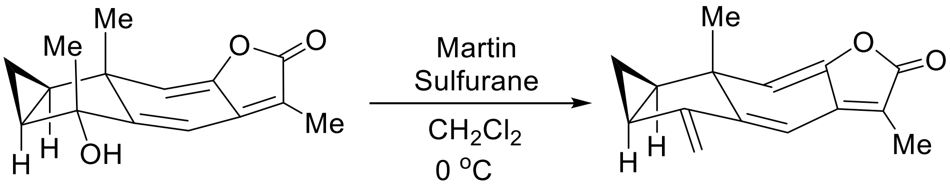

21 **S17**

A solution of Martin sulfurane dehydrating reagent (66.0 mg, 0.098 mmol) in anhydrous CH_2Cl_2 (1.0 mL) was added into a solution of compound **21** (12.0 mg, 0.049 mmol) in anhydrous CH_2Cl_2 (1.0 mL) at 0 ℃ under argon atmosphere. After stirring at this temperature for 5 min, the reaction was quenched with saturated aqueous $NaHCO_3$ (1.0 mL), diluted with EtOAc (30 mL), and washed with

water (10 mL), brine (10 mL). The organic layer was dried over Na_2SO_4, filtered and concentrated (some solvent left). The residue was purified by column chromatography on silica gel (the silica gel was rinsed with hexane containing 1% triethylamine before purification) (hexane/EtOAc 12:1). Eluent containing product was combined, added toluene and concentrated. The product was stored in toluene as a solution at –20 ℃. In order to collect NMR data, d_6-DMSO was added in to the solution and other solvents were removed by rotary evaporation and oil pump. R_f = 0.75 (hexane/EtOAc 4:1); ^{1}H NMR (400 MHz, DMSO) δ 6.64 (s, 1H), 6.28 (s, 1H), 5.63 (s, 1H), 5.28 (s, 1H), 2.19 – 2.12 (m, 1H), 1.93 (s, 3H), 1.78 (td, J = 7.4, 3.7 Hz, 1H), 1.10 – 0.99 ppm (m, 5H); ^{13}C NMR (100 MHz, DMSO) δ 171.2, 166.5 147.0, 146.8, 142.01, 116.0, 114.5, 110.0, 107.9, 45.8, 29.5, 24.2, 23.6, 17.0, 8.3 ppm; HRMS (ESI) m/z calcd for $C_{15}H_{14}O_2$ $[M+Na]^+$ 249.0886, found 249.0887.

Procedure for synthesis of Compounds *exo*-S18 and *exo*-S19:

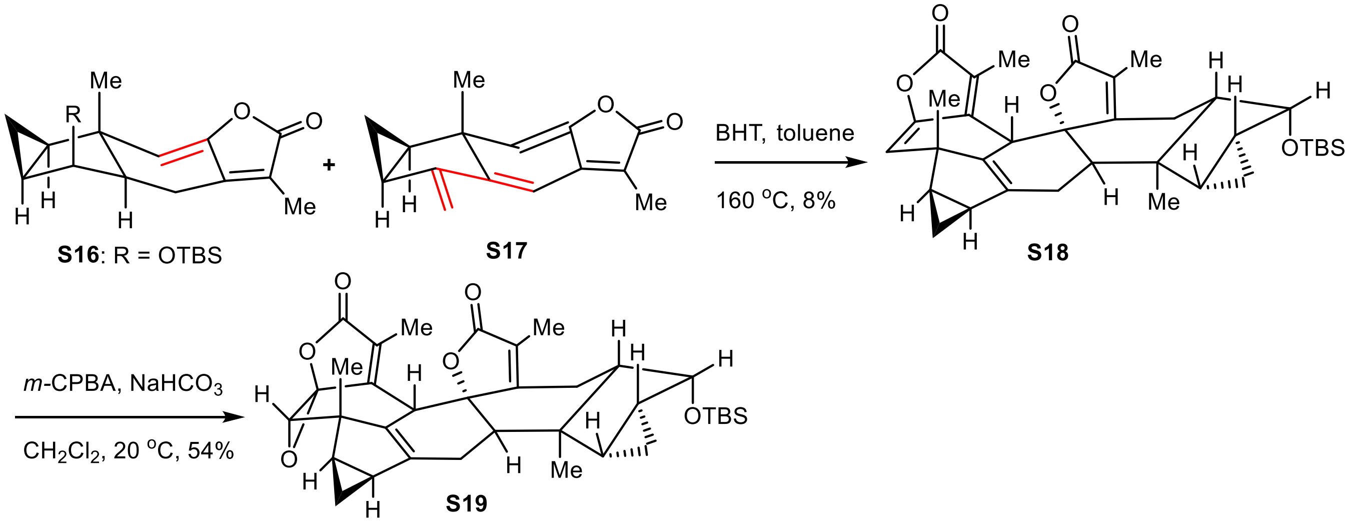

A solution of tetraene **S17** (from 19.6 mg precursor, 0.080 mmol), dienophile **S16** (33.3 mg, 0.0096 mmol) and BHT (trace amount) was heated at 170 ℃ in a sealed tube for 7 days. The reaction mixture was concentrated and the residue was purified by column chromatography on silica gel (12 g, hexane/EtOAc 12:1-5:1) to give *exo*-DA product **S18** (3.7 mg, 8%) as a pale yellow gum. To a solution of compound **S18** (10.0 mg, 0.017 mmol) and $NaHCO_3$ (14.7 mg, 0.17 mmol) in CH_2Cl_2 (1.8 mL) was added *m*-CPBA (17 mg, 70%, 0.069 mmol). The reaction was stirred at 20 ℃ for 70 min and quenched with saturated aqueous $Na_2S_2O_3$ (1.0 mL). The solution was diluted with EtOAc (50 mL), washed with saturated aqueous $NaHCO_3$ (10 mL), water (10 mL × 2) and brine (15 mL). The organic layer was dried

over Na_2SO_4, filtered and concentrated. The residue was purified by column chromatography on silica gel (5 g, hexane/EtOAc 4:1) to give the epoxide **S19** (5.4 mg, 54 %) as a white solid. Single crystal was obtained from CH_2Cl_2 solution.

S19: R_f = 0.25 (hexane/EtOAc 4:1); m.p.: decomposed over 250 °C; IR (film): ν = 3419, 2931, 2863, 1770, 1450, 1385, 1252, 1060 cm^{-1}; ^1H NMR (400 MHz, $CDCl_3$) δ 4.36 (t, J = 5.3 Hz, 1H), 3.74 (s, 1H), 3.45 (s, 1H), 2.76 (dd, J = 17.1, 12.9 Hz, 1H), 2.63 (dd, J = 17.1, 8.9 Hz, 1H), 2.55 – 2.46 (m, 1H), 2.31-2.15 (m, 2H), 2.12 (dd, J = 8.8, 6.2 Hz, 1H), 1.97 – 1.81 (m, 3H), 1.78 (s, 3H), 1.70 (s, 3H), 1.55 – 1.49 (m, 1H), 1.17 (dd, J = 8.8, 4.3 Hz, 1H), 1.03 (s, 3H), 0.92 (s, 9H), 0.89 (s, 1H), 0.85 (s, 3H), 0.62 (td, J = 8.8, 5.5 Hz, 1H), 0.34 (dd, J = 7.5, 3.9 Hz, 1H), 0.08 ppm (d, J = 8.1 Hz, 6H); ^{13}C NMR (100 MHz, $CDCl_3$) δ 172.4, 169.6, 162.9, 150.1, 143.7, 130.7, 129.3, 125.9, 88.7, 87.7, 72.0, 64.9, 55.3, 52.3, 50.3, 44.3, 38.7, 27.6, 27.4, 26.1, 26.0, 25.4, 24.7, 22.0, 20.6, 18.3, 16.5, 11.2, 10.7, 9.1, –4.4, –5.2 ppm; HRMS (ESI) m/z calcd for $C_{35}H_{44}O_6Si$ $[M+Na]^+$ 611.2799, found 611.2798.

Procedure for the preparation of (±)-9-*epi*-shizukaol A (30)

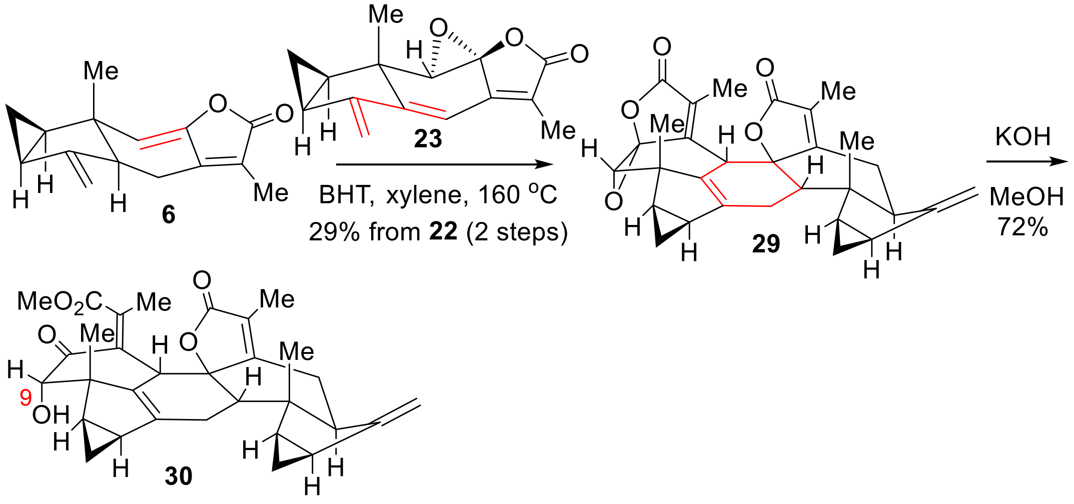

To a refluxing solution (160 °C oil bath) of dienophile **6** (700 mg, 3.07 mmol, 16.2 equiv.) and BHT (55 mg, 0.25 mmol, 1.32 equiv.) in xylene (10 mL) was added a solution of triene **23** (from 50 mg precursor, 0.19 mmol, 1.00 equiv.) in xylene (50 mL) via syringe pump (0.33 mL/h). After the addition, the reaction was refluxed for another 10 h. Then the solvent was evaporated using membrane pump and the residue was purified by column chromatography on silica gel (hexane/EtOAc 12:1–6:1) to give crude compound **29** (26.0 mg, 29% from **22**) as a pale-yellow oil: R_f = 0.30 (hexane/EtOAc 4:1), together with recovery of compound **6** (520 mg, 74%).

To a solution of crude compound **29** (40.2 mg, 0.085 mmol, 1.00 equiv.) in MeOH (10 mL) was

added KOH (9.6 mg, 0.17 mmol, 2.00 equiv.) at 20 °C. The reaction was stirred for 5 min then saturated aqueous NH_4Cl (1 mL) was added. The organic solvent was evaporated and the residue was diluted with EtOAc (50 mL). The organic layer was washed with water (20 mL) and brine (20 mL), then dried over Na_2SO_4, filtered and concentrated. The residue was purified by column chromatography on silica gel (hexane/EtOAc 4:1) to give compound **30** (30.8 g, 72%) as a colorless oil.

30: R_f = 0.20 (hexane/EtOAc 4:1); IR (film): ν = 3839, 3749, 3674, 3650, 2921, 2851, 1754, 1725, 1685, 1541, 1522, 1456, 1436, 1375, 1277, 1229, 1108, 1085, 996, 876, 799 cm^{-1}; ^1H NMR (500 MHz, $CDCl_3$) δ 4.92 – 4.85 (m, 1H), 4.59 (s, 1H), 3.83 (d, J = 3.7 Hz, 1H), 3.80 (s, 1H), 3.68 (s, 3H), 2.79 (dd, J = 16.2, 2.0 Hz, 1H), 2.57 (ddd, J = 16.3, 5.7, 4.0 Hz, 1H), 2.44 – 2.39 (m, 1H), 2.34 – 2.28 (m, 1H), 2.12 (dd, J = 17.7, 13.5 Hz, 1H), 1.94 – 1.90 (m, 2H), 1.89 – 1.84 (m, 2H), 1.81 (d, J = 1.7 Hz, 3H), 1.76 (d, J = 1.0 Hz, 3H), 1.55 (td, J = 7.8, 3.7 Hz, 2H), 1.17 (s, 3H), 0.92 (dt, J = 7.7, 3.9 Hz, 1H), 0.76 (td, J = 8.6, 5.3 Hz, 1H), 0.71 – 0.68 (m, 1H), 0.48 (s, 3H), 0.34 ppm (td, J = 4.4, 3.3 Hz, 1H); ^{13}C NMR (125 MHz, $CDCl_3$) δ 205.7, 173.8, 169.6, 166.0, 150.5, 141.6, 141.4, 138.3, 130.5, 123.5, 106.5, 92.6, 79.1, 59.6, 54.2, 52.7, 49.6, 42.3, 41.9, 25.6, 25.5, 25.4, 25.0, 23.6, 23.4, 22.7, 22.6, 18.5, 16.5, 16.3, 15.6, 8.7 ppm; HRMS (ESI) m/z calcd for $C_{31}H_{34}O_6$ $[M+Na]^+$ 525.2248, found 525.2263.

Total synthesis of (±)-shizukaol A (1):

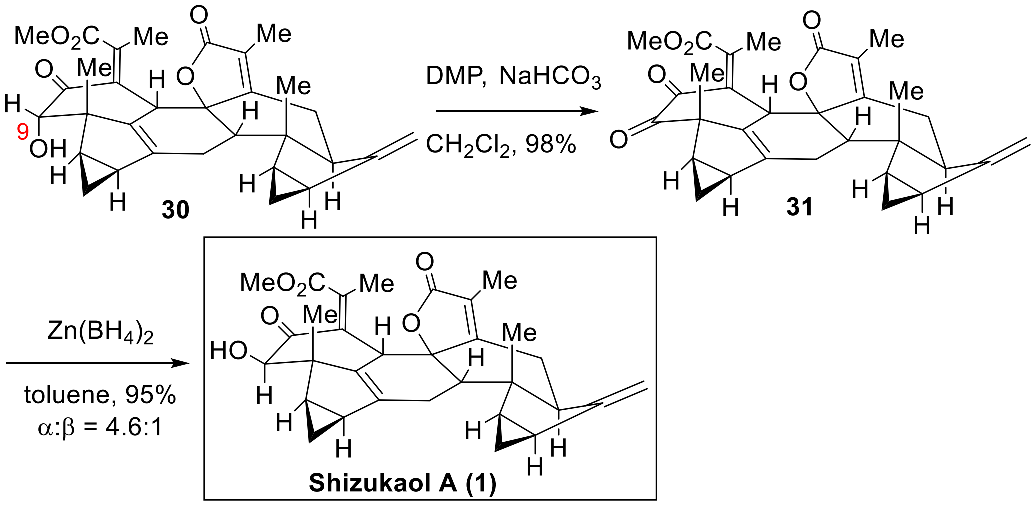

To a solution of compound **30** (7.2 mg, 0.014 mmol, 1.00 equiv.) and $NaHCO_3$ (9.6 mg, 0.11 mmol, 8.0 equiv.) in CH_2Cl_2 (2.0 mL) was added Dess-Martin periodinane (12.2 mg, 0.029 mmol, 2.00 equiv.). The reaction was stirred at room temperature for 30 min and quenched with saturated aqueous $Na_2S_2O_3$ (0.5 mL). The mixture was diluted with CH_2Cl_2 (30 mL) and washed with water (10 mL), brine (10 mL), then dried over Na_2SO_4, filtered and evaporated. The residue was purified by column chromatography

on silica gel (hexane/EtOAc 4:1) to afford dione **31** (7.0 mg, 98%) as a yellow oil.

31: R_f = 0.25 (hexane/EtOAc 4:1); IR (film): v = 3674, 3691, 2921, 2857, 1752, 1735, 1699, 1651, 1559, 1542, 1457, 1384, 1267, 1125, 1085 cm^{-1}; ^1H NMR (400 MHz, $CDCl_3$) δ 4.89 (s, 1H), 4.55 (s, 1H), 4.01 (d, *J* = 3.6 Hz, 1H), 3.74 (s, 3H), 2.82 (dd, *J* = 16.5, 2.1 Hz, 1H), 2.62 (ddd, *J* = 16.5, 5.6, 3.8 Hz, 1H), 2.51 (ddd, *J* = 8.1, 5.9, 4.5 Hz, 1H), 2.17 (d, *J* = 9.8 Hz, 2H), 2.00 (d, *J* = 9.5, 2.7 Hz, 1H), 1.97 – 1.89 (m, 2H), 1.87 (s, 3H), 1.83 (s, 3H), 1.81 – 1.76 (m, 1H), 1.40 (dt, *J* = 7.6, 3.9 Hz, 1H), 1.36 (s, 3H), 1.17 (td, *J* = 7.8, 4.5 Hz, 1H), 0.74 (td, *J* = 8.6, 5.4 Hz, 1H), 0.69 – 0.64 (m, 1H), 0.48 (s, 3H), 0.39 (td, *J* = 4.5, 3.0 Hz, 1H); ^{13}C NMR (125 MHz, $CDCl_3$) δ 192.0, 184.9, 173.5, 169.7, 165.1, 148.5, 148.3, 147.5, 134.3, 128.1, 124.1, 107.5, 92.5, 61.2, 61.1, 54.1, 53.1, 42.1, 41.6, 26.4, 25.5, 25.4, 24.0, 23.5, 22.8, 22.7, 19.4, 19.2, 17.5, 16.3, 8.7.; HRMS (ESI) *m/z* calcd for $C_{31}H_{32}O_6$ $[M+Na]^+$ 523.2091, found 523.2093.

To a solution of dione **31** (19.8 mg, 0.040 mmol, 1.00 equiv.) in toluene (3.0 mL) was added $Zn(BH_4)_2$ (3 drops, 1.0 M in THF) at 20 ℃ under Argon atmosphere. The reaction was stirred for about 5 min until the yellow color vanished. HOAc (0.2 mL, 1.0 M in EtOAc) was added to quench the excess reagent. The solvent was evaporated and the residue was purified by column chromatography on silica gel (hexane/EtOAc 4:1) to obtain **(±)-**shizukaol A (**1**) (3.3 mg, 17%) as a colorless oil, and its epimer **30** (15.5 mg, 78%).

(±)-Shizukaol A (1): R_f = 0.20 (hexane/EtOAc 4:1); IR (film): v = 2920, 2855, 1748, 1606, 1442, 1391, 1275, 1093, 1008, 808, 603 cm^{-1}; ^1H NMR (400 MHz, $CDCl_3$) δ 4.91 (dd, *J* = 2.9, 1.3 Hz, 1H), 4.58 (t, *J* = 2.2 Hz, 1H), 3.88 (d, *J* = 3.7 Hz, 1H), 3.87 (s, 1H), 3.80 (s, 3H), 3.21 (brs, 1H), 2.77 (dd, *J* = 16.4, 2.0 Hz, 1H), 2.63 (ddd, *J* = 16.2, 5.6, 4.0 Hz, 1H), 2.48 – 2.45 (m, 1H), 2.38 – 2.31 (m, 1H), 2.27 – 2.19 (m, 2H), 2.02 (ddd, *J* = 8.3, 5.9, 4.3 Hz, 1H), 1.91 (dd, *J* = 5.7, 2.1 Hz, 1H), 1.89 – 1.83 (m, 2H), 1.81 (s, 6H), 1.56 (td, *J* = 7.6, 3.8 Hz, 1H), 1.01 (s, 3H), 0.98 (ddd, *J* = 7.8, 7.8, 4.0 Hz, 1H), 0.82 (ddd, *J* = 8.5, 8.5, 5.4 Hz, 1H), 0.75 (ddd, *J* = 5.3, 5.3, 3.7 Hz, 1H), 0.50 (s, 3H), 0.30 (ddd, *J* = 4.3, 44.3, 2.9 Hz, 1H); ^{13}C NMR (125 MHz, $CDCl_3$) δ 200.1, 173.5, 170.9, 165.2, 148.9, 146.8, 142.2, 132.0, 131.9, 124.4, 107.2, 93.0, 79.6, 59.8, 54.5, 52.6, 51.1, 41.8, 40.9, 25.7, 25.6, 25.0, 24.9, 23.7, 23.1, 22.8, 20.1, 16.5, 15.8, 15.3, 8.6 ppm; HRMS (ESI) *m/z* calcd for $C_{31}H_{34}O_6$ $[M+Na]^+$ 525.2248, found 525.2245.

Supplementary Tables

Supplementary Table 1. Diels-Alder reaction between diene S9 and dienophile S10

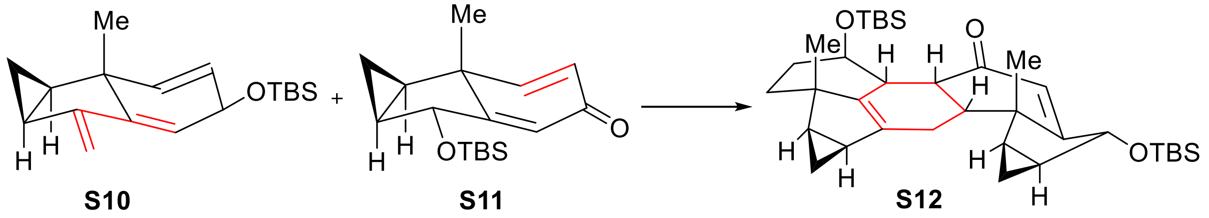

Entry	Condition	Result
1	$ZnCl_2$, CH_2Cl_2, –78 °C to 20 °C	only **S11** recovered
2	Eu(tfc)$_3$, CH_2Cl_2, –78 °C to 20 °C	**S10** & **S11** recovered
3	$AlEt_2Cl$, CH_2Cl_2, –78 °C to –45 °C	only **S11** recovered
4	20 °C, neat reaction, 10 days	only **S11** recovered
5	neat reaction, 110 °C, 22 h	trace product detected by TLC
6	BHT [a], sealed-tube, toluene 160 °C, 10 days [b]	dimers obtained in 76% (92% BRSM), dr = 1:14 (*endo:exo*) [c]

[a] BHT = butylated hydroxy-toluene; [b] temperature was optimized from 50 °C to 160 °C gradually; [c] ratio was detected by ^{1}H NMR spectrum

Supplementary Table 2. Diels-Alder reaction between diene S17 and dienophile S16

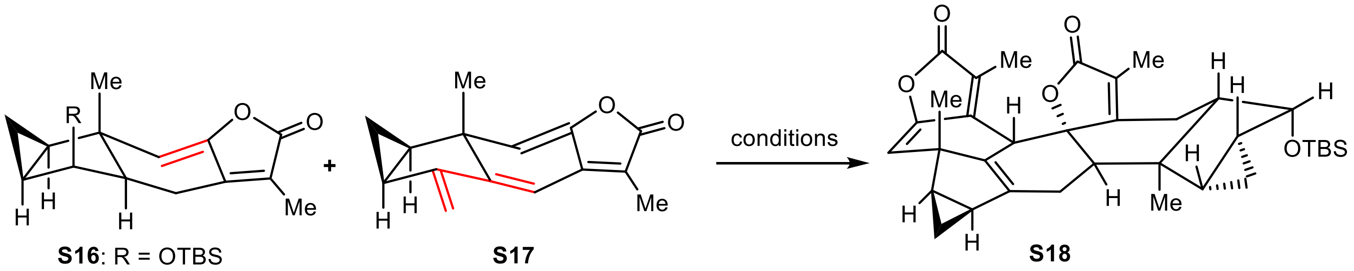

Entry	Conditions	Results
1	$ZnCl_2$, CH_2Cl_2, 20 °C	**S16** and **S17** were recovered
2	Eu(hfc)$_3$, CH_2Cl_2, 20 °C	**S16** and **S17** were recovered
3	$NHTf_2$, CH_2Cl_2, –78 °C to 20 °C	only **S16** recovered
4	20 °C, neat reaction, 10 days	only *endo*-dimer of **S12** with **S16** recovered
5	BHT [a], sealed-tube, toluene 160 °C, 7 days	8% **S18** with 41% dimer of **S17**
6	$LiClO_4$ (5 M), Et_2O, 20 °C	same with **entry 5** by TLC
7	$LiNTf_2$ (4 M), acetone, 20 °C	same with **entry 5** by TLC

[a] BHT = butylated hydroxy-toluene

Supplementary Table 3 Optimization of reduction of **27**

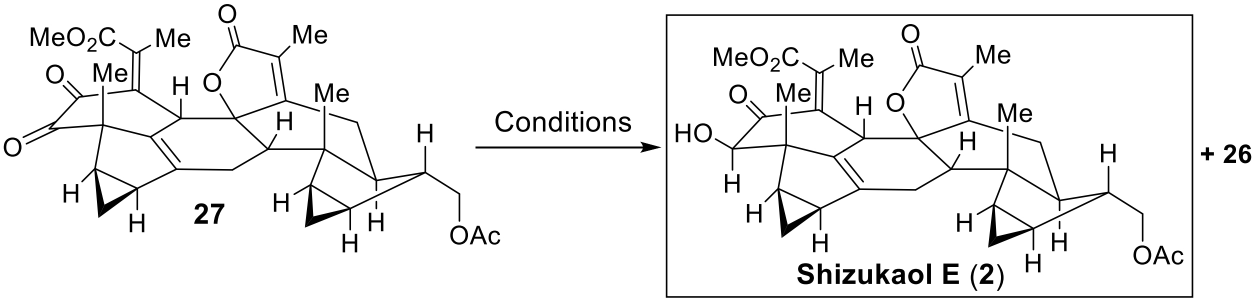

Entry	Conditions	dr (α: β)
1	$NaBH_4$, MeOH, 20 °C	only α compoud found
2	LiAl(*t*BuO)$_3$H, THF, 20 °C	only α compoud found
3	L-Selectride, toluene, 20 °C	decomposed
4	$Zn(BH_4)_2$, THF, 20 °C	5.6:1-3.5:1
5	$Zn(BH_4)_2$, toluene, 20 °C	2.8 : 1
6	$Zn(BH_4)_2$, toluene, –78 - –50 °C	3.4 : 1
7	Al(O*i*Pr)$_3$, *i*PrOH, toluene, 50 °C	no reaction

The dr value was detected by ^{1}H NMR

Supplementary Table 4 Optimization of reduction of **31**

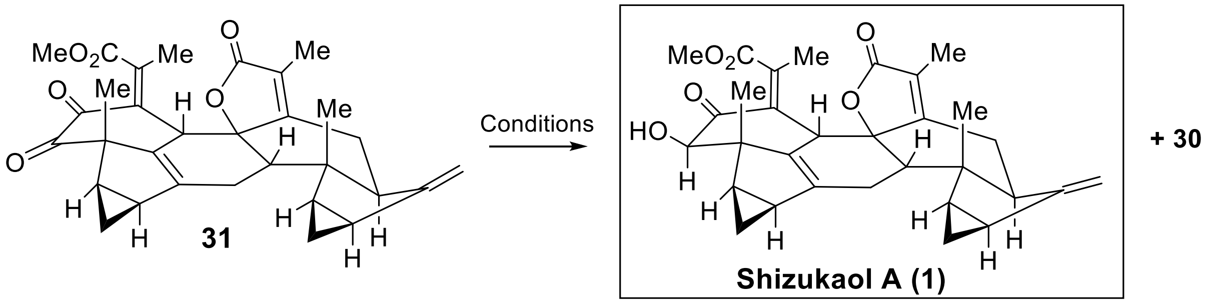

Entry	Conditions	dr (α: β)
1	$NaBH_4$, MeOH, 20 ^oC	10.6:1
2	KBH_4, MeOH, 20 ^oC	7.3:1
3	$Zn(BH_4)_2$, MeOH, 20 ^oC	decomposed
4	$Zn(BH_4)_2$, CH_2Cl_2, 20 ^oC	7.2:1
5	$Zn(BH_4)_2$, toluene, 20 ^oC	4.6 : 1

The dr value was detected by ^{1}HNMR

Supplementary Table 5 Comparisons of ^{1}H NMR data of natural and synthetic shizukaol E (**2**)[5]

	^{1}H NMR of shizukaol E		
	Synthetic (400 MHz, $CDCl_3$)	Natural (500 MHz, $CDCl_3$)	Δ δ
1 CH	2.04 ddd (8.2, 5.8, 4.4)	2.04 ddd (7.4, 5.5, 3.9)	0.00
2 CH_2	α: 0.98 ddd (7.5, 7.5, 3.4)<br>β: 0.27 ddd (4.2, 4.2, 3.1)	α: 0.98 ddd (7.4, 7.1, 3.2)<br>β: 0.27 ddd (3.9, 3.4, 3.2)	0.00<br>0.00
3 CH	1.84 dd (7.3, 2.8)	1.83 ddd (7.1, 5.5, 3.4)	0.01
4 C			
5 C			
6 CH	3.87 d (3.6)	3.87 brd (3.3)	0.00
7 C			
8 C			
9 CH	4.04 s	4.05 s	−0.01
10 C			
11 C			
12 C			
13 CH_3	1.80 s	1.80 s	0.00
14 CH_3	1.01 s	1.01 s	0.00
15 CH_2	α: 2.71 dd (16.4, 1.6)<br>β: 2.61 ddd (16.5, 5.7, 3.9)	α: 2.72 dd (16.5, 1.7)<br>β: 2.61 ddd (16.5, 5.7, 3.3)	−0.01<br>0.00
1' CH	1.41 ddd (8.4, 8.2, 4.0)	1.41 ddd (8.3, 8.1, 4.2)	0.00
2' CH_2	α: 0.75 ddd (8.4, 8.4, 5.5)<br>β: 0.83 m	α: 0.76 ddd (8.3, 8.2, 5.4)<br>β: 0.83 ddd (5.4, 4.2, 4.2)	−0.01<br>0.00
3' CH	1.09 dddd (8.1, 8.1, 3.8, 3.8)	1.09 dddd (8.2, 8.1, 4.2, 3.2)	0.00
4' C(H)	1.56 m	1.57 dddd (11.1, 8.0, 6.8, 3.2)	−0.01
5' CH	1.75 m	1.75 ddd (13.4, 11.1, 6.1)	0.00
6' CH_2	α: 2.40 ddq (18.1, 6.1, 2.1)<br>β: 2.24 dd (17.8, 13.3)	α: 2.40 ddq (18.0, 6.1, 2.0)<br>β: 2.25 dd (18.0, 13.4)	0.00<br>−0.01
7' C			
8' C			
9' CH	1.81 dd (6.0, 2.0)	1.82 dd (5.7, 1.7)	−0.01
10' C			
11' C			
12' C			
13' CH_3	1.80 s	1.80 br s	0.00
14' CH_3	0.61 s	0.61 s	0.00
15' CH_2	3.77 dd (11.1, 8.0)<br>3.95 dd (11.1, 6.8)	3.77 dd (11.0, 8.0)<br>3.95 dd (11.0, 6.8)	0.00<br>0.00
OMe	3.78 s	3.79 s	−0.01
OAc	2.08 s	2.09 s	−0.01
OH	3.35 s		

Supplementary Table 6 Comparison of ^{13}C NMR data of natural and synthetic shizukaol E (**2**)[3]

	^{13}C NMR of shizukaol E		
	Synthetic (125 MHz, $CDCl_3$)	Natural (125 MHz, $CDCl_3$)	Δ δ
1	25.4	25.4	0.0
2	15.8	15.8	0.0
3	24.7	24.7	0.0
4	142.3	142.3	0.0
5	131.5	131.5	0.0
6	40.5	40.5	0.0
7	131.8	131.8	0.0
8	200.6	200.6	0.0
9	80.1	80.1	0.0
10	51.2	51.2	0.0
11	147.2	147.1	0.1
12	170.7	170.8	−0.1
13	20.3	20.3	0.0
14	15.1	15.1	0.0
15	25.6	25.6	0.0
1'	24.3	24.3	0.0
2'	16.6	16.6	0.0
3'	21.8	21.8	0.0
4'	43.0	43.0	0.0
5'	59.3	59.3	0.0
6'	25.0	25.0	0.0
7'	165.5	165.5	0.0
8'	92.5	92.5	0.0
9'	54.7	54.7	0.0
10'	44.0	44.0	0.0
11'	124.2	124.2	0.0
12'	173.4	173.4	0.0
13'	8.6	8.6	0.0
14'	23.9	23.9	0.0
15'	66.2	66.2	0.0
OMe	52.6	52.6	0.0
OAc	171.0<br>20.8	171.0<br>20.8	0.0<br>0.0

<u>Supplementary Table 7</u> Comparison of ^{1}H NMR data of natural and synthetic shizukaol A (**1**)[6]

	^{1}H NMR of shizukaol A		
	Synthetic (400 MHz, $CDCl_3$)	Natural (500 MHz, $CDCl_3$)	Δ δ
1 CH	2.02 ddd (8.3, 5.9, 4.3)	2.03 ddd (7.7, 5.6, 4.3)	−0.01
2 CH_2	α: 0.98 ddd (7.8, 7.8, 4.0)<br>β: 0.30 ddd (4.3, 4.3, 2.9)	α: 0.99 ddd (7.8, 7.7, 3.4)<br>β: 0.30 ddd (4.3, 4.3,3.4)	−0.01<br>0.00
3 CH	1.86 m	1.86 ddd (7.8, 5.6, 3.4)	0.00
4 C			
5 C			
6 CH	3.88 d (3.7)	3.88 d (3.4)	0.00
7 C			
8 C			
9 CH	3.87 s	3.87 s	0.00
10 C			
11 C			
12 C			
13 CH_3	1.81 s	1.82 s	−0.01
14 CH_3	1.01 s	1.01 s	0.00
15 CH_2	α: 2.77 dd (16.4, 2.0)<br>β: 2.63 ddd (16.2, 5.6, 4.0)	α: 2.77 dd (16.2, 1.7)<br>β: 2.63 ddd (16.2, 5.6, 3.4)	0.00<br>0.00
1' CH	1.56 td (7.6, 3.8)	1.57 dd (7.8, 7.3)	0.00
2' CH_2	α: 0.82 ddd (8.5, 8.5, 5.4)<br>β: 0.75 ddd (5.3, 5.3, 3.7)	α: 0.82 ddd (7.8, 7.8, 4.6)<br>β: 0.76 ddd (4.6, 4.6, 3.4)	0.00<br>−0.01
3' CH	1.86 m	1.86 ddd (7.8,7.3, 4.6)	0.00
4' C(H)			
5' CH	2.46 m	2.46 dd (13.3, 6.0)	0.00
6' CH_2	α: 2.26 m<br>β: 2.21 m	α: 2.27 dd (17.5, 6.0)<br>β: 2.21 ddq (17.5, 13.3, 1.7)	−0.01<br>0.00
7' C			
8' C			
9' CH	1.91 dd (5.7, 2.1)	1.91 dd (5.6,1.7)	0.00
10' C			
11' C			
12' C			
13' CH_3	1.81 s	1.82 s	−0.01
14' CH_3	0.50 s	0.51 s	−0.01
15' CH_2	4.58 t (2.2)<br>4.91 dd (2.9, 1.3)	4.58 m<br>4.91 m	0.00<br>0.00
OMe	3.80 s	3.80 s	0.00
OH	3.21 brs		

Supplementary Table 8 Comparison of ^{13}C NMR data of natural and synthetic shizukaol A (**1**)[4]

	^{13}C NMR of shizukaol A		
	Synthetic (125 MHz, $CDCl_3$)	Natural (125 MHz, $CDCl_3$)	Δ δ
1	25.6	25.7	−0.1
2	15.8	15.8	0.0
3	24.9	24.9	0.0
4	142.2	142.2	0.0
5	132.0	132.1	−0.1
6	40.9	41.0	−0.1
7	132.0	132.0	0.0
8	200.1	200.1	0.0
9	79.6	79.7	−0.1
10	51.1	51.2	−0.1
11	146.8	146.8	0.1
12	170.9	170.9	0.0
13	20.1	20.1	0.0
14	15.3	15.3	0.0
15	25.6	25.6	0.0
1'	23.7	23.7	0.0
2'	16.5	16.5	0.0
3'	22.8	22.9	−0.1
4'	148.9	149.0	−0.1
5'	59.8	59.8	0.0
6'	23.1	23.1	0.0
7'	165.2	165.2	0.0
8'	93.0	93.0	0.0
9'	54.5	54.5	0.0
10'	41.8	41.9	−0.1
11'	124.4	124.4	0.0
12'	173.5	173.5	0.0
13'	8.6	8.6	0.0
14'	25.0	25.0	0.0
15'	107.2	107.2	0.0
OMe	52.6	52.6	0.0

Computational Methods and Results

Density Functional theory (DFT) calculations were carried out using Gaussian09 program.[7] For Diels-Aldel reaction, geometry optimizations were performed with dispersion-corrected density functional method M06-2X functional[8-10] and a double-ζ valence 6-31G(d) basis set in gas phase. Single point energies with a larger triple-ζ valence polarized 6-311+G(d,p) basis set and SMD continuum solvation model[11] with intrinsic atomic Coulomb radii were evaluated to include the effect of xylene (xylene-mixture with ε=2.3879 used) on the computed Gibbs energy profile.

For $Zn(BH_4)_2$ reduction of dione **31**, for comparison reasons, geometry optimizations were carried out with B3LYP[12-14], M06-2X and M06 functionals separately[8-10]. For all these three functional, LANL2DZ basis set [15] with effective core potential (ECP) and associated double-ζ valence basis set was used for Zn, and the 6-31G(d,p) basis set was used for other atoms. Single-point energies were evaluated with M06-2X functional (or M06 for the structures optimized with M06 functional) with triple-ζ valence polarized 6-311+G(d,p) basis set for all non-metal atoms and SDD ECP[16,17] for Zn with SMD continuum solvation model[11] in toluene (ε=2.3741) with intrinsic atomic Coulomb radii. Both local minima and transition structures are confirmed by vibrational frequencies with 0 and 1 imaginary frequency, respectively. All transition structures are checked by intrinsic reaction coordinate (IRC) calculation. Images were prepared using CYLview[18].

Computational study of Diels-Alder reaction between diene 23 and ethylene 6.

To better explain the observed stereochemical outcome of the *endo*-Diels-Alder reaction between diene **23** and ethylene **17** and **6**, we carried out computational studies of diene **23** and ethylene **6**. Due to the existence of the C14' methyl group on ethylene **6**, the diene **23** is expected to approach from the opposite side of the C14' methyl group (the *Si* face of C9') more easily than approaching from the same side of the C14' methyl group (the *Re* face of C9'), and this speculation is supported by our calculation. As shown in **Supplementary Figure 75** and **Supplementary Figure 76** , four possible transition states for the reaction from the *Si* face of C9' on ethylene **6** labelled **C9'*Si*-TS-*endo*-*Si***, **C9'*Si*-TS-*endo*-*Re***, **C9'*Si*-TS-*exo*-*Si*** and **C9'*Si*-TS-*exo*-*Re***(with the second *Si/Re* defined using C6 center of diene **23)**, and

the other five TSs for the reaction from the *Re* face of C9', labelled **C9'*Re*-TS-*endo-Si***, **C9'*Re*-TS-*endo-Re***, **C9'*Re*-TS-*exo-Si1***, **C9'*Re*-TS-*exo-Si2*** and **C9'*Re*-TS-*exo-Re***, were located, with the C9'*Si* TSs (**Supplementary Figure 75**) generally having lower Gibbs free energy than C9'*Re* TSs (**Supplementary Figure 76**). And among the 9 TSs, the activation Gibbs free energy for the *endo*-type Diels-Alder reaction at the *Re* face (defined using C6 center) of diene **23** via transition state **C9'*Si*-TS-*endo-Re*** is 27.6 kcal mol^{-1}, lower than all the other TSs; and the corresponding product **29** is formed with 10.9 kcal mol^{-1} exergonic. In a series of papers, Domingo *et al.* have investigated a variety of reactions and proposed that Diels–Alder reactions could be classified as non-polar (*N*), polar (*P*), and ionic (*I*), depending on electrophilicity and nucleophilicity of the reagents[19]. And the selectivity is controlled by the favorable electrostatic interactions taking place at the TSs[20]. A review publish by Houk *et al.* also summarized and highlighted the work in this field[21]. Using similar approach, we could calculate the global electrophilicity indexes[22] (ω, a measure of the stabilization energy when the system acquires additional electronic charges from the environmental) of diene **23** and ethylene **6** as 1.48 eV and 1.27 eV respectively (**Supplementary Table 9**), with the electrophility of **23** higher than that of **6**. Global electron density transfer (GEDT) in these TSs, carried out with Natural Bond Orbital (NBO 6.0)[23] revailed that the electron transfer is in the range of 0 to 0.05 e in different TSs (**Supplementary Table 10**), indicating a low polar character in the Diels-Alder reaction. The high temperature (i.e. 160 ℃ in xylene) needed for this Diels-Alder reaction is in agreement with the low polar character of the reaction. The differences between the lengths of the two forming σ bonds in these TSs varies from 0.17 Å (in **C9'*Re*-TS-*endo-Re***) to 1.35 Å (in **C9'*Re*-TS-*endo-Si*1).** For instance, the bond distance of the forming C15-C9' bond in **C9'*Si*-TS-*endo-Re*** is shorter by 0.85 Å than that of the forming C6-C8' bond, indicating C15, C9' carbons have stronger interaction in the transition state and this is supported by the distortion model [24,25] analysis (**Supplementary Table 11** and **Supplementary Figure 77**), where the

interaction energy (as evaluated by M06-2X/6-31G(d) in gas phase) was found to be 25.5 kcal mol^{-1}, the highest among all the TSs, meanwhile, the distortion energies for both diene **23** and ethylene **6** in **C9'*Si*-TS-*endo*-*Re*** were found to be the lowest in these TSs. All other TSs in **Supplementary Figures 75** and **76** containing this C9'-C15 interaction have relatively lower energy than the ones without this bond formation, as well as a shorter C9'-C15 bond distances, comparing to the other σ bond formed in the same TS. The large differences between the distances of the two pairs of interacting centers and the IRC calculations from these TSs to cycloadducts suggest that the reaction undergoes in non-concerted two-stage one-step mechanism[26], with one σ bond formed before the other σ bond starts to form in the TSs, although with different degree of asynchronicity, except **C9'*Re*-TS-*endo*-*Si*** for which the reaction can be seen as a two-step process, with two TSs, i.e. **C9'*Re*-TS-*endo*-*Si1* *and* C9'*Re*-TS-*endo*-*Si2*** being located.

Furthermore, steric repulsion is also a factor of the origin of the difference in the Gibbs free energy of these TSs. For instance, in **Supplementary Figure 75**, all other transition states have higher energy than **C9'*Si*-TS-*endo*-Re** by at least 9.6 kcal mol^{-1} (see **C9'*Si*-TS-*exo*-*Si***). Especially in **C9'*Si*-TS-*endo*-*Si*** where the C14 on the diene **23** points towards the ethylene **6** which causes steric repulsion, the Gibbs free energy is 48.8 kcal mol^{-1}. When that steric repulsion is avoided in transition state **C9'*Si*-TS-*exo*-*Re***, the Gibbs free energy is 39.2 kcal mol^{-1}. The relative Gibbs free energy of transition state **C9'*Si*-TS-*exo*-*Si*** is the second lowest among these four, i.e. 37.2 kcal mol^{-1}, because in this structure, the C14 methyl group cannot cause much steric repulsion. Therefore, our theoretical calculations revealed that the desired DA product was generated from the transition state **C9'*Si*-TS-*endo*-*Re*** with an activation Gibbs free energy of 27.6 kcal mol^{-1}.We suggest that the relatively low Gibbs free energy in **C9'*Si*-TS-*endo*-*Re*** is a result of the favorable interaction between C9' and C15 and the steric freedom in this TS which allows such favorable interaction to be at its greatest extent.

Supplementary Figures and Tables for Computational Results

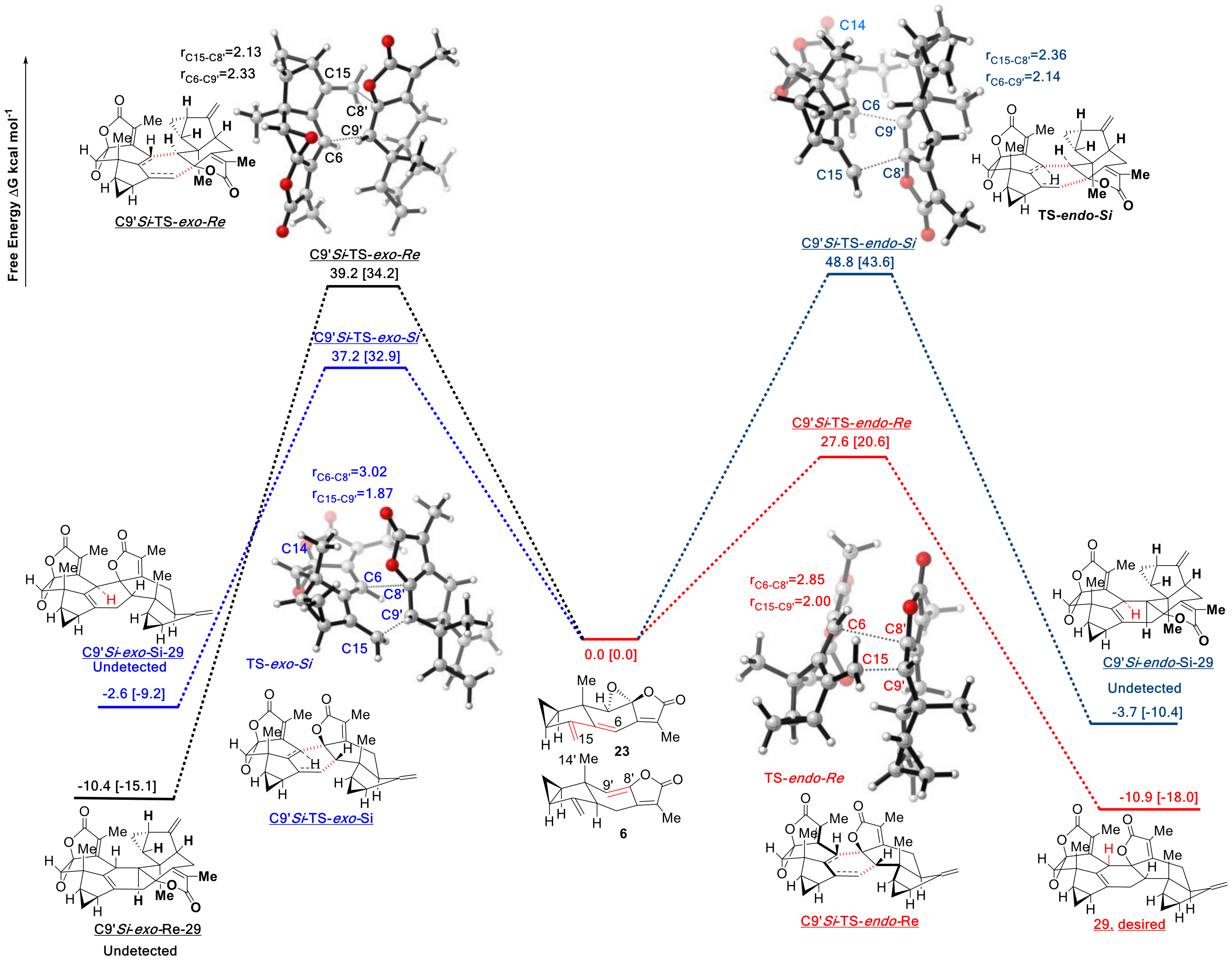

Supplementary Figure 75 | Computed free energy profile for the Diels-Alder reaction between diene 23 and ethylene 6. TSs with 23 approaches from the opposite side (C9' *Si* face) of the C14' methyl group on 6. The relative free energies in Xylene given in kcal mol^{-1} are calculated by M06-2X/6-311+G(d,p) // M06-2X/6-31G(d) using SMD solvation model. Free energies calculated in gas phase are quoted in square brackets. Bond distance in Å. Molecular graphics were produced by CYLview.

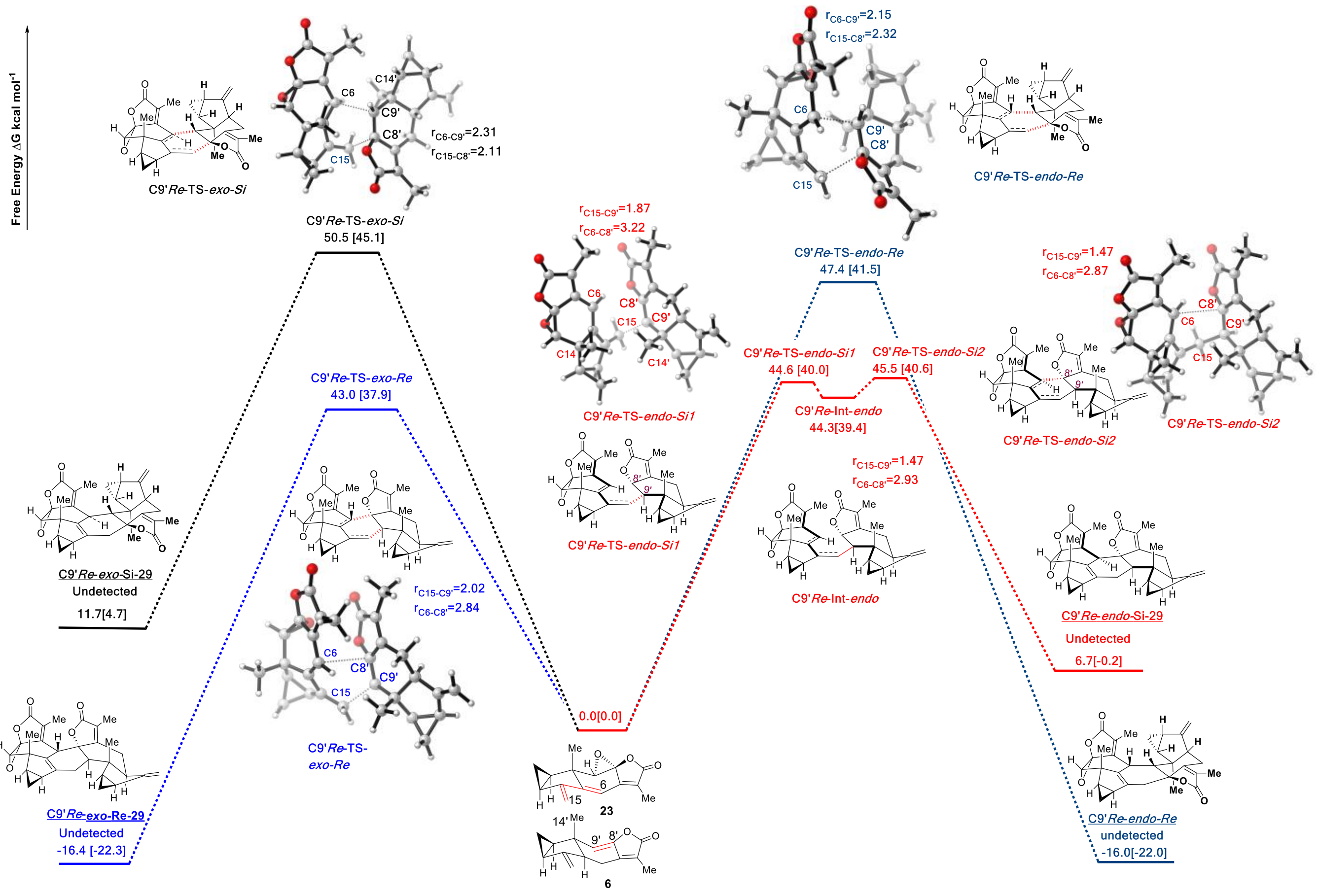

Supplementary Figure 76 | Computed free energy profile for the Diels-Alder reaction between diene 23 and ethylene 6. TSs with 23 approaches from the same side (C9' Re face) of the C14' methyl group on 6. The relative free energies in Xylene given in kcal mol^{-1} are calculated by M06-2X/6-311+G(d,p) // M06-2X/6-31G(d) using SMD solvation model. Free energies calculated in gas phase are quoted in square brackets. Bond distance in Å. Molecular graphics were produced by CYLview.

Supplementary Table 9 Electronic chemical potential (μ), chemical hardness (η), global electrophilicity index (ω) for diene **23** and ethylene **6**.

	ε_H/au	ε_L/au	μ/au	η/au	ω/eV[a]
diene **23**	-0.0421	-0.27792	-0.16001	0.23582	1.48
ethylene **6**	-0.0277	-0.27929	-0.1535	0.25159	1.27

[a]The global electrophilicity index, ω, is calculated using $\omega = (\mu^2/2\eta)$ (eV), where μ is the electronic chemical potential $\mu = (\varepsilon_H + \varepsilon_L)/2$ and chemical hardness $\eta = (\varepsilon_L - \varepsilon_H)$.

Supplementary Table 10 Global electron density transfer (GEDT) from the diene **23** to ethylene **6** at the corresponding TSs in the Diels–Alder reaction of diene **23** and ethylene **6** and the difference between the lengths of the two forming σ bonds in the reaction, i.e. Δ*d*=dist1 – dist2.

	GEDT	dist1/Å	dist2/Å	Δ*d*/Å
**C9'*Si*-TS-*endo*-*Re***	0.01	2.85	2.00	0.85
**C9'*Si*-TS-*exo*-*Re***	0.02	2.33	2.13	0.20
**C9'*Si*-TS-*endo*-*Si***	0.05	2.36	2.14	0.22
**C9'*Si*-TS-*exo*-*Si***	0.05	3.02	1.87	1.15
**C9'Re-TS-endo-Si1**	0.06	3.22	1.87	1.35
**C9'*Re*-TS-*exo*-*Si***	0.04	2.31	2.11	0.20
**C9'*Re*-TS-*endo*-*Re***	0.01	2.32	2.15	0.17
**C9'*Re*-TS-*exo*-*Re***	–0.01	2.84	2.02	0.82

Supplementary Table 11 M062X/6-31G(d) energetics for Diels–Alder reactions of diene **23** and ethylene **6** in kcal mol^{-1}.

	E_{act}	H_{act}	G_{act}	E_{rxn}	G_{rxn}	E_{dist_diene}	$E_{dist\_ethylene}$	E_{dist}	$E_{interaction}$
**C9'*Si*-TS-*endo*-*Re***	2.3	3.0	20.6	-39.1	-18.0	14.6	13.1	27.7	25.5
**C9'*Si*-TS-*exo*-*Re***	17.3	17.8	34.2	-35.0	-15.1	18.3	17.5	35.9	18.6
**C9'*Si*-TS-*endo*-*Si***	26.8	27.2	43.6	-31.8	-10.4	28.0	18.9	46.8	20.1
**C9'*Si*-TS-*exo*-*Si***	15.9	15.6	32.9	-30.2	-9.2	17.2	15.8	33.1	17.2
**C9'Re-TS-endo-Si1**	24.4	25.1	40.0	21.8	39.4	24.9	24.4	49.3	24.9
**C9'*Re*-TS-*exo*-*Si***	28.0	28.4	45.1	-17.4	4.7	21.7	23.8	45.5	17.5
**C9'*Re*-TS-*endo*-*Re***	24.1	25.0	41.5	-42.6	-22.0	18.7	18.0	36.7	12.6
**C9'*Re*-TS-*exo*-*Re***	20.1	20.5	37.9	-43.5	-22.3	19.3	22.3	41.6	21.6

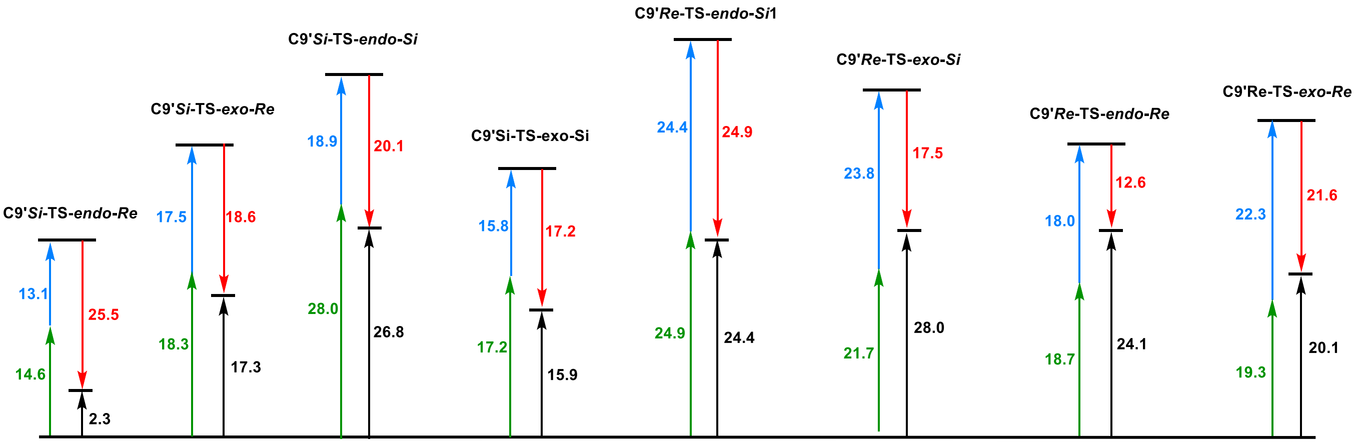

Supplementary Figure 77 | Activation, distortion, and interaction energies (M062X/6-31G(d): green: diene distortion energy, blue: ethylene distortion energy, red: interaction energy, and black: activation energy (kcal mol^{-1}).

Computational study of $Zn(BH_4)_2$ reduction of dione 31.

DFT calculation revealed that the reaction occurs with $Zn(BH_4)_2$ coordinated on the two carbonyl groups at C8 and C9 of dione **31**. The transition state, **TS-β-OH**, leading to the desired reduced product with β-OH at C9, *i.e*. Shizukaol A (**1**), was found to be higher in free energy than that led to the product with α-OH at C9 by 0.5 kcal mol^{-1} (with M06), 1.1 kcal mol^{-1} (with M06-2X) and 2.1 kcal mol^{-1} (with b3lyp) (-0.3, 1.4 and 1.2 kcal mol^{-1} in gas phase respectively) (see detailed information tin **Supplementary Table 10**). In both TSs, Zn is coordinated on the O8 and O9 oxygens, and the bond distances between C9 and $H(BH_3)$ are 1.55 Å and 1.47 Å in **TS-α-OH** and **TS-β-OH** respectively as predicted by M06 functional (**Supplementary Figure 78**). Both TSs led to an intermediate **Int-α/β-OH-2** with C9-H(BH3) bond formed while the reacting boron is only bonded to O9 weakly (see the bond lengths in **Supplementary Figure 78**), and when this boron is bonded to O9 strongly with r_{O9-B} 1.52 and 1.54 in **Zn-β-OH** and **Zn-α-OH** respectively, the Gibbs free energy is lowered to -23.3 and -18.6 respectively. It is worth to mention that, with b3lyp functional, **TS-β-OH** led to a product complex with C9H-BH_3 bond broken and BH_3 coordinated on O9 directly.

Supplementary Table 12 Corrected Gibbs free energies for the transition states and intermediates for $Zn(BH_4)_2$ reduction of dione **31** as predicted by B3lyp[a], M06-2X[b] and M06[c] functionals. Free energies calculated in gas phase are quoted in square brackets.

	**B3lyp[a]**			**M06-2X[b]**			**M06[c]**		
	**α-OH**	**β-OH**		**α-OH**	**β-OH**		**α-OH**	**β-OH**	
**Int-α/β-OH-1**	-1.6<br>[7.3]	-0.1<br>[7.7]		-2.8<br>[-9.8]	6.0<br>[0.5]		-2.0<br>[-2.2]	-2.9<br>[-1.5]	
**TS-α/β-OH**	12.4<br>[21.4]	14.5<br>[22.6]	ΔΔG≠=<br>2.1[1.2]	11.5<br>[0.0]	12.6<br>[1.4]	ΔΔG≠=<br>1.1[1.4]	13.7<br>[7.6]	14.2<br>[7.3]	ΔΔG≠=<br>0.5[-0.3]
**Int-α/β-OH-2**	6.5<br>[16.1]	NA[d]		3.6<br>[-8.6]	6.9<br>[-1.4]		4.1<br>[-1.5]	6.7<br>[3.9]	
**Zn-α/β-OH-3**	-20.6<br>[-7.5]	-24.9<br>[-13.1]		-25.0<br>[-38.6]	-28.1<br>[-37.5]		-18.6<br>[-27.1]	-23.3<br>[26.6]	

[a]Computed SMD(toluene)-M06-2X/6-311+G(d,p)-SDD // b3lyp/6-31G(d,p)-LANL2DZ) Gibbs free energy relevant to $Zn(BH_4)_2$ and starting dione **31** is quoted; [b] Computed SMD(toluene)-M06-2X/6-311+G(d,p)-SDD // M06-2X/6-31G(d,p)-LANL2DZ Gibbs free energy relevant to $Zn(BH_4)_2$ and starting dione **31** is quoted; [c] Computed SMD(toluene)-M06/6-311+G(d,p)-SDD // M06/6-31G(d,p)-LANL2DZ) Gibbs free energy relevant to $Zn(BH_4)_2$ and starting dione **31** is quoted; [d] optimization of the IRC forward structure led to **Zn-β-OH** directly.

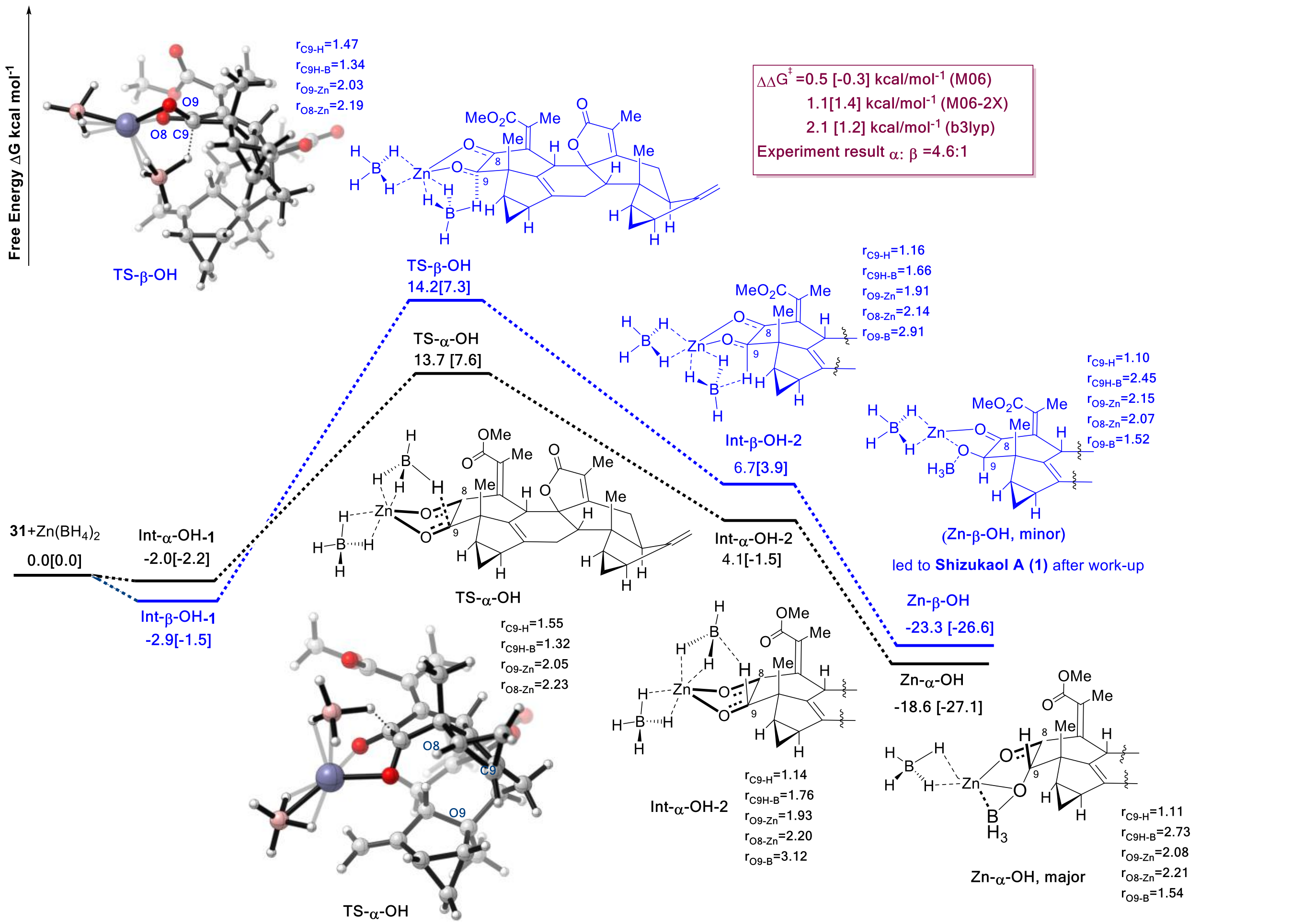

Supplementary Figure 78 | Transition state geometry and energy profile for $Zn(BH_4)_2$ reduction of dione 31. Computed (M06/6-31G(d,p)-LANL2DZ) transition structures and intermediates for $Zn(BH_4)_2$ reduction of **31**. The relative free energies in toluene given in kcal mol^{-1} are calculated by M06/6-311+G(d,p)-SDD // M06/6-31G(d, p)-LANL2DZ using SMD solvation model. Gibbs free energies calculated in gas phase are quoted in square brackets. Bond distance in Å. Molecular graphics were produced by CYLview. The $\Delta\Delta G^{\ddagger}$ is calculated with the details showing in **Table S12**.

Supplementary Table 13 Energies, enthalpies, and free energies (in Hartrees) of optimized structures for the Diels-Alder reaction between diene **23** and ethylene **6** calculated at M06-2X/6-311+G(d,p)-SMD(Xylene-mixture) // M06-2X/6-31G(d) level of theory.

Structure	E^a	ZPE^b	H^c	G^d	$E_{xylene}{}^e$	Imaginary Frequency (cm^{-1})
**Diene 23**	-805.2852222	0.259014	-805.010304	-805.067969	-805.522484	
**6**	-731.3133876	0.278393	-731.019632	-731.075717	-731.5279084	
**C9'*Si*-TS-*endo*-*Re***	-1536.595007	0.539946	-1536.025193	-1536.11079	-1537.035708	-485.59
**29**	-1536.660982	0.545084	-1536.086625	-1536.172375	-1537.101449	
**C9'*Si*-TS-*exo*-*Re***	-1536.571016	0.539186	-1536.001525	-1536.089235	-1537.014797	-534.63
**C9'*Si*-*exo*-*Re*-29**	-1536.654385	0.544726	-1536.079815	-1536.167777	-1537.098707	
**C9'*Si*-TS-*endo*-*Si***	-1536.555932	0.539086	-1535.986565	-1536.074133	-1536.999488	-540.32
**C9'*Si*-*endo*-*Si*-29**	-1536.649277	0.544297	-1536.076094	-1536.160257	-1537.090358	
**C9'*Si*-TS-*exo*-*Si***	-1536.573241	0.538573	-1536.005003	-1536.091215	-1537.018205	-430.77
**C9'*Si*-*exo*-*Si*-29**	-1536.646735	0.544431	-1536.072814	-1536.158335	-1537.08795	
**C9'*Re*-TS-*endo*-*Si*1**	-1536.559792	0.5394	-1535.989998	-1536.079864	-1537.004279	-436.54
**C9'*Re*-Int-*endo***	-1536.563931	0.540938	-1535.992654	-1536.080856	-1537.007988	
**C9'*Re*-TS-*endo*-*Si*2**	-1536.563924	0.540954	-1535.993475	-1536.078967	-1537.00793	-34.27
**C9'*Re*-*endo*-*Si*-29**	-1536.633466	0.54509	-1536.059259	-1536.143977	-1537.074326	
**C9'*Re*-TS-*exo*-*Si***	-1536.554003	0.539143	-1535.984674	-1536.07181	-1536.99712	-589.91
**C9'*Re*-*exo*-*Si*-29**	-1536.626367	0.544846	-1536.053187	-1536.13621	-1537.067011	
**C9'*Re*-TS-*endo*-*Re***	-1536.560141	0.539784	-1535.990109	-1536.077517	-1537.002598	-539.5
**C9'*Re*-*endo*-*Re***	-1536.666423	0.544692	-1536.091974	-1536.178719	-1537.108618	
**C9'*Re*-TS-*exo*-*Re***	-1536.566634	0.539533	-1535.997205	-1536.08332	-1537.010302	-535.92
**C9'*Re*-*exo*-*Re*-29**	-1536.667921	0.544565	-1536.09379	-1536.179271	-1537.110209	

[a] Electronic energy; [b] Zero point energy; [c] Enthalpy; [d] Gibbs free energy; [abcd] Energies calculated with M06-2X/6-31G(d) level of theory in gas phase as in 298.15 Kelvin and 1.0 atm [e] Electronic energy with SMD solvation model.

Supplementary Tables 14a-c Energies, enthalpies, and free energies (in Hartrees) of optimized structures for $Zn(BH_4)_2$ reduction of dione **31** at:

a) SMD(toluene)-M06-2X/6-311+G(d,p)-SDD // b3lyp/6-31G(d,p)-LANL2DZ level of theory.

Structure	E[a]	ZPE[b]	H[c]	G[d]	$E_{toluene}$[e]	Imaginary Frequency (cm^{-1})
**31**	-1651.818785	0.56669	-1651.217745	-1651.315907	-1651.624014	
**$Zn(BH_4)_2$**	-120.0749053	0.075846	-119.990454	-120.032591	-281.6707763	
**Int-α-OH-1**	-1771.90726	0.645632	-1771.218734	-1771.336922	-1933.322496	
**TS-α-OH**	-1771.887125	0.644867	-1771.200784	-1771.314416	-1933.302619	-293.68
**Int-α-OH-2**	-1771.897467	0.646922	-1771.208042	-1771.322793	-1933.313868	
**Zn-α-OH**	-1771.943114	0.651577	-1771.250762	-1771.360486	-1933.365116	
**Int-β-OH-1**	-1771.908849	0.645972	-1771.220175	-1771.33626	-1933.32236	
**TS-β-OH**	-1771.885647	0.64507	-1771.199181	-1771.312546	-1933.299543	-296.49
**Zn-β-OH**	-1771.949325	0.651133	-1771.257179	-1771.369438	-1933.36909	

[a] Electronic energy; [b] Zero point energy; [c] Enthalpy; [d] Gibbs free energy; [abcd] Energies calculated with b3lyp/6-31G(d,p) level of theory in gas phase as in 298.15 Kelvin and 1.0 atm [e] Electronic energy with SMD solvation model

b) SMD(toluene)-M06-2X/6-311+G(d,p)-SDD // M06-2X/6-31G(d,p)-LANL2DZ level of theory.

Structure	E[a]	ZPE[b]	H[c]	G[d]	$E_{toluene}$[e]	Imaginary Frequency (cm^{-1})
**31**	-1651.182873	0.574078	-1650.575327	-1650.670277	-1651.627279	
**$Zn(BH_4)_2$**	-119.8592666	0.076881	-119.773991	-119.813775	-281.6678246	
**Int-α-OH-1**	-1771.084166	0.654365	-1770.389073	-1770.499605	-1933.326081	
**TS-α-OH**	-1771.071792	0.654634	-1770.377411	-1770.483996	-1933.306482	-302.34
**Int-α-OH-2**	-1771.086274	0.656909	-1770.388641	-1770.497829	-1933.31973	
**Zn-α-OH**	-1771.139521	0.660385	-1770.439542	-1770.545636	-1933.370681	
**Int-β-OH-1**	-1771.083171	0.655414	-1770.38625	-1770.498824	-1933.327299	
**TS-β-OH**	-1771.065941	0.653519	-1770.371985	-1770.481773	-1933.301031	-325.51
**Int- β -OH-2**	-1771.071255	0.655777	-1770.374407	-1770.48629	-1933.310912	
**Zn-β-OH**	-1771.134716	0.660089	-1770.434626	-1770.543813	-1933.372651	

[a] Electronic energy; [b] Zero point energy; [c] Enthalpy; [d] Gibbs free energy; [abcd] Energies calculated with M06-2X/6-31G(d,p) level of theory in gas phase as in 298.15 Kelvin and 1.0 atm [e] Electronic energy with SMD solvation model

c) SMD(toluene)-M06/6-311+G(d,p)-SDD // M06/6-31G(d,p)-LANL2DZ level of theory.

Structure	E[a]	ZPE[b]	H[c]	G[d]	$E_{toluene}$[e]	Imaginary Frequency (cm^{-1})
**31**	-1650.77623	0.568267	-1650.174839	-1650.26747	-1651.163707	
**$Zn(BH_4)_2$**	-119.8592678	0.076893	-119.773972	-119.813795	-281.6310335	
**Int-α-OH-1**	-1770.666927	0.649302	-1769.977124	-1770.084724	-1932.825941	
**TS-α-OH**	-1770.650408	0.64719	-1769.963559	-1770.069175	-1932.799842	-353.23
**Int-α-OH-2**	-1770.666015	0.649184	-1769.976319	-1770.08364	-1932.816299	
**Zn-α-OH**	-1770.713389	0.653634	-1770.020543	-1770.12452	-1932.859097	
**Int-β-OH-1**	-1770.665672	0.648455	-1769.977319	-1770.083634	-1932.827095	
**TS-β-OH**	-1770.648335	0.645834	-1769.962389	-1770.069646	-1932.796594	-291.31
**Int- β -OH-2**	-1770.653919	0.648386	-1769.96452	-1770.075009	-1932.80879	
**Zn-β-OH**	-1770.710789	0.653308	-1770.018076	-1770.123701	-1932.864669	

[a] Electronic energy; [b] Zero point energy; [c] Enthalpy; [d] Gibbs free energy; [abcd] Energies calculated with M06/6-31G(d,p) level of theory in gas phase as in 298.15 Kelvin and 1.0 atm [e] Electronic energy with SMD solvation model

I. Supplementary References:

[1] M. Shindo, Y. Sato, T. Yoshikawa, R. Koretsune, K. Shishido, Stereoselective Olefination of Unfunctionalized Ketones via Ynolates, *J. Org. Chem.*, **69**, 3912 – 3916 (2004).

[2] M. Shindo, T. Yoshikawa, Y. Itou, S. Mori, T. Nishii, K. Shishido, Heteroatom-Guided Torquoselective Olefination of a-Oxy and a-Amino Ketones via Ynolates, *Chem. Eur. J.* **12**, 524 – 536 (2006).

[3] M. Shindo, K. Matsumoto, K. Shishido, Generation of Ynolate and Z-Selective Olefination of Acylsilanes: (*Z*)-2-Methyl-3-Trimethyl- silyl-2-Butenoic Acid, *Org. Syn.*, **84**, 11 – 21 (2007).

[4] S. Qian, G. Zhao, Enantioselective total synthesis of (+)-sarcandralactone A, *Tetrahedron*, **69**, 11169-11173 (2013).

[5] J. Kawabata, E. Fukushi, J. Mizutani, Sesquiterpene dimers from *Chloranthus japonicas*, *Phytochemistry*, **39**, 121 – 125 (1995).

[6] J. Kawabata, Y. Fukushi, S. Tahara, J. Mizutani, Shizukaol a, a sesquiterpene dimer from *Chloranthus japonicas*, *Phytochemistry*, **29**, 2332 – 2334 (1990).

[7] Gaussian 09, Revision E.01, M. J. Frisch, G. W. Trucks, H. B. Schlegel, G. E. Scuseria, M. A. Robb, J. R. Cheeseman, G. Scalmani, V. Barone, B. Mennucci, G. A. Petersson, H. Nakatsuji, M. Caricato, X. Li, H. P. Hratchian, A. F. Izmaylov, J. Bloino, G. Zheng, J. L. Sonnenberg, M. Hada, M. Ehara, K. Toyota, R. Fukuda, J. Hasegawa, M. Ishida, T. Nakajima, Y. Honda, O. Kitao, H. Nakai, T. Vreven, J. A. Montgomery, Jr., J. E. Peralta, F. Ogliaro, M. Bearpark, J. J. Heyd, E. Brothers, K. N. Kudin, V. N. Staroverov, R. Kobayashi, J. Normand, K. Raghavachari, A. Rendell, J. C. Burant, S. S. Iyengar, J. Tomasi, M. Cossi, N. Rega, J. M. Millam, M. Klene, J. E. Knox, J. B. Cross, V. Bakken, C. Adamo, J. Jaramillo, R. Gomperts, R. E. Stratmann, O. Yazyev, A. J. Austin, R. Cammi, C. Pomelli, J. W. Ochterski, R. L. Martin, K. Morokuma, V. G. Zakrzewski, G. A. Voth, P. Salvador, J. J. Dannenberg, S. Dapprich, A. D. Daniels, Ö. Farkas, J. B. Foresman, J. V. Ortiz, J. Cioslowski, and D. J. Fox, Gaussian, Inc., Wallingford CT, 2013.

[8] Y. Zhao, D. G. Truhlar, The M06 suite of density functionals for main group thermochemistry, thermochemical kinetics, noncovalent interactions, excited states, and transition elements: two new functionals and systematic testing of four M06-class functionals and 12 other functionals, *Theor. Chem. Acc.*, **120**, 215 – 241 (2008).

[9] Y. Zhao, D. G. Truhlar, A new local density functional for main-group thermochemistry, transition metal bonding, thermochemical kinetics, and noncovalent interactions, *J. Chem. Phys.*, **125**, 194101 – 194118 (2006).

[10] Y. Zhao, D. G. Truhlar, Density functionals with broad applicability in chemistry, *Acc. Chem. Res.*, **41**, 157 – 167 (2008).

[11] A. V. Marenich; C. J. Cramer, D. G. Truhlar, Universal solvation model based on solute electron density and on a continuum model of the solvent defined by the bulk dielectric constant and atomic surface tensions, *J. Phys. Chem. B.* **113**, 6378 – 6396 (2009).

[12] C. Lee, W. Yang, R. G. Parr, Development of the Colle-Salvetti correlation-energy formula into a functional of the electron density, *Phys. Rev. B.* **37**, 785 – 789 (1988).

[13] A. D. Becke, Density-functional thermochemistry. III. The role of exact exchange, *J. Chem. Phys.* **98**, 5648 – 5652 (1993).

[14] R. G. Parr, W. Yang, Density-Functional Theory of the Electronic Structure of Molecules, *Annu. Rev. Phys. Chem.* **46**, 701 – 728 (1995).

[15] W. R. Wadt and P. J. Hay, Ab initio effective core potentials for molecular calculations. Potentials for main group elements Na to Bi, *J. Chem. Phys*., **82**, 284 – 298 (1985).

[16] M. Dolg, U. Wedig, H. Stoll, H. Preuss. Energy-adjusted ab initio pseudopotentials for the first row transition elements, *J. Chem. Phys.* **86**, 866 – 872 (1987).

[17] D. Andrae, U. Häußermann, M. Dolg, H. Stoll, H. Preuß, Energy-adjusted*ab initio* pseudopotentials for the second and third row transition elements, *Theor. Chem. Acc.* **77,** 123 – 141 (1990).

[18] CYLview, 1.0b; C. Y. Legault, Université de Sherbrooke, 2009, http://www.cylview.org.

[19] L. R. Domingo, J. A. Sáez, Understanding the mechanism of polar Diels–Alder reactions, *Org. Biomol. Chem*. **7**, 3576 – 3583 (2009).

[20] L. R. Domingo A new C–C bond formation model based on the quantum chemical topology of electron density. *RSC Adv*. **4**, 32415-32428 (2014).

[21] D.H. Ess, J. O. Gavin, K. N. Houk, Conceptual, Qualitative, and Quantitative Theories of 1,3-Dipolar and Diels–Alder Cycloadditions Used in Synthesis, *Adv. Synth. Catal*. **348**, 2337 – 2361 (2006).

[22] R. G. Parr, L. von Szentpaly and S. Liu, Electrophilicity Index, *J. Am. Chem. Soc.*, **121**,1922–1924 (1999).

[23] NBO 6.0. E. D. Glendening, J. K. Badenhoop, A. E. Reed,J. E. Carpenter, J. A. Bohmann, C. M. Morales, C. R. Landis, F. Weinhold (Theoretical Chemistry Institute, University of Wisconsin, Madison, WI, 2013); http://nbo6.chem.wisc.edu/.

[24] D. N. Ess, K. N. Houk, Distortion/interaction energy control of 1,3-dipolar cycloaddition reactivity, *J. Am. Chem. Soc*. **129**, 10646 – 10647 (2007).

[25] R. S. Paton, S. Kim, A. Ross, S. J. Danishefsky, K. N. Houk, Experimental Diels-Alder reactivities of cycloalkenones and cyclic dienes explained through transition-state distortion energies, *Angew. Chem. Int. Ed*. **50**, 10366 – 10368 (2011).

[26] L. R. Domingo, J. Sáez, R. J. Zaragozá, M. Arno, Understanding the participation of quadricyclane as nucleophile in polar [2sigma + 2sigma + 2pi] cycloadditions toward electrophilic pi molecules, *J. Org. Chem*. **73**, 8791 – 8799 (2008).
