# Supplementary material for: Total syntheses of shizukaols A and E
Source: Nat Commun. 2018 Oct 2;9:4040. doi: 10.1038/s41467-018-06245-7 (PMC6168560; doi:10.1038/s41467-018-06245-7)
Supplement: Supplementary file 3 — Description of Additional Supplementary Files [file 41467_2018_6245_MOESM3_ESM.pdf]

Description of Additional Supplementary Files

File Name: Supplementary Data 1
Description: This is the dataset for calculation part.
